# Genome-wide evolutionary characterization and analysis of bZIP transcription factors and their expression profiles in response to multiple abiotic stresses in *Brachypodium distachyon*

**DOI:** 10.1186/s12864-015-1457-9

**Published:** 2015-03-22

**Authors:** Xiang Liu, Zhaoqing Chu

**Affiliations:** Shanghai Chenshan Plant Science Research Center, Shanghai Chenshan Botanical Garden, Shanghai Key Laboratory of Plant Functional Genomics and Resources, Shanghai Institutes for Biological Sciences, Chinese Academy of Sciences, 3888 Chenhua Road, 201602 Shanghai, Songjiang China

**Keywords:** Abiotic stresses, Basic leucine zipper (bZIP), *Brachypodium distachyon*, Evolutionary comparisons, Gene expression, Heavy metals

## Abstract

**Background:**

Plant basic leucine zipper (bZIP) transcription factors are one of the largest and most diverse gene families and play key roles in regulating diverse stress processes. *Brachypodium distachyon* is emerging as a widely recognized model plant for the temperate grass family and the herbaceous energy crops, however there is no comprehensive analysis of bZIPs in *B. distachyon*, especially those involved in stress tolerances.

**Results:**

In this study, 96 bZIP genes (*BdbZIP*s) were identified distributing unevenly on each chromosome of *B. distachyon*, and most of them were scattered in the low CpG content regions. Gene duplications were widespread throughout *B. distachyon* genome. Evolutionary comparisons suggested *B. distachyon* and rice’s bZIPs had the similar evolutionary patterns. The exon splicing in BdbZIP motifs were more complex and diverse than those in other plant species. We further revealed the potential close relationships between *BdbZIP* gene expressions and items including gene structure, exon splicing pattern and dimerization features. In addition, multiple stresses expression profile demonstrated that *BdbZIP*s exhibited significant expression patterns responding to 14 stresses, and those responding to heavy metal treatments showed opposite expression pattern comparing to the treatments of environmental factors and phytohormones. We also screened certain up- and down-regulated *BdbZIP* genes with fold changes ≥2, which were more sensitive to abiotic stress conditions.

**Conclusions:**

*BdbZIP* genes behaved diverse functional characters and showed discrepant and some regular expression patterns in response to abiotic stresses. Comprehensive analysis indicated these *BdbZIP*s’ expressions were associated not only with gene structure, exon splicing pattern and dimerization feature, but also with abiotic stress treatments. It is possible that our findings are crucial for revealing the potentialities of utilizing these candidate *BdbZIPs* to improve productivity of grass plants and cereal crops.

**Electronic supplementary material:**

The online version of this article (doi:10.1186/s12864-015-1457-9) contains supplementary material, which is available to authorized users.

## Background

In plants, the leucine zipper (bZIP) transcription factors are one of the largest and most conserved gene families and play key roles in regulating diverse biological processes [1-5]. The bZIP domain contains about 60 to 80 amino acids and characteristically harbors two distinct function regions: a highly conserved basic region N-x7-R/K-x9 and a less conserved leucine zipper coiled-coil motif [6]. And the basic region and leucine zipper coiled-coil motif region was linked by a hinge region. The bZIP proteins bind to DNA by forming heterotypic or homotypic complexes [7,8]. The basic region is responsible for nuclear localization and DNA binding specifically, and the following leucine zipper motif consisting of several repeats of leucine or other hydrophobic amino acids and grant for recognition and dimerization specificity [9]. The intron patterns within the basic and the hinge region are very important for their functional evolution due to different status of exon splicing in these regions. In plant species such as rice, maize and Arabidopsis, the patterns of those motifs exhibited regular conservation and diversity [10,11]. The bZIP proteins are dimerized transcription factors in all eukaryotes, and the leucine zipper is responsible for the dimerization of bZIP proteins. The rules of dimerization specificity for bZIP proteins have been depicted [12-14]. Depend upon the basis of the presence of attractive or repulsive interhelical g↔e electrostatic interactions and the presence of polar or charged amino acids in the a and d positions of the hydrophobic interface of the leucine zipper region, dimerization specificity of bZIP proteins in plant species such as Arabidopsis, maize and rice have been predicted [10,11,15].

The evolution of genetic network complexities in flowering plants has revealed the important roles of regulatory transcription factor evolution to physiological variation among species [16,17]. As one of important transcription factor family, the plant bZIP transcription factors play pivotal roles in developmental processes and multiple stresses in response to environmental tolerance. The ancestor of green plants possessed four bZIP genes functionally involved in oxdative stress and unfolded protein responses that are bZIP-mediated processes in all eukaryotes [18]. Furthermore, bZIP genes regulate diverse biological processes such as seed development, flower maturation, pathogen defense, and light and stress signaling [3,6]. Fundamentally, various transcription factors had been observed to regulate the ABA-responsive gene expression [19,20]. The transcriptional drought, cold, and salinity stress gene expression have been concluded [21]. So far, members of bZIP transcription factors have been identified or predicted in most of plant species analyzed [22-26]. In cucumber, 64 bZIPs were observed and all of the select genes displayed down-regulated with PEG treatment [24]. In maize, *ZmbZIP17* functions as an endoplasmic reticulum stress transducer and interact with ABA-responsive cis-elements (ABRE) [5]. In rice, plenty of *OsbZIP*s displayed different expression patterns when dealed with cold or salt stresses [10]. *OsbZIP71* was strongly induced in ABA-mediated drought and salt tolerance in rice [27]. *OsbZIP46* expression was strongly induced by drought, heat, and ABA, and functions as a positive regulator of ABA signaling and drought stress tolerance of rice depending on its activation [28]. *OsbZIP52/RISBZ5* could function as a negative regulator in cold and drought stress environments [29]. A bZIP gene *ABI5* played an important role in ABA-arrested seed germination, was robustly associated with the flower transition in Arabidopsis [30]. A bZIP gene *ThbZIP1* from *Tamarix hispida* in response to abiotic stresses had been characterized and showed to have an increased tolerance to drought and salt. Microarray analysis had been shown that many ROS scavenging genes were up-regulated by *ThbZIP1* under salt stress conditions [31]. *MabZIP3* was isolated from banana fruit, it was responsive to MeJA, ABA, and chilling stress [32]. As the most dangerous pollutions, heavy metals had been regarded as the new stress factors affecting the growth of plants. Foods contaminated with heavy metal tolerance profile of different native or gene-modified plant species had been applied [33-39]. Though studies had shown that bZIP transcription factors played key roles when plants grew under environmental factors and phytohormones, there was few research of bZIP genes study in heavy metal stresses so far.

*B. distachyon* is a new emerging model plant of Poaceae family and the first species of sequenced grass subfamily Pooideae [40]. Due to its high efficiency for genetic manipulation and compact genome, *B. distachyon* has become more crucial in applied functional genomics [41]. Researches on *B. distachyon* are moving forward rapidly, and the research field has covered grain development and starch deposition, biotic and abiotic stress responses, and biofuel production [42-44]. Global gene expression in *B. distachyon* had revealed extensive network plasticity in response to abiotic stress [45]. Evolutionary studies on bZIP gene families had been shown that a shifting landscape of biochemical functions related to signaling and gene expression contributed to species diversity [46]. Although these studies reported were involved in various plant species, none of researches were associated with the evolution and the molecular biology of stress in detail, especially in the grain model plant *B. distachyon*. There is no investigation of bZIP transcription factors in *B. distachyon* so far*.* Understanding the detailed evolutionary history of *BdbZIPs* and their corresponding functions in stress biology is of great importance in *B. distachyon*.

In this study, we identified *BdbZIP* genes genome-widely in *B.distachyon* and further investigated their chromosomal localization and evolutionary relationship with their counterparts from monocot *O. sativa* and dicot *A. thalinana.* We analyzed their exons splicing of basic and hinge region of bZIP domain, which are very important for bZIP functional evolution. We also characterized dimerization pattern within the leucine zipper motif and gene structures, and obtained tissue-specific gene expression profile and genes expression profile responding to multiple stresses including environmental factors, phytohormones, and heavy metals. This study increased our understanding of *BdbZIP* family genes associated with stress adaptation and tolerance, which was crucial for further study to improve the productivity of grass plants and cereal crops.

## Methods

### Plant growth condition and treatments

The seeds of *B. distachyon* Bd21-3 were surface sterilized with 20% NaOCl and planted on 0.6% agar containing 0.5× Murashige and Skoog and 0.3% Sucrose. Plants were grown at 22°C under 16-h-light/8-h-dark conditions and the light intensity was 120 μm m^−2^ s^−1^. As for stress expression analysis, 2-week-old seedlings were treated with 3 major treatment groups including group 1-environmental factors ( cold, heat, H_2_O_2_, PEG, and NaCl), group 2-heavy metals (Cu, Zn, Mn, Cd, and Pb) and group 3-phytohormones (SA, 6-BA, ABA, and MeJA) (As for detailed treatments, please refer to Additional file 1: Table S1).

### bZIP sequence extraction and structure analysis

The sequences of *B. distachyon* bZIP genes were obtained from BGD (Brachypodium Genome Database) (http://Brachypodium.org) and Plant Transcription Factor Database verition 3.0 (Plant-TFDB 3.0) (http://planttfdb.cbi.pku.edu.cn) [47]. The Arabidopsis and rice bZIP genes were retrieved from The Arabidopsis Information Resource (TAIR), Plant-TFDB and National Rice Gene Database (http://www.ricedata.cn/gene). The status of intron and exon were annotated according to the database. All bZIP domains were verified by SMART (http://smart.embl-heidelberg.de) and Pfam (http://pfam.sanger.ac.uk). All BdbZIP motifs were analyzed by MEME (http://meme.nbcr.net/meme). The limits of minimum width, maximum width and maximum number of motifs were specified as 10, 50 and 50 respectively. Fifteen motifs including bZIP domain were finally verified with the low E-value (<−43). The motifs were numbered according to their order displayed in MEME.

### Evolutionary analysis

Phylogenetic and molecular evolutionary analyses were calculated in MEGA 5.0 package by using the *p*-distance model, and the Neighbor-joining statistical method followed by 1000 bootstrap replications were applied [48]. The bZIP protein sequences of *B. distachyon*, Arabidopsis and rice were loaded into MEGA 5.0. In addition, homology searches were performed in rice and Arabidopsis with BLAST, and the SCORE value and E-value were taken into account for judging the homologous genes. *bZIP* gene duplication events among *B. distachyon*, Arabidopsis and rice were analyzed through Plant Genome Duplication Database (http://chibba.agtec.uga.edu/duplication) [49]. The Ka and Ks values were listed as Additional file 1: Table S2. The data of the phylogenetic tree was deposited in Treebase Web (Accession URL: http://purl.org/phylo/treebase/phylows/study/TB2:S17110).

### Chromosomal distribution and duplication of *BdbZIP*s

The location information of each *BdbZIP* gene on each chromosome was detected from BGD (Brachypodium Genome Database). The genetic linkage map was constructed with MapDraw [50]. The number of CpG in every 100 kb scale was measured and the status of CpG content was constructed by PermutMatrix. Every duplicated *BdbZIP* gene pairs were put in same bracket.

### Expression data analyses

To analyze tissue (root, stem, leaf and early spikelet) specific expression, 2-week-old seedlings and early spikelets from 5-week-old of *B. distachyon* were sampled. Total RNA was extracted as described previously [51]. The cDNA was synthesized with 3.0 μg of total RNA by using PrimeScript Reverse Transcriptase (TAKARA). The reaction mixtures were diluted 20 times with distilled water and used as templates for quantitative real-time PCR. The primers used in this paper were listed in Additional file 1: Table S3. The qPCR was conducted and repeated three times. The reaction condition was as follows: 95°C for 3 mins, 40 cycles of 95°C for 10 s, 55°C for 30 s. The expression profiles were calculated with –ΔΔCT values. Fold changes were also calculated with the formula “fold change = 2^–ΔΔCT^”. Expression data and hierarchical clustering analysis of all the samples were carried out using PermutMatrix 1.9.3, and shown with green-red gradient. The up-regulated genes were defined as a fold change of ≥ 2 with *p*-value <0.05 and marked with red color, and a fold change of ≤ 0.5 were defined as down-regulated genes with *p*-value <0.05 and marked with green color. All qPCR data were submitted to NCBI GEO dataset. The accession number is GSE66458.

## Results and discussion

### Identification of bZIP genes in plants

**Figure 1:**
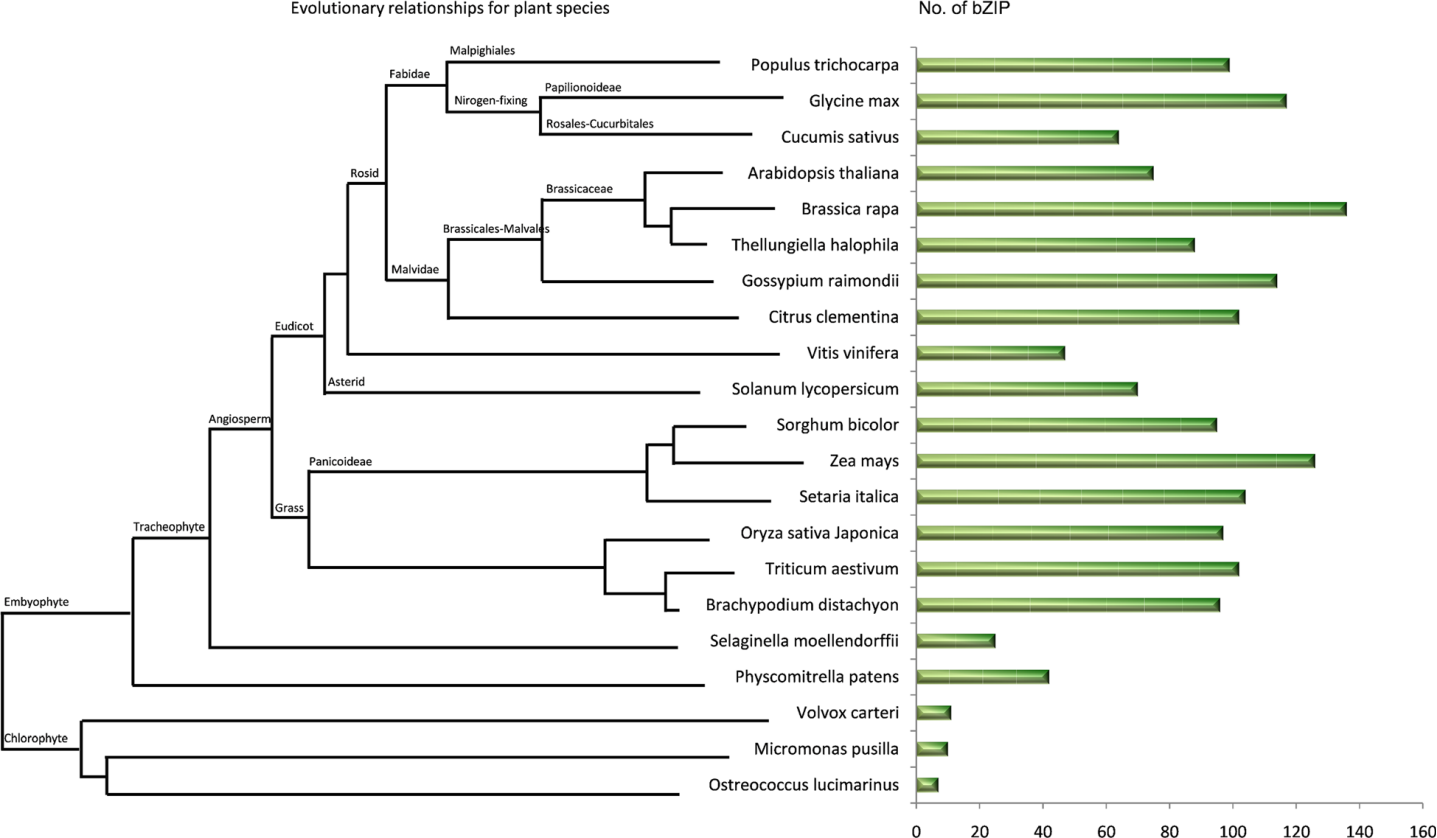
**bZIP family transcription factors distribution in plant species.**

To investigate the evolution of bZIP genes in plant species, 21 plants from low to high grade were selected and the numbers of their bZIP genes were identified. The green plants were covered from Chlorophyta to Embyophyta subkingdoms. The evolutionary tree for these species was constructed as in Figure 1. After searching BGD and TFDB database and further validated by SMART and Pfam domain analysis, 96 unique bZIP genes were identified from *B. distachyon*.The number of bZIP genes in plants examined varied from 7 to 136. Some plant species maintained the high number of bZIP genes, which might contribute to their numerous tandem duplications and large-scale segmental duplications [52]. Except *Zea mays* had a little bit more (126). bZIP genes, the plant species sub-grouped in monocot clades had approximately the close numbers (95–104), which might due that the species in this clade shared the common ancestor of Poaceae and had similar whole genome duplication [53]. *B. distachyon* fell into monocot and shared the same clade with *Triticum aestivum*, which was consistent with the notion that these two species had closer relationships. Comparing to Embrophyte, the Chlorophyta species had much less bZIPs. We inferred that high grade plants had stronger environment adaptation abilities with more bZIP genes. Speculatively, during evolution, *B. distachyon* and other monocot species maintained the high number of bZIP genes might contribute to large-scale segmental duplications.

### Chromosomal location and duplication of *BdbZIP* genes

**Figure 2:**
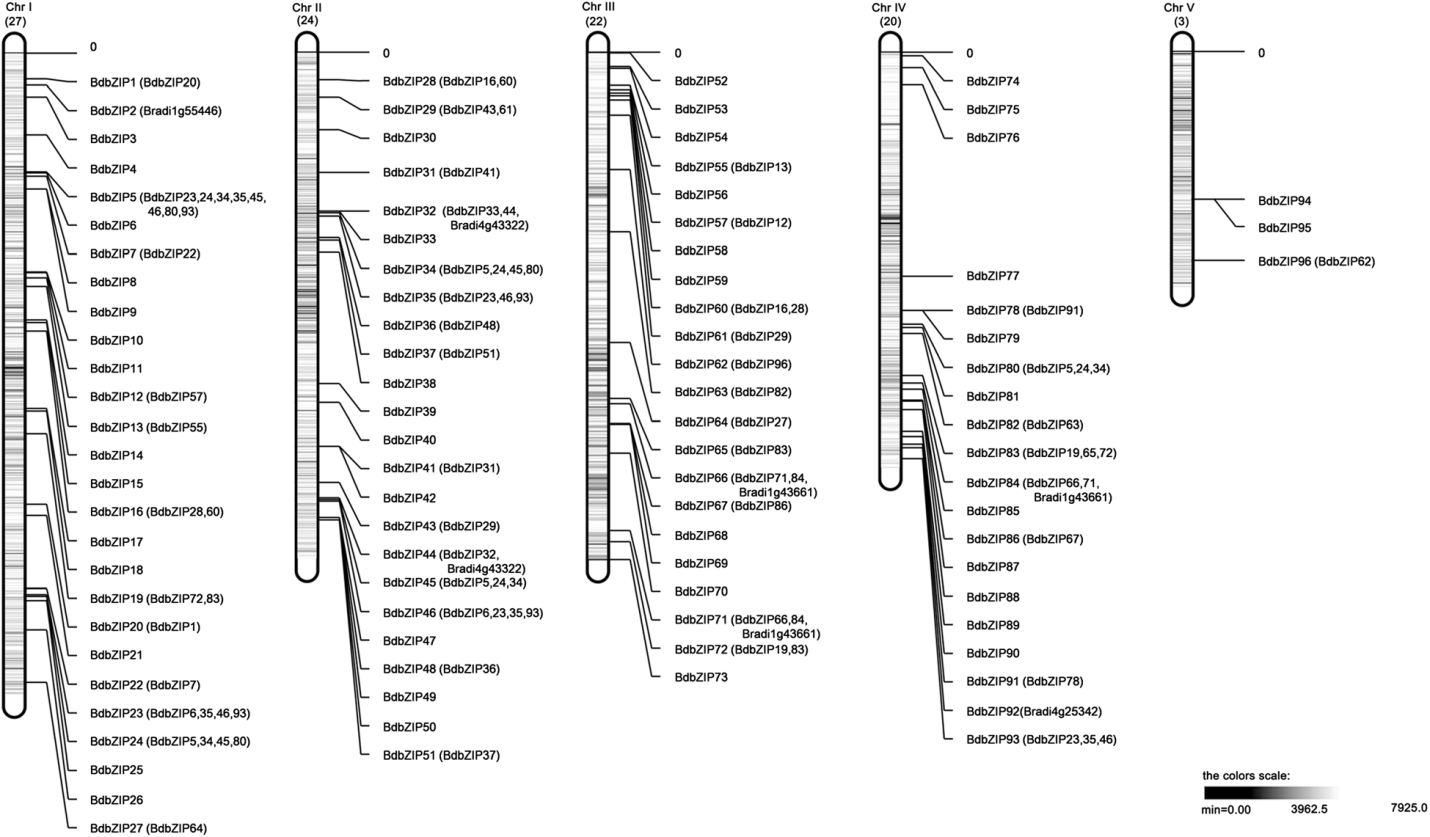
**Chromosomal locations and regional duplication for** ***B. distachyon*** **bZIP genes with CpG profile.** *BdbZIP* genes are numbered from 1 to 96. The chromosome information of *BdbZIP*s was given in Additional file 1: Table S4. *B. distachyon* CpG distributions were calculated and given in Additional file 1: Table S5.

Although the basic leucine zipper transcription factors are widely distributed in all plant kingdoms, the chromosomal location of bZIP genes in *B. distachyon* is still unclear. Based on our analysis, bZIP genes existed in *B. distachyon* were designated as *BdbZIP1-96* according to their top-to-bottom position on chromosomes from I to V (Figure 2 and Additional file 1: Table S4). *BdbZIP* genes were scattered on each chromosome, but their distributions were obviously not uniform in density. Certain chromosome regions had relatively high density of *BdbZIP* genes. Except for the smallest chromosome V, there were one or two *BdbZIP* clusters on each chromosome.

To probe the potential evolutionary mechanisms of *BdbZIP* gene family, according to described in [50] both tandem and segmental duplication events in terms of intragenome were examined in *B. distachyon*. It was observed that gene duplications were widespread throughout *B. distachyon* lineages (Figure 2). About 66% of *BdbZIP* genes were found to be duplicated at a maximal length of 100 kb. All the duplicated genes were confined to chromosomal block duplication and none of *BdbZIP* genes were found to be arranged in tandem form. The phenomena were very similar to the bZIP gene family in rice [10]. This result further revealed why *B. distachyon* had high amount of bZIP genes.

Much evidence showed that CpG content and distribution might have influence on variability in chromatin structure and gene distribution [54]. Based on this, we infered CpG content might affect the functional properties of *BdbZIP*s. To dig into the evolutionary relationships between *BdbZIP*s distribution and CpG content, we also counted the CpG content in the whole *B. distachyon* genome (Additional file 1: Table S5). The landscape of CpG content was generated and integrated to the genetic linkage map (Figure 2). It was observed that the distribution of CpG content was not uniform. Each chromosome had several relatively high CpG content regions (Figure 2). Except a few of *BdbZIP*s (*BdbZIP5-7*, *32*–*35*, *65*, *66*, *73*, and *83–87*) were distributed in the regions of high CpG content, most of *BdbZIP* genes were scattered in the regions of low CpG content. This finding showed that those *BdZIP*s located in the regions of low CpG content might have high frequent mRNA transcripts, which required to implement their functions.

### Phylogenetic and molecular evolutionary analysis of Bd*bZIP* genes

**Figure 3:**
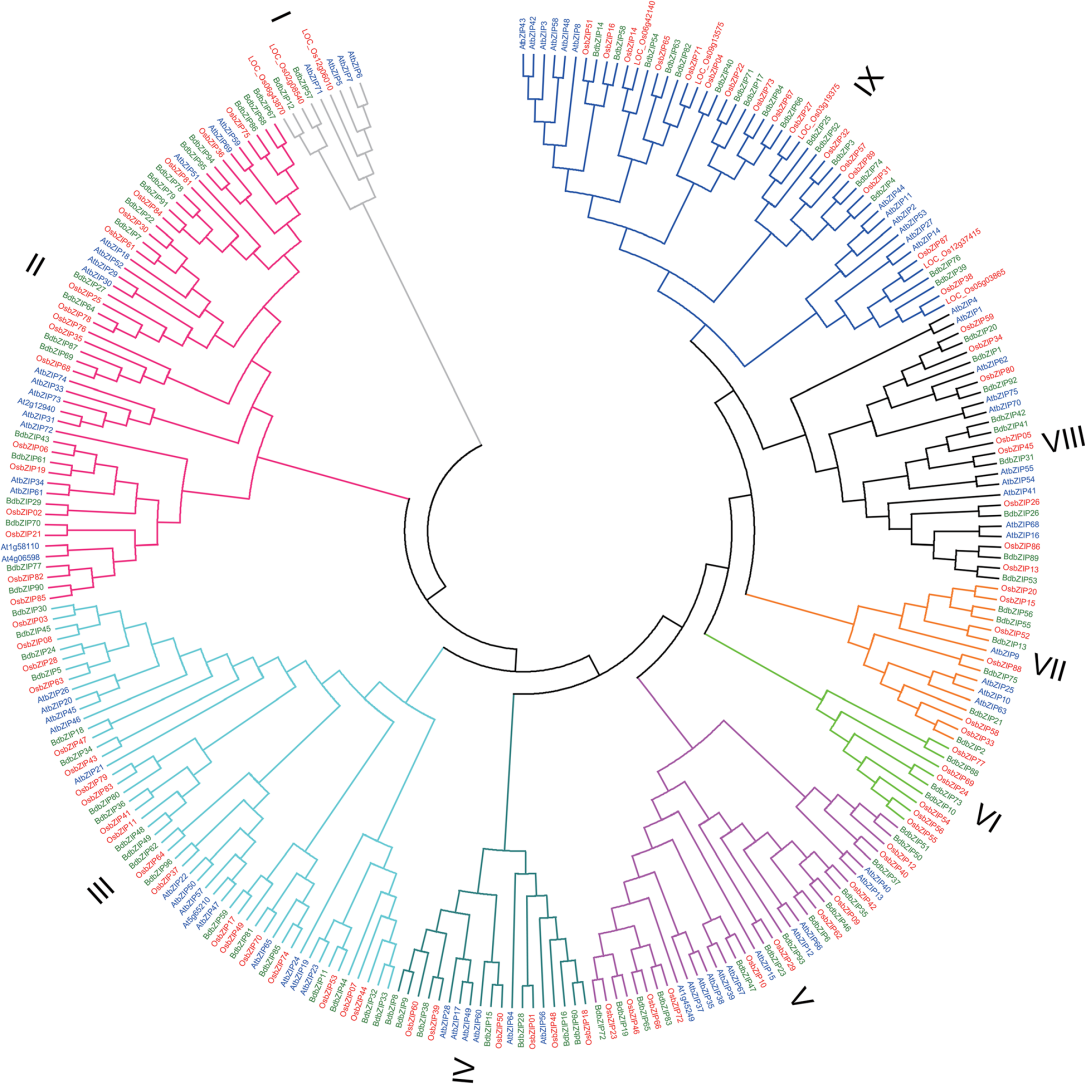
**Phylogenic analyses of bZIP proteins in** ***B. distachyon*** **,** ***O. sativa*** **and** ***A. thaliana*** **.** The phylogenetic analysis of bZIP proteins based on the bZIP protein sequences in *B. distachyon*, *O.sativa* and *A. thaliana*. Each clades is marked by one type of colors. The *B. distachyon*, *O. sativa* and *A. thaliana*. bZIPs proteins are marked by green, red and blue colors respectively. The phylogenetic tree was constructed by MEGA 5.0 Bootstrap values from 1000 replicates are indicated at each node.

Furthermore, to evaluate the evolutionary history of *BdbZIP* genes and relationships with other plant bZIP family genes, the bZIP proteins from other two model plants, Arabidopsis and rice, were performed for analyzing and comparing. A total of 268 bZIPs (Additional file 1: Table S6 and Additional file 1: Table S7) were de-tected and a phylogenetic tree was constructed (Figure 3). All bZIP proteins were grouped into 9 clades, designated as clade I to IX (Figure 3). It was observed that each clade had both *AtbZIP* and *OsbZIP* genes, which indicating that the evolution of bZIP genes was conservative between dicots and monocots. Most of the *BdbZIP* and *OsbZIP* genes were hierarchical clustered together in the same clades and exhibited closer relationship than those in *AtbZIP* genes (Figure 3). To identify the bZIP genes in terms of intragenome or cross-genome syntenic relationships among species *B. distachyon*, rice, and Arabidopsis, we also calculated the synonymous (Ks) and non-synonymous (Ka) substitution rates of bZIP genes among them [50]. We found that most of BdbZIP genes had syntenic regions in rice, but few in Arabidopsis (Additional file 1: Table S2). These results were consist-ent with the notion that *B. distachyon* and rice were monocots and their bZIP families might have the similar evolutionary patterns. However, it was still mentioned that some BdbZIP proteins were hierarchical clustered together with AtbZIP proteins in the same clade, the reason was that these BdbZIPs were expected to be orthologs of the AtbZIP proteins and they shared other additional conserved motifs outside of bZIP domains. Furthermore, homologous bZIP genes were detected in rice and Arabidopsis using BLAST tools. Seventy-two and forty-seven BdbZIPs were found to have homolo-gous bZIP genes in rice and Arabidopsis respectively (Additional file 1: Table S8). The comparative phylogenetic analysis and BLAST results showed that the bZIP genes in *B. distachyon* had closer relationships with rice than those in Arabidopsis.

### Gene structure analysis of *BdbZIP* genes

Alternative splicing events were spread in the whole *B. distachyon* genome [55]. As the overall pattern of intron position acted as an index to the phylogenetic relationships in a gene family evolution [56], so we also examined the intron and exon organization of *BdbZIP*s (Additional file 1: Table S4). It showed that most of *BdbZIP*s (81 of 96 *BdbZIP*s) containing introns, only 15 of total *BdbZIP* genes were intronless. As for the genes containing introns, the numbers of introns varied from 1 to 13. Diverse status of exon and intron splicing might be meaningful for *BdbZIP* gene evolution.

**Figure 4:**
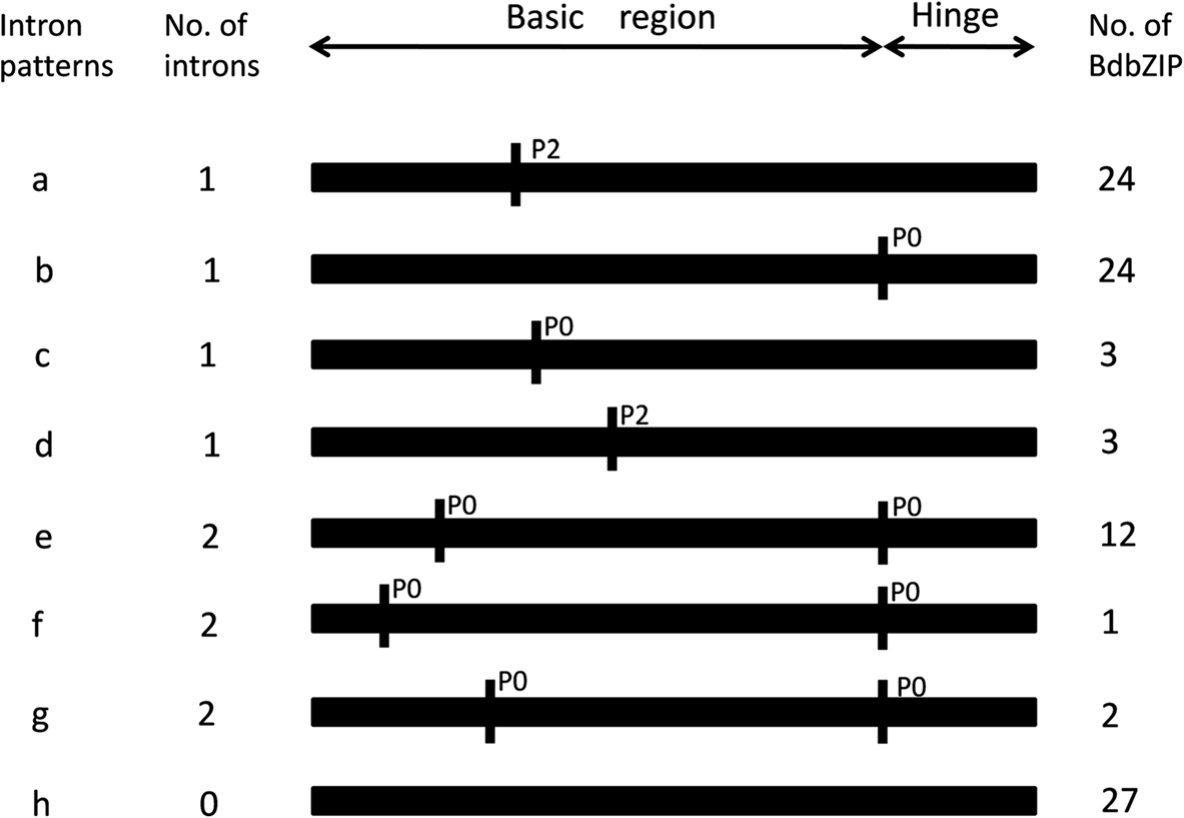
**Patterns of intron within the basic and hinge regions of the bZIP domains of BdbZIP proteins.** Intron patterns (a–h) are depicted. The number of introns and the number of BdbZIP proteins having a particular pattern are also indicated. P0 and P2 indicate the splicing phases of the basic and hinge regions of the bZIP domains. P0 represent the intron splicing site between codons, P2 means the intron splicing site locating between the second nucleotide and the third nucleotide in one codon.

In addition, the intron patterns within the basic and hinge region of the bZIP domain are most conserved and particularly important for their functional evolution. Any splicing change in the hinge region would change the code of the bZIP domain of the proteins and further affect their function. Among the 81 genes containing introns, 17 had 1–2 intron/introns in this region. Based on the intron presence, position, and splicing phase, *BdbZIP* genes were divided into 8 patterns, which were designated as pattern a to pattern h (Figure 4 and Additional file 2: Figure S1). The pattern a, b, e, and h were the most prevalent. Both pattern a and pattern b had one intron, pattern e had two introns and pattern h was intronless. But the pattern a had one intron in phase 2 (P2) in the basic region, whereas pattern b had an intron in phase 0 (P0) in the hinge region. Pattern c, d, f and g were uncommon. Compared with those in rice and maize [10,11], pattern f and pattern g were novel types. Pattern c and pattern d had one intron in P0 and P2 respectively. Pattern f and pattern g had two introns in P0. Pattern h was found in 27 *BdbZIP* genes, of which only 17 were intronless. The result showed that when the introns existed in the hinge region, the phase and position were conserved. However, when the introns were present in the basic regions, the positions of P0 and P2 were variable. The results showed that in *B. distachyon*, the splicing phase had remained conserved during the course of evolution of *BdbZIP* family genes, which were very consistent with those in rice [10]. In brief, our results showed that the intron patterns in the BdbZIP domains demonstrated more diverse than those in rice and maize.

### Dimerization properties of BdbZIP proteins

**Figure 5:**
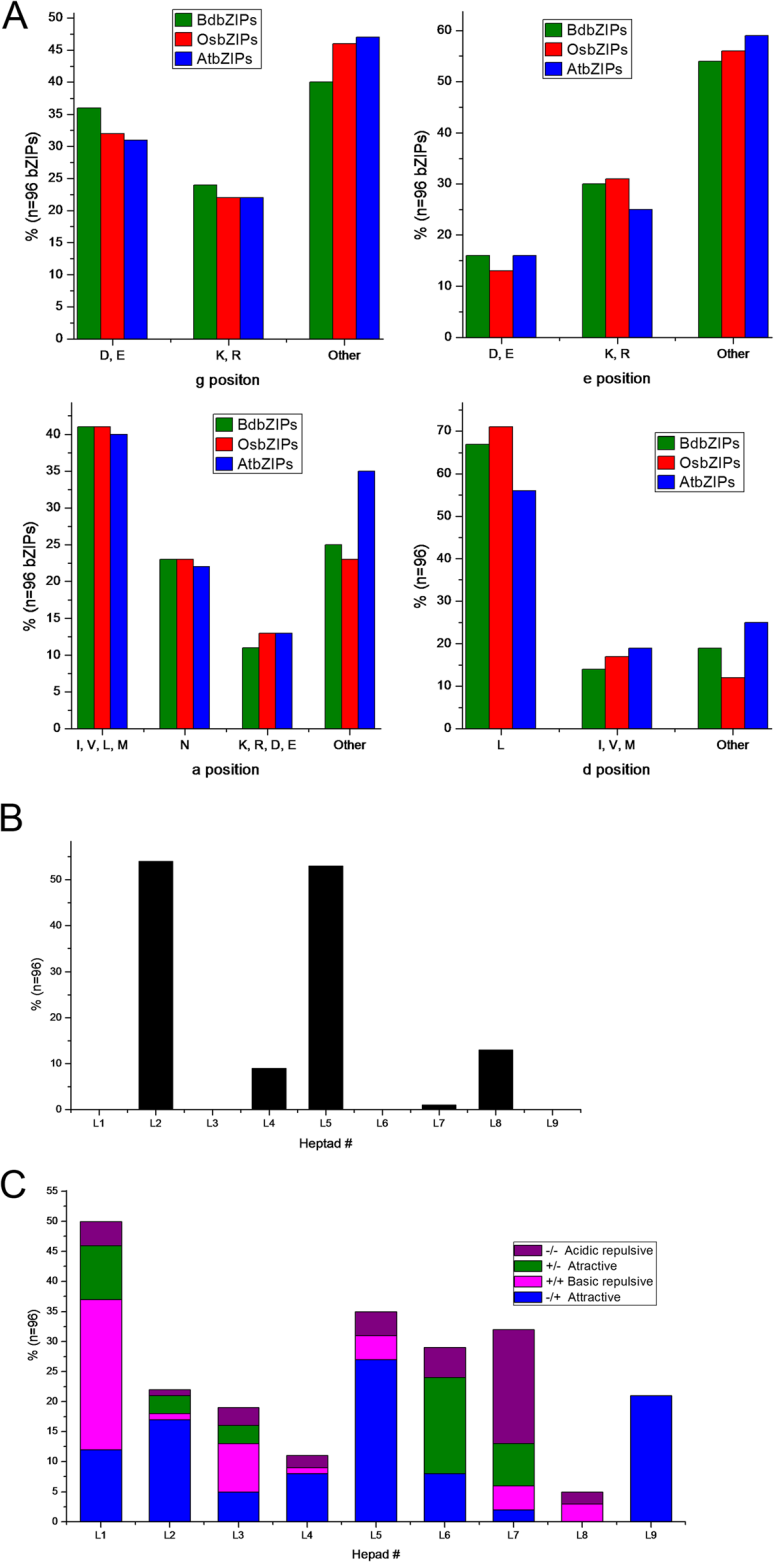
**Amino acid analysis at the g, e, a, and d positions of the leucine zippers. A**. Histogram of frequency of amino acids in the g, e, a, and d positions of the leucine zippers from BdbZIP, AtbZIP, and OsbZIP proteins. **B**. Histogram of the frequency of Asn residue in a position of the leucine zippers for all BdbZIP proteins. **C**. Histogram of the frequency of attractive or repulsive g↔e pairs per heptad for all BdbZIP proteins.

The amino acids at positions nearing leucine zipper interface of leucine zipper determined the dimerization stability and specificity [13-15]. To predict the dimerization specificity and stability of all BdbZIP proteins, we adopted the standard nomenclature for the amino acid positions in heptads to arrange manually leucine zipper regions [15]. The N-terminal and C-terminal boundaries of BdbZIP leucine zippers were demarcated following the criteria used for the bZIP proteins of Arabidopsis, rice and maize [10,11,15]. According to their positions, the amino acids in the heptad repeats in leucine zipper region were named as position a, b, c, d, e, f, and g (Additional file 3: Figure S2). The types of amino acids present at the g, e, a, and d positions in BdbZIP proteins were analyzed and compared with those of Arabidopsis and rice. We found that BdbZIPs had the lowest frequency of charged amino acids in these positions. This result showed that interactions between g↔e pairs might be more prominent in regulating specificity in BdbZIPs (Figure 5A). However, the frequency of charged amino acids of BdbZIPs and OsbZIPs at g and e positions had a closer proximity than that in AtbZIPs. This might due that BdbZIPs and OsbZIPs had closer evolutionary relationships. As Asns existing in a position contribute the most to dimerization specificity [57], we also examined the frequency of Asn in a position. About 23% of amino acids presenting at a position are Asns (Figure 5A), and about 55% of Asns in a position in heptad 2 and heptad 5 were observed in BdbZIPs (Figure 5B). The similar phenomena had been observed in AtbZIP, OsbZIPs and ZmbZIPs [10,11,15]. It was known that the leucine present in d position was responsible for dimerization stability [57], so the leucine in the d positions was calculated. The abundance of leucine of BdbZIPs in d positions was 68%, which was slightly lower than that in OsbZIPs (71%) and significantly greater than that in AtbZIPs (56%). The number of heptads was variable and ranged from four to nine (Additional file 3: Figure S2), and higher frequency of leucine was speculated to be responsible for dimer stability of long zippers.

To evaluate the contribution of charged residues responsible for dimerization properties of BdbZIPs, the frequency of attractive and repulsive g↔e pairs in each heptad of BdbZIP leucine zippers was computed and the corresponding histogram was demonstrated (Figure 5C). It was found that the frequency of interactive g↔e pairs was the maximum in the first heptad, with a sharp decrease in the next three heptads (L2, L3, and L4). Then the trend increased in the fifth heptad and decreased sharply in the eighth heptad. Moreover, only repulsive g↔e pairs were observed in the eighth heptad and attractive g↔e pairs were observed in the ninth heptad. Attractive g↔e pairs and presence of Asns in a position contribute to homo-dimerization. Repulsive and incomplete g↔e pairs, and charged residues in a positions may favour hetero-dimerization. Based on this principle, all BdbZIPs were classified into three sub-families (sub-family I, II, and III, Additional file 3: Figure S2). According to above analyses, we observed that the dimerization patterns in the leucine zippers of BdbZIPs were more complex and diverse than those in other species. Our results indicated that there were many BdbZIPs with trends to form homo-dimerization.

### BdbZIP proteins structure and expression patterns

**Figure 6:**
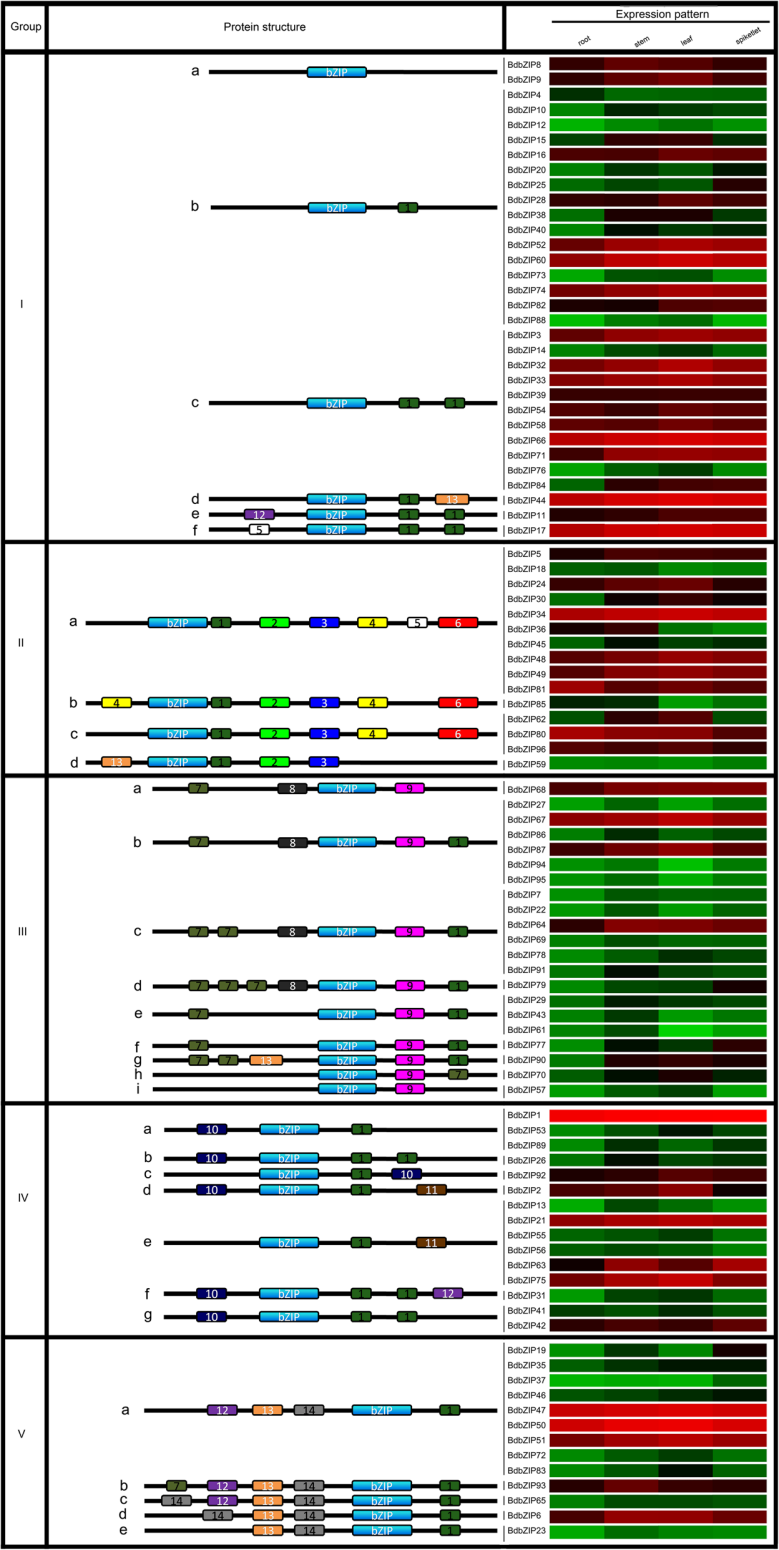
**Tissue-specific expression profiles of** ***BdbZIP*** **genes with bZIP domain and motif information identified by MEME.** Tissue-specific expression analysis was performed. Four tissues/organs including root, stem, leaf, and early spikelet were examined. The position of the bZIP domain and the presence of additional conserved motifs outside the bZIP domain were identified by MEME. BdbZIP domains are shown in blue. Additional motifs are marked in different color boxes with numbers 1 to 14, where the same number refers to the same motif present in the different BdbZIP proteins. The details of predicted conserved motifs are given in Additional file 2: Figure S1.

It was important to answer the question whether BdbZIP protein structure had any correlation with their functions in different tissues/organs. Due that motifs contributed to determine specific functions for gene members, the additional conserved motifs of bZIPs had been detected and classified in plant species [10,11,23]. Based on sequence similarity of conserved motifs, a total of 15 conserved motifs including the bZIP domain were identified (Figure 6). According to the presence of the bZIP region and the additional conserved motifs, we classified 96 BdbZIP proteins into 5 groups (group I-V, Figure 6). All the predicated motifs were exhibited as Additional file 4: Figure S3.

Furthermore, we also investigated tissues/organs specific expression pattern of *BdbZIP*s genes. Four tissues/organs including root, stem, leaf, and early spikelet were selected. The –ΔΔCT changes and fold changes of 96 *BdbZIP* genes were calculated (Figure 6 and Additional file 1: Table S9). Our results showed that the expression levels of *BdbZIP* genes in four tissues/organs displayed with different patterns.

BdbZIP protein structure results showed that except for motif 1 and 7, most of motifs had just one copy. Certain motifs appeared in specific groups and some motifs were shared by several groups. This phenomenon might reflect the case that the functions of some conserved motifs were important and diverse in BdbZIPs. A large number of *BdbZIP* genes belonged to group I and II behaved high expression levels. Except some members in group III, IV, and V had higher expression, the expression for most of the *BdbZIP* genes in these three groups was relatively low. It should be noted that though some of *BdbZIP* genes with same structure were grouped to the same groups, the expression patterns were not completely consistent with the gene structural profiles. These results indicated that the structure of BdbZIP protein was not the single factor in determining their functions in different tissues/organs.

### Stress expression analysis of *BdbZIP* genes

**Figure 7:**
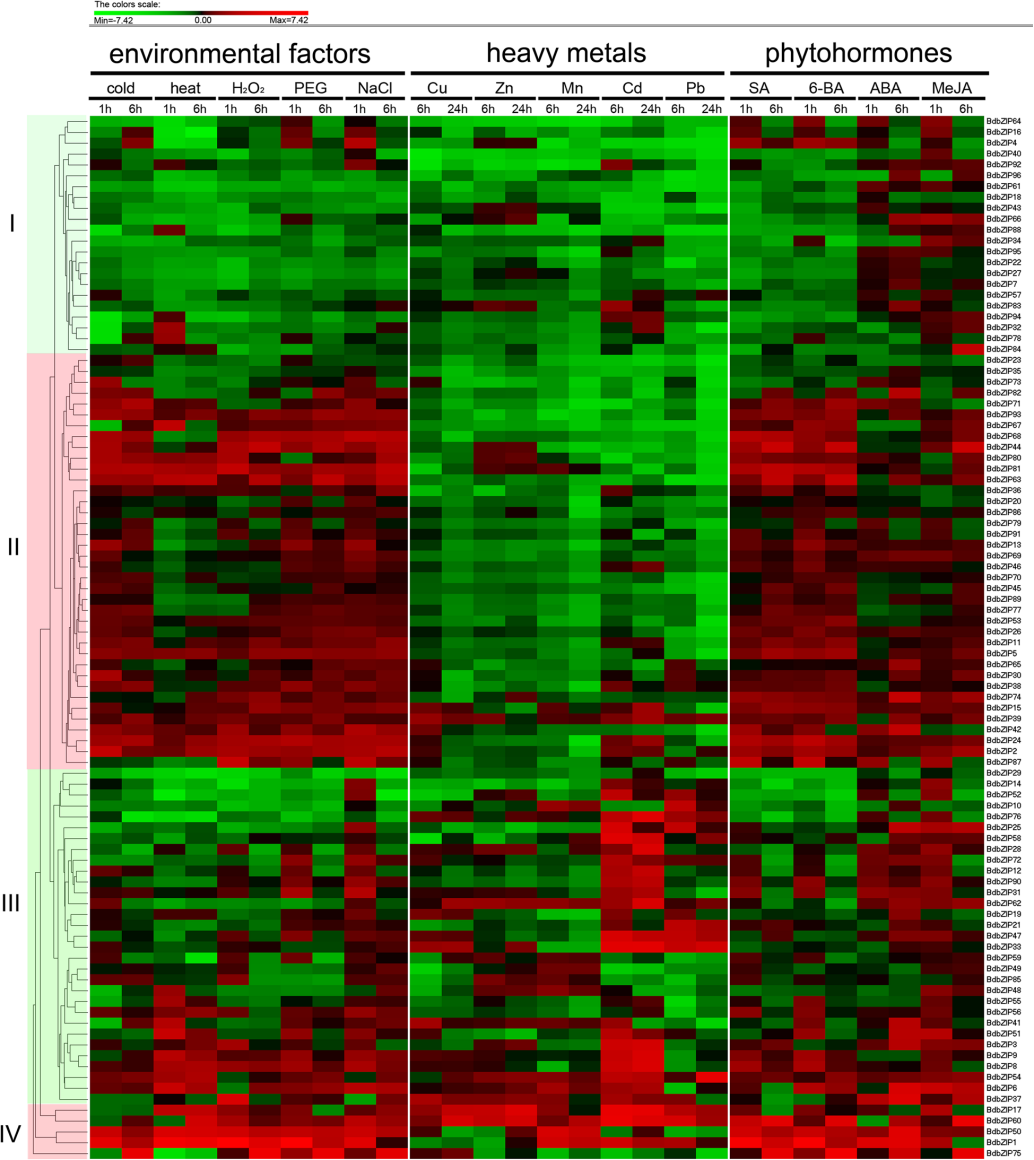
**Expression profiles of** ***BdbZIP*** **genes differentially expressed under multiple stress conditions.** Three major treatment groups including group 1-environmental factors (cold, heat, H_2_O_2_, PEG, NaCl), group 2-heavy metals (Cu, Zn, Mn, Cd, Pb) and group 3-phytohormones (SA, 6-BA, ABA, MeJA), 14 abiotic stresses in total were applied for treatment. After hierarchical cluster analysis, 4 major gene hierarchical clusters (I, II, III, and IV) were obtained.

Plant growth and productivity are greatly affected by various environmental stresses. Stress tolerance is an intricate phenomenon because plants may undergo multiple abiotic stresses, which are the principle cause of reducing crop yields. Given the potential roles of *BdbZIP* genes may play in response to environmental stresses in *B. distachyon*, it is of great importance to demonstrate all *BdbZIP* gene expression profiles under multiple stresses. To investigate the expression patterns, we treated *B. distachyon* with 3 major types of abiotic stresses including environmental factors (cold, heat, H_2_O_2_, PEG, and NaCl), heavey metals (Cu, Zn, Mn, Cd, and Pb) and phytohormones (SA, 6-BA, ABA, and MeJA) respectively. We obtained 4 major clusters (I, II, III, and IV) after hierarchical clustering analysis of all the data upon different types of stress treatments (Figure 7 and Additional file 1: Table S10). According to the expression profile, *BdbZIP*s in cluster I showed down regulation upon almost all three major treatments except ABA and MeJA treatments. As for *BdbZIP* genes in cluster II, we observed that they were up-regulated upon both environmental factors and phytohormone treatments, but they were down-regulated under heavy metal treatments (Figure 7). The *BdbZIP* genes in cluster III and cluster IV had no obvious expression patterns upon different types of stress treatments (Figure 7).

We also investigated whether the *BdbZIP* genes expression cluster had any enrichment with specific phylogenic clade genes, intron pattern, dimerization pattern or motif groups. It was observed that the expression of *BdbZIP*s in cluster II behaved heavy metal specific expression (Figure 7). We found that this cluster was composed of the high proportion of *BdbZIP*s belong to intron pattern b (46%), c (67%), d (67%), and e (50%). So we proposed that the 4 type of intron patterns were crucial in regulation of *BdbZIP*s responding to heavy metal stresses.

In order to further look BdbZIP genes expression pattern to specific group of treatments, we also did hierarchical cluster analysis of the *BdbZIP* genes expression according to environment factors, heavy metal stresses and phytohormone treatments respectively (Additional file 5: Figure S4, Additional file 6: Figure S5, Additional file 7: Figure S6).

### Environmental factors (cold, heat, H2O2, PEG, and NaCl)

The proportion of down-regulated to up-regulated *BdbZIP* genes under environmental factors’ treatment was approximately 50% to 50%. Too high and low temperature were major negative factors on plant development due to the limiting the geographical locations suitable for plant growing and led to catastrophic loss of crop yield [58,59]. To uncover mechanism underlying temperature stresses, *B. distachyon* plants were subjected to heat and cold stress and a set of up- and down- regulated genes were identified, suggesting a prominent role for the bZIP genes responding to these environmental factors. Compared with the global analysis of the transcriptome of *B. distachyon* in cold, heat, drought and salt stress [45], 22 *BdbZIP*s were fell into 13 of 22 modules (Additional file 1: Table S11), which illustrated certain *BdbZIP*s were significant in processes of stress tolerance. Previous studies had shown that when rice was treated with cold, the bZIP genes *OsbZIP14*, *65*, and *83* behaved down-regulated [60]. The expression patterns of these *OsbZIP*s were similar to their homologous *BdbZIP54*, *63*, and *80* respectively. Moreover, many evidences had proposed that excessive NaCl was toxic to plants, because NaCl caused cellular ion imbalances and hyperosmotic stress [61]. So, we also examined the expression profiles of *BdbZIP*s under NaCl and osmotic (PEG) tolerance (Additional file 5: Figure S4 and Additional file 1: Table S10). In salt stress environment, the expressions of *BdbZIP30* and *BdbZIP41* were increased, which were very similar to their homologous *OsbZIP63* and *OsbZIP05* respectively [60].

### Heavy metal stresses

With the development of industry, heavy metals contaminations are well known serious problems. Phytoremediation technologies including hyperaccumulation and uptake widely used to remove heavy metal pollutants are particularly important. Significant progresses have been made in recent years in native plants or genetic modified plants for phytoremediation of pollutants.

Genes associated with heavy metal tolerance or accumulation were identified in green alga, poplar, and maize [62-67]. To gain further insight of the potential roles of *BdbZIP*s may play in phytoremediation, we examined their expression patterns under heavy metals of Zn, Mn, Cu, Cd, and Pb. A series of *BdbZIP*s sensitive to heavy metal were detected (Additional file 6: Figure S5). Eighty percent of the *BdbZIP* genes were suppressed by heavy metal treatments (Additional file 6: Figure S5). The expression patterns of the *BdbZIP* genes under Zn and Mn were similar (Additional file 6: Figure S5).

It was notable that after dealing with heavy metals, there existed a few of *BdbZIP*s with high expression levels. Those *BdbZIP* genes with specific expression under heavy metal treatments might be potential for application of phytoremediation.

### Pytohormones’ treatments

Phytohormones act as endogenous messengers when plants go through stress. During responding to environmental stresses, phytohormones such as auxin, ABA, salicylic acid, gibberellic acid play key roles and coordinate various signal transduction pathways [68]. In plant treated with exogenous hormones, the genome-wide transcript profiles changed rapidly and transiently [69]. Complex networks of transcription factors regulation by phytohormones under abiotic stresses had been reported [68]. In this part, we treated *B. distachyon* seedlings with 4 types of phytohormones (SA, 6-BA, ABA, and MeJA) and investigated the *BdbZIP*s expression patterns. We found about 75% of *BdbZIP* genes were up-regulated upon these phytohormes treatments (Additional file 7: Figure S6). The expression profile of *BdbZIP* genes under SA and 6-BA treatments had similar expression patterns. The expression pattern of *BdbZIP* genes under ABA and MeJA were similar. Our findings showed that the expression patterns of *BdbZIP*s can be regulated by different phytohormones. So we proposed that stress tolerance of *B. distachyon* could be adjusted by applying different phytohormones.

## Conclusions

Ninety six bZIP genes were first identified from the new grass model plant *B.distachon*. The *BdbZIP* genes chromosomal localization with gene duplication and CpG density were analyzed. Phylogenic analysis of these genes with their counterpart species of rice and Arabidopsis were investigated. Further characterization of bZIP domain of these genes in terms of exon splicing of basic and hinge region and identification of dimerization groups were performed. Finally, genes expression profiling of all *BdbZIP* genes upon 14 different stress conditions in *B.distachon* were obtained.

Most of the *BdbZIP* genes were located in the regions with low CpG density in chromosomes. *BdbZIP* gene duplications were widespread throughout the *B. distachyon* genome. Evolutionary analyses suggested that *B. distachyon* and monocot species have the similar evolutionary patterns, which lies two points: (1) *B. distachyon* and other monocot species maintained the similar and high number of bZIP genes, and (2) Seventy-five percent of total *BdbZIP*s have homologous genes in rice. bZIP domain characterization in terms of exon splicing of basic and hinge region and dimerization patterns of leucine zipper exhibited more complex and diverse than those in *O. sativa* and *A. thalinana.* All BdbZIP domains were classified into 3 major dimerization groups, and those BdbZIPs forming homo-dimerization were clustered into the same expression clusters.

Multiple stresses expression profile showed that *BdbZIPs* exhibited significant expression patterns, and those *BdbZIP*s responding to heavy metal treatments showed opposite expression pattern to those of the treatments of environmental factors and phytohormones. Certain *BdbZIP*s with expression level of fold changes ≥2 up- and down-regulated upon multiple-stress treatments were screened.

Abiotic stresses are important research areas of investigating mechanisms associated with crops yields under stress conditions. Identification of novel *BdbZIP* genes associated with stress tolerance and development of some strategies to obtain stress-tolerant plants are our currently major topics or future researches. Determination of the up-regulated, down-regulated, or stress specific *BdbZIP* genes, and utilizing the *BdbZIP* genes to improve productivity of grass plants and cereal crops upon complicated stress environment are crucial and significant.

## Additional files

Additional file 1: Table S1. Treatment conditions. **Table S2.** The syntenic relationships of bZIPs in *B. distrachyon* , rice, and Arabidopsis. **Table S3.** Primers used in this study. **Table S4.** Identification of BdbZIP proteins and their related information. **Table S5.** CpG numbers in *B. distachyon* genome. **Table S6.** *BdbZIP*s, *OsbZIP*s and *AtbZIP*s used. **Table S7.** BdbZIP protein sequences. **Table S8.** Homologous bZIPs in rice and Arabidopsis. **Table S9.** Tissue specific expression datas. **Table S10.** Stress expression datas. **Table S11.** *BdbZIP*s in modules. Additional file 2: Figure S1. Intron patterns within the basic and hinge regions of *B. distachyon* bZIP domains. Additional file 3: Figure S2. The leucine zipper regions. BdbZIPs were classified as three major sub-families according to the predicted dimerization specificity. BdbZIPs in Sub-family I are homo-dimerization specific. BdbZIPs in Sub-family II are homo-hetero-dimerization, and BdbZIPs in sub-family III are hetero-dimerization specific. The leucine zipper regions are divided into heptads (gabcdef) from L0 to L9 to display the potential g↔ e' pairs. Based on the electrostatic charges at the g and e positions, the g↔e pairs were grouped into 4 types, which were displayed with 4 different colors: the frequency of the attractive basic-acidic pairs (+/−) was displayed with green color, attractive acidic-basic pairs (−/+) was displayed with blue color, repulsive acidic pairs (−/−) was displayed with purple color, and repulsive basic pairs (+/+) was displayed with pink color. If single amino acid at the positions e or g is charged, the residue is colored pink for basic amino acid and purple for acidic amino acid. If a or d position is polar, it is colored grey and if either is charged, it is colored orange. Asparagines at a position are colored red. The prolines and glycines are boxed to indicate a potential break in α-helix. The predicted C-terminal boundary is denoted by the symbol ●. Additional file 4: Figure S3. The additional conserved motifs of BdbZIP proteins predicted by MEME. Additional file 5: Figure S4. Exression profiles of BdbZIP genes differentially expressed under environmental factors (cold, heat, H_2_O_2_, PEG, NaCl). Additional file 6: Figure S5. Exression profiles of BdbZIP genes differentially expressed under heavy metal factors (Cu, Zn, Mn, Cd, Pb). Additional file 7: Figure S6. Exression profiles of BdbZIP genes differentially expressed under hytohormones (SA, 6-BA, ABA, MeJA).
