# Supplementary material for: Genome-wide evolutionary characterization and analysis of bZIP transcription factors and their expression profiles in response to multiple abiotic stresses in Brachypodium distachyon
Source: BMC Genomics. 2015 Mar 22;16(1):227. doi: 10.1186/s12864-015-1457-9 (PMC4393604; doi:10.1186/s12864-015-1457-9)
Supplement: Additional file 1: Table S1. — Treatment conditions. Table S2. The syntenic relationships of bZIPs in B. distrachyon , rice, and Arabidopsis. Table S3. Primers used in this study. Table S4. Identification of BdbZIP proteins and their related information. Table S5. CpG numbers in B. distachyon genome. Table S6. BdbZIPs, OsbZIPs and AtbZIPs used. Table S7. BdbZIP protein sequences. Table S8. Homologous bZIPs in rice and Arabidopsis. Table S9. Tissue specific expression datas. Table S10. Stress expression datas. Table S11. BdbZIPs in modules. [file 12864_2015_1457_MOESM1_ESM.pdf]

Additional table S1-S11

Table S1 Treatment conditions

treatments	conditions	time	
cold	4 °C	1h	6h
heat	45 °C	1h	6h
H_2O_2	10 mM	1h	6h
PEG	20%	1h	6h
NaCl	200 mM	1h	6h
$ZnSO_4$	0.5 mM	6h	24h
$CuSO_4$	0.05 mM	6h	24h
$MnCl_2$	1.0 mM	6h	24h
$CdCl_2$	0.07 mM	6h	24h
$PbCl_2$	0.75 mM	6h	24h
SA	1 mM	1h	6h
6-BA	20 µM	1h	6h
ABA	100 µM	1h	6h
MeJA	100 µM	1h	6h

Table S2 The syntenic relationships of bZIPs in *B. distrachyon* , rice, and Arabidopsis

BdbZIPs	Syntenic regions	Ka	Ks	E-value	Score
	BdbZIP20	0.47	1.22	2.00E-91	2228
	OsbZIP59	0.45	1.11	1.00E-59	966
**BdbZIP1**	OsbZIP34	0.3	0.74	1.00E-68	312
	Bradi1g55446	0.75	1.55	2.00E-91	2228
	OsbZIP58	0.41	0.92	1.00E-59	966
**BdbZIP2**	OsbZIP33	0.13	0.71	0	20120
**BdbZIP3**	OsbZIP32	0.15	0.45	0	20120
	BdbZIP80	0.4	0	0	290
	BdbZIP45	0.15	0	1.00E-151	232
	BdbZIP34	0.25	0	2.00E-101	347
	BdbZIP24	0.05	1.02	5.00E-127	2571
	BdbZIP93	0.54	0	8.00E-69	504
	BdbZIP46	0.38	3.28	1.00E-151	232
	BdbZIP35	0.38	3.18	2.00E-101	347
	BdbZIP23	0.38	0.53	5.00E-127	2571
**BdbZIP5**	OsbZIP62	0.29	0.46	9.00E-110	7744
**BdbZIP6**	OsbZIP29	0.36	0.66	5.00E-167	2156
**BdbZIP7**	BdbZIP22	0.22	0.67	5.00E-127	2571
**BdbZIP9**	OsbZIP60	0.18	0.67	9.00E-110	7744
	OsbZIP55	0.15	0.51	5.00E-83	945
**BdbZIP10**	OsbZIP56	0.15	0.51	1.00E-43	543
**BdbZIP11**	OsbZIP53	0.15	0.79	5.00E-83	945
	BdbZIP57	0.39	0.87	5.00E-97	2727
	LOC_Os06g43870	0.29	0.62	0	8543
**BdbZIP12**	LOC_Os02g08540	0.37	0.94	8.00E-89	2096
	BdbZIP55	0.45	1.54	5.00E-97	2727
	OsbZIP52	0.25	0.82	0.00E+00	8543
**BdbZIP13**	OsbZIP15	0.49	1.83	8.00E-89	2096
**BdbZIP15**	OsbZIP50	0.17	0.36	3.00E-79	5311
	BdbZIP60	0.16	0.77	7.00E-26	3033
	BdbZIP28	0.42	0.97	2.00E-31	364
	OsbZIP48	0.11	0.44	3.00E-79	5311
**BdbZIP16**	OsbZIP18	0.21	0.93	2.00E-10	1686
	BdbZIP83	0.37	0.8	1.00E-125	1051
	BdbZIP72	0.22	0.76	1.00E-11	2896
	OsbZIP46	0.12	0.47	8.00E-27	13184
**BdbZIP19**	OsbZIP23	0.21	0.7	1.00E-16	3951
	BdbZIP1	0.47	1.22	2.00E-91	2228
	OsbZIP59	0.09	0.54	0	6833
**BdbZIP20**	OsbZIP34	0.33	1.2	7.00E-113	1214
**BdbZIP22**	BdbZIP7	0.22	0.67	5.00E-127	2571
	BdbZIP93	0.54	2.54	3.00E-102	457
	BdbZIP46	0.41	6.02	0	549
	BdbZIP35	0.44	2.46	0	587
	BdbZIP6	0.38	0.53	5.00E-127	2571
	OsbZIP62	0.33	0.56	5.00E-38	3648
	OsbZIP42	0.4	2.33	0	459
	OsbZIP29	0.26	0.51	0	19568
**BdbZIP23**	OsbZIP09	0.39	1.67	0	242
	BdbZIP80	0.38	0	8.00E-15	327
	BdbZIP45	0.16	2.3	0	549
	BdbZIP34	0.3	0	0	587
**BdbZIP24**	BdbZIP5	0.05	1.02	5.00E-127	2571
	[illegible]	0.41	0.41	0	[illegible]

**BdbZIP26**	OsbZIP26	0.07	0.42	0	988
**BdbZIP27**	BdbZIP64	0.32	0.61	4.00E-96	543
	BdbZIP60	0.31	0.83	9.00E-62	255
	BdbZIP16	0.42	0.97	2.00E-31	364
**BdbZIP28**	OsbZIP01	0.1	0.33	2.00E-35	19294
	BdbZIP61	0.41	1.35	5.00E-17	953
	BdbZIP43	0.48	1.01	3.00E-168	263
	OsbZIP02	0.11	0.37	2.00E-35	19294
**BdbZIP29**	AtbZIP34	0.48	1.97	1.00E-39	213
	BdbZIP41	0.4	0.98	3.00E-35	13810
	OsbZIP45	0.12	0.6	4.00E-92	19523
**BdbZIP31**	OsbZIP05	0.28	0.88	3.00E-145	2015
	Bradi4g43322	0.44	2.45	0	699
	BdbZIP44	0.36	1.05	3.00E-35	13810
	OsbZIP44	0.2	0.67	4.00E-92	19523
**BdbZIP32**	OsbZIP07	0.29	1.61	6.00E-65	6906
	BdbZIP80	0.47	2.3	1.00E-75	224
	BdbZIP45	0.21	1.33	3.00E-35	13810
	BdbZIP24	0.3	0	0	587
**BdbZIP34**	BdbZIP5	0.25	0	2.00E-101	347
	BdbZIP93	0.39	1.71	0	699
	BdbZIP46	0.22	1.07	3.00E-35	13810
	BdbZIP23	0.44	2.46	0	587
	BdbZIP6	0.38	3.18	2.00E-101	347
	OsbZIP42	0.13	0.6	4.00E-92	19523
	OsbZIP29	0.42	0	7.00E-77	260
	OsbZIP09	0.23	0.93	6.00E-65	6906
**BdbZIP35**	AtbZIP12	0.41	0	1.00E-173	212
**BdbZIP36**	BdbZIP48	0.22	1.11	3.00E-35	13810
	BdbZIP51	0.37	1.08	3.00E-35	13810
	OsbZIP40	0.12	0.54	4.00E-92	19523
**BdbZIP37**	OsbZIP12	0.25	0.95	6.00E-65	6906
**BdbZIP38**	OsbZIP39	0.12	0.54	4.00E-92	19523
**BdbZIP39**	OsbZIP38	0.08	0.3	0	2478
	BdbZIP31	0.4	0.98	3.00E-35	13810
**BdbZIP41**	OsbZIP05	0.18	0.49	0	50118
	BdbZIP29	0.48	1.01	3.00E-168	263
**BdbZIP43**	OsbZIP06	0.12	0.43	0	50118
	Bradi4g43322	0.43	0	3.00E-40	740
	BdbZIP32	0.36	1.05	3.00E-35	13810
	OsbZIP44	0.34	1.48	1.00E-128	10088
**BdbZIP44**	OsbZIP07	0.33	1.37	0	50118
	BdbZIP34	0.21	1.33	3.00E-35	13810
	BdbZIP24	0.16	2.3	0	549
**BdbZIP45**	BdbZIP5	0.15	0	1.00E-151	232
	BdbZIP93	0.42	1.7	3.00E-40	740
	BdbZIP35	0.22	1.07	3.00E-35	13810
	BdbZIP23	0.41	6.02	0	549
	BdbZIP6	0.38	3	1.00E-151	232
	OsbZIP42	0.23	1.03	1.00E-128	10088
**BdbZIP46**	OsbZIP09	0.18	0.65	0	50118
	OsbZIP10	0.22	1.18	0	50118
**BdbZIP47**	AtbZIP39	0.67	0	3.00E-89	218
**BdbZIP48**	BdbZIP36	0.22	1.11	3.00E-35	13810
	BdbZIP37	0.37	1.08	3.00E-35	13810
	OsbZIP40	0.38	1.01	1.00E-128	10088

**BdbZIP53**	OsbZIP13	0.11	0.45	0	18847
**BdbZIP54**	OsbZIP14	0.14	0.27	0	18847
	BdbZIP13	0.45	1.54	5.00E-97	2727
	OsbZIP52	0.46	1.19	2.00E-10	3347
**BdbZIP55**	OsbZIP15	0.15	0.57	0	18847
	BdbZIP12	0.39	0.87	5.00E-97	2727
	LOC_Os06g43870	0.27	0.61	2.00E-10	3347
**BdbZIP57**	LOC_Os02g08540	0.26	0.45	0	18847
	BdbZIP28	0.31	0.83	9.00E-62	255
	BdbZIP16	0.16	0.77	9.00E-62	255
	OsbZIP48	0.22	0.66	2.00E-10	3347
**BdbZIP60**	OsbZIP18	0.07	0.47	0	18847
	BdbZIP29	0.41	1.35	5.00E-17	953
	OsbZIP19	0.07	0.53	0	18847
	OsbZIP02	0.36	1.31	7.00E-11	388
**BdbZIP61**	AtbZIP34	0.39	0	6.00E-54	210
	BdbZIP96	0.46	0.71	6.00E-81	947
	OsbZIP64	0.35	0.57	6.00E-73	3680
**BdbZIP62**	OsbZIP37	0.45	1.09	1.00E-81	429
**BdbZIP63**	BdbZIP82	0.39	0.97	0	481
**BdbZIP64**	BdbZIP27	0.32	0.61		
	BdbZIP83	0.23	0.94	2.00E-105	416
	OsbZIP72	0.2	0.87	9.00E-44	490
	OsbZIP66	0.1	0.61	1.00E-147	548
**BdbZIP65**	OsbZIP23	0.35	1.04	3.00E-146	207
	BdbZIP84	0.37	0.97	9.00E-17	4924
	BdbZIP71	0.59	0	2.00E-38	222
	Bradi1g43661	0.79	1.54	0	314
	OsbZIP73	0.37	0.7	2.00E-16	3616
**BdbZIP66**	OsbZIP67	0.19	0.56	6.00E-10	12150
**BdbZIP67**	BdbZIP86	0.26	0.76	9.00E-17	4924
**BdbZIP69**	OsbZIP76	0.8	1.4	2.00E-16	3616
**BdbZIP70**	OsbZIP21	0.1	0.61	3.00E-78	23302
	BdbZIP84	0.64	2.02	8.00E-38	262
	BdbZIP66	0.59	0	2.00E-38	222
	Bradi1g43661	0.49	2.34	3.00E-110	742
**BdbZIP71**	OsbZIP22	0.1	0.29	8.00E-28	1221
	BdbZIP83	0.41	1.07	0	701
	BdbZIP19	0.22	0.76	1.00E-11	2896
	OsbZIP72	0.39	1.14	2.00E-20	513
	OsbZIP66	0.38	1.55	1.00E-19	228
	OsbZIP46	0.23	0.91	4.00E-174	2195
	OsbZIP23	0.14	0.57	3.00E-27	6264
**BdbZIP72**	AT1G45249	0.43	0	5.00E-24	261
**BdbZIP73**	OsbZIP24	0.33	0.76	6.00E-147	1503
**BdbZIP75**	OsbZIP88	0.21	0.61	3.00E-128	8119
**BdbZIP76**	OsbZIP87	0.08	0.26	3.00E-128	8119
**BdbZIP77**	OsbZIP82	0.1	0.69	0	4313
	BdbZIP91	0.14	0.72	2.00E-136	1522
**BdbZIP78**	OsbZIP81	0.19	0.53	0	4313
	BdbZIP34	0.47	2.3	1.00E-75	224
	BdbZIP5	0.4	0	0	290
**BdbZIP80**	BdbZIP24	0.38	0	8.00E-15	327
	BdbZIP63	0.39	0.97	0	481
**BdbZIP82**	OsbZIP71	0.16	0.74	6.00E-95	24205
	BdbZIP65	0.23	0.94	2.00E-105	416

	BdbZIP19	0.37	0.8	1.00E-125	1051
	OsbZIP72	0.15	0.48	6.00E-95	24205
	OsbZIP66	0.23	0.99	1.00E-74	5730
	OsbZIP46	0.32	1.06	9.00E-21	349
**BdbZIP83**	OsbZIP23	0.36	1.22	2.00E-107	463
	BdbZIP71	0.64	2.02	8.00E-38	262
	BdbZIP66	0.37	0.97	9.00E-17	4924
	Bradi1g43661	0.8	1.05	0	255
	OsbZIP73	0.24	0.45	6.00E-95	24205
**BdbZIP84**	OsbZIP67	0.3	0.55	1.00E-74	5730
**BdbZIP86**	BdbZIP67	0.26	0.76	9.00E-17	4924
**BdbZIP87**	OsbZIP76	0.56	0.92	3.00E-17	358
	OsbZIP77	0.33	0.41	6.00E-95	24205
**BdbZIP88**	AtbZIP14	0.83	0	3.00E-24	224
**BdbZIP89**	OsbZIP86	0.07	0.38	0	2230
	BdbZIP78	0.14	0.72	2.00E-136	1522
**BdbZIP91**	OsbZIP81	0.15	0.78	0	5137
**BdbZIP92**	Bradi4g25342	0.39	0.63	2.00E-136	1522
	BdbZIP35	0.39	1.71	0	699
	BdbZIP46	0.42	1.7	3.00E-40	740
**BdbZIP93**	BdbZIP23	0.54	2.54	3.00E-102	457
	BdbZIP62	0.46	0.71	6.00E-81	974
	OsbZIP64	0.47	0.7	2.00E-63	239
**BdbZIP96**	OsbZIP37	0.2	0.74	0	37364

Table S3 Primers used in this study

Primer name	Sequence (5'-3')	Primer name	Sequence (5'-3')
**BdbZIP1-F**	ATATCGGGATGGGTCATTCA	**BdbZIP1-R**	TAATCCTTTGGCCAGGTCAC
**BdbZIP2-F**	ACGGAGGAGGAAGCAATCAA	**BdbZIP2-R**	GTCCTCTGCCATCTTCACCT
**BdbZIP3-F**	AGGAGGCGATTGACTTTGAC	**BdbZIP3-R**	AGCTCCTCCTCGTCCTCTTC
**BdbZIP4-F**	ATCCAATGCGTTCCAATCAT	**BdbZIP4-R**	CTCCGCTCCTTGTTGAAGAC
**BdbZIP5-F**	GGGTTAACAAACCTCCAGCA	**BdbZIP5-R**	TTCTCAAGCGTTCCGAGTTT
**BdbZIP6-F**	AGATGACGCTCGAGGACTTC	**BdbZIP6-R**	CGGTTCTTGATCATCCGCTT
**BdbZIP7-F**	CTCTTTCGGCACAACTGACA	**BdbZIP7-R**	ATTTTGTCGTCTCCCCTGTG
**BdbZIP8-F**	TCCTGCAGTCAATCGGATGT	**BdbZIP8-R**	CTTGGGAGATACAACAGCGC
**BdbZIP9-F**	TCCTGCAGTCAATCGGATGT	**BdbZIP9-R**	CTTGGGAGATACAACAGCGC
**BdbZIP10-F**	CAGCTCGCTCTTCTCCATCT	**BdbZIP10-R**	TCTTCCTTGCTCTGGATCGT
**BdbZIP11-F**	CCCGTGAAGAACGTTGATTT	**BdbZIP11-R**	AACGCCTGACCATTCATAGC
**BdbZIP12-F**	GCACGCTAGAGACAAGGTTG	**BdbZIP12-R**	CGCTGGAATGTTGAGATCGG
**BdbZIP13-F**	CAGGCCCTTGAAAGTGAGTC	**BdbZIP13-R**	AATTGTGCGTGCTTTCTCCT
**BdbZIP14-F**	CAGCTGGATGAGCTGTCG	**BdbZIP14-R**	GCAGCAGCTTCTTCTTCGTC
**BdbZIP15-F**	AGGTGGATGTGGAGAAGGTG	**BdbZIP15-R**	CCAACAGGCCTATCCTTCAA
**BdbZIP16-F**	AGAGGAAGAAGGCGTACGTG	**BdbZIP16-R**	CTGGTCCTCTCCTGTTTCCA
**BdbZIP17-F**	ATCTTTGGTGATGGGACTGC	**BdbZIP17-R**	GCCAGAGTATGAACGGAGGA
**BdbZIP18-F**	GAACTCCTGAAGGTGCTTGC	**BdbZIP18-R**	GGCCATCTGGCTCATGTAGT
**BdbZIP19-F**	GTCGAGAAGGTGGTCGAAAG	**BdbZIP19-R**	ACGTCGCAAGCAAAATCTCT
**BdbZIP20-F**	TGCCTCAAAGCTTCCTGTTT	**BdbZIP20-R**	TCTCCTTGCAGACTCCCTGT
**BdbZIP21-F**	GGTGCCCATGAAACTTTTGT	**BdbZIP21-R**	GCACTGATGAACTTGGAGCA
**BdbZIP22-F**	GACAGAACTTGAGCGGAAGG	**BdbZIP22-R**	CATTCAGAGCATCACGCAGT
**BdbZIP23-F**	GGAAGAAGCGGATGATCAAG	**BdbZIP23-R**	GTTCTTCGGCTCCTCTTGTG
**BdbZIP24-F**	TATCCAACCTCCAGCAGTCC	**BdbZIP24-R**	TTGCTGCCGTAGATTGTCAG
**BdbZIP25-F**	TGCTCCAGCAGGAGAACC	**BdbZIP25-R**	TTGATCAATTTGTCGCTCCA
**BdbZIP26-F**	TTCAAGCATCAAATGGGACA	**BdbZIP26-R**	TCCACTTCCACTCTCGCTTT
**BdbZIP27-F**	GACAGAACTTGAGCGGAAGG	**BdbZIP27-R**	CATTCAGAGCATCACGCAGT
**BdbZIP28-F**	GAGAGCGACGAGGAGATACG	**BdbZIP28-R**	CGTCATGTAGGCCTTCTTCC
**BdbZIP29-F**	GCATCCGACGAGTTCCTAGA	**BdbZIP29-R**	GTGAGGTCGTCGGAGAACAT
**BdbZIP30-F**	CTCGTCAACAGGGCATTTTT	**BdbZIP30-R**	TCGCTTGCATGAGCATTAAC
**BdbZIP31-F**	GGCTGAGACAGAGGAGTTGG	**BdbZIP31-R**	GGTTGTGTCGTCGATCATTG
**BdbZIP32-F**	CAAGATTGACGCTGAGGTCG	**BdbZIP32-R**	CCATGGAATGCACCTGTTCC
**BdbZIP33-F**	CAAGATTGACGCTGAGGTCG	**BdbZIP33-R**	CCATGGAATGCACCTGTTCC
**BdbZIP34-F**	GCTGGAGGAGCACAATAAGC	**BdbZIP34-R**	GTCCACGCTCCTGCTAGAAC
**BdbZIP35-F**	TGGAGCACAGTGGTTACAGC	**BdbZIP35-R**	GCATTGGTGATGTCATCTGG
**BdbZIP36-F**	TAAGCAGCTGCACCAATCAC	**BdbZIP36-R**	CTCCGATGGAGAGAAAGCAC
**BdbZIP37-F**	TGATGACCCTGGAGGATTTC	**BdbZIP37-R**	GTTTCCTCTCCCTGGACCTC
**BdbZIP38-F**	CGAGAAAGCAGTGGCACATA	**BdbZIP38-R**	CTGAAATACCTCGCTGCACA
**BdbZIP39-F**	GATGCTGTCGAACAGGGAGT	**BdbZIP39-R**	CGTCCACCTTGCTCAGCTC
**BdbZIP40-F**	GAAGCGCAAGGAGTCGAAC	**BdbZIP40-R**	GTCCATGCACTGGATGAGGT
**BdbZIP41-F**	TCACATCCTTTCATGCCCCT	**BdbZIP41-R**	TTGATCCTCCTACGCTGCAA
**BdbZIP42-F**	TCACATCCTTTCATGCCCCT	**BdbZIP42-R**	TTGATCCTCCTACGCTGCAA
**BdbZIP43-F**	CGACCCCAAGAGAGTCAAGA	**BdbZIP43-R**	TCTTGATGAGCGTCCTTGAA
**BdbZIP44-F**	CGGCTTCGATGAATTCCTAA	**BdbZIP44-R**	GGCCTTCTTCTTCTCCCTGT
**BdbZIP45-F**	AACCGTGAAGCTGCTAGGAA	**BdbZIP45-R**	AGACTGATCCCCTGACGATG
**BdbZIP46-F**	ATCTGGGGCACAGTGGTTAG	**BdbZIP46-R**	GGCCATCAGAGTAGGCAGAG

**BdbZIP47-F**	TCGGAGATGAGCAAGAACCT	**BdbZIP47-R**	CGAACTCGTCCATGTTGATG
**BdbZIP48-F**	AGAGGAAACATGGACTCGCA	**BdbZIP48-R**	CCCTCCAAGAAATGTCCCCT
**BdbZIP49-F**	AGAGGAAACATGGACTCGCA	**BdbZIP49-R**	CCCTCCAAGAAATGTCCCCT
**BdbZIP50-F**	GTTTCTCAACGGGCCAGATG	**BdbZIP50-R**	TCTCGTTTTGCTCCTCCTGT
**BdbZIP51-F**	GTTTCTCAACGGGCCAGATG	**BdbZIP51-R**	TCTCGTTTTGCTCCTCCTGT
**BdbZIP52-F**	GAAGGACATGTCGTCCCTGT	**BdbZIP52-R**	CAGCCGAGGTAGGTGAACTC
**BdbZIP53-F**	GAATGGACTACTGGGGTGCT	**BdbZIP53-R**	TTGACCGAGCTCTTCACACT
**BdbZIP54-F**	TCCCTCAACGACATCCTCTC	**BdbZIP54-R**	GGCTGGAACATGAAGAGGTC
**BdbZIP55-F**	AGTGGTTCAGACTCCGACAG	**BdbZIP55-R**	AACCTGTGTCTCGAGCTCAA
**BdbZIP56-F**	TTGAGCTCGAGACACAGGTT	**BdbZIP56-R**	TGCGACACCATTTTCTCTGC
**BdbZIP57-F**	GTCAGCGCCAAAGGAAGAAA	**BdbZIP57-R**	TTTGTGAGTTGGATCGCTGC
**BdbZIP58-F**	GCGACGAGAGGAAGAAGAGG	**BdbZIP58-R**	TCCCATCTCATCGAGCTTCT
**BdbZIP59-F**	GTCCCAGATGGAACAGGAGA	**BdbZIP59-R**	TGCTGCAGGTTGTTATCGTC
**BdbZIP60-F**	ACGACGAGATAGGGATGGTG	**BdbZIP60-R**	CACCTCCAGATCACCCAAAT
**BdbZIP61-F**	CCCCAAGAGGGTTAAAAGGA	**BdbZIP61-R**	GACCTCTGGTGATCCAGGAA
**BdbZIP62-F**	ACATCCAGAACCTGGAGACG	**BdbZIP62-R**	GATTGAAGGATGGTGGCAGT
**BdbZIP63-F**	GCGGAAGAGGAGAAAGGAGT	**BdbZIP63-R**	GCACTGAGTTCTGGGTCTCC
**BdbZIP64-F**	AGCTGTTCAGCCTCGAGTTC	**BdbZIP64-R**	AGGATCTGCACCTTGTGCTC
**BdbZIP65-F**	ATGTGGAGAAGGTGGTGGAG	**BdbZIP65-R**	TGGGTTGTTGATCACTTCCA
**BdbZIP66-F**	AAACCCTTCCTCCTCCTTCA	**BdbZIP66-R**	CCAGCACTACCCTCCTGGTA
**BdbZIP67-F**	ATAGAGACACTGCTGGGCTC	**BdbZIP67-R**	ATTGCTGTCTGGCTCTGGAT
**BdbZIP68-F**	CCAAGAAGGCCATGTCCAAC	**BdbZIP68-R**	GAGCCCAGCAGTGTCTCTAT
**BdbZIP69-F**	AAGGAGCGCAAGATGAGGTA	**BdbZIP69-R**	TCACCAGTTGCGAGTTTCAG
**BdbZIP70-F**	CAAGCTCCCAATTTTTCCAA	**BdbZIP70-R**	TACTGCAGGGGGTTGTTAGG
**BdbZIP71-F**	CAACACAGCCTTGCAGAAGA	**BdbZIP71-R**	CAAGCTGCTGTTTCAGTTCG
**BdbZIP72-F**	AGCCACAGATGCTGTTTCCT	**BdbZIP72-R**	CAAACCCATTGGACAAAACC
**BdbZIP73-F**	AGCTCCCTCTTCTCCGTCTC	**BdbZIP73-R**	GACCTCCTTCTCGAGCTCCT
**BdbZIP74-F**	ACATCGACGCCTTCCTCG	**BdbZIP74-R**	GACTCCCGGTTCGAGATCC
**BdbZIP75-F**	GACATGGCCGGAAGTAGTGT	**BdbZIP75-R**	CCACTGGGAACAACAGGAGT
**BdbZIP76-F**	ATGTCGTCGTCGTCTCTGTC	**BdbZIP76-R**	GTCCAGGTGCTGCTGCTT
**BdbZIP77-F**	AAACGCGTTCAACAGGAGAT	**BdbZIP77-R**	GCGTTTGGGTCTTTGTGTTT
**BdbZIP78-F**	AAGATCAAGTACACCGGCGA	**BdbZIP78-R**	CTTCCATGGACTGCAATCGG
**BdbZIP79-F**	AAGATCAAGTACACCGGCGA	**BdbZIP79-R**	CTTCCATGGACTGCAATCGG
**BdbZIP80-F**	AAGCACTCACCTGATCACCA	**BdbZIP80-R**	CCTTGCCAGTCCTGTCACTA
**BdbZIP81-F**	GAGAACACAGGACCCCAAGA	**BdbZIP81-R**	AACATCCCAGCAACTGAAGG
**BdbZIP82-F**	CACCTTGACGACCTCTCCTC	**BdbZIP82-R**	GCTGGATTTGTGGTGTTCCT
**BdbZIP83-F**	GAGATCTGGCCTTGTGCAAT	**BdbZIP83-R**	AACGAGTACGGCATTGGTTC
**BdbZIP84-F**	AGCTCAACCAAGCAATGAGG	**BdbZIP84-R**	AGCGGTGGCAGTAATTTTGT
**BdbZIP85-F**	CGGTACAACAACAGCACCAG	**BdbZIP85-R**	GCTGCTGGATGTAAGCCTTC
**BdbZIP86-F**	CTAACAGACAATCCGCAGCA	**BdbZIP86-R**	CTCCGTTTTCAGGGTGTCAT
**BdbZIP87-F**	TGCTGACCGATCCTAAAAGG	**BdbZIP87-R**	CTCAACTGTGCTTGCTGCTC
**BdbZIP88-F**	AAGCGCATGATCAAGAACCG	**BdbZIP88-R**	AGCACCCTCTTGAGCGTC
**BdbZIP89-F**	GCAATGCTGATGCTACTGGA	**BdbZIP89-R**	TTGCCATGTTCCACACAGTT
**BdbZIP90-F**	TCCCAGGCAGATGTTCCTAC	**BdbZIP90-R**	TTGGTGTCTGCCTCTGACTG
**BdbZIP91-F**	ACTGGTGAGCTGGAGAGGAA	**BdbZIP91-R**	GGGATTTCCGTTCATGTTTG
**BdbZIP92-F**	GAGAGGAGGAAGACGACACC	**BdbZIP92-R**	TCATCCTGGTGAGCTCCTTC
**BdbZIP93-F**	CAGTCAATTGCACAGCCACT	**BdbZIP93-R**	CGAGCTCATTTGTGTAGGCC
**BdbZIP94-F**	TTGCAGCTCAGTTGTCCATG	**BdbZIP94-R**	TCTTGAGCTGCTGAACCTCA

**BdbZIP95-F**	TTGCAGCTCAGTTGTCCATG	**BdbZIP95-R**	TCTTGAGCTGCTGAACCTCA
**BdbZIP96-F**	TTGAAGAGGTTCCAGGGATG	**BdbZIP96-R**	CTAGCTGCGCCAGCTTTATC
**BdGAPD-F**	GCTGGTGCCGATTATGTCGT	**BdGAPD-R**	AGTGGTGCAGCTAGCATTTGAGACAAT

Table S4 Identification of BdbZIP proteins and their related information

Name	Gene ID	Gene position	protein length	position of bzip domain	No. of introns	patterns of introns
**BdbZIP1**	bradi1g04510	3027649 - 3030843	373	258-320	7	b
**BdbZIP2**	bradi1g05480	3718882 - 3723615	168	229-280	5	a
**BdbZIP3**	bradi1g07310	5138398 - 5138973	191	54-101	0	h
**BdbZIP4**	bradi1g12620	9497384 - 9497890	168	64-108	0	h
**BdbZIP5**	bradi1g17210	13799342 - 13801948	340	50-89	7	e
**BdbZIP6**	bradi1g17335	13859363 - 13861306	270	161-212	2	b
**BdbZIP7**	bradi1g17700	14275458 - 14280590	351	146-194	3	a
**BdbZIP8**	bradi1g19700	15780612-15771044	1035	118-183	3	h
**BdbZIP9**	bradi1g19702	15777919 - 15780633	578	127-187	1	h
**BdbZIP10**	bradi1g29920	25408086 - 25411112	153	81-137	2	b
**BdbZIP11**	bradi1g30140	25546820 - 25549746	269	93-138	1	h
**BdbZIP12**	bradi1g30750	26113326 - 26118403	273	158-205	4	g
**BdbZIP13**	bradi1g31700	27161490 - 27164071	294	132-181	5	a
**BdbZIP14**	bradi1g35550	31095647 - 31096311	177	81-132	0	h
**BdbZIP15**	bradi1g35790	31373776 - 31376534	292	132-178	2	a
**BdbZIP16**	bradi1g36750	32421876 - 32423425	141	56-117	2	a
**BdbZIP17**	bradi1g43660	41467845 - 41472473	439	325-381	4	h
**BdbZIP18**	bradi1g43900	41768905 - 41771421	365	77-118	7	e
**BdbZIP19**	bradi1g46060	44341065 - 44348362	328	248-299	3	b
**BdbZIP20**	bradi1g54180	52574419 - 52579115	377	253-315	8	b
**BdbZIP21**	bradi1g55450	53892906 - 53896870	421	221-273	5	a
**BdbZIP22**	bradi1g63170	62379687 - 62383641	358	162-209	3	a
**BdbZIP23**	bradi1g63840	63090295 - 63093451	223	153-195	2	b
**BdbZIP24**	bradi1g64060	63261797 - 63266448	334	49-91	8	e
**BdbZIP25**	bradi1g64550	63729973 - 63730588	182	18-78	0	h
**BdbZIP26**	bradi1g68560	67117639 - 67124207	392	300-362	11	b
**BdbZIP27**	bradi1g76690	73174527 - 73176830	426	280-333	3	a
**BdbZIP28**	bradi2g04590	3198561 - 3199996	186	95-148	2	h
**BdbZIP29**	bradi2g06790	5199168 - 5200866	266	131-178	4	a
**BdbZIP30**	bradi2g10830	9048037 - 9053559	329	45-85	8	e
**BdbZIP31**	bradi2g15940	14078936 - 14084078	378	240-302	11	b
**BdbZIP32**	bradi2g21197	18595360-18596061	233	77-123	0	h
**BdbZIP33**	bradi2g21200	18595360-18598135	298	70-141	1	h
**BdbZIP34**	bradi2g21380	18754986 - 18756835	310	25-62	6	e
**BdbZIP35**	bradi2g21820	19173454 - 19176099	331	261-304	2	b
**BdbZIP36**	bradi2g23890	21649234 - 21654211	471	163-191	11	e
**BdbZIP37**	bradi2g24120	21981310 - 21984976	241	159-211	3	b
**BdbZIP38**	bradi2g25400	23426305 - 23429795	642	170-230	1	h
**BdbZIP39**	bradi2g38380	38691853 - 38693237	150	25-83	0	h
**BdbZIP40**	bradi2g40580	40859127 - 40859507	126	27-85	0	h
**BdbZIP41**	bradi2g45567	45971262-45974197	372	238-299	10	b
**BdbZIP42**	bradi2g45570	45974197-45971390	379	237-302	10	b
**BdbZIP43**	bradi2g50220	50160740 - 50163812	276	151-199	3	a
**BdbZIP44**	bradi2g52590	51927312 - 51928125	218	85-130	0	h
**BdbZIP45**	bradi2g52860	52116507 - 52122329	476	190-231	10	e

**BdbZIP46**	bradi2g53060	52281872 - 52285991	333	263-306	2	b
**BdbZIP47**	bradi2g55550	54161519 - 54163078	415	332-376	3	b
**BdbZIP48**	bradi2g55567	54170494 - 54176864	544	233-263	11	e
**BdbZIP49**	bradi2g55570	54176202-54170824	506	192-268	10	e
**BdbZIP50**	bradi2g56080	54506707-54497690	273	178-242	3	b
**BdbZIP51**	bradi2g56096	54504165 - 54506707	262	180-233	3	b
**BdbZIP52**	bradi3g00300	93061 - 94218	190	118-167	1	h
**BdbZIP53**	bradi3g02730	1672435 - 1677861	319	248-310	13	b
**BdbZIP54**	bradi3g02980	1901143 - 1902688	193	37-77	0	h
**BdbZIP55**	bradi3g05577	3938297 - 3941300	312	143-197	5	a
**BdbZIP56**	bradi3g05580	3939872-3940793	186	15-79	3	a
**BdbZIP57**	bradi3g06160	4438924 - 4444696	240	143-185	4	g
**BdbZIP58**	bradi3g06670	4862848 - 4863464	166	63-111	0	h
**BdbZIP59**	bradi3g07030	5212213 - 5216012	295	103-133	5	b
**BdbZIP60**	bradi3g07540	5705282 - 5707940	156	80-141	3	a
**BdbZIP61**	bradi3g09340	7459853 - 7462181	243	116-160	3	a
**BdbZIP62**	bradi3g15590	13851942 - 13853895	424	102-134	3	f
**BdbZIP63**	bradi3g22040	21143026 - 21145398	183	36-95	1	h
**BdbZIP64**	bradi3g31890	34118752 - 34120817	379	228-285	3	a
**BdbZIP65**	bradi3g38200	40739538 - 40744954	353	275-327	3	b
**BdbZIP66**	bradi3g38840	41303635 - 41304341	220	104-157	0	h
**BdbZIP67**	bradi3g41817	43632626 - 43635160	345	170-228	3	a
**BdbZIP68**	bradi3g41820	43635160-43632222	267	166-230	3	c
**BdbZIP69**	bradi3g41980	43755511 - 43759060	467	316-368	3	a
**BdbZIP70**	bradi3g45170	47114542 - 47119299	323	179-238	3	d
**BdbZIP71**	bradi3g56290	56234835 - 56235578	170	79-137	0	h
**BdbZIP72**	bradi3g57960	57578254 - 57582475	355	275-318	3	b
**BdbZIP73**	bradi3g60870	59661881 - 59662620	146	65-124	1	h
**BdbZIP74**	bradi4g00810	415064 - 415796	191	71-120	0	h
**BdbZIP75**	bradi4g02570	1833523 - 1838483	307	111-164	5	a
**BdbZIP76**	bradi4g04720	3875691 - 3876975	159	32-90	0	h
**BdbZIP77**	bradi4g22130	26176810 - 26180896	358	227-260	4	d
**BdbZIP78**	bradi4g24937	30132694 - 30137170	340	173-231	3	a
**BdbZIP79**	bradi4g24940	30138551-30132694	496	325-389	5	c
**BdbZIP80**	bradi4g26670	31762448 - 31767303	464	165-193	10	e
**BdbZIP81**	bradi4g27100	32144473 - 32152246	472	180-208	10	e
**BdbZIP82**	bradi4g27720	32892174 - 32894374	151	30-74	1	h
**BdbZIP83**	bradi4g32090	37815661 - 37819266	359	280-331	3	b
**BdbZIP84**	bradi4g32920	38717437 - 38718119	188	84-136	0	h
**BdbZIP85**	bradi4g33740	39416189 - 39422784	529	210-242	10	e
**BdbZIP86**	bradi4g35240	40688906 - 40694073	378	180-238	3	a
**BdbZIP87**	bradi4g35370	40806491 - 40809376	467	314-366	3	a
**BdbZIP88**	bradi4g36587	41762220 - 41762920	186	117-169	1	h
**BdbZIP89**	bradi4g39630	44284132 - 44289289	389	291-353	12	b
**BdbZIP90**	bradi4g40540	44919143-44921906	359	215-274	3	d
**BdbZIP91**	bradi4g41890	45837336 - 45840741	329	161-219	3	a
**BdbZIP92**	bradi4g42120	46188160 - 46189982	353	122-171	3	b
**BdbZIP93**	bradi4g43850	47446805 - 47450282	314	242-287	3	b

**BdbZIP94**	bradi5g14497	17873779 - 17876984	372	170-227	3	a
**BdbZIP95**	bradi5g14500	17876984-17874142	434	232-292	5	c
**BdbZIP96**	bradi5g23340	25312831 - 25315109	388	92-134	5	e

Table S5 CpG numbers in *B. distachyon* genome

locus	positions		GC numbers
bdis1	1	100000	3868
bdis1	100001	200000	3654
bdis1	200001	300000	4239
bdis1	300001	400000	4309
bdis1	400001	500000	3392
bdis1	500001	600000	4548
bdis1	600001	700000	3807
bdis1	700001	800000	4354
bdis1	800001	900000	4110
bdis1	900001	1000000	3830
bdis1	1000001	1100000	4541
bdis1	1100001	1200000	4714
bdis1	1200001	1300000	4285
bdis1	1300001	1400000	3805
bdis1	1400001	1500000	4621
bdis1	1500001	1600000	4132
bdis1	1600001	1700000	4171
bdis1	1700001	1800000	3451
bdis1	1800001	1900000	4001
bdis1	1900001	2000000	3666
bdis1	2000001	2100000	4849
bdis1	2100001	2200000	5037
bdis1	2200001	2300000	5266
bdis1	2300001	2400000	4529
bdis1	2400001	2500000	4862
bdis1	2500001	2600000	4601
bdis1	2600001	2700000	4345
bdis1	2700001	2800000	4214
bdis1	2800001	2900000	4277
bdis1	2900001	3000000	4801
bdis1	3000001	3100000	3928
bdis1	3100001	3200000	4346
bdis1	3200001	3300000	4359
bdis1	3300001	3400000	3653
bdis1	3400001	3500000	3613
bdis1	3500001	3600000	4587
bdis1	3600001	3700000	4082
bdis1	3700001	3800000	4360
bdis1	3800001	3900000	3460
bdis1	3900001	4000000	4512
bdis1	4000001	4100000	4336
bdis1	4100001	4200000	5638
bdis1	4200001	4300000	3974
bdis1	4300001	4400000	3870
bdis1	4400001	4500000	4408
bdis1	4500001	4600000	4612

locus	positions		GC numbers
bdis3	1400001	1500000	4159
bdis3	1500001	1600000	4305
bdis3	1600001	1700000	3816
bdis3	1700001	1800000	3336
bdis3	1800001	1900000	3813
bdis3	1900001	2000000	4242
bdis3	2000001	2100000	3576
bdis3	2100001	2200000	3531
bdis3	2200001	2300000	3677
bdis3	2300001	2400000	3269
bdis3	2400001	2500000	3461
bdis3	2500001	2600000	4283
bdis3	2600001	2700000	4246
bdis3	2700001	2800000	3839
bdis3	2800001	2900000	3530
bdis3	2900001	3000000	4374
bdis3	3000001	3100000	4352
bdis3	3100001	3200000	4246
bdis3	3200001	3300000	3720
bdis3	3300001	3400000	4847
bdis3	3400001	3500000	4214
bdis3	3500001	3600000	3431
bdis3	3600001	3700000	4101
bdis3	3700001	3800000	3703
bdis3	3800001	3900000	4309
bdis3	3900001	4000000	4296
bdis3	4000001	4100000	4066
bdis3	4100001	4200000	3796
bdis3	4200001	4300000	3928
bdis3	4300001	4400000	4066
bdis3	4400001	4500000	5169
bdis3	4500001	4600000	4444
bdis3	4600001	4700000	4493
bdis3	4700001	4800000	3903
bdis3	4800001	4900000	5025
bdis3	4900001	5000000	3759
bdis3	5000001	5100000	4058
bdis3	5100001	5200000	3720
bdis3	5200001	5300000	3626
bdis3	5300001	5400000	4012
bdis3	5400001	5500000	4826
bdis3	5500001	5600000	4147
bdis3	5600001	5700000	3984
bdis3	5700001	5800000	4906
bdis3	5800001	5900000	4980
bdis3	5900001	6000000	3038

bdis1 4600001 4700000 4030
bdis1 4700001 4800000 3835
bdis1 4800001 4900000 4169
bdis1 4900001 5000000 4258
bdis1 5000001 5100000 4868
bdis1 5100001 5200000 4534
bdis1 5200001 5300000 4802
bdis1 5300001 5400000 4228
bdis1 5400001 5500000 4403
bdis1 5500001 5600000 5016
bdis1 5600001 5700000 3940
bdis1 5700001 5800000 4962
bdis1 5800001 5900000 3404
bdis1 5900001 6000000 4769
bdis1 6000001 6100000 4599
bdis1 6100001 6200000 4501
bdis1 6200001 6300000 3912
bdis1 6300001 6400000 4238
bdis1 6400001 6500000 5257
bdis1 6500001 6600000 4686
bdis1 6600001 6700000 4285
bdis1 6700001 6800000 3743
bdis1 6800001 6900000 4874
bdis1 6900001 7000000 3611
bdis1 7000001 7100000 4904
bdis1 7100001 7200000 4491
bdis1 7200001 7300000 4150
bdis1 7300001 7400000 3527
bdis1 7400001 7500000 4762
bdis1 7500001 7600000 4046
bdis1 7600001 7700000 4294
bdis1 7700001 7800000 4215
bdis1 7800001 7900000 5691
bdis1 7900001 8000000 3734
bdis1 8000001 8100000 4428
bdis1 8100001 8200000 4875
bdis1 8200001 8300000 4358
bdis1 8300001 8400000 4082
bdis1 8400001 8500000 4405
bdis1 8500001 8600000 4014
bdis1 8600001 8700000 4326
bdis1 8700001 8800000 4523
bdis1 8800001 8900000 4621
bdis1 8900001 9000000 3959
bdis1 9000001 9100000 4229
bdis1 9100001 9200000 3745
bdis1 9200001 9300000 4055
bdis1 9300001 9400000 3500
bdis3 6000001 6100000 4331
bdis3 6100001 6200000 3622
bdis3 6200001 6300000 4699
bdis3 6300001 6400000 4064
bdis3 6400001 6500000 3635
bdis3 6500001 6600000 3700
bdis3 6600001 6700000 4274
bdis3 6700001 6800000 3902
bdis3 6800001 6900000 4308
bdis3 6900001 7000000 3710
bdis3 7000001 7100000 3465
bdis3 7100001 7200000 4823
bdis3 7200001 7300000 3738
bdis3 7300001 7400000 4450
bdis3 7400001 7500000 4650
bdis3 7500001 7600000 4225
bdis3 7600001 7700000 5143
bdis3 7700001 7800000 4403
bdis3 7800001 7900000 3594
bdis3 7900001 8000000 4129
bdis3 8000001 8100000 4161
bdis3 8100001 8200000 3530
bdis3 8200001 8300000 3200
bdis3 8300001 8400000 5072
bdis3 8400001 8500000 3052
bdis3 8500001 8600000 4736
bdis3 8600001 8700000 4019
bdis3 8700001 8800000 3844
bdis3 8800001 8900000 4497
bdis3 8900001 9000000 4995
bdis3 9000001 9100000 4447
bdis3 9100001 9200000 3908
bdis3 9200001 9300000 4436
bdis3 9300001 9400000 5379
bdis3 9400001 9500000 3385
bdis3 9500001 9600000 2974
bdis3 9600001 9700000 4498
bdis3 9700001 9800000 3958
bdis3 9800001 9900000 4251
bdis3 9900001 10000000 4862
bdis3 10000001 10100000 3827
bdis3 10100001 10200000 5419
bdis3 10200001 10300000 3895
bdis3 10300001 10400000 5419
bdis3 10400001 10500000 5786
bdis3 10500001 10600000 5477
bdis3 10600001 10700000 4442
bdis3 10700001 10800000 5217

bdis1 9400001 9500000 3684
bdis1 9500001 9600000 4205
bdis1 9600001 9700000 4413
bdis1 9700001 9800000 3904
bdis1 9800001 9900000 4670
bdis1 9900001 10000000 5144
bdis1 10000001 10100000 4373
bdis1 10100001 10200000 5135
bdis1 10200001 10300000 3719
bdis1 10300001 10400000 5158
bdis1 10400001 10500000 4012
bdis1 10500001 10600000 3482
bdis1 10600001 10700000 5052
bdis1 10700001 10800000 3431
bdis1 10800001 10900000 5099
bdis1 10900001 11000000 4637
bdis1 11000001 11100000 3926
bdis1 11100001 11200000 4270
bdis1 11200001 11300000 4868
bdis1 11300001 11400000 3512
bdis1 11400001 11500000 3533
bdis1 11500001 11600000 3535
bdis1 11600001 11700000 4059
bdis1 11700001 11800000 5524
bdis1 11800001 11900000 4796
bdis1 11900001 12000000 3474
bdis1 12000001 12100000 3443
bdis1 12100001 12200000 3703
bdis1 12200001 12300000 3274
bdis1 12300001 12400000 3563
bdis1 12400001 12500000 4092
bdis1 12500001 12600000 3949
bdis1 12600001 12700000 5002
bdis1 12700001 12800000 4140
bdis1 12800001 12900000 4135
bdis1 12900001 13000000 3816
bdis1 13000001 13100000 4202
bdis1 13100001 13200000 4858
bdis1 13200001 13300000 6763
bdis1 13300001 13400000 4707
bdis1 13400001 13500000 4077
bdis1 13500001 13600000 5584
bdis1 13600001 13700000 4812
bdis1 13700001 13800000 3862
bdis1 13800001 13900000 4942
bdis1 13900001 14000000 4800
bdis1 14000001 14100000 6073
bdis1 14100001 14200000 4202
bdis3 10800001 10900000 4491
bdis3 10900001 11000000 6013
bdis3 11000001 11100000 6093
bdis3 11100001 11200000 5448
bdis3 11200001 11300000 5056
bdis3 11300001 11400000 4104
bdis3 11400001 11500000 4058
bdis3 11500001 11600000 3394
bdis3 11600001 11700000 3974
bdis3 11700001 11800000 3391
bdis3 11800001 11900000 5810
bdis3 11900001 12000000 4758
bdis3 12000001 12100000 4568
bdis3 12100001 12200000 3850
bdis3 12200001 12300000 4946
bdis3 12300001 12400000 4534
bdis3 12400001 12500000 4198
bdis3 12500001 12600000 4098
bdis3 12600001 12700000 3996
bdis3 12700001 12800000 4904
bdis3 12800001 12900000 4245
bdis3 12900001 13000000 5279
bdis3 13000001 13100000 4172
bdis3 13100001 13200000 3526
bdis3 13200001 13300000 4517
bdis3 13300001 13400000 4916
bdis3 13400001 13500000 3537
bdis3 13500001 13600000 4574
bdis3 13600001 13700000 3324
bdis3 13700001 13800000 3599
bdis3 13800001 13900000 5355
bdis3 13900001 14000000 4857
bdis3 14000001 14100000 3726
bdis3 14100001 14200000 4721
bdis3 14200001 14300000 3975
bdis3 14300001 14400000 3961
bdis3 14400001 14500000 4537
bdis3 14500001 14600000 4021
bdis3 14600001 14700000 4119
bdis3 14700001 14800000 3956
bdis3 14800001 14900000 4459
bdis3 14900001 15000000 3483
bdis3 15000001 15100000 2894
bdis3 15100001 15200000 3829
bdis3 15200001 15300000 5154
bdis3 15300001 15400000 4079
bdis3 15400001 15500000 5018
bdis3 15500001 15600000 5126

bdis1 14200001 14300000 4509
bdis1 14300001 14400000 5587
bdis1 14400001 14500000 4742
bdis1 14500001 14600000 4249
bdis1 14600001 14700000 4681
bdis1 14700001 14800000 5242
bdis1 14800001 14900000 3906
bdis1 14900001 15000000 3794
bdis1 15000001 15100000 3835
bdis1 15100001 15200000 3452
bdis1 15200001 15300000 4271
bdis1 15300001 15400000 4672
bdis1 15400001 15500000 4525
bdis1 15500001 15600000 3868
bdis1 15600001 15700000 5062
bdis1 15700001 15800000 4022
bdis1 15800001 15900000 5046
bdis1 15900001 16000000 3664
bdis1 16000001 16100000 4835
bdis1 16100001 16200000 3896
bdis1 16200001 16300000 5380
bdis1 16300001 16400000 4551
bdis1 16400001 16500000 4218
bdis1 16500001 16600000 5529
bdis1 16600001 16700000 4690
bdis1 16700001 16800000 4488
bdis1 16800001 16900000 6140
bdis1 16900001 17000000 4298
bdis1 17000001 17100000 4190
bdis1 17100001 17200000 4235
bdis1 17200001 17300000 4187
bdis1 17300001 17400000 4329
bdis1 17400001 17500000 3920
bdis1 17500001 17600000 4452
bdis1 17600001 17700000 5393
bdis1 17700001 17800000 4851
bdis1 17800001 17900000 5189
bdis1 17900001 18000000 5856
bdis1 18000001 18100000 3914
bdis1 18100001 18200000 4151
bdis1 18200001 18300000 5012
bdis1 18300001 18400000 5250
bdis1 18400001 18500000 5014
bdis1 18500001 18600000 4755
bdis1 18600001 18700000 5323
bdis1 18700001 18800000 5319
bdis1 18800001 18900000 4371
bdis1 18900001 19000000 4906
bdis3 15600001 15700000 3841
bdis3 15700001 15800000 4381
bdis3 15800001 15900000 4158
bdis3 15900001 16000000 4045
bdis3 16000001 16100000 3907
bdis3 16100001 16200000 4458
bdis3 16200001 16300000 3783
bdis3 16300001 16400000 5193
bdis3 16400001 16500000 3867
bdis3 16500001 16600000 5213
bdis3 16600001 16700000 3817
bdis3 16700001 16800000 4726
bdis3 16800001 16900000 4654
bdis3 16900001 17000000 4952
bdis3 17000001 17100000 4436
bdis3 17100001 17200000 3812
bdis3 17200001 17300000 4488
bdis3 17300001 17400000 3896
bdis3 17400001 17500000 3961
bdis3 17500001 17600000 3692
bdis3 17600001 17700000 4163
bdis3 17700001 17800000 4822
bdis3 17800001 17900000 5703
bdis3 17900001 18000000 4712
bdis3 18000001 18100000 4314
bdis3 18100001 18200000 3837
bdis3 18200001 18300000 3615
bdis3 18300001 18400000 4559
bdis3 18400001 18500000 3901
bdis3 18500001 18600000 5089
bdis3 18600001 18700000 4311
bdis3 18700001 18800000 3722
bdis3 18800001 18900000 3917
bdis3 18900001 19000000 3526
bdis3 19000001 19100000 4151
bdis3 19100001 19200000 4343
bdis3 19200001 19300000 5016
bdis3 19300001 19400000 2874
bdis3 19400001 19500000 3267
bdis3 19500001 19600000 3672
bdis3 19600001 19700000 3829
bdis3 19700001 19800000 4737
bdis3 19800001 19900000 4151
bdis3 19900001 20000000 5288
bdis3 20000001 20100000 4069
bdis3 20100001 20200000 5032
bdis3 20200001 20300000 4607
bdis3 20300001 20400000 3927

bdis1 19000001 19100000 4923
bdis1 19100001 19200000 4618
bdis1 19200001 19300000 4440
bdis1 19300001 19400000 3720
bdis1 19400001 19500000 4622
bdis1 19500001 19600000 4869
bdis1 19600001 19700000 5361
bdis1 19700001 19800000 4692
bdis1 19800001 19900000 3363
bdis1 19900001 20000000 4208
bdis1 20000001 20100000 5384
bdis1 20100001 20200000 3848
bdis1 20200001 20300000 4097
bdis1 20300001 20400000 4338
bdis1 20400001 20500000 4203
bdis1 20500001 20600000 3998
bdis1 20600001 20700000 4446
bdis1 20700001 20800000 6031
bdis1 20800001 20900000 4379
bdis1 20900001 21000000 3907
bdis1 21000001 21100000 4972
bdis1 21100001 21200000 3914
bdis1 21200001 21300000 4194
bdis1 21300001 21400000 3647
bdis1 21400001 21500000 4680
bdis1 21500001 21600000 4082
bdis1 21600001 21700000 3500
bdis1 21700001 21800000 4803
bdis1 21800001 21900000 5234
bdis1 21900001 22000000 4919
bdis1 22000001 22100000 3604
bdis1 22100001 22200000 3728
bdis1 22200001 22300000 3839
bdis1 22300001 22400000 4330
bdis1 22400001 22500000 4269
bdis1 22500001 22600000 5350
bdis1 22600001 22700000 5778
bdis1 22700001 22800000 5714
bdis1 22800001 22900000 3380
bdis1 22900001 23000000 5623
bdis1 23000001 23100000 4291
bdis1 23100001 23200000 3918
bdis1 23200001 23300000 3828
bdis1 23300001 23400000 3773
bdis1 23400001 23500000 4753
bdis1 23500001 23600000 4573
bdis1 23600001 23700000 3578
bdis1 23700001 23800000 6070
bdis3 20400001 20500000 4415
bdis3 20500001 20600000 3872
bdis3 20600001 20700000 3978
bdis3 20700001 20800000 4725
bdis3 20800001 20900000 5570
bdis3 20900001 21000000 4015
bdis3 21000001 21100000 4149
bdis3 21100001 21200000 5331
bdis3 21200001 21300000 4097
bdis3 21300001 21400000 3161
bdis3 21400001 21500000 3268
bdis3 21500001 21600000 5348
bdis3 21600001 21700000 4387
bdis3 21700001 21800000 4574
bdis3 21800001 21900000 3461
bdis3 21900001 22000000 4555
bdis3 22000001 22100000 3497
bdis3 22100001 22200000 4652
bdis3 22200001 22300000 4889
bdis3 22300001 22400000 5239
bdis3 22400001 22500000 5408
bdis3 22500001 22600000 4798
bdis3 22600001 22700000 3791
bdis3 22700001 22800000 3920
bdis3 22800001 22900000 5407
bdis3 22900001 23000000 4248
bdis3 23000001 23100000 5203
bdis3 23100001 23200000 5886
bdis3 23200001 23300000 4355
bdis3 23300001 23400000 5038
bdis3 23400001 23500000 5031
bdis3 23500001 23600000 6608
bdis3 23600001 23700000 5496
bdis3 23700001 23800000 5112
bdis3 23800001 23900000 5238
bdis3 23900001 24000000 5774
bdis3 24000001 24100000 6622
bdis3 24100001 24200000 4885
bdis3 24200001 24300000 3776
bdis3 24300001 24400000 4654
bdis3 24400001 24500000 5391
bdis3 24500001 24600000 3887
bdis3 24600001 24700000 6348
bdis3 24700001 24800000 5599
bdis3 24800001 24900000 6400
bdis3 24900001 25000000 5223
bdis3 25000001 25100000 4308
bdis3 25100001 25200000 3485

bdis1 23800001 23900000 3161
bdis1 23900001 24000000 3512
bdis1 24000001 24100000 4102
bdis1 24100001 24200000 3872
bdis1 24200001 24300000 3542
bdis1 24300001 24400000 3224
bdis1 24400001 24500000 4728
bdis1 24500001 24600000 3969
bdis1 24600001 24700000 4442
bdis1 24700001 24800000 3398
bdis1 24800001 24900000 4748
bdis1 24900001 25000000 4615
bdis1 25000001 25100000 3810
bdis1 25100001 25200000 3325
bdis1 25200001 25300000 4572
bdis1 25300001 25400000 4148
bdis1 25400001 25500000 3554
bdis1 25500001 25600000 5271
bdis1 25600001 25700000 4763
bdis1 25700001 25800000 4106
bdis1 25800001 25900000 3457
bdis1 25900001 26000000 4031
bdis1 26000001 26100000 4690
bdis1 26100001 26200000 4666
bdis1 26200001 26300000 4318
bdis1 26300001 26400000 5646
bdis1 26400001 26500000 4518
bdis1 26500001 26600000 4664
bdis1 26600001 26700000 5227
bdis1 26700001 26800000 5189
bdis1 26800001 26900000 4233
bdis1 26900001 27000000 4362
bdis1 27000001 27100000 4812
bdis1 27100001 27200000 4329
bdis1 27200001 27300000 4952
bdis1 27300001 27400000 3753
bdis1 27400001 27500000 4800
bdis1 27500001 27600000 4761
bdis1 27600001 27700000 3517
bdis1 27700001 27800000 4656
bdis1 27800001 27900000 4163
bdis1 27900001 28000000 4080
bdis1 28000001 28100000 4328
bdis1 28100001 28200000 4101
bdis1 28200001 28300000 4289
bdis1 28300001 28400000 4991
bdis1 28400001 28500000 4382
bdis1 28500001 28600000 3774
bdis3 25200001 25300000 4799
bdis3 25300001 25400000 2808
bdis3 25400001 25500000 3532
bdis3 25500001 25600000 4861
bdis3 25600001 25700000 5046
bdis3 25700001 25800000 4379
bdis3 25800001 25900000 4208
bdis3 25900001 26000000 4599
bdis3 26000001 26100000 6301
bdis3 26100001 26200000 4720
bdis3 26200001 26300000 4004
bdis3 26300001 26400000 4333
bdis3 26400001 26500000 4206
bdis3 26500001 26600000 5405
bdis3 26600001 26700000 5837
bdis3 26700001 26800000 5373
bdis3 26800001 26900000 5725
bdis3 26900001 27000000 5224
bdis3 27000001 27100000 4166
bdis3 27100001 27200000 5850
bdis3 27200001 27300000 5290
bdis3 27300001 27400000 4276
bdis3 27400001 27500000 4205
bdis3 27500001 27600000 4954
bdis3 27600001 27700000 3753
bdis3 27700001 27800000 6320
bdis3 27800001 27900000 4516
bdis3 27900001 28000000 5311
bdis3 28000001 28100000 4767
bdis3 28100001 28200000 4992
bdis3 28200001 28300000 4622
bdis3 28300001 28400000 5294
bdis3 28400001 28500000 5759
bdis3 28500001 28600000 3904
bdis3 28600001 28700000 4501
bdis3 28700001 28800000 3520
bdis3 28800001 28900000 4882
bdis3 28900001 29000000 4202
bdis3 29000001 29100000 3542
bdis3 29100001 29200000 4699
bdis3 29200001 29300000 5453
bdis3 29300001 29400000 5184
bdis3 29400001 29500000 4110
bdis3 29500001 29600000 4340
bdis3 29600001 29700000 5606
bdis3 29700001 29800000 4080
bdis3 29800001 29900000 4612
bdis3 29900001 30000000 4600

bdis1 28600001 28700000 3825
bdis1 28700001 28800000 4604
bdis1 28800001 28900000 4598
bdis1 28900001 29000000 3525
bdis1 29000001 29100000 4865
bdis1 29100001 29200000 5115
bdis1 29200001 29300000 5133
bdis1 29300001 29400000 5188
bdis1 29400001 29500000 6420
bdis1 29500001 29600000 4776
bdis1 29600001 29700000 3472
bdis1 29700001 29800000 3902
bdis1 29800001 29900000 3635
bdis1 29900001 30000000 4084
bdis1 30000001 30100000 4828
bdis1 30100001 30200000 4293
bdis1 30200001 30300000 5336
bdis1 30300001 30400000 4085
bdis1 30400001 30500000 4599
bdis1 30500001 30600000 4354
bdis1 30600001 30700000 4098
bdis1 30700001 30800000 4455
bdis1 30800001 30900000 5642
bdis1 30900001 31000000 4577
bdis1 31000001 31100000 5824
bdis1 31100001 31200000 4241
bdis1 31200001 31300000 4147
bdis1 31300001 31400000 3653
bdis1 31400001 31500000 3720
bdis1 31500001 31600000 3669
bdis1 31600001 31700000 5108
bdis1 31700001 31800000 6031
bdis1 31800001 31900000 4232
bdis1 31900001 32000000 4465
bdis1 32000001 32100000 4062
bdis1 32100001 32200000 4005
bdis1 32200001 32300000 4200
bdis1 32300001 32400000 4738
bdis1 32400001 32500000 5719
bdis1 32500001 32600000 4015
bdis1 32600001 32700000 4184
bdis1 32700001 32800000 4913
bdis1 32800001 32900000 4904
bdis1 32900001 33000000 4161
bdis1 33000001 33100000 3901
bdis1 33100001 33200000 4375
bdis1 33200001 33300000 3834
bdis1 33300001 33400000 3987
bdis3 30000001 30100000 3699
bdis3 30100001 30200000 5647
bdis3 30200001 30300000 4284
bdis3 30300001 30400000 5089
bdis3 30400001 30500000 4806
bdis3 30500001 30600000 4336
bdis3 30600001 30700000 4633
bdis3 30700001 30800000 5777
bdis3 30800001 30900000 6030
bdis3 30900001 31000000 4288
bdis3 31000001 31100000 4480
bdis3 31100001 31200000 4304
bdis3 31200001 31300000 4375
bdis3 31300001 31400000 4277
bdis3 31400001 31500000 4339
bdis3 31500001 31600000 5828
bdis3 31600001 31700000 3810
bdis3 31700001 31800000 4506
bdis3 31800001 31900000 3299
bdis3 31900001 32000000 3799
bdis3 32000001 32100000 5501
bdis3 32100001 32200000 4071
bdis3 32200001 32300000 4843
bdis3 32300001 32400000 4838
bdis3 32400001 32500000 5418
bdis3 32500001 32600000 4775
bdis3 32600001 32700000 4305
bdis3 32700001 32800000 4255
bdis3 32800001 32900000 3837
bdis3 32900001 33000000 4426
bdis3 33000001 33100000 5540
bdis3 33100001 33200000 5404
bdis3 33200001 33300000 5651
bdis3 33300001 33400000 6412
bdis3 33400001 33500000 5428
bdis3 33500001 33600000 5186
bdis3 33600001 33700000 5030
bdis3 33700001 33800000 5716
bdis3 33800001 33900000 5396
bdis3 33900001 34000000 5018
bdis3 34000001 34100000 5572
bdis3 34100001 34200000 5809
bdis3 34200001 34300000 6004
bdis3 34300001 34400000 5017
bdis3 34400001 34500000 4557
bdis3 34500001 34600000 4314
bdis3 34600001 34700000 5596
bdis3 34700001 34800000 4309

bdis1 33400001 33500000 4210
bdis1 33500001 33600000 3891
bdis1 33600001 33700000 3310
bdis1 33700001 33800000 4169
bdis1 33800001 33900000 3827
bdis1 33900001 34000000 4080
bdis1 34000001 34100000 4487
bdis1 34100001 34200000 4544
bdis1 34200001 34300000 4808
bdis1 34300001 34400000 3625
bdis1 34400001 34500000 3962
bdis1 34500001 34600000 6535
bdis1 34600001 34700000 4353
bdis1 34700001 34800000 5067
bdis1 34800001 34900000 5540
bdis1 34900001 35000000 4363
bdis1 35000001 35100000 4500
bdis1 35100001 35200000 5904
bdis1 35200001 35300000 5399
bdis1 35300001 35400000 6121
bdis1 35400001 35500000 4984
bdis1 35500001 35600000 5408
bdis1 35600001 35700000 4061
bdis1 35700001 35800000 3527
bdis1 35800001 35900000 2798
bdis1 35900001 36000000 2826
bdis1 36000001 36100000 6202
bdis1 36100001 36200000 5052
bdis1 36200001 36300000 6000
bdis1 36300001 36400000 7309
bdis1 36400001 36500000 6805
bdis1 36500001 36600000 5873
bdis1 36600001 36700000 4770
bdis1 36700001 36800000 5627
bdis1 36800001 36900000 6361
bdis1 36900001 37000000 6322
bdis1 37000001 37100000 5156
bdis1 37100001 37200000 5444
bdis1 37200001 37300000 6813
bdis1 37300001 37400000 4879
bdis1 37400001 37500000 5060
bdis1 37500001 37600000 4701
bdis1 37600001 37700000 6049
bdis1 37700001 37800000 4415
bdis1 37800001 37900000 4470
bdis1 37900001 38000000 4475
bdis1 38000001 38100000 4705
bdis1 38100001 38200000 4143
bdis3 34800001 34900000 5801
bdis3 34900001 35000000 5123
bdis3 35000001 35100000 3959
bdis3 35100001 35200000 4843
bdis3 35200001 35300000 4760
bdis3 35300001 35400000 5550
bdis3 35400001 35500000 4600
bdis3 35500001 35600000 4780
bdis3 35600001 35700000 4317
bdis3 35700001 35800000 4443
bdis3 35800001 35900000 3752
bdis3 35900001 36000000 4679
bdis3 36000001 36100000 3468
bdis3 36100001 36200000 4290
bdis3 36200001 36300000 4913
bdis3 36300001 36400000 3712
bdis3 36400001 36500000 4710
bdis3 36500001 36600000 4622
bdis3 36600001 36700000 4896
bdis3 36700001 36800000 3866
bdis3 36800001 36900000 3541
bdis3 36900001 37000000 3164
bdis3 37000001 37100000 3779
bdis3 37100001 37200000 3494
bdis3 37200001 37300000 4038
bdis3 37300001 37400000 3888
bdis3 37400001 37500000 4200
bdis3 37500001 37600000 3826
bdis3 37600001 37700000 5188
bdis3 37700001 37800000 4897
bdis3 37800001 37900000 5337
bdis3 37900001 38000000 5007
bdis3 38000001 38100000 4410
bdis3 38100001 38200000 4705
bdis3 38200001 38300000 4758
bdis3 38300001 38400000 5347
bdis3 38400001 38500000 4026
bdis3 38500001 38600000 6551
bdis3 38600001 38700000 4101
bdis3 38700001 38800000 3896
bdis3 38800001 38900000 5320
bdis3 38900001 39000000 4134
bdis3 39000001 39100000 4406
bdis3 39100001 39200000 4576
bdis3 39200001 39300000 4265
bdis3 39300001 39400000 4229
bdis3 39400001 39500000 5340
bdis3 39500001 39600000 5451

bdis1 38200001 38300000 4893
bdis1 38300001 38400000 4985
bdis1 38400001 38500000 4409
bdis1 38500001 38600000 5106
bdis1 38600001 38700000 3644
bdis1 38700001 38800000 3708
bdis1 38800001 38900000 5034
bdis1 38900001 39000000 6550
bdis1 39000001 39100000 5216
bdis1 39100001 39200000 5894
bdis1 39200001 39300000 4089
bdis1 39300001 39400000 3271
bdis1 39400001 39500000 4504
bdis1 39500001 39600000 5107
bdis1 39600001 39700000 5619
bdis1 39700001 39800000 4483
bdis1 39800001 39900000 5173
bdis1 39900001 40000000 4270
bdis1 40000001 40100000 3421
bdis1 40100001 40200000 3485
bdis1 40200001 40300000 5085
bdis1 40300001 40400000 4185
bdis1 40400001 40500000 3815
bdis1 40500001 40600000 5367
bdis1 40600001 40700000 4008
bdis1 40700001 40800000 5016
bdis1 40800001 40900000 3181
bdis1 40900001 41000000 4817
bdis1 41000001 41100000 4047
bdis1 41100001 41200000 5495
bdis1 41200001 41300000 4491
bdis1 41300001 41400000 4113
bdis1 41400001 41500000 3390
bdis1 41500001 41600000 4811
bdis1 41600001 41700000 3551
bdis1 41700001 41800000 5345
bdis1 41800001 41900000 5108
bdis1 41900001 42000000 5810
bdis1 42000001 42100000 6217
bdis1 42100001 42200000 4295
bdis1 42200001 42300000 3472
bdis1 42300001 42400000 4918
bdis1 42400001 42500000 4474
bdis1 42500001 42600000 4526
bdis1 42600001 42700000 3740
bdis1 42700001 42800000 3801
bdis1 42800001 42900000 4263
bdis1 42900001 43000000 4853
bdis3 39600001 39700000 5119
bdis3 39700001 39800000 6210
bdis3 39800001 39900000 6153
bdis3 39900001 40000000 5006
bdis3 40000001 40100000 4542
bdis3 40100001 40200000 5428
bdis3 40200001 40300000 5073
bdis3 40300001 40400000 4734
bdis3 40400001 40500000 5534
bdis3 40500001 40600000 5799
bdis3 40600001 40700000 4238
bdis3 40700001 40800000 5639
bdis3 40800001 40900000 4746
bdis3 40900001 41000000 4648
bdis3 41000001 41100000 5062
bdis3 41100001 41200000 4192
bdis3 41200001 41300000 4367
bdis3 41300001 41400000 4739
bdis3 41400001 41500000 5047
bdis3 41500001 41600000 4644
bdis3 41600001 41700000 3994
bdis3 41700001 41800000 3687
bdis3 41800001 41900000 3995
bdis3 41900001 42000000 5466
bdis3 42000001 42100000 4747
bdis3 42100001 42200000 5470
bdis3 42200001 42300000 4851
bdis3 42300001 42400000 4355
bdis3 42400001 42500000 4896
bdis3 42500001 42600000 4610
bdis3 42600001 42700000 3838
bdis3 42700001 42800000 4815
bdis3 42800001 42900000 5065
bdis3 42900001 43000000 4341
bdis3 43000001 43100000 4352
bdis3 43100001 43200000 4574
bdis3 43200001 43300000 4160
bdis3 43300001 43400000 3895
bdis3 43400001 43500000 4138
bdis3 43500001 43600000 3787
bdis3 43600001 43700000 4844
bdis3 43700001 43800000 4662
bdis3 43800001 43900000 5170
bdis3 43900001 44000000 4010
bdis3 44000001 44100000 3860
bdis3 44100001 44200000 3702
bdis3 44200001 44300000 3862
bdis3 44300001 44400000 5341

bdis1 43000001 43100000 4626
bdis1 43100001 43200000 5049
bdis1 43200001 43300000 4272
bdis1 43300001 43400000 4273
bdis1 43400001 43500000 4424
bdis1 43500001 43600000 3799
bdis1 43600001 43700000 3996
bdis1 43700001 43800000 5002
bdis1 43800001 43900000 5091
bdis1 43900001 44000000 4102
bdis1 44000001 44100000 4414
bdis1 44100001 44200000 4254
bdis1 44200001 44300000 5051
bdis1 44300001 44400000 5377
bdis1 44400001 44500000 5613
bdis1 44500001 44600000 5856
bdis1 44600001 44700000 6000
bdis1 44700001 44800000 4465
bdis1 44800001 44900000 4093
bdis1 44900001 45000000 4367
bdis1 45000001 45100000 4155
bdis1 45100001 45200000 4675
bdis1 45200001 45300000 3261
bdis1 45300001 45400000 4521
bdis1 45400001 45500000 3835
bdis1 45500001 45600000 4972
bdis1 45600001 45700000 4465
bdis1 45700001 45800000 5855
bdis1 45800001 45900000 4424
bdis1 45900001 46000000 5599
bdis1 46000001 46100000 4080
bdis1 46100001 46200000 6078
bdis1 46200001 46300000 4385
bdis1 46300001 46400000 4937
bdis1 46400001 46500000 4172
bdis1 46500001 46600000 4456
bdis1 46600001 46700000 3924
bdis1 46700001 46800000 4385
bdis1 46800001 46900000 4312
bdis1 46900001 47000000 5015
bdis1 47000001 47100000 4391
bdis1 47100001 47200000 4496
bdis1 47200001 47300000 3964
bdis1 47300001 47400000 4708
bdis1 47400001 47500000 3993
bdis1 47500001 47600000 6016
bdis1 47600001 47700000 5086
bdis1 47700001 47800000 5613
bdis3 44400001 44500000 4442
bdis3 44500001 44600000 3836
bdis3 44600001 44700000 2759
bdis3 44700001 44800000 3839
bdis3 44800001 44900000 3828
bdis3 44900001 45000000 4230
bdis3 45000001 45100000 4412
bdis3 45100001 45200000 4079
bdis3 45200001 45300000 4630
bdis3 45300001 45400000 4508
bdis3 45400001 45500000 3843
bdis3 45500001 45600000 4403
bdis3 45600001 45700000 4378
bdis3 45700001 45800000 5682
bdis3 45800001 45900000 4354
bdis3 45900001 46000000 3743
bdis3 46000001 46100000 3525
bdis3 46100001 46200000 4269
bdis3 46200001 46300000 4460
bdis3 46300001 46400000 4020
bdis3 46400001 46500000 4892
bdis3 46500001 46600000 3765
bdis3 46600001 46700000 4171
bdis3 46700001 46800000 4373
bdis3 46800001 46900000 4546
bdis3 46900001 47000000 4805
bdis3 47000001 47100000 4697
bdis3 47100001 47200000 4187
bdis3 47200001 47300000 5552
bdis3 47300001 47400000 4421
bdis3 47400001 47500000 4461
bdis3 47500001 47600000 3335
bdis3 47600001 47700000 3480
bdis3 47700001 47800000 3579
bdis3 47800001 47900000 4249
bdis3 47900001 48000000 5222
bdis3 48000001 48100000 4941
bdis3 48100001 48200000 3819
bdis3 48200001 48300000 5339
bdis3 48300001 48400000 4201
bdis3 48400001 48500000 4518
bdis3 48500001 48600000 4329
bdis3 48600001 48700000 3348
bdis3 48700001 48800000 4501
bdis3 48800001 48900000 4036
bdis3 48900001 49000000 3634
bdis3 49000001 49100000 3600
bdis3 49100001 49200000 4347

bdis1 47800001 47900000 4502
bdis1 47900001 48000000 4224
bdis1 48000001 48100000 4416
bdis1 48100001 48200000 5537
bdis1 48200001 48300000 4117
bdis1 48300001 48400000 4257
bdis1 48400001 48500000 5641
bdis1 48500001 48600000 4583
bdis1 48600001 48700000 4464
bdis1 48700001 48800000 5025
bdis1 48800001 48900000 3973
bdis1 48900001 49000000 3237
bdis1 49000001 49100000 5340
bdis1 49100001 49200000 3903
bdis1 49200001 49300000 4931
bdis1 49300001 49400000 4572
bdis1 49400001 49500000 3691
bdis1 49500001 49600000 4180
bdis1 49600001 49700000 3770
bdis1 49700001 49800000 4413
bdis1 49800001 49900000 4787
bdis1 49900001 50000000 3289
bdis1 50000001 50100000 3767
bdis1 50100001 50200000 4173
bdis1 50200001 50300000 3252
bdis1 50300001 50400000 4621
bdis1 50400001 50500000 4420
bdis1 50500001 50600000 4062
bdis1 50600001 50700000 3637
bdis1 50700001 50800000 4028
bdis1 50800001 50900000 3964
bdis1 50900001 51000000 3187
bdis1 51000001 51100000 2971
bdis1 51100001 51200000 4376
bdis1 51200001 51300000 4951
bdis1 51300001 51400000 5319
bdis1 51400001 51500000 2909
bdis1 51500001 51600000 4031
bdis1 51600001 51700000 4901
bdis1 51700001 51800000 3649
bdis1 51800001 51900000 4338
bdis1 51900001 52000000 5542
bdis1 52000001 52100000 4231
bdis1 52100001 52200000 4234
bdis1 52200001 52300000 4464
bdis1 52300001 52400000 3749
bdis1 52400001 52500000 5069
bdis1 52500001 52600000 3877
bdis3 49200001 49300000 4435
bdis3 49300001 49400000 3495
bdis3 49400001 49500000 4464
bdis3 49500001 49600000 4076
bdis3 49600001 49700000 4360
bdis3 49700001 49800000 4792
bdis3 49800001 49900000 4060
bdis3 49900001 50000000 3935
bdis3 50000001 50100000 4391
bdis3 50100001 50200000 3493
bdis3 50200001 50300000 4342
bdis3 50300001 50400000 3872
bdis3 50400001 50500000 4435
bdis3 50500001 50600000 4680
bdis3 50600001 50700000 4074
bdis3 50700001 50800000 4106
bdis3 50800001 50900000 3818
bdis3 50900001 51000000 4631
bdis3 51000001 51100000 4538
bdis3 51100001 51200000 4498
bdis3 51200001 51300000 4673
bdis3 51300001 51400000 5263
bdis3 51400001 51500000 4104
bdis3 51500001 51600000 4631
bdis3 51600001 51700000 4627
bdis3 51700001 51800000 4852
bdis3 51800001 51900000 5443
bdis3 51900001 52000000 3373
bdis3 52000001 52100000 4238
bdis3 52100001 52200000 4519
bdis3 52200001 52300000 4752
bdis3 52300001 52400000 3854
bdis3 52400001 52500000 4471
bdis3 52500001 52600000 4209
bdis3 52600001 52700000 4706
bdis3 52700001 52800000 4908
bdis3 52800001 52900000 4527
bdis3 52900001 53000000 4807
bdis3 53000001 53100000 4718
bdis3 53100001 53200000 4567
bdis3 53200001 53300000 4192
bdis3 53300001 53400000 4679
bdis3 53400001 53500000 4383
bdis3 53500001 53600000 4301
bdis3 53600001 53700000 3483
bdis3 53700001 53800000 4027
bdis3 53800001 53900000 4551
bdis3 53900001 54000000 4374

bdis1 52600001 52700000 4230
bdis1 52700001 52800000 3145
bdis1 52800001 52900000 3844
bdis1 52900001 53000000 3650
bdis1 53000001 53100000 4659
bdis1 53100001 53200000 4319
bdis1 53200001 53300000 3514
bdis1 53300001 53400000 4263
bdis1 53400001 53500000 3564
bdis1 53500001 53600000 3633
bdis1 53600001 53700000 3559
bdis1 53700001 53800000 3428
bdis1 53800001 53900000 4217
bdis1 53900001 54000000 4050
bdis1 54000001 54100000 3909
bdis1 54100001 54200000 3953
bdis1 54200001 54300000 4214
bdis1 54300001 54400000 4606
bdis1 54400001 54500000 4719
bdis1 54500001 54600000 4249
bdis1 54600001 54700000 5380
bdis1 54700001 54800000 4190
bdis1 54800001 54900000 4674
bdis1 54900001 55000000 5025
bdis1 55000001 55100000 5747
bdis1 55100001 55200000 4446
bdis1 55200001 55300000 4466
bdis1 55300001 55400000 3775
bdis1 55400001 55500000 4979
bdis1 55500001 55600000 4352
bdis1 55600001 55700000 4219
bdis1 55700001 55800000 3609
bdis1 55800001 55900000 4477
bdis1 55900001 56000000 3924
bdis1 56000001 56100000 4442
bdis1 56100001 56200000 3621
bdis1 56200001 56300000 4009
bdis1 56300001 56400000 4120
bdis1 56400001 56500000 2978
bdis1 56500001 56600000 4924
bdis1 56600001 56700000 4245
bdis1 56700001 56800000 4687
bdis1 56800001 56900000 4111
bdis1 56900001 57000000 4286
bdis1 57000001 57100000 4657
bdis1 57100001 57200000 3486
bdis1 57200001 57300000 4125
bdis1 57300001 57400000 4888
bdis3 54000001 54100000 4059
bdis3 54100001 54200000 5003
bdis3 54200001 54300000 4078
bdis3 54300001 54400000 5090
bdis3 54400001 54500000 3756
bdis3 54500001 54600000 4402
bdis3 54600001 54700000 5107
bdis3 54700001 54800000 4476
bdis3 54800001 54900000 3518
bdis3 54900001 55000000 3596
bdis3 55000001 55100000 5130
bdis3 55100001 55200000 4491
bdis3 55200001 55300000 4993
bdis3 55300001 55400000 4499
bdis3 55400001 55500000 3720
bdis3 55500001 55600000 3789
bdis3 55600001 55700000 3774
bdis3 55700001 55800000 4528
bdis3 55800001 55900000 4436
bdis3 55900001 56000000 4264
bdis3 56000001 56100000 4962
bdis3 56100001 56200000 4151
bdis3 56200001 56300000 3974
bdis3 56300001 56400000 4433
bdis3 56400001 56500000 4384
bdis3 56500001 56600000 5071
bdis3 56600001 56700000 4245
bdis3 56700001 56800000 3989
bdis3 56800001 56900000 3747
bdis3 56900001 57000000 4605
bdis3 57000001 57100000 4960
bdis3 57100001 57200000 4885
bdis3 57200001 57300000 4405
bdis3 57300001 57400000 4436
bdis3 57400001 57500000 5272
bdis3 57500001 57600000 4889
bdis3 57600001 57700000 4516
bdis3 57700001 57800000 5558
bdis3 57800001 57900000 6431
bdis3 57900001 58000000 6491
bdis3 58000001 58100000 4987
bdis3 58100001 58200000 4573
bdis3 58200001 58300000 4491
bdis3 58300001 58400000 4557
bdis3 58400001 58500000 4418
bdis3 58500001 58600000 4746
bdis3 58600001 58700000 3719
bdis3 58700001 58800000 4623

bdis1 57400001 57500000 5207
bdis1 57500001 57600000 4309
bdis1 57600001 57700000 3106
bdis1 57700001 57800000 6347
bdis1 57800001 57900000 4350
bdis1 57900001 58000000 4078
bdis1 58000001 58100000 4385
bdis1 58100001 58200000 4016
bdis1 58200001 58300000 4591
bdis1 58300001 58400000 3500
bdis1 58400001 58500000 3798
bdis1 58500001 58600000 5456
bdis1 58600001 58700000 4761
bdis1 58700001 58800000 4142
bdis1 58800001 58900000 4345
bdis1 58900001 59000000 4646
bdis1 59000001 59100000 4621
bdis1 59100001 59200000 5143
bdis1 59200001 59300000 6253
bdis1 59300001 59400000 4082
bdis1 59400001 59500000 5541
bdis1 59500001 59600000 4037
bdis1 59600001 59700000 4287
bdis1 59700001 59800000 4215
bdis1 59800001 59900000 3351
bdis1 59900001 60000000 4327
bdis1 60000001 60100000 3990
bdis1 60100001 60200000 4291
bdis1 60200001 60300000 3716
bdis1 60300001 60400000 4365
bdis1 60400001 60500000 3855
bdis1 60500001 60600000 4336
bdis1 60600001 60700000 5266
bdis1 60700001 60800000 4571
bdis1 60800001 60900000 4005
bdis1 60900001 61000000 3732
bdis1 61000001 61100000 3940
bdis1 61100001 61200000 4407
bdis1 61200001 61300000 3848
bdis1 61300001 61400000 3362
bdis1 61400001 61500000 4304
bdis1 61500001 61600000 3433
bdis1 61600001 61700000 3837
bdis1 61700001 61800000 4221
bdis1 61800001 61900000 3461
bdis1 61900001 62000000 3755
bdis1 62000001 62100000 5220
bdis1 62100001 62200000 3973
bdis3 58800001 58900000 4164
bdis3 58900001 59000000 4455
bdis3 59000001 59100000 3895
bdis3 59100001 59200000 3958
bdis3 59200001 59300000 4079
bdis3 59300001 59400000 4466
bdis3 59400001 59500000 4066
bdis3 59500001 59600000 3956
bdis3 59600001 59700000 4030
bdis3 59700001 59800000 4030
bdis3 59800001 59900000 4030
bdis4 1 100000 5358
bdis4 100001 200000 4313
bdis4 200001 300000 4313
bdis4 300001 400000 3926
bdis4 400001 500000 4365
bdis4 500001 600000 4019
bdis4 600001 700000 3645
bdis4 700001 800000 3579
bdis4 800001 900000 3889
bdis4 900001 1000000 3692
bdis4 1000001 1100000 3919
bdis4 1100001 1200000 4042
bdis4 1200001 1300000 3503
bdis4 1300001 1400000 3573
bdis4 1400001 1500000 4725
bdis4 1500001 1600000 3797
bdis4 1600001 1700000 3685
bdis4 1700001 1800000 4898
bdis4 1800001 1900000 3812
bdis4 1900001 2000000 4251
bdis4 2000001 2100000 4036
bdis4 2100001 2200000 4297
bdis4 2200001 2300000 3952
bdis4 2300001 2400000 4088
bdis4 2400001 2500000 2964
bdis4 2500001 2600000 3855
bdis4 2600001 2700000 3532
bdis4 2700001 2800000 4087
bdis4 2800001 2900000 4099
bdis4 2900001 3000000 3566
bdis4 3000001 3100000 3408
bdis4 3100001 3200000 3568
bdis4 3200001 3300000 4253
bdis4 3300001 3400000 4608
bdis4 3400001 3500000 3543
bdis4 3500001 3600000 3938
bdis4 3600001 3700000 3558

bdis1 62200001 62300000 4593
bdis1 62300001 62400000 4559
bdis1 62400001 62500000 4067
bdis1 62500001 62600000 3447
bdis1 62600001 62700000 3817
bdis1 62700001 62800000 5162
bdis1 62800001 62900000 5138
bdis1 62900001 63000000 4342
bdis1 63000001 63100000 5263
bdis1 63100001 63200000 3749
bdis1 63200001 63300000 4384
bdis1 63300001 63400000 4262
bdis1 63400001 63500000 4095
bdis1 63500001 63600000 4220
bdis1 63600001 63700000 4250
bdis1 63700001 63800000 4287
bdis1 63800001 63900000 4730
bdis1 63900001 64000000 4104
bdis1 64000001 64100000 5426
bdis1 64100001 64200000 4385
bdis1 64200001 64300000 4595
bdis1 64300001 64400000 4019
bdis1 64400001 64500000 4112
bdis1 64500001 64600000 4875
bdis1 64600001 64700000 4134
bdis1 64700001 64800000 4603
bdis1 64800001 64900000 3672
bdis1 64900001 65000000 4726
bdis1 65000001 65100000 3788
bdis1 65100001 65200000 4298
bdis1 65200001 65300000 4404
bdis1 65300001 65400000 4437
bdis1 65400001 65500000 4580
bdis1 65500001 65600000 4479
bdis1 65600001 65700000 4635
bdis1 65700001 65800000 4417
bdis1 65800001 65900000 3608
bdis1 65900001 66000000 4321
bdis1 66000001 66100000 4257
bdis1 66100001 66200000 4365
bdis1 66200001 66300000 4366
bdis1 66300001 66400000 4297
bdis1 66400001 66500000 4808
bdis1 66500001 66600000 3884
bdis1 66600001 66700000 4416
bdis1 66700001 66800000 5064
bdis1 66800001 66900000 5762
bdis1 66900001 67000000 5476
bdis4 3700001 3800000 4115
bdis4 3800001 3900000 3467
bdis4 3900001 4000000 3944
bdis4 4000001 4100000 3669
bdis4 4100001 4200000 3747
bdis4 4200001 4300000 3997
bdis4 4300001 4400000 3924
bdis4 4400001 4500000 4096
bdis4 4500001 4600000 4011
bdis4 4600001 4700000 3423
bdis4 4700001 4800000 3286
bdis4 4800001 4900000 3906
bdis4 4900001 5000000 4177
bdis4 5000001 5100000 3556
bdis4 5100001 5200000 4223
bdis4 5200001 5300000 3918
bdis4 5300001 5400000 4126
bdis4 5400001 5500000 4016
bdis4 5500001 5600000 3721
bdis4 5600001 5700000 4160
bdis4 5700001 5800000 3734
bdis4 5800001 5900000 3630
bdis4 5900001 6000000 4084
bdis4 6000001 6100000 3290
bdis4 6100001 6200000 4087
bdis4 6200001 6300000 3888
bdis4 6300001 6400000 3541
bdis4 6400001 6500000 4301
bdis4 6500001 6600000 4238
bdis4 6600001 6700000 3821
bdis4 6700001 6800000 3528
bdis4 6800001 6900000 3681
bdis4 6900001 7000000 2983
bdis4 7000001 7100000 3756
bdis4 7100001 7200000 2770
bdis4 7200001 7300000 4964
bdis4 7300001 7400000 4010
bdis4 7400001 7500000 3603
bdis4 7500001 7600000 4226
bdis4 7600001 7700000 2991
bdis4 7700001 7800000 3679
bdis4 7800001 7900000 3106
bdis4 7900001 8000000 4301
bdis4 8000001 8100000 3451
bdis4 8100001 8200000 5903
bdis4 8200001 8300000 6282
bdis4 8300001 8400000 5468
bdis4 8400001 8500000 3634

bdis1 67000001 67100000 4703
bdis1 67100001 67200000 3932
bdis1 67200001 67300000 4610
bdis1 67300001 67400000 4928
bdis1 67400001 67500000 3905
bdis1 67500001 67600000 4802
bdis1 67600001 67700000 4166
bdis1 67700001 67800000 5058
bdis1 67800001 67900000 5053
bdis1 67900001 68000000 4868
bdis1 68000001 68100000 3824
bdis1 68100001 68200000 4662
bdis1 68200001 68300000 4865
bdis1 68300001 68400000 4619
bdis1 68400001 68500000 3709
bdis1 68500001 68600000 3796
bdis1 68600001 68700000 3622
bdis1 68700001 68800000 3762
bdis1 68800001 68900000 4252
bdis1 68900001 69000000 4730
bdis1 69000001 69100000 4437
bdis1 69100001 69200000 4844
bdis1 69200001 69300000 4848
bdis1 69300001 69400000 4866
bdis1 69400001 69500000 4364
bdis1 69500001 69600000 4337
bdis1 69600001 69700000 4519
bdis1 69700001 69800000 4732
bdis1 69800001 69900000 5565
bdis1 69900001 70000000 3616
bdis1 70000001 70100000 5662
bdis1 70100001 70200000 4561
bdis1 70200001 70300000 4758
bdis1 70300001 70400000 4951
bdis1 70400001 70500000 4504
bdis1 70500001 70600000 4726
bdis1 70600001 70700000 3877
bdis1 70700001 70800000 4692
bdis1 70800001 70900000 4421
bdis1 70900001 71000000 6208
bdis1 71000001 71100000 5805
bdis1 71100001 71200000 4032
bdis1 71200001 71300000 4533
bdis1 71300001 71400000 4152
bdis1 71400001 71500000 3985
bdis1 71500001 71600000 4335
bdis1 71600001 71700000 4632
bdis1 71700001 71800000 4339
bdis4 8500001 8600000 3569
bdis4 8600001 8700000 3866
bdis4 8700001 8800000 3410
bdis4 8800001 8900000 5008
bdis4 8900001 9000000 3557
bdis4 9000001 9100000 3656
bdis4 9100001 9200000 3871
bdis4 9200001 9300000 3756
bdis4 9300001 9400000 3230
bdis4 9400001 9500000 4216
bdis4 9500001 9600000 3646
bdis4 9600001 9700000 3330
bdis4 9700001 9800000 3509
bdis4 9800001 9900000 3624
bdis4 9900001 10000000 4226
bdis4 10000001 10100000 4119
bdis4 10100001 10200000 4687
bdis4 10200001 10300000 5148
bdis4 10300001 10400000 4368
bdis4 10400001 10500000 4518
bdis4 10500001 10600000 4652
bdis4 10600001 10700000 4680
bdis4 10700001 10800000 4733
bdis4 10800001 10900000 4554
bdis4 10900001 11000000 4395
bdis4 11000001 11100000 4464
bdis4 11100001 11200000 4005
bdis4 11200001 11300000 3457
bdis4 11300001 11400000 3956
bdis4 11400001 11500000 4282
bdis4 11500001 11600000 4013
bdis4 11600001 11700000 5158
bdis4 11700001 11800000 4362
bdis4 11800001 11900000 5219
bdis4 11900001 12000000 3584
bdis4 12000001 12100000 4970
bdis4 12100001 12200000 3791
bdis4 12200001 12300000 4254
bdis4 12300001 12400000 4130
bdis4 12400001 12500000 4888
bdis4 12500001 12600000 3452
bdis4 12600001 12700000 3990
bdis4 12700001 12800000 4962
bdis4 12800001 12900000 3697
bdis4 12900001 13000000 4963
bdis4 13000001 13100000 4397
bdis4 13100001 13200000 3851
bdis4 13200001 13300000 4595

bdis1 71800001 71900000 4302
bdis1 71900001 72000000 3951
bdis1 72000001 72100000 4948
bdis1 72100001 72200000 3932
bdis1 72200001 72300000 4386
bdis1 72300001 72400000 3855
bdis1 72400001 72500000 5237
bdis1 72500001 72600000 5043
bdis1 72600001 72700000 4699
bdis1 72700001 72800000 5456
bdis1 72800001 72900000 5241
bdis1 72900001 73000000 4268
bdis1 73000001 73100000 4980
bdis1 73100001 73200000 3874
bdis1 73200001 73300000 4567
bdis1 73300001 73400000 5366
bdis1 73400001 73500000 4601
bdis1 73500001 73600000 3914
bdis1 73600001 73700000 4009
bdis1 73700001 73800000 4988
bdis1 73800001 73900000 4802
bdis1 73900001 74000000 4569
bdis1 74000001 74100000 4089
bdis1 74100001 74200000 3998
bdis1 74200001 74300000 3856
bdis1 74300001 74400000 4421
bdis1 74400001 74500000 3654
bdis1 74500001 74600000 3657
bdis1 74600001 74700000 3973
bdis1 74700001 74800000 4202
bdis1 74800001 74900000 4202
bdis2 1 100000 4608
bdis2 100001 200000 5257
bdis2 200001 300000 3897
bdis2 300001 400000 3048
bdis2 400001 500000 4453
bdis2 500001 600000 3967
bdis2 600001 700000 4510
bdis2 700001 800000 3926
bdis2 800001 900000 4317
bdis2 900001 1000000 5184
bdis2 1000001 1100000 4143
bdis2 1100001 1200000 4796
bdis2 1200001 1300000 4257
bdis2 1300001 1400000 4816
bdis2 1400001 1500000 5298
bdis2 1500001 1600000 5211
bdis2 1600001 1700000 5708
bdis4 13300001 13400000 3771
bdis4 13400001 13500000 4383
bdis4 13500001 13600000 4100
bdis4 13600001 13700000 4121
bdis4 13700001 13800000 3923
bdis4 13800001 13900000 4237
bdis4 13900001 14000000 4103
bdis4 14000001 14100000 4562
bdis4 14100001 14200000 4035
bdis4 14200001 14300000 4276
bdis4 14300001 14400000 5732
bdis4 14400001 14500000 4066
bdis4 14500001 14600000 4199
bdis4 14600001 14700000 4530
bdis4 14700001 14800000 3876
bdis4 14800001 14900000 4445
bdis4 14900001 15000000 4064
bdis4 15000001 15100000 4441
bdis4 15100001 15200000 5140
bdis4 15200001 15300000 4575
bdis4 15300001 15400000 4626
bdis4 15400001 15500000 4852
bdis4 15500001 15600000 4020
bdis4 15600001 15700000 4073
bdis4 15700001 15800000 3830
bdis4 15800001 15900000 4312
bdis4 15900001 16000000 5848
bdis4 16000001 16100000 4783
bdis4 16100001 16200000 4562
bdis4 16200001 16300000 5663
bdis4 16300001 16400000 4206
bdis4 16400001 16500000 6071
bdis4 16500001 16600000 5842
bdis4 16600001 16700000 4106
bdis4 16700001 16800000 4924
bdis4 16800001 16900000 4176
bdis4 16900001 17000000 4393
bdis4 17000001 17100000 4002
bdis4 17100001 17200000 4719
bdis4 17200001 17300000 4404
bdis4 17300001 17400000 4148
bdis4 17400001 17500000 4051
bdis4 17500001 17600000 4162
bdis4 17600001 17700000 3254
bdis4 17700001 17800000 4793
bdis4 17800001 17900000 5383
bdis4 17900001 18000000 4297
bdis4 18000001 18100000 4535

bdis2 1700001 1800000 4842
bdis2 1800001 1900000 4272
bdis2 1900001 2000000 3649
bdis2 2000001 2100000 3328
bdis2 2100001 2200000 4139
bdis2 2200001 2300000 3631
bdis2 2300001 2400000 5642
bdis2 2400001 2500000 3815
bdis2 2500001 2600000 3950
bdis2 2600001 2700000 3880
bdis2 2700001 2800000 3926
bdis2 2800001 2900000 4081
bdis2 2900001 3000000 4437
bdis2 3000001 3100000 4299
bdis2 3100001 3200000 4615
bdis2 3200001 3300000 4240
bdis2 3300001 3400000 4050
bdis2 3400001 3500000 4160
bdis2 3500001 3600000 4269
bdis2 3600001 3700000 4555
bdis2 3700001 3800000 4209
bdis2 3800001 3900000 4128
bdis2 3900001 4000000 3682
bdis2 4000001 4100000 4400
bdis2 4100001 4200000 4927
bdis2 4200001 4300000 4782
bdis2 4300001 4400000 4341
bdis2 4400001 4500000 3985
bdis2 4500001 4600000 4708
bdis2 4600001 4700000 4558
bdis2 4700001 4800000 4144
bdis2 4800001 4900000 3986
bdis2 4900001 5000000 4324
bdis2 5000001 5100000 4533
bdis2 5100001 5200000 4592
bdis2 5200001 5300000 5090
bdis2 5300001 5400000 4717
bdis2 5400001 5500000 4019
bdis2 5500001 5600000 4826
bdis2 5600001 5700000 4569
bdis2 5700001 5800000 4651
bdis2 5800001 5900000 4666
bdis2 5900001 6000000 5119
bdis2 6000001 6100000 4213
bdis2 6100001 6200000 4614
bdis2 6200001 6300000 4680
bdis2 6300001 6400000 4337
bdis2 6400001 6500000 3659
bdis4 18100001 18200000 3644
bdis4 18200001 18300000 5155
bdis4 18300001 18400000 4811
bdis4 18400001 18500000 3644
bdis4 18500001 18600000 3979
bdis4 18600001 18700000 4787
bdis4 18700001 18800000 4614
bdis4 18800001 18900000 5754
bdis4 18900001 19000000 5763
bdis4 19000001 19100000 6518
bdis4 19100001 19200000 5883
bdis4 19200001 19300000 7951
bdis4 19300001 19400000 6348
bdis4 19400001 19500000 6466
bdis4 19500001 19600000 4631
bdis4 19600001 19700000 4447
bdis4 19700001 19800000 5204
bdis4 19800001 19900000 6830
bdis4 19900001 20000000 7489
bdis4 20000001 20100000 4301
bdis4 20100001 20200000 5815
bdis4 20200001 20300000 4589
bdis4 20300001 20400000 5614
bdis4 20400001 20500000 4319
bdis4 20500001 20600000 6108
bdis4 20600001 20700000 4187
bdis4 20700001 20800000 4926
bdis4 20800001 20900000 5124
bdis4 20900001 21000000 4783
bdis4 21000001 21100000 5342
bdis4 21100001 21200000 5133
bdis4 21200001 21300000 3837
bdis4 21300001 21400000 6661
bdis4 21400001 21500000 5441
bdis4 21500001 21600000 4466
bdis4 21600001 21700000 4085
bdis4 21700001 21800000 3870
bdis4 21800001 21900000 4590
bdis4 21900001 22000000 5865
bdis4 22000001 22100000 5461
bdis4 22100001 22200000 3720
bdis4 22200001 22300000 5096
bdis4 22300001 22400000 6017
bdis4 22400001 22500000 4561
bdis4 22500001 22600000 4567
bdis4 22600001 22700000 4464
bdis4 22700001 22800000 4900
bdis4 22800001 22900000 5006

bdis2 6500001 6600000 4963
bdis2 6600001 6700000 5503
bdis2 6700001 6800000 4853
bdis2 6800001 6900000 5386
bdis2 6900001 7000000 4470
bdis2 7000001 7100000 4492
bdis2 7100001 7200000 4609
bdis2 7200001 7300000 4622
bdis2 7300001 7400000 4333
bdis2 7400001 7500000 3680
bdis2 7500001 7600000 4052
bdis2 7600001 7700000 3783
bdis2 7700001 7800000 3886
bdis2 7800001 7900000 3916
bdis2 7900001 8000000 3346
bdis2 8000001 8100000 4023
bdis2 8100001 8200000 4732
bdis2 8200001 8300000 3420
bdis2 8300001 8400000 5225
bdis2 8400001 8500000 3927
bdis2 8500001 8600000 4756
bdis2 8600001 8700000 3904
bdis2 8700001 8800000 4108
bdis2 8800001 8900000 4047
bdis2 8900001 9000000 3901
bdis2 9000001 9100000 3684
bdis2 9100001 9200000 4701
bdis2 9200001 9300000 4174
bdis2 9300001 9400000 5209
bdis2 9400001 9500000 4036
bdis2 9500001 9600000 4871
bdis2 9600001 9700000 4452
bdis2 9700001 9800000 3898
bdis2 9800001 9900000 4317
bdis2 9900001 10000000 5100
bdis2 10000001 10100000 4491
bdis2 10100001 10200000 4082
bdis2 10200001 10300000 5229
bdis2 10300001 10400000 4422
bdis2 10400001 10500000 3405
bdis2 10500001 10600000 4890
bdis2 10600001 10700000 4036
bdis2 10700001 10800000 4924
bdis2 10800001 10900000 4004
bdis2 10900001 11000000 3860
bdis2 11000001 11100000 3747
bdis2 11100001 11200000 3808
bdis2 11200001 11300000 3926
bdis4 22900001 23000000 4038
bdis4 23000001 23100000 5038
bdis4 23100001 23200000 4103
bdis4 23200001 23300000 4866
bdis4 23300001 23400000 4667
bdis4 23400001 23500000 3913
bdis4 23500001 23600000 3433
bdis4 23600001 23700000 4687
bdis4 23700001 23800000 4699
bdis4 23800001 23900000 4098
bdis4 23900001 24000000 3721
bdis4 24000001 24100000 4550
bdis4 24100001 24200000 4454
bdis4 24200001 24300000 3945
bdis4 24300001 24400000 4866
bdis4 24400001 24500000 3954
bdis4 24500001 24600000 4467
bdis4 24600001 24700000 4986
bdis4 24700001 24800000 4795
bdis4 24800001 24900000 6239
bdis4 24900001 25000000 4278
bdis4 25000001 25100000 4528
bdis4 25100001 25200000 4306
bdis4 25200001 25300000 5577
bdis4 25300001 25400000 5002
bdis4 25400001 25500000 4388
bdis4 25500001 25600000 5280
bdis4 25600001 25700000 6226
bdis4 25700001 25800000 4112
bdis4 25800001 25900000 4249
bdis4 25900001 26000000 4331
bdis4 26000001 26100000 4280
bdis4 26100001 26200000 4626
bdis4 26200001 26300000 4988
bdis4 26300001 26400000 5516
bdis4 26400001 26500000 4632
bdis4 26500001 26600000 3989
bdis4 26600001 26700000 5290
bdis4 26700001 26800000 4770
bdis4 26800001 26900000 5623
bdis4 26900001 27000000 5027
bdis4 27000001 27100000 3821
bdis4 27100001 27200000 4996
bdis4 27200001 27300000 4036
bdis4 27300001 27400000 4337
bdis4 27400001 27500000 5078
bdis4 27500001 27600000 3870
bdis4 27600001 27700000 5132

bdis2 11300001 11400000 3849
bdis2 11400001 11500000 4087
bdis2 11500001 11600000 4343
bdis2 11600001 11700000 3373
bdis2 11700001 11800000 3972
bdis2 11800001 11900000 3064
bdis2 11900001 12000000 3576
bdis2 12000001 12100000 3295
bdis2 12100001 12200000 5446
bdis2 12200001 12300000 4233
bdis2 12300001 12400000 5346
bdis2 12400001 12500000 3757
bdis2 12500001 12600000 4364
bdis2 12600001 12700000 6581
bdis2 12700001 12800000 4518
bdis2 12800001 12900000 3891
bdis2 12900001 13000000 3607
bdis2 13000001 13100000 4372
bdis2 13100001 13200000 3601
bdis2 13200001 13300000 4680
bdis2 13300001 13400000 5276
bdis2 13400001 13500000 4254
bdis2 13500001 13600000 5045
bdis2 13600001 13700000 4573
bdis2 13700001 13800000 4909
bdis2 13800001 13900000 4515
bdis2 13900001 14000000 4645
bdis2 14000001 14100000 4475
bdis2 14100001 14200000 4711
bdis2 14200001 14300000 4400
bdis2 14300001 14400000 5221
bdis2 14400001 14500000 4783
bdis2 14500001 14600000 4388
bdis2 14600001 14700000 4335
bdis2 14700001 14800000 4686
bdis2 14800001 14900000 5645
bdis2 14900001 15000000 4185
bdis2 15000001 15100000 5257
bdis2 15100001 15200000 6091
bdis2 15200001 15300000 4578
bdis2 15300001 15400000 4576
bdis2 15400001 15500000 4906
bdis2 15500001 15600000 4437
bdis2 15600001 15700000 5120
bdis2 15700001 15800000 4721
bdis2 15800001 15900000 4678
bdis2 15900001 16000000 3417
bdis2 16000001 16100000 5060
bdis4 27700001 27800000 4424
bdis4 27800001 27900000 4149
bdis4 27900001 28000000 4960
bdis4 28000001 28100000 4208
bdis4 28100001 28200000 4076
bdis4 28200001 28300000 4123
bdis4 28300001 28400000 4283
bdis4 28400001 28500000 4776
bdis4 28500001 28600000 4734
bdis4 28600001 28700000 4747
bdis4 28700001 28800000 3597
bdis4 28800001 28900000 5145
bdis4 28900001 29000000 4094
bdis4 29000001 29100000 3548
bdis4 29100001 29200000 4731
bdis4 29200001 29300000 4570
bdis4 29300001 29400000 4110
bdis4 29400001 29500000 3776
bdis4 29500001 29600000 3345
bdis4 29600001 29700000 4911
bdis4 29700001 29800000 4086
bdis4 29800001 29900000 4339
bdis4 29900001 30000000 4549
bdis4 30000001 30100000 3561
bdis4 30100001 30200000 4406
bdis4 30200001 30300000 4159
bdis4 30300001 30400000 4899
bdis4 30400001 30500000 4491
bdis4 30500001 30600000 5094
bdis4 30600001 30700000 5302
bdis4 30700001 30800000 4481
bdis4 30800001 30900000 5833
bdis4 30900001 31000000 3796
bdis4 31000001 31100000 3617
bdis4 31100001 31200000 3447
bdis4 31200001 31300000 3508
bdis4 31300001 31400000 4645
bdis4 31400001 31500000 4382
bdis4 31500001 31600000 3847
bdis4 31600001 31700000 4551
bdis4 31700001 31800000 3900
bdis4 31800001 31900000 3878
bdis4 31900001 32000000 4418
bdis4 32000001 32100000 3836
bdis4 32100001 32200000 4078
bdis4 32200001 32300000 4023
bdis4 32300001 32400000 5302
bdis4 32400001 32500000 3512

bdis2 16100001 16200000 3630
bdis2 16200001 16300000 5628
bdis2 16300001 16400000 4240
bdis2 16400001 16500000 4780
bdis2 16500001 16600000 4291
bdis2 16600001 16700000 4064
bdis2 16700001 16800000 4794
bdis2 16800001 16900000 4026
bdis2 16900001 17000000 5132
bdis2 17000001 17100000 4958
bdis2 17100001 17200000 4061
bdis2 17200001 17300000 4338
bdis2 17300001 17400000 4671
bdis2 17400001 17500000 4412
bdis2 17500001 17600000 4732
bdis2 17600001 17700000 4681
bdis2 17700001 17800000 3224
bdis2 17800001 17900000 5380
bdis2 17900001 18000000 4888
bdis2 18000001 18100000 3858
bdis2 18100001 18200000 5423
bdis2 18200001 18300000 5618
bdis2 18300001 18400000 4507
bdis2 18400001 18500000 5110
bdis2 18500001 18600000 5394
bdis2 18600001 18700000 4851
bdis2 18700001 18800000 4838
bdis2 18800001 18900000 4911
bdis2 18900001 19000000 5139
bdis2 19000001 19100000 7054
bdis2 19100001 19200000 3978
bdis2 19200001 19300000 5089
bdis2 19300001 19400000 4633
bdis2 19400001 19500000 5323
bdis2 19500001 19600000 5366
bdis2 19600001 19700000 4811
bdis2 19700001 19800000 4585
bdis2 19800001 19900000 5627
bdis2 19900001 20000000 4781
bdis2 20000001 20100000 4852
bdis2 20100001 20200000 5253
bdis2 20200001 20300000 4707
bdis2 20300001 20400000 4264
bdis2 20400001 20500000 5610
bdis2 20500001 20600000 4315
bdis2 20600001 20700000 5406
bdis2 20700001 20800000 4512
bdis2 20800001 20900000 3734
bdis4 32500001 32600000 3531
bdis4 32600001 32700000 3809
bdis4 32700001 32800000 3940
bdis4 32800001 32900000 4353
bdis4 32900001 33000000 3771
bdis4 33000001 33100000 4240
bdis4 33100001 33200000 4780
bdis4 33200001 33300000 3837
bdis4 33300001 33400000 3834
bdis4 33400001 33500000 3934
bdis4 33500001 33600000 5039
bdis4 33600001 33700000 4541
bdis4 33700001 33800000 4063
bdis4 33800001 33900000 4274
bdis4 33900001 34000000 4148
bdis4 34000001 34100000 3784
bdis4 34100001 34200000 4408
bdis4 34200001 34300000 3741
bdis4 34300001 34400000 4089
bdis4 34400001 34500000 4886
bdis4 34500001 34600000 5339
bdis4 34600001 34700000 4069
bdis4 34700001 34800000 4350
bdis4 34800001 34900000 5502
bdis4 34900001 35000000 4517
bdis4 35000001 35100000 4777
bdis4 35100001 35200000 4984
bdis4 35200001 35300000 4359
bdis4 35300001 35400000 6176
bdis4 35400001 35500000 4301
bdis4 35500001 35600000 3582
bdis4 35600001 35700000 4445
bdis4 35700001 35800000 4722
bdis4 35800001 35900000 4277
bdis4 35900001 36000000 4302
bdis4 36000001 36100000 5052
bdis4 36100001 36200000 4697
bdis4 36200001 36300000 4401
bdis4 36300001 36400000 4920
bdis4 36400001 36500000 5476
bdis4 36500001 36600000 4989
bdis4 36600001 36700000 5090
bdis4 36700001 36800000 4926
bdis4 36800001 36900000 4143
bdis4 36900001 37000000 4635
bdis4 37000001 37100000 3791
bdis4 37100001 37200000 5897
bdis4 37200001 37300000 3958

bdis2 20900001 21000000 4479
bdis2 21000001 21100000 4068
bdis2 21100001 21200000 5041
bdis2 21200001 21300000 4226
bdis2 21300001 21400000 6080
bdis2 21400001 21500000 4427
bdis2 21500001 21600000 5033
bdis2 21600001 21700000 4097
bdis2 21700001 21800000 5203
bdis2 21800001 21900000 5649
bdis2 21900001 22000000 6098
bdis2 22000001 22100000 4318
bdis2 22100001 22200000 3977
bdis2 22200001 22300000 5134
bdis2 22300001 22400000 5864
bdis2 22400001 22500000 5566
bdis2 22500001 22600000 4467
bdis2 22600001 22700000 5102
bdis2 22700001 22800000 4595
bdis2 22800001 22900000 4163
bdis2 22900001 23000000 5552
bdis2 23000001 23100000 4548
bdis2 23100001 23200000 5246
bdis2 23200001 23300000 4397
bdis2 23300001 23400000 5392
bdis2 23400001 23500000 4416
bdis2 23500001 23600000 3758
bdis2 23600001 23700000 3940
bdis2 23700001 23800000 4171
bdis2 23800001 23900000 5504
bdis2 23900001 24000000 4876
bdis2 24000001 24100000 4403
bdis2 24100001 24200000 5245
bdis2 24200001 24300000 4442
bdis2 24300001 24400000 4948
bdis2 24400001 24500000 5120
bdis2 24500001 24600000 4786
bdis2 24600001 24700000 4591
bdis2 24700001 24800000 4363
bdis2 24800001 24900000 4583
bdis2 24900001 25000000 5722
bdis2 25000001 25100000 7010
bdis2 25100001 25200000 4459
bdis2 25200001 25300000 4771
bdis2 25300001 25400000 4729
bdis2 25400001 25500000 4201
bdis2 25500001 25600000 4427
bdis2 25600001 25700000 3698
bdis4 37300001 37400000 5768
bdis4 37400001 37500000 5049
bdis4 37500001 37600000 5276
bdis4 37600001 37700000 3964
bdis4 37700001 37800000 5037
bdis4 37800001 37900000 5742
bdis4 37900001 38000000 4041
bdis4 38000001 38100000 5316
bdis4 38100001 38200000 4987
bdis4 38200001 38300000 6037
bdis4 38300001 38400000 5068
bdis4 38400001 38500000 5504
bdis4 38500001 38600000 5395
bdis4 38600001 38700000 4586
bdis4 38700001 38800000 4807
bdis4 38800001 38900000 3991
bdis4 38900001 39000000 4810
bdis4 39000001 39100000 4392
bdis4 39100001 39200000 5278
bdis4 39200001 39300000 5773
bdis4 39300001 39400000 4636
bdis4 39400001 39500000 4946
bdis4 39500001 39600000 4319
bdis4 39600001 39700000 3835
bdis4 39700001 39800000 3794
bdis4 39800001 39900000 4274
bdis4 39900001 40000000 4586
bdis4 40000001 40100000 4858
bdis4 40100001 40200000 4596
bdis4 40200001 40300000 4554
bdis4 40300001 40400000 3811
bdis4 40400001 40500000 4966
bdis4 40500001 40600000 3313
bdis4 40600001 40700000 4007
bdis4 40700001 40800000 4670
bdis4 40800001 40900000 4512
bdis4 40900001 41000000 5537
bdis4 41000001 41100000 4517
bdis4 41100001 41200000 4958
bdis4 41200001 41300000 4667
bdis4 41300001 41400000 4608
bdis4 41400001 41500000 3229
bdis4 41500001 41600000 4381
bdis4 41600001 41700000 5268
bdis4 41700001 41800000 4637
bdis4 41800001 41900000 5168
bdis4 41900001 42000000 5367
bdis4 42000001 42100000 4512

bdis2 25700001 25800000 4109
bdis2 25800001 25900000 4647
bdis2 25900001 26000000 4720
bdis2 26000001 26100000 4049
bdis2 26100001 26200000 5873
bdis2 26200001 26300000 4248
bdis2 26300001 26400000 5476
bdis2 26400001 26500000 5772
bdis2 26500001 26600000 4585
bdis2 26600001 26700000 4048
bdis2 26700001 26800000 4828
bdis2 26800001 26900000 5677
bdis2 26900001 27000000 5026
bdis2 27000001 27100000 5339
bdis2 27100001 27200000 4079
bdis2 27200001 27300000 3805
bdis2 27300001 27400000 4886
bdis2 27400001 27500000 4805
bdis2 27500001 27600000 5780
bdis2 27600001 27700000 3387
bdis2 27700001 27800000 4391
bdis2 27800001 27900000 6971
bdis2 27900001 28000000 4453
bdis2 28000001 28100000 7252
bdis2 28100001 28200000 5501
bdis2 28200001 28300000 5504
bdis2 28300001 28400000 4904
bdis2 28400001 28500000 5881
bdis2 28500001 28600000 6363
bdis2 28600001 28700000 5366
bdis2 28700001 28800000 5908
bdis2 28800001 28900000 5425
bdis2 28900001 29000000 1233
bdis2 29000001 29100000 4978
bdis2 29100001 29200000 3909
bdis2 29200001 29300000 4849
bdis2 29300001 29400000 5369
bdis2 29400001 29500000 4710
bdis2 29500001 29600000 5537
bdis2 29600001 29700000 4924
bdis2 29700001 29800000 5729
bdis2 29800001 29900000 7946
bdis2 29900001 30000000 4669
bdis2 30000001 30100000 6396
bdis2 30100001 30200000 3839
bdis2 30200001 30300000 5376
bdis2 30300001 30400000 6201
bdis2 30400001 30500000 4896
bdis4 42100001 42200000 4017
bdis4 42200001 42300000 5182
bdis4 42300001 42400000 4194
bdis4 42400001 42500000 4799
bdis4 42500001 42600000 5307
bdis4 42600001 42700000 4165
bdis4 42700001 42800000 4272
bdis4 42800001 42900000 4271
bdis4 42900001 43000000 3882
bdis4 43000001 43100000 3865
bdis4 43100001 43200000 3783
bdis4 43200001 43300000 4279
bdis4 43300001 43400000 3677
bdis4 43400001 43500000 4559
bdis4 43500001 43600000 3652
bdis4 43600001 43700000 4649
bdis4 43700001 43800000 3108
bdis4 43800001 43900000 4309
bdis4 43900001 44000000 3847
bdis4 44000001 44100000 4094
bdis4 44100001 44200000 3754
bdis4 44200001 44300000 4615
bdis4 44300001 44400000 4365
bdis4 44400001 44500000 4625
bdis4 44500001 44600000 3631
bdis4 44600001 44700000 3551
bdis4 44700001 44800000 3865
bdis4 44800001 44900000 3772
bdis4 44900001 45000000 3380
bdis4 45000001 45100000 3812
bdis4 45100001 45200000 4864
bdis4 45200001 45300000 4169
bdis4 45300001 45400000 4115
bdis4 45400001 45500000 4270
bdis4 45500001 45600000 4288
bdis4 45600001 45700000 4512
bdis4 45700001 45800000 4172
bdis4 45800001 45900000 3811
bdis4 45900001 46000000 4653
bdis4 46000001 46100000 3996
bdis4 46100001 46200000 4750
bdis4 46200001 46300000 4455
bdis4 46300001 46400000 4458
bdis4 46400001 46500000 4982
bdis4 46500001 46600000 5457
bdis4 46600001 46700000 4381
bdis4 46700001 46800000 4418
bdis4 46800001 46900000 4618

bdis2 30500001 30600000 5949
bdis2 30600001 30700000 6369
bdis2 30700001 30800000 5454
bdis2 30800001 30900000 4036
bdis2 30900001 31000000 5419
bdis2 31000001 31100000 3854
bdis2 31100001 31200000 6454
bdis2 31200001 31300000 5344
bdis2 31300001 31400000 4114
bdis2 31400001 31500000 5911
bdis2 31500001 31600000 4275
bdis2 31600001 31700000 3974
bdis2 31700001 31800000 4125
bdis2 31800001 31900000 4469
bdis2 31900001 32000000 4898
bdis2 32000001 32100000 4926
bdis2 32100001 32200000 5481
bdis2 32200001 32300000 4481
bdis2 32300001 32400000 5282
bdis2 32400001 32500000 4139
bdis2 32500001 32600000 4431
bdis2 32600001 32700000 6706
bdis2 32700001 32800000 5713
bdis2 32800001 32900000 5659
bdis2 32900001 33000000 6084
bdis2 33000001 33100000 3954
bdis2 33100001 33200000 4093
bdis2 33200001 33300000 4278
bdis2 33300001 33400000 4600
bdis2 33400001 33500000 4630
bdis2 33500001 33600000 4004
bdis2 33600001 33700000 4078
bdis2 33700001 33800000 4557
bdis2 33800001 33900000 4916
bdis2 33900001 34000000 5333
bdis2 34000001 34100000 5757
bdis2 34100001 34200000 4491
bdis2 34200001 34300000 4112
bdis2 34300001 34400000 4204
bdis2 34400001 34500000 4198
bdis2 34500001 34600000 3764
bdis2 34600001 34700000 3811
bdis2 34700001 34800000 4435
bdis2 34800001 34900000 3366
bdis2 34900001 35000000 3435
bdis2 35000001 35100000 3475
bdis2 35100001 35200000 3587
bdis2 35200001 35300000 3350
bdis4 46900001 47000000 5032
bdis4 47000001 47100000 4734
bdis4 47100001 47200000 3851
bdis4 47200001 47300000 3876
bdis4 47300001 47400000 3507
bdis4 47400001 47500000 4023
bdis4 47500001 47600000 3913
bdis4 47600001 47700000 4285
bdis4 47700001 47800000 4156
bdis4 47800001 47900000 4301
bdis4 47900001 48000000 4204
bdis4 48000001 48100000 4785
bdis4 48100001 48200000 3430
bdis4 48200001 48300000 3370
bdis4 48300001 48400000 3670
bdis4 48400001 48500000 3448
bdis4 48500001 48600000 5001
bdis4 48600001 48700000 5001
bdis5 1 100000 9158
bdis5 100001 200000 5220
bdis5 200001 300000 5208
bdis5 300001 400000 3349
bdis5 400001 500000 3333
bdis5 500001 600000 3820
bdis5 600001 700000 4016
bdis5 700001 800000 4507
bdis5 800001 900000 3720
bdis5 900001 1000000 3869
bdis5 1000001 1100000 3612
bdis5 1100001 1200000 3804
bdis5 1200001 1300000 5001
bdis5 1300001 1400000 3983
bdis5 1400001 1500000 5815
bdis5 1500001 1600000 5571
bdis5 1600001 1700000 4250
bdis5 1700001 1800000 3744
bdis5 1800001 1900000 4626
bdis5 1900001 2000000 3575
bdis5 2000001 2100000 4033
bdis5 2100001 2200000 4418
bdis5 2200001 2300000 4722
bdis5 2300001 2400000 3372
bdis5 2400001 2500000 3156
bdis5 2500001 2600000 4330
bdis5 2600001 2700000 4582
bdis5 2700001 2800000 4585
bdis5 2800001 2900000 4598
bdis5 2900001 3000000 3811

bdis2 35300001 35400000 4614
bdis2 35400001 35500000 4779
bdis2 35500001 35600000 3980
bdis2 35600001 35700000 4289
bdis2 35700001 35800000 4888
bdis2 35800001 35900000 4328
bdis2 35900001 36000000 4375
bdis2 36000001 36100000 4526
bdis2 36100001 36200000 3954
bdis2 36200001 36300000 4121
bdis2 36300001 36400000 4028
bdis2 36400001 36500000 4030
bdis2 36500001 36600000 4668
bdis2 36600001 36700000 3701
bdis2 36700001 36800000 3181
bdis2 36800001 36900000 4657
bdis2 36900001 37000000 4409
bdis2 37000001 37100000 4534
bdis2 37100001 37200000 4455
bdis2 37200001 37300000 4272
bdis2 37300001 37400000 4231
bdis2 37400001 37500000 4863
bdis2 37500001 37600000 4296
bdis2 37600001 37700000 4251
bdis2 37700001 37800000 3927
bdis2 37800001 37900000 4410
bdis2 37900001 38000000 4403
bdis2 38000001 38100000 3665
bdis2 38100001 38200000 3194
bdis2 38200001 38300000 4870
bdis2 38300001 38400000 4894
bdis2 38400001 38500000 5419
bdis2 38500001 38600000 4832
bdis2 38600001 38700000 4467
bdis2 38700001 38800000 3148
bdis2 38800001 38900000 3444
bdis2 38900001 39000000 4498
bdis2 39000001 39100000 4113
bdis2 39100001 39200000 3871
bdis2 39200001 39300000 3052
bdis2 39300001 39400000 4044
bdis2 39400001 39500000 3548
bdis2 39500001 39600000 4597
bdis2 39600001 39700000 4612
bdis2 39700001 39800000 3548
bdis2 39800001 39900000 3196
bdis2 39900001 40000000 3404
bdis2 40000001 40100000 4321
bdis5 3000001 3100000 3468
bdis5 3100001 3200000 4184
bdis5 3200001 3300000 4595
bdis5 3300001 3400000 4651
bdis5 3400001 3500000 4782
bdis5 3500001 3600000 5697
bdis5 3600001 3700000 3586
bdis5 3700001 3800000 4995
bdis5 3800001 3900000 5184
bdis5 3900001 4000000 4405
bdis5 4000001 4100000 5069
bdis5 4100001 4200000 5994
bdis5 4200001 4300000 3444
bdis5 4300001 4400000 3822
bdis5 4400001 4500000 4321
bdis5 4500001 4600000 5266
bdis5 4600001 4700000 4794
bdis5 4700001 4800000 4327
bdis5 4800001 4900000 5663
bdis5 4900001 5000000 4984
bdis5 5000001 5100000 4169
bdis5 5100001 5200000 5038
bdis5 5200001 5300000 5531
bdis5 5300001 5400000 5482
bdis5 5400001 5500000 6772
bdis5 5500001 5600000 5130
bdis5 5600001 5700000 4459
bdis5 5700001 5800000 5545
bdis5 5800001 5900000 4348
bdis5 5900001 6000000 4705
bdis5 6000001 6100000 4840
bdis5 6100001 6200000 3886
bdis5 6200001 6300000 5985
bdis5 6300001 6400000 4518
bdis5 6400001 6500000 4721
bdis5 6500001 6600000 3418
bdis5 6600001 6700000 5377
bdis5 6700001 6800000 4031
bdis5 6800001 6900000 6112
bdis5 6900001 7000000 6676
bdis5 7000001 7100000 4998
bdis5 7100001 7200000 4976
bdis5 7200001 7300000 5432
bdis5 7300001 7400000 4659
bdis5 7400001 7500000 5930
bdis5 7500001 7600000 3488
bdis5 7600001 7700000 5265
bdis5 7700001 7800000 5216

bdis2 40100001 40200000 5029
bdis2 40200001 40300000 4657
bdis2 40300001 40400000 5887
bdis2 40400001 40500000 4440
bdis2 40500001 40600000 3790
bdis2 40600001 40700000 4022
bdis2 40700001 40800000 4160
bdis2 40800001 40900000 5168
bdis2 40900001 41000000 3935
bdis2 41000001 41100000 4085
bdis2 41100001 41200000 3816
bdis2 41200001 41300000 3887
bdis2 41300001 41400000 4098
bdis2 41400001 41500000 3492
bdis2 41500001 41600000 4055
bdis2 41600001 41700000 4222
bdis2 41700001 41800000 3457
bdis2 41800001 41900000 4597
bdis2 41900001 42000000 6281
bdis2 42000001 42100000 3972
bdis2 42100001 42200000 3953
bdis2 42200001 42300000 3640
bdis2 42300001 42400000 4796
bdis2 42400001 42500000 4031
bdis2 42500001 42600000 3196
bdis2 42600001 42700000 4848
bdis2 42700001 42800000 4335
bdis2 42800001 42900000 3477
bdis2 42900001 43000000 3189
bdis2 43000001 43100000 4678
bdis2 43100001 43200000 4826
bdis2 43200001 43300000 3930
bdis2 43300001 43400000 5211
bdis2 43400001 43500000 3943
bdis2 43500001 43600000 4153
bdis2 43600001 43700000 4227
bdis2 43700001 43800000 4530
bdis2 43800001 43900000 4734
bdis2 43900001 44000000 4667
bdis2 44000001 44100000 3492
bdis2 44100001 44200000 3591
bdis2 44200001 44300000 4566
bdis2 44300001 44400000 3913
bdis2 44400001 44500000 4445
bdis2 44500001 44600000 3891
bdis2 44600001 44700000 4164
bdis2 44700001 44800000 4262
bdis2 44800001 44900000 4844
bdis5 7800001 7900000 4565
bdis5 7900001 8000000 4373
bdis5 8000001 8100000 5390
bdis5 8100001 8200000 6496
bdis5 8200001 8300000 4372
bdis5 8300001 8400000 5611
bdis5 8400001 8500000 5015
bdis5 8500001 8600000 4851
bdis5 8600001 8700000 5395
bdis5 8700001 8800000 6003
bdis5 8800001 8900000 5251
bdis5 8900001 9000000 6022
bdis5 9000001 9100000 5250
bdis5 9100001 9200000 5218
bdis5 9200001 9300000 5484
bdis5 9300001 9400000 6016
bdis5 9400001 9500000 4536
bdis5 9500001 9600000 3820
bdis5 9600001 9700000 3412
bdis5 9700001 9800000 3755
bdis5 9800001 9900000 5673
bdis5 9900001 10000000 5326
bdis5 10000001 10100000 4426
bdis5 10100001 10200000 4832
bdis5 10200001 10300000 4707
bdis5 10300001 10400000 4799
bdis5 10400001 10500000 4181
bdis5 10500001 10600000 5186
bdis5 10600001 10700000 3723
bdis5 10700001 10800000 4430
bdis5 10800001 10900000 4308
bdis5 10900001 11000000 4308
bdis5 11000001 11100000 4227
bdis5 11100001 11200000 2806
bdis5 11200001 11300000 5162
bdis5 11300001 11400000 4732
bdis5 11400001 11500000 4933
bdis5 11500001 11600000 4869
bdis5 11600001 11700000 4991
bdis5 11700001 11800000 4599
bdis5 11800001 11900000 4669
bdis5 11900001 12000000 4514
bdis5 12000001 12100000 5088
bdis5 12100001 12200000 4883
bdis5 12200001 12300000 4707
bdis5 12300001 12400000 4119
bdis5 12400001 12500000 6023
bdis5 12500001 12600000 4809

bdis2 44900001 45000000 3756
bdis2 45000001 45100000 4850
bdis2 45100001 45200000 5579
bdis2 45200001 45300000 4625
bdis2 45300001 45400000 3953
bdis2 45400001 45500000 4763
bdis2 45500001 45600000 4250
bdis2 45600001 45700000 5313
bdis2 45700001 45800000 4117
bdis2 45800001 45900000 4458
bdis2 45900001 46000000 4238
bdis2 46000001 46100000 5375
bdis2 46100001 46200000 5056
bdis2 46200001 46300000 3795
bdis2 46300001 46400000 4174
bdis2 46400001 46500000 4393
bdis2 46500001 46600000 3845
bdis2 46600001 46700000 3627
bdis2 46700001 46800000 5637
bdis2 46800001 46900000 4024
bdis2 46900001 47000000 3969
bdis2 47000001 47100000 4108
bdis2 47100001 47200000 4414
bdis2 47200001 47300000 3729
bdis2 47300001 47400000 4504
bdis2 47400001 47500000 4594
bdis2 47500001 47600000 4616
bdis2 47600001 47700000 3381
bdis2 47700001 47800000 4913
bdis2 47800001 47900000 6352
bdis2 47900001 48000000 5035
bdis2 48000001 48100000 4390
bdis2 48100001 48200000 4911
bdis2 48200001 48300000 3739
bdis2 48300001 48400000 4639
bdis2 48400001 48500000 3280
bdis2 48500001 48600000 5542
bdis2 48600001 48700000 4580
bdis2 48700001 48800000 4500
bdis2 48800001 48900000 4126
bdis2 48900001 49000000 4854
bdis2 49000001 49100000 4189
bdis2 49100001 49200000 4377
bdis2 49200001 49300000 5157
bdis2 49300001 49400000 4103
bdis2 49400001 49500000 5692
bdis2 49500001 49600000 4803
bdis2 49600001 49700000 3443
bdis5 12600001 12700000 4286
bdis5 12700001 12800000 4741
bdis5 12800001 12900000 4440
bdis5 12900001 13000000 4863
bdis5 13000001 13100000 3942
bdis5 13100001 13200000 4259
bdis5 13200001 13300000 4125
bdis5 13300001 13400000 4770
bdis5 13400001 13500000 3793
bdis5 13500001 13600000 4845
bdis5 13600001 13700000 3907
bdis5 13700001 13800000 4484
bdis5 13800001 13900000 5252
bdis5 13900001 14000000 4370
bdis5 14000001 14100000 4195
bdis5 14100001 14200000 5094
bdis5 14200001 14300000 4476
bdis5 14300001 14400000 5392
bdis5 14400001 14500000 5241
bdis5 14500001 14600000 3579
bdis5 14600001 14700000 5029
bdis5 14700001 14800000 4121
bdis5 14800001 14900000 4981
bdis5 14900001 15000000 6374
bdis5 15000001 15100000 5519
bdis5 15100001 15200000 4992
bdis5 15200001 15300000 4863
bdis5 15300001 15400000 4552
bdis5 15400001 15500000 6012
bdis5 15500001 15600000 5061
bdis5 15600001 15700000 4911
bdis5 15700001 15800000 3997
bdis5 15800001 15900000 4764
bdis5 15900001 16000000 5160
bdis5 16000001 16100000 5395
bdis5 16100001 16200000 4680
bdis5 16200001 16300000 4922
bdis5 16300001 16400000 3933
bdis5 16400001 16500000 4692
bdis5 16500001 16600000 4904
bdis5 16600001 16700000 5311
bdis5 16700001 16800000 4828
bdis5 16800001 16900000 4911
bdis5 16900001 17000000 4375
bdis5 17000001 17100000 5156
bdis5 17100001 17200000 3913
bdis5 17200001 17300000 4297
bdis5 17300001 17400000 3659

bdis2 49700001 49800000 4953
bdis2 49800001 49900000 4701
bdis2 49900001 50000000 4531
bdis2 50000001 50100000 3561
bdis2 50100001 50200000 4254
bdis2 50200001 50300000 4561
bdis2 50300001 50400000 4712
bdis2 50400001 50500000 3956
bdis2 50500001 50600000 3959
bdis2 50600001 50700000 4379
bdis2 50700001 50800000 3817
bdis2 50800001 50900000 5175
bdis2 50900001 51000000 3475
bdis2 51000001 51100000 3617
bdis2 51100001 51200000 4440
bdis2 51200001 51300000 3162
bdis2 51300001 51400000 3862
bdis2 51400001 51500000 4598
bdis2 51500001 51600000 5035
bdis2 51600001 51700000 4938
bdis2 51700001 51800000 4974
bdis2 51800001 51900000 4061
bdis2 51900001 52000000 4719
bdis2 52000001 52100000 4468
bdis2 52100001 52200000 3281
bdis2 52200001 52300000 4562
bdis2 52300001 52400000 3736
bdis2 52400001 52500000 4212
bdis2 52500001 52600000 3979
bdis2 52600001 52700000 4340
bdis2 52700001 52800000 5551
bdis2 52800001 52900000 4897
bdis2 52900001 53000000 4840
bdis2 53000001 53100000 4634
bdis2 53100001 53200000 3938
bdis2 53200001 53300000 4744
bdis2 53300001 53400000 4072
bdis2 53400001 53500000 5065
bdis2 53500001 53600000 5028
bdis2 53600001 53700000 4194
bdis2 53700001 53800000 4609
bdis2 53800001 53900000 4602
bdis2 53900001 54000000 5264
bdis2 54000001 54100000 4007
bdis2 54100001 54200000 4654
bdis2 54200001 54300000 4853
bdis2 54300001 54400000 4709
bdis2 54400001 54500000 4701
bdis5 17400001 17500000 4239
bdis5 17500001 17600000 3929
bdis5 17600001 17700000 3640
bdis5 17700001 17800000 4388
bdis5 17800001 17900000 5035
bdis5 17900001 18000000 4273
bdis5 18000001 18100000 4355
bdis5 18100001 18200000 3848
bdis5 18200001 18300000 4888
bdis5 18300001 18400000 4480
bdis5 18400001 18500000 3475
bdis5 18500001 18600000 4051
bdis5 18600001 18700000 4611
bdis5 18700001 18800000 4593
bdis5 18800001 18900000 3874
bdis5 18900001 19000000 3310
bdis5 19000001 19100000 4407
bdis5 19100001 19200000 5655
bdis5 19200001 19300000 3902
bdis5 19300001 19400000 4178
bdis5 19400001 19500000 5976
bdis5 19500001 19600000 5309
bdis5 19600001 19700000 4728
bdis5 19700001 19800000 4742
bdis5 19800001 19900000 4653
bdis5 19900001 20000000 4842
bdis5 20000001 20100000 5069
bdis5 20100001 20200000 4698
bdis5 20200001 20300000 4560
bdis5 20300001 20400000 4751
bdis5 20400001 20500000 4274
bdis5 20500001 20600000 4415
bdis5 20600001 20700000 4643
bdis5 20700001 20800000 4908
bdis5 20800001 20900000 5196
bdis5 20900001 21000000 3459
bdis5 21000001 21100000 5036
bdis5 21100001 21200000 4208
bdis5 21200001 21300000 5017
bdis5 21300001 21400000 3954
bdis5 21400001 21500000 4041
bdis5 21500001 21600000 4659
bdis5 21600001 21700000 4239
bdis5 21700001 21800000 4493
bdis5 21800001 21900000 5171
bdis5 21900001 22000000 5166
bdis5 22000001 22100000 4889
bdis5 22100001 22200000 5061

bdis2 54500001 54600000 4221
bdis2 54600001 54700000 3893
bdis2 54700001 54800000 4460
bdis2 54800001 54900000 4459
bdis2 54900001 55000000 4271
bdis2 55000001 55100000 5126
bdis2 55100001 55200000 4156
bdis2 55200001 55300000 5052
bdis2 55300001 55400000 4017
bdis2 55400001 55500000 4575
bdis2 55500001 55600000 4952
bdis2 55600001 55700000 4211
bdis2 55700001 55800000 4113
bdis2 55800001 55900000 4308
bdis2 55900001 56000000 4645
bdis2 56000001 56100000 3602
bdis2 56100001 56200000 4586
bdis2 56200001 56300000 4143
bdis2 56300001 56400000 4146
bdis2 56400001 56500000 4370
bdis2 56500001 56600000 3821
bdis2 56600001 56700000 4822
bdis2 56700001 56800000 4176
bdis2 56800001 56900000 4689
bdis2 56900001 57000000 3562
bdis2 57000001 57100000 4121
bdis2 57100001 57200000 4065
bdis2 57200001 57300000 4653
bdis2 57300001 57400000 4897
bdis2 57400001 57500000 4647
bdis2 57500001 57600000 4406
bdis2 57600001 57700000 4038
bdis2 57700001 57800000 4234
bdis2 57800001 57900000 4132
bdis2 57900001 58000000 4858
bdis2 58000001 58100000 4650
bdis2 58100001 58200000 4926
bdis2 58200001 58300000 5462
bdis2 58300001 58400000 5155
bdis2 58400001 58500000 4119
bdis2 58500001 58600000 3432
bdis2 58600001 58700000 4004
bdis2 58700001 58800000 4341
bdis2 58800001 58900000 4286
bdis2 58900001 59000000 3629
bdis2 59000001 59100000 3984
bdis2 59100001 59200000 4063
bdis2 59200001 59300000 3778
bdis5 22200001 22300000 4165
bdis5 22300001 22400000 4519
bdis5 22400001 22500000 4549
bdis5 22500001 22600000 5156
bdis5 22600001 22700000 4825
bdis5 22700001 22800000 5219
bdis5 22800001 22900000 4314
bdis5 22900001 23000000 4141
bdis5 23000001 23100000 4946
bdis5 23100001 23200000 4345
bdis5 23200001 23300000 3902
bdis5 23300001 23400000 5119
bdis5 23400001 23500000 3808
bdis5 23500001 23600000 4126
bdis5 23600001 23700000 3576
bdis5 23700001 23800000 3865
bdis5 23800001 23900000 5101
bdis5 23900001 24000000 4813
bdis5 24000001 24100000 4170
bdis5 24100001 24200000 3929
bdis5 24200001 24300000 4312
bdis5 24300001 24400000 4655
bdis5 24400001 24500000 3347
bdis5 24500001 24600000 4301
bdis5 24600001 24700000 4878
bdis5 24700001 24800000 4483
bdis5 24800001 24900000 3813
bdis5 24900001 25000000 4987
bdis5 25000001 25100000 4787
bdis5 25100001 25200000 4416
bdis5 25200001 25300000 5029
bdis5 25300001 25400000 4922
bdis5 25400001 25500000 4581
bdis5 25500001 25600000 3807
bdis5 25600001 25700000 4567
bdis5 25700001 25800000 5302
bdis5 25800001 25900000 4113
bdis5 25900001 26000000 4745
bdis5 26000001 26100000 4527
bdis5 26100001 26200000 4884
bdis5 26200001 26300000 4939
bdis5 26300001 26400000 4044
bdis5 26400001 26500000 4154
bdis5 26500001 26600000 5192
bdis5 26600001 26700000 4323
bdis5 26700001 26800000 4992
bdis5 26800001 26900000 4102
bdis5 26900001 27000000 4284

bdis2 59300001 59400000 3778
bdis3 1 100000 4074
bdis3 100001 200000 3659
bdis3 200001 300000 4127
bdis3 300001 400000 3957
bdis3 400001 500000 4030
bdis3 500001 600000 4307
bdis3 600001 700000 4001
bdis3 700001 800000 3659
bdis3 800001 900000 4428
bdis3 900001 1000000 3923
bdis3 1000001 1100000 4760
bdis3 1100001 1200000 4305
bdis3 1200001 1300000 3937
bdis3 1300001 1400000 4655
bdis5 27000001 27100000 4830
bdis5 27100001 27200000 4752
bdis5 27200001 27300000 4490
bdis5 27300001 27400000 3745
bdis5 27400001 27500000 4579
bdis5 27500001 27600000 4485
bdis5 27600001 27700000 3946
bdis5 27700001 27800000 4572
bdis5 27800001 27900000 6297
bdis5 27900001 28000000 4880
bdis5 28000001 28100000 4316
bdis5 28100001 28200000 3648
bdis5 28200001 28300000 4544
bdis5 28300001 28400000 4544
bdis5 28400001 28500000 4544

Table S6 BdbZIPs, OsbZIPs and AtbZIPs used

Gene name	locus	Gene name	locus	Gene name	locus
BdbZIP1	bradi1g04510	OsbZIP01	LOC_Os01g07880	AT1G45249	AT1G45249
BdbZIP2	bradi1g05480	OsbZIP02	LOC_Os01g11350	AT1G58110	AT1G58110
BdbZIP3	bradi1g07310	OsbZIP03	LOC_Os01g17260	AT2G12940	AT2G12940
BdbZIP4	bradi1g12620	OsbZIP04	LOC_Os01g36220	AT4G06598	AT4G06598
BdbZIP5	bradi1g17210	OsbZIP05	LOC_Os01g46970	AT5G65210	AT5G65210
BdbZIP6	bradi1g17335	OsbZIP06	LOC_Os01g55150	AtbZIP1	AT5G49450
BdbZIP7	bradi1g17700	OsbZIP07	LOC_Os01g58760	AtbZIP2	AT2G18160
BdbZIP8	bradi1g19700	OsbZIP08	LOC_Os01g59350	AtbZIP3	AT5G15830
BdbZIP9	bradi1g19702	OsbZIP09	LOC_Os01g59760	AtbZIP4	AT1G59530
BdbZIP10	bradi1g29920	OsbZIP10	LOC_Os01g64000	AtbZIP5	AT3G49760
BdbZIP11	bradi1g30140	OsbZIP11	LOC_Os01g64020	AtbZIP6	AT2G22850
BdbZIP12	bradi1g30750	OsbZIP12	LOC_Os01g64730	AtbZIP7	AT4G37730
BdbZIP13	bradi1g31700	OsbZIP13	LOC_Os02g03580	AtbZIP8	AT1G68880
BdbZIP14	bradi1g35550	OsbZIP14	LOC_Os02g03960	AtbZIP9	AT5G24800
BdbZIP15	bradi1g35790	OsbZIP15	LOC_Os02g07840	AtbZIP10	AT4G02640
BdbZIP16	bradi1g36750	OsbZIP16	LOC_Os02g09830	AtbZIP11	AT4G34590
BdbZIP17	bradi1g43660	OsbZIP17	LOC_Os02g10140	AtbZIP12	AT2G41070
BdbZIP18	bradi1g43900	OsbZIP18	LOC_Os02g10860	AtbZIP13	AT5G44080
BdbZIP19	bradi1g46060	OsbZIP19	LOC_Os02g14910	AtbZIP14	AT4G35900
BdbZIP20	bradi1g54180	OsbZIP20	LOC_Os02g16680	AtbZIP15	AT5G42910
BdbZIP21	bradi1g55450	OsbZIP21	LOC_Os02g33560	AtbZIP16	AT2G35530
BdbZIP22	bradi1g63170	OsbZIP22	LOC_Os02g49560	AtbZIP17	AT2G40950
BdbZIP23	bradi1g63840	OsbZIP23	LOC_Os02g52780	AtbZIP18	AT2G40620
BdbZIP24	bradi1g64060	OsbZIP24	LOC_Os02g58670	AtbZIP19	AT4G35040
BdbZIP25	bradi1g64550	OsbZIP25	LOC_Os03g03550	AtbZIP20	AT5G06950
BdbZIP26	bradi1g68560	OsbZIP26	LOC_Os03g13614	AtbZIP21	AT1G08320
BdbZIP27	bradi1g76690	OsbZIP27	LOC_Os03g19370	AtbZIP22	AT1G22070
BdbZIP28	bradi2g04590	OsbZIP28	LOC_Os03g20310	AtbZIP23	AT2G16770
BdbZIP29	bradi2g06790	OsbZIP29	LOC_Os03g20650	AtbZIP24	AT3G51960
BdbZIP30	bradi2g10830	OsbZIP30	LOC_Os03g21800	AtbZIP25	AT3G54620
BdbZIP31	bradi2g15940	OsbZIP31	LOC_Os03g47200	AtbZIP26	AT5G06960
BdbZIP32	bradi2g21197	OsbZIP32	LOC_Os03g56010	AtbZIP27	AT2G17770
BdbZIP33	bradi2g21200	OsbZIP33	LOC_Os03g58250	AtbZIP28	AT3G10800
BdbZIP34	bradi2g21380	OsbZIP34	LOC_Os03g59460	AtbZIP29	AT4G38900
BdbZIP35	bradi2g21820	OsbZIP35	LOC_Os04g10260	AtbZIP30	AT2G21230
BdbZIP36	bradi2g23890	OsbZIP36	LOC_Os04g41820	AtbZIP31	AT2G13150
BdbZIP37	bradi2g24120	OsbZIP37	LOC_Os04g54474	AtbZIP33	AT2G12900
BdbZIP38	bradi2g25400	OsbZIP38	LOC_Os05g03860	AtbZIP34	AT2G42380
BdbZIP39	bradi2g38380	OsbZIP39	LOC_Os05g34050	AtbZIP35	AT1G49720
BdbZIP40	bradi2g40580	OsbZIP40	LOC_Os05g36160	AtbZIP37	AT4G34000
BdbZIP41	bradi2g45567	OsbZIP41	LOC_Os05g37170	AtbZIP38	AT3G19290
BdbZIP42	bradi2g45570	OsbZIP42	LOC_Os05g41070	AtbZIP39	AT2G36270

BdbZIP43	bradi2g50220	OsbZIP43	LOC_Os05g41280	AtbZIP40	AT1G03970
BdbZIP44	bradi2g52590	OsbZIP44	LOC_Os05g41540	AtbZIP41	AT4G36730
BdbZIP45	bradi2g52860	OsbZIP45	LOC_Os05g49420	AtbZIP42	AT3G30530
BdbZIP46	bradi2g53060	OsbZIP46	LOC_Os06g10880	AtbZIP43	AT5G38800
BdbZIP47	bradi2g55550	OsbZIP47	LOC_Os06g15480	AtbZIP44	AT1G75390
BdbZIP48	bradi2g55567	OsbZIP48	LOC_Os06g39960	AtbZIP45	AT3G12250
BdbZIP49	bradi2g55570	OsbZIP49	LOC_Os06g41100	AtbZIP46	AT1G68640
BdbZIP50	bradi2g56080	OsbZIP50	LOC_Os06g41770	AtbZIP47	AT5G65210
BdbZIP51	bradi2g56096	OsbZIP51	LOC_Os06g42690	AtbZIP48	AT2G04038
BdbZIP52	bradi3g00300	OsbZIP52	LOC_Os06g45140	AtbZIP49	AT3G56660
BdbZIP53	bradi3g02730	OsbZIP53	LOC_Os06g50310	AtbZIP50	AT1G77920
BdbZIP54	bradi3g02980	OsbZIP54	LOC_Os06g50480	AtbZIP51	AT1G43700
BdbZIP55	bradi3g05577	OsbZIP55	LOC_Os06g50600	AtbZIP52	AT1G06850
BdbZIP56	bradi3g05580	OsbZIP56	LOC_Os06g50830	AtbZIP53	AT3G62420
BdbZIP57	bradi3g06160	OsbZIP57	LOC_Os07g03220	AtbZIP54	AT4G01120
BdbZIP58	bradi3g06670	OsbZIP58	LOC_Os07g08420	AtbZIP55	AT2G46270
BdbZIP59	bradi3g07030	OsbZIP59	LOC_Os07g10890	AtbZIP56	AT5G11260
BdbZIP60	bradi3g07540	OsbZIP60	LOC_Os07g44950	AtbZIP57	AT5G10030
BdbZIP61	bradi3g09340	OsbZIP61	LOC_Os07g48180	AtbZIP58	AT1G13600
BdbZIP62	bradi3g15590	OsbZIP62	LOC_Os07g48660	AtbZIP59	AT2G31370
BdbZIP63	bradi3g22040	OsbZIP63	LOC_Os07g48820	AtbZIP60	AT1G42990
BdbZIP64	bradi3g31890	OsbZIP64	LOC_Os08g07970	AtbZIP61	AT3G58120
BdbZIP65	bradi3g38200	OsbZIP65	LOC_Os08g26880	AtbZIP62	AT1G19490
BdbZIP66	bradi3g38840	OsbZIP66	LOC_Os08g36790	AtbZIP63	AT5G28770
BdbZIP67	bradi3g41817	OsbZIP67	LOC_Os08g38020	AtbZIP64	AT3G17609
BdbZIP68	bradi3g41820	OsbZIP68	LOC_Os08g43090	AtbZIP65	AT5G06839
BdbZIP69	bradi3g41980	OsbZIP69	LOC_Os08g43600	AtbZIP66	AT3G56850
BdbZIP70	bradi3g45170	OsbZIP70	LOC_Os09g10840	AtbZIP67	AT3G44460
BdbZIP71	bradi3g56290	OsbZIP71	LOC_Os09g13570	AtbZIP68	AT1G32150
BdbZIP72	bradi3g57960	OsbZIP72	LOC_Os09g28310	AtbZIP69	AT1G06070
BdbZIP73	bradi3g60870	OsbZIP73	LOC_Os09g29820	AtbZIP70	AT5G60830
BdbZIP74	bradi4g00810	OsbZIP74	LOC_Os09g31390	AtbZIP71	AT2G24340
BdbZIP75	bradi4g02570	OsbZIP75	LOC_Os09g34060	AtbZIP72	AT5G07160
BdbZIP76	bradi4g04720	OsbZIP76	LOC_Os09g34880	AtbZIP73	AT2G13130
BdbZIP77	bradi4g22130	OsbZIP77	LOC_Os09g36910	AtbZIP74	AT2G21235
BdbZIP78	bradi4g24937	OsbZIP78	LOC_Os10g38820	AtbZIP75	AT5G08141
BdbZIP79	bradi4g24940	OsbZIP79	LOC_Os11g05480		
BdbZIP80	bradi4g26670	OsbZIP80	LOC_Os11g05640		
BdbZIP81	bradi4g27100	OsbZIP81	LOC_Os11g06170		
BdbZIP82	bradi4g27720	OsbZIP82	LOC_Os11g11100		
BdbZIP83	bradi4g32090	OsbZIP83	LOC_Os12g05680		
BdbZIP84	bradi4g32920	OsbZIP84	LOC_Os12g06520		
BdbZIP85	bradi4g33740	OsbZIP85	LOC_Os12g09250		
BdbZIP86	bradi4g35240	OsbZIP86	LOC_Os12g13170		

**BdbZIP87**	bradi4g35370	**OsbZIP87**	LOC_Os12g37410
**BdbZIP88**	bradi4g36587	**OsbZIP88**	LOC_Os12g40920
**BdbZIP89**	bradi4g39630	**OsbZIP89**	LOC_Os12g43790
**BdbZIP90**	bradi4g40540	**LOC_OS02G08540**	LOC_OS02G08540
**BdbZIP91**	bradi4g41890	**LOC_OS03G19375**	LOC_OS03G19375
**BdbZIP92**	bradi4g42120	**LOC_OS05G03865**	LOC_OS05G03865
**BdbZIP93**	bradi4g43850	**LOC_OS06G42140**	LOC_OS06G42140
**BdbZIP94**	bradi5g14497	**LOC_OS06G43870**	LOC_OS06G43870
**BdbZIP95**	bradi5g14500	**LOC_OS09G13575**	LOC_OS09G13575
**BdbZIP96**	bradi5g23340	**LOC_OS12G06100**	LOC_OS12G06100
		**LOC_OS12G37415**	LOC_OS12G37415

Table S7 BdbZIP protein sequences

>BdbZIP1

MAASSSSVTSGDNGPPNGGNGTPPPPIHGDWASSMQAYYVAVSVAGHHPYAAWPPPPQGAPYMVGEAASAVTVEGNNRKR
KTTRVPSGDDASDDDGSQGSSAKTAPGADPDQKV

GENDSWHFSGEPSQAATTMMHNAVTEAPFMGKGRSASKLSVLAPGRVARTNAIPNLNIGMGHSNTSSSTMMPSGQGEVNV
GASSQSNGSLSRMVWFSCMESDNAIGRMETSCNL

DEHGVVELVSVLALRDVTWPKDYLYLLSDERELKRERRKQANRDSARRSRLRKQQECEELAQKVTELTAINGVLKSEIDQ
LKKDCEDMEAENTQLMDEVLTHDDEMLESEDPSV

LTTLSIQVDVLTARRGRNSKLHKSNNDVSKG
>BdbZIP2

MERVFSVEEIPDPFWASQPPASRDSNAGTGAGPAAPGEGGGGAMNRCPSEWYFQKFLEEAVLDSPVGGNPSPRAAPGGGG
GVVVGGAEAVEVKQQPAPAPAAAAAAAATSAVVD

PVEFNAMLKQKLEKDLAAVAMWRATGVMPPERFAASSSLPNADVSHIGTTNPIGGNVVPVQNQLVGGTSGEQGPHFVQSD
TLVKQAASSSSREQSDDDDMEEDEITGNANPTDQ

RLRRRKQSNRESARRSRSRKAAHLNELEAQVSQLRVENSTLLRRLADVNQKYNGAAVDNRVLKADVETLRAKVKMAEDSV
KRVTGMSALFPPGSDMSSLSMPFTGSPSDATSDA

AVPDDLNNYFSTNSDVGGSNGYMPEMASSAQEDDDFINGALAPGKMGRAESLHRVASLEHLQKRMCGGPGPASSGSTS
>BdbZIP3

MLSLQEAIDFDHLDVMTSGFGGFTPWGPDTCPTLEQLMASSSSSVAAEEEDEEELQRRQRRKLSNRLSARRSRARKQQRL
DELRAEAAQLRAQKKELGARLHAAARHGLAARGQ

NARLRAEAAALARRLRDARRLLALQRLTRQLLVLRPPQPAQPAGNGAAGQAGPGAADFAAPAVAAPQGLLAAASLMT
>BdbZIP4

MTTSQGSNAFQSFAPQSNNGFGTNAELTAHPLLPIEDPACAAASAAIGGVPDHEASDGVFNKERRLKRKISNRESARRSR
ARKQRHLDDLRALAARLRHGNRELSARARAARGR

VALVRLANAELRAEADALGRRLEAAARQALALGQLYAAAHGHGAFEQTMASLMV
>BdbZIP5

MADASSRTDTSTVVDNHSKNHRLEQGQSGALMASNSSDRSDRSDKPLDQKTLRRLAQNREAARKSRLRKKSYVQQLESSK
LKLAQLEQELQKARQQGIFISSSGDQTHAMSGNG

ALTFDIEYTRWLEEQNKQINELRTAVNAHASDSDLRLIVDGIMAHYDEIFKVKGVAAKADVFHILSGMWKTPAERCFLWL
GGFRPSELLKLLANHLEPLTEQQLLGLTNLQQSS

QQAEDALSQGMEALQQSLAETLAGSLGTSGSSGSSGSSGNVANYMGQMAMAMGKLGTLENFLRQADNLRQQTLHQMQRIL
TIRQASRALLAIHDYFSRLRALSSLWLARPRE
>BdbZIP6

MASLGGGGGGGGRQGAVYGLTLNEVESRLGSPLRSMNLDELLRTVLPAAAAGGGPGPGSGKKTVDEVWRDIESGARGRQS
AAMEVGEMTLEDFLSRAGVPVDGGGAHWLLRQYH

PPPRSLPVFGHGGGGGGGFLSPSHAGGGRKRGAGGEDEGGGGGGVERRQKRMIKNRESAARSRARKQAYMNELENKVSRL
EEENRRLKELKRLEPMVQVQCVTRPEPMLQRLEP

MVVHYVTRPESMVVQYVPEPEPEPEKQLQHQLQLRRTISASF
>BdbZIP7

MPPKPGEPPKPPPSGRSPNSLNLPSPLPPVPQPGHHHSPAAGGHHPPPHHRRARSEVAFRFPANVLAGIAGPEDELFSTF
MDTDKIAGTSSSPPRSAKHRHSASFDGGGGAGKH

KGGVGGVFSDVLEAKKAMSSEELSELAFVDPKRVKRIIANRQSAARSKERKARYITELERKVQTLQTEATTLSAQLTLFQ
RDTTGLSAENTELKIRLQAMEQQAQLRDALNDAL

KQEVERLRIATGETTKSNEAYDRGMHHVPYSPSFFQLSEQHSVQHRASVHQLPPQFQPPHPSAPGHQMASHPNTFPDMMQ
QDSLGRLQGLDIGKGSVPVKSEADEPLKSEGSSL

SANESNSTF
>BdbZIP8

MAEPCLFADMPSFLDDLPEFPHPPDDETFALEDFDLEDLDFDFDLDLFSTDDAQLSTPPPPPPPLATSSSSAGSPGGGSS
SSGAAVDGAGGGLKNDESSESSSRSASAVSDGKP

RNGEDEEAKRRARLVRNRESAHMSRQRKKQYVEELEGKVKAMQATIADLSARISCAAAENAGLKRQLSGVAGTPAPPPLP
MYPGLYPLPPPWLHPAYAMRGSQVPLVPIPRLKT

RKPAPAEVEPPAKKTRKTKKVASVSLLGLLFLVMVCGCLVPAVNRMYGTVDSGEGIVLAPSHHGRVLAVEGPRNGAPDGI
DSKLPQNSSETLPALLYLPRNGKHVKINGNLVIQ

SVVASEKASSRMSQYDPKISGNPGKEETSLAIPGHVAKLDSGEVPKSAQGIKNKLMVLPPGDRTIYREDDELLPQWFSEA
MSGPTLSSGMCTEVFQFDISPTSADANGIVPVYS

SAMPNSSHNLTENLPSTRPQKIKNRRILHSVPIPLQGSTSNHTDRLKEHPKNESFAGNKPASSVVVSVLADPREDSEGRI
SSKSLSRIFVVVLVDSVKYVTYSCVYLSCRVASM

YIRVKRHKSTYFIQCDPTETILNIKQKLESIIDHPPNNQRLILLATNNVLNDSKTLADQKVENDTIVALTLRKVIAVTCD
SPLPLKKVQGKKLVLITVGENEQRICNLQECAGI

HRRMAESLISVWTKYHPYSLKSSKGAPLPPRPTLVFLIAVFGLYVCYLSFNQIRLENEGGENSAEEHTEHVCTKPSVPSE
ELRYVHLPKPKGYNRGECSCTPVRFFVIVSMQRS

GSGWFETLLNSHPNISSNGEIFNRIDRRENLSSIVQTLDKLYNLDWLTSAAKNECTAAFGLKWMLNQGFMDHHDDIVSYF
NQKGVSLIFLFRRNTLRRLISVLANNYDRDAKQL

NGTHKSHVHSEEEAEILAKFKPELDVSTLILDIRDIEKYIRDCLDRFNTTRHMILYYEDIISNRNALFRVQEFLGVPARK
LVSKQVKIHTRPLPDLVKNWEDVNSKLNGTEYAR

FLDGADYVK
>BdbZIP9

MTGSAAHMAEPCLFADMPSFLDDLPEFPHPPDDETFALEDFDLEDLDFDFDLDLFSTDDAQLSTPPPPPPPLATSSSSAG
SPGGGSSSSGAAVDGAGGGLKNDESSESSSRSAS

AVSDGKPRNGEDEEAKRRARLVRNRESAHMSRQRKKQYVEELEGKVKAMQATIADLSARISCAAAENAGLKRQLSGVAGT
PAPPPLPMYPGLYPLPPPWLHPAYAMRGSQVPLV

PIPRLKTRKPAPAEVEPPAKKTRKTKKVASVSLLGLLFLVMVCGCLVPAVNRMYGTVDSGEGIVLAPSHHGRVLAVEGPR
NGAPDGIDSKLPQNSSETLPALLYLPRNGKHVKI

NGNLVIQSVVASEKASSRMSQYDPKISGNPGKEETSLAIPGHVAKLDSGEVPKSAQGIKNKLMVLPPGDRTIYREDDELL
PQWFSEAMSGPTLSSGMCTEVFQFDISPTSADAN

GIVPVYSSAMPNSSHNLTENLPSTRPQKIKNRRILHSVPIPLQGSTSNHTDRLKEHPKNESFAGNKPASSVVVSVLADPR
EDSEGRISSKSLSRIFVVVLVDSVKYVTYSCVLP

FKNHSPHL
>BdbZIP10

MASFGGQYVGTGAWMREPESPQLSLMSGCSSLFSISVLRDGDDLGGGVRSLPATPVSLAGFVGAGDEVEMMDHLRQGSGD
EDRRTVRMMRNRESALRSRARKRAYVEELEKEVR

RLVDDNLKLKKQCKELKQEVAALVLPSKSSLRRTSSTQF
>BdbZIP11

MDDGDLDFNPDTYLCSGAAAGGTETPGACSMDSYFDEILKDTEHLACTHTHTCNPPVHDLSHTHTCVHVHTKIVSASSDG
AESPAENTTSGTSKKRRPSGNRAAVRKYREKKKA

HTALLEEEVVHLKALNKELMKKVQNHAALEAEVARLRCLLVDIRGRIEGEIGAFPYQRPVKNVDLVSGGVDLLGGGSQVM
NSCDFRCNDQLYCNPGMQMRTVGDDGAMNGQAFG

QGTGDFVNVQCLGSAKSGSTISPGCGGMSNMPFGCLPNAKK
>BdbZIP12

MAEVNEEESCLAVLAEAAGIICSLRAGDLAGWTPPWTTGPTAEALVGPTKEEMGAWPAVTRGKRSRSSRGSGSAAAKVGR
RRARGSPASPLDYSAASGSGSGVSTSGGEDGAVF

CSPPAPAAAASAAAVPQSVPTTAATASSAKVCSAGVRRPPPRPAAPGPRSRKKMRLPEVQQLVRSLAAENDGLREEVESL
QRACSALSRENGTLETRLEHSSSKRKRTVSEEED

RPRQRKPMLQQLGQHAAEHRGAREGFVLPDLNIPAAAPDDVGSAP
>BdbZIP13

MKKCASELELEAFIRQHLAAERNRPSPGTDAGVFSSTHGGGLPVPGLCFGDSQKALELEGSDAGHQWWSDGVCAPHPAVS
STMGSQTAAVSASPRETTSGNQALESESESGSES

LIDIEGGQCNRKSTDTMRIRRMVSNRESARRSRRRKHAQLTDLELQVEQLKSESASLFKQLTEASQHFTSAVTDNRILKS
DVETLRVKVKMAEDMVARTAMSCNVGQLGSAPFL

NSRKMCQALDMLTATGLDLPGNHALFKDPTPARQVQTSTVQSTASLESLDNRMSSEVTSCAGDMWP
>BdbZIP14

MQRDLASLACYSSAAGAASFFLPQAQQQPGSSSSDVVVDGLLGSVSVGGHGHGHHGPQCSGGAGASWRAAGAPVDVAGSC
EEARRKARRLASNRESARRSRVRRRRQLDELSAC

AAELRADNQRLVVALNRAEARHARVVRENQRLREEARRLRERLGESGDDDEEDEEEAAAGRAP
>BdbZIP15

MDADLDLDALLASFDGESAISSLFPPPPPDAEAGSPESVSSRSNPAGEEVLSEIERFLMEEEAAEAEGVEGISVEEFFDA
LFDGAEGAEKAKESEAGGSTDGDSGREEERVEVL

TPETEVDVEKVDGDDPISKKKKRQMRNRDSAMKSRERKKTYVKDLEVKSKYLEAECRRLSYALQCCAAENVALRQSMLKD
RPVGAPTAMQESAVLTETLPLVSLLWLVSIVCLF

LTPGLPNQSPVAPRSVGRDLVMVAGKLSSDQPETLELLLHGRRHRGKRGRMKLDALPFHAAAAA
>BdbZIP16

MDADLDLDALLASFDGESAISSLFPPPPPDAEAGSPESVSSRSNPAGEEVLSEIERFLMEEEAAEAEGVEGISVEEFFDA
LFDGAEGAEKAKESEAGGSTDGDSGREEERVEVL

TPETEVDVEKVDGDDPISKKKKRQMRNRDSAMKSRERKKTYVKDLEVKSKYLEAECRRLSYALQCCAAENVALRQSMLKD
RPVGAPTAMQESAVLTETLPLVSLLWLVSIVCLF

LTPGLPNQSPVAPRSVGRDLVMVAGKLSSDQPETLELLLHGRRHRGKRGRMKLDALPFHAAAAA

>BdbZIP17

MTCTKARALRQRMRREWKLPAEANLRCSGNDWVLILLDAVDAKTRQFLMLLWWRAWHLRNDAIFGDGTASVEVSASFISN
YAIALDNINAGDKAQVSCTLSTTGTKVQQRVLTA

GSSSSCQNQLEASWGAVLRSYSGQVLAFAWGHGPRCSSADEAEAHACLHGIEAPCADVNMKLGDKLRSNITLTKSASSPN
GTADVLLYTYVGLSDSHGRKRPNETKNAPWSMST

ASPKRNPALEGRVQAISIGGTPHRADASTVSFEPHRYQYHQIAAAHDHFLFQYCHGFVDDEPFPLDNPPPPAPAIRNNGS
TTSSDEPAAGAERQRAEERRKRRVASNRESARRS

RVRKQKQLGQLRAQAAQLRDANRELLDRLNRAIRDCARVVRDNSRLREERAELHRRLRELVVPVPVVDGDAGVEVESSVA
GIDEDDDESIMVAIATT
>BdbZIP18

MTAPGALTEVDRFCLPRASAQFENWGDSGIVVTSPLTETSTDLDDSADKRLVSMGGGGGAQRWVGGCVDTSERKGDQKIE
RRLAQNREAARKSRIRKKAYVQQLESSRSKLAQL

EQELQRARQQGIFVGSGGSSDHGCSTGGALAFDLQYARWLDGYQYHVNDLRVGVHANISDDELRILVEAVMLHYDHLFRL
KSIATKSDVFHVMSGMWMSPAERFFMWLGGFRSS

ELLKVLASQLEPLTDQQLMGICNLQQSSLQAEDALSQGMEALQQALAETLAFAAAVVPSTGSGDNVTNYMSQMAIAMAKL
STLENFLRQGDLLRQQTLQQMHRILTTRQAARAL

LVISDYFSRLRALSSLWLARPRA
>BdbZIP19

MEMPGGSGAPALARQGSIYSLTFDEFQSALGGAGKDFGSMNMDELLRNIWTAEESNAIAAAATATTAVPASNVDAQPPQP
QQQAILRQGSLTLPRTLSQMTVDEVWRDIMGFCD

DEPEAPVPAQLPAQAQRQPTLGAMTLEEFLVRAGVVREDMGGQTVVVPARAQALFPQSNVVTPTMQVGNGMVHGVVGQGA
GGGMTVAAPATPGVLNGFGKVEGGDLSSLSPVPY

PFDSAMRVRKGPTVEKVVERRQRRMIKNRESAARSRQRKQAYIMELEAEVAKLKEHNEELQKKQVEMLKEQKNEVVERIS
QQLGPKAKRFCLRRTLTGPW
>BdbZIP20

MASSSDEQPKPPEPPAAAVATAVPPQTHAEWAASVQAFYAAAGHPYAAWPAQHLMAAAASGAPYGAPVPFPMYHPGAAMA
YYAQASMAAGVPYPTAEAVAAAPAVAEGKGKGKG

GGVSPEKGSSAAPSGDDGSRSCDSGSDDSSDTRDYDTDHKDSSAAKKRKSGNTSAEGEPSQTAVVTYAAESPYQLKARSA
SKLPVSAPGRAALPNATPNLNIGIDLWSASQPVA

VLPGQGEASPGLALARCDGVGQLDEREIKRERRKQSNRESARRSRLRKQQECEELSRKVAELTTENNALRTELDQLKKAC
EDMEAQNTRLMGEMIQSQEPAAVTTTLGMSIEAP

KVKQHEDEGKLHKKSNNNSNGKYVGGSRKPEANSR
>BdbZIP21

MKRKFPLEEETPNPDPFRIPSPPSPLSMFQTQVVDGGVEGSSGDVMNPSLPDWCFLEESLLSIPNPSASNPNDLVLDPDV
MMDTSPKRRCVDPEVERVEVIPRPLPSPATASVM

QDPAVYNAMLREKLDEDLAAVALLTASRSSQSNTTSNQGSQNYIGDNEILVQQMSGGGQYGGTSPNLAQNPDVSVRQVSS
PLREQSPSTNNIEGEAETMGNMNFSAEKVKMRRE

SNRISARLSRYKKATQMQNLQHQLSLLEAENKYLVKRQADLIQKYSSAVIDNRVLKANVETLETKVKLVEEIIKRFTSTH
DVPQVVSSLTSLGFPLSASPSNGAHETFVPTQNT

PFNYFTSVTTNGGVNNIYTPEATSTFQIQDPVALLQMQSESSLEHLQRRVCDSAPSSSVLAPQEVTSFNPNEFINMGMQ
>BdbZIP22

MQQPKPADPPARPFPPPSPAMAAAATAMRGAHHRRARSEVAFRLPDDLDLGSAGEGDGSAGFDEIGSEDDLFSTFMDIEK
ISSGPAAAAAAGSDRDRAAETSSPPRPKHRYSSS

VDGSGLFSAGSSAARKDAAAAQALADVLEAKKAMSPEQLAELAAIDPKRAKRILANRQSAARSKERKARYMTELERKVQT
LQTEATTLSAQLTLFQRDTTGLSAENAELKIRLQ

AMEQQAQLRDALNDALKQEVERLKMATGEMSNSNDTYSMGLQHVLYNSSFFPQSQQNTSQHQGGARFPPPFHPPHPNVPN
HQMLSHPNTLSDVMQQDHLARLQGLDISKGHPVV

KSESSSISASESSSTF
>BdbZIP23

MSSGGAISRQGSVCSLTLSEVEGQLHGVNLDDLLRTAGSARKTADEVWRDIQSGGGRALPPAPGQMTLEDFLSKSVSDAR
WAEQYNPPPPAPAKGGQQQQRHSVGRPLPRPLGV

GAEPVLDALLYHDGPPPLNGRKRAAEAGLGGPGEKTVERRKKRMIKNRESAARSRARKQAYTNELENKISRLEEENELLR
SYKAFEPVVHYVPQEEPKNQLRRRNSASF
>BdbZIP24

MADASSRTDNSIVVDTDDKNQRMENGQNGAMVPSNSSDPSDRSDRPMDQKVLRRLAQNREAARKSRLRKKAYVQQLESSK
LKLASLEQELQKARQQGIFISSSGDQTHAMSGNG

AMTFDLEYTRWLEEQNKQINELRTAVNAHASDSDLRLIVDGIMAHYDEIFKLKGAAAKADVFHMLSGMWKTPAERCFLWL
GGFRSSELLKLLVNQLEPLTEQQLLGLSNLQQSS

QQAEDALSQGMEALQQSLAETLAGSLGPSGSSGNVANYMGQMAMAMGKLGTLENFLRQADNLRQQTLHQMQRILTIRQAA
RALLAIHDYFSRLRALSSLWLARPRE
>BdbZIP25

MVPRQRPALAPRRRAITEEERRRNRMTSNRLSAQRSRMKRQQREEDLAAQASRLKLENEAMRAAAGIRQQQCRLLQQENR
VRAAHARELYAVLQLRNSQLRMLGQAADLPLDVP

EVSAHLTQLYGGGPPAVPPLPPEIYQMLQFQPPLERQIDQMLFQPPLSPEIDQTLFQPPDDVMDDEAS
>BdbZIP26

MGKGDGTTRSKSQKSSATQNEQSTPTNPPTAYPDWSQFQAYYNVPGTAPMTPPAFYHSAVAPSPQGHPYMWGPQMMPPYG
TPPPYATMYAQGTPYQQAPMPPGSHPYSPYPVQA

SNGTVQTPPSGAGGSETDKSSKNKRKTPLKRSKGSLGSLDVVTVKNKMSPAKPLASSSNEGSSQSESGSGSYSEGSSTNS
KSGSRTKDEHGQGNDASNKGATASSAVEPTQVSS

GPVVLNPMMPYWPVPPPMAGPAGPATGVNMGMDYWGAPTSVPMHGKVAAAPTSAPSSNSRDIILSDPAIKDEREVKRQKR
KQSNRESARRSRLRKQAEWEEVANRADLLKQENS

SLKEELKRLQEKCDSLTSENTSLHVSCKLCLLFLSILSHSCSVLTHGLVT
>BdbZIP27

MDSRAAKLMQQQGMQPLSLPMATSFATEAAAAKPRAAGLPPTPPFSAAAGGGVQRDVCMEDSSSSAAAALKAAAHRRSRS
DVPYGYSPSMASAMAMGRLQPKTEWGQPQQHLVE

NNGFNGNGNGGDDLFNAYMNLEGLDALNAAASSSPDSRGSSVKAESSENEGYGSEEGAVRGALWADGNAGSGSNKRSAAA
AGAVGEPAAANVARHARSLSMDSLMGRLNFASGA

GASASAARAANGGAGGGSVFSLEFGSGEFTPVEMKKIMADERLAEMALADPKRVKRVLANRQSAARSKERKMRYIVELEQ
KVQMLQTEATTLSAQLTLLQRDSAGVATQNNELR

FRLQAMEQQAQLRDALNDALTGEVQRLKIATAEIGVGDSCSSSGMAQQNQMFQLQQHQGQATPRPFYQLQQQQQQHNGNH
EPRE
>BdbZIP28

MAAQEQEQEKQQAKTSTTSSLPSSSERSSSSGPNNLKEGGAESDEEIRRVPEMGGGSASSGAGDGKQLLLQQHGAGGQPP
ASASGKKRGRAAGDKEQNRLKRLLRNRVSAQQAR

ERKKAYMTELEVKAKDLELRNAELEQKVSTLQNENNTLRQILKNTTAHAGKKSGGGGKGGDGGKKQHHFSKS
>BdbZIP29

MAPALAGQQGAAAPASDEFLDFSAAKRGAHRRTVSDSVAFLDDDNAGAGAAAHEFERLDDAQLMSMFSDDLTPPPPPQQP
AAPSASSPSDHNSNINDEKQDNKGGEAEEAQSGC

NGGGGGSAAPYSPATVDPKRVKRILANRQSAQRSRVRKLQYISELERSVTSLQTEVSALSPRVAFLDHQRSLLTLGNSHL
KQRIAALAQDNIFKDAHQEALKKEIERLRQIYQQ

QSPKNAESPTPDAPLQVRSDNGLIANEGTAGPAPCLPS
>BdbZIP30

MAEVSPRTDTSTDDTDENHTLEPGQVALVVSDSSDRSRDKNGDQKTMRRLAQNREAARKSRLRKKAYVQQLENSRLKLTQ
LEQELQRARQQGIFISSSADQSHSTSGNGAIAFD

MEYSRWLEEHNRQVNELRAAVNAHASDNDLHSVVEKIMSHYEEIYKQKGNAAKADVFHVLSGMWKTPAERCFLWLGGFRP
SEVLKLLSTQLEPLTEQQLSGICNLQHSSQQAED

ALSQGMEALQQSLAETLAGSIGTSGSTGNVANYMGQMAMAMGKLGTLENFLRQADNLRQQTLQQMQRILTTRQSARALLV
ISDYSSRLRALSSLWLARPKE
>BdbZIP31

MAHDEAVVTHKTGKTASPPKDQPAPCPYPDWSAIQAYYGSGVLPPTYFAPAIAPGHAPPPYMWGPQPLMPPPFGTPYAAM
YPHGGAYPHPLVPMMANPLSMEPAKSASSKEKSS

NKKLKEIDGTAVSTGSGNSKKTTSSSEAYSGEGSSDVNDLKVSRTPRKRSMDGGLGTEATGAARNEDVLMGNGAILPNHL
FPAPVIKPSVSNAAKSGAMGTPISPPPGVIIPSH

TVVSTELSTKDERDLKREKRKQSNRESARRSRLRKQAETEELATQVESLTAENTSLRSEISKLTENSEKLRLENSALAVK
LKNTTVPTNVEMPVDKPAAVASSSPRIVENFLSM

IDDTTKSNVNNHTEHSEPKLRQLLGSSATTDVVAAS
>BdbZIP32

MDDGVDLPSQFLFSHPEPEMPGAFDDLLSNNASTSSCSHTHTCNAPGPSAAMHSHTCQHTHTKVFATGSEDDDGNPAAKT
RRPLGNREAVRKYREKKKARAAFLEEEVRKLRAA

NQQLLRRLQGHGALEAEVVRLRSLLPDVRAKIDAEVAVPVAVTPFQKMPLQCSVGSVVCSDRPALCFNGNSEAGAWEESS
RPAAAGCRFEEDGNGGVAREIDVLEQVHSMDVAD

LCFHS
>BdbZIP33

MDDGVDLPSQFLFSHPEPEMPGAFDDLLSNNASTSSCSHTHTCNAPGPSAAMHSHTCQHTHTKVFATGSEDDDGNPAAKT
RRPLGNREAVRKYREKKKARAAFLEEEVRKLRAA

NQQLLRRLQGHGALEAEVVRLRSLLPDVRAKIDAEVAVPVAVTPFQKMPLQCSVGSVVCSDRPALCFNGNSEAGAWEESS
RPAAAGCRFEEDGNGGVAREIDVLEQVHSMDVRI

FISNLSIKGKKLTYGIGLSTYSMKLFSKLSYEDHYRCKLLAAPIRIPSYQLLKEVRNPAHQRTSGHKPAW

>BdbZIP34

MDYASPGGTDTSTDPGIAKKNQMTLRRLAQNRDAARKSRLRKKAYVQKLESSSLKLAQLEQELLRARQQGYVTSTLGEQP
HPANGNGALALDVEYGRWLEEHNKQIDELRAAIS

ARATDGDLHAIVENIMAHVDEIFRLKSVATKANAFHVLAGAWTTPVERCFLWLSGFRPSELPKLLASQLEPLTEKQLASI
CSLRQSSQQAEDTLSRDMEVLLQSAAEIVASGTS

PTWYPAGSSGDTGQMSAAIGKLGAVESLLQQADELRLRILRDVQRILTTRQSARALLAISGYFSRLRALSSLWIARPSTG
MN
>BdbZIP35

MIQAMSSHAGGSGGGGGYGGQARRGQMQSLARQGSLYSLTLNQVQSQLGEPLISMNLDELLKSVFPDEYDPESGPVASQS
EQALGLQRQGSIMMPPELSKKTVDEVWKCIQDSP

NTGAEEGGQQRRERQPTLGETTLEDFLVKAGVVTEGYLKDPNDLTANVNVVGSSVIASGAPSLNPGAQWLQQYQQQALEP
HHPSMPGSFMASQLGPQPLAVGTGDILESIYSDG

QMTSPMLGALSDPQTPGRKRSASGGVPDKVVERKQKRMIKNRESAARSRARKQAYTNELENKVSRLEEENERLKKQKELD
MIIFSAPPPEPKYQLRRTSSSPV
>BdbZIP36

MPKAWILELQPAAAAYFGELGEALIHGTCAGVNPVMIEGDAHTKSAAYLAARPPTLEIFPSWPMSHLQQPYSANSQSVGS
TDSSSAQNTMSQAELVSPVSMRTDCGQQQEVLMV

TIDDYNYNQGLGPAAAATVTAPSFQQHAGGQDKRKHGSTRKDDKLLDPKTERRLAQNREAARKSRLRKKAYVQQLETGRI
RLQQIEQELQRGRSQGLLIGGCSAPGDTSPGAVM

FDMEYARWLDEDSKYMIELQSALQAHILDGNLGTIVEECLRHYDELFHLRGVLARSDVFHLMTGMWATQSERCFLWMAGF
RPSEILKMLTPQLDPLTEQQLLGMFNLQQSSEQA

EEALAQGLKQLHQSLADAVGAGPLNDGADVANYTSLMALALDRLDNLESFYREADNLRRQTLHRMRQILTTRQTARCFLS
IGEYHRRLRALSSIWAARPRENFIMAENVSSTGT

EFQVHQSQQNQFSGL
>BdbZIP37

MAASSQPPRSGSSDLARFRSASGIGSMNMDDILRNIYGETPPAGAGGASGEPSPAPEAAARRTAEEVWKEISATGGLSAP
VPAPAPAGAGGGDGGGASVMTLEDFLAREEDARV

TAVEGNMEVGFPDGAEGVVGGRRRGGGGGGRGRKRAPMDPMDRAATQRQKRMIKNRESAARSRERKQAYIAELEAQVTQL
EEEHAELLREQEEQNEKRLNELKEQAFQVVIRKK

PSQDLRRTNSMEW
>BdbZIP38

MAEPALLDPSPFDLRHFPPYLFDTDLHLAGDDLPLENFAGGGDGCDDLDFDLPVDFSVEDFLLRSPDRGDSGEGSAAGSG
PTGSSSSPAASAADSVVAGGSCGVKHEESDEGRS

GAAPNWGLKRKQACPAVSSDAAKSRRSGDGEVSPSASASRAAMESDDGGTVGEGEDTRRAARLIRNRESAQLSRQRKKRY
VEELEEKVKSMHSVINDLNSKISFIVAENATLRQ

QLSSGGGSCPPPGVYPPAPMPGMHFPWVPGYALRPHGSHVPLVPIPRLKPQQSVSATKVTKKHENKKAVDSKSKTKTKKV
ASVSLIGLLLFVLLFGAFVPGFNHNFGMRGGSDS

TMFRSFGHPPGRVLSFTNHGKGTKGGSNNSDMIDVDSGMMMANGDSTEQKHAPNSSETLPALLYVPRNGKHVKINGNLII
HSVLASEKAVAHTASKHNNGQSDIDHKETSVAIA

RHLSLPGNNMKPQEKSPVDGPLPQWFREGMAGPILNSGMCSEVFQFDISTASTNSGGVIPASPIVNSSSINATQSIPTPP
PAYLGKLKNRRIMYNEAIPLTGKTVNNTEPFNRT

SENSKLPDKKPSSSVVVSVLADPREAGDGDRDPRISTKSLSRIFVVVLLDGVRYVTYSCTLPFKSASPHLVN
>BdbZIP39

MSRRSSSPESNIDGGSGSGSAGDERKRKRMLSNRESARRSRARKQQRVEELIAEASRLQAENARVEAQVGAYAAELSKVD
GENAVLRARHGELAGRLQALSGVLEIFQVAGAPV

DIPEIPDPLLRPWQSPFAPQLIPATAAGAMADAFQF
>BdbZIP40

MASPGITTSSAGSVGAATPAVALTEERKRKRKESNRLSAQRSRARKQRQVDDLEAQVAAMRARNCAMAAAANEAERLCAA
VQAENALLSARALELSARLESLTDLIQCMDAVMY

SNNNTNNNSYYY
>BdbZIP41

MGHDEAVVIQNSGKAPSPPKDQPALYPCLDWSTMQAYYGPGIMPPTYFCPGLVPGHAPPPYMWSPQPLLPSASAKPYTAV
HPHGGGFSHPFMPLMVNPLSVEPAKSVNSDENNQ

NKKLKEVDGTAVSTGSGHSEKTSWDCSVGGSSDGNIQKASGTPKKRRLHGTPIADGKKALHYSAALETGGTTTGNDVPGE
QGRPTNLPSLYIPDRAIKPNASTASDFSVIGTPI

STEFPDQDRKESKRERRKQSNRESARRSRLRKQAETEELAKKVELLTAENTSLRRDIRRLTESSKKLRSENSALMATLTE
AAPDQTPEASADQTAEQSARAAKNFMPVMDSTSA

SRNSGHMAHGVPKLRQLLGSRLASDAVAAR
>BdbZIP42

MGHDEAVVIQNSGKAPSPPKDQPALYPCLDWSTMQAYYGPGIMPPTYFCPGLVPGHAPPPYMWSPQPLLPSASAKPYTAV
HPHGGGFSHPFMPLMVNPLSVEPAKSVNSDENNQ

NKKLKEVDGTAVSTGSGHSEKTSWDCSVGGSSDGNIQKASGTPKKRRLHGTPIADGKKALHYSAALETGGTTTGNDVPGE
QGRPTNLPSLYIPDRAIKPNASTASDFSVIGTPI

STEFPDQQDRKESKRERRKQSNRESARRSRLRKQAETEELAKKVELLTAENTSLRRDIRRLTESSKKLRSENSALMSHLL
SQATLTEAAPDQTPEASADQTAEQSARAAKNFMP

VMDSTSASRNSGHMAHGVPKLRQLLGSRLASDAVAAR
>BdbZIP43

MAQLPPKIPMPAHHHWPGGVADAHHHHHQVAWADEFAEFAAARRGAHRRSLSDSVAFVEVMAPGGAGDFDRLDDDQLMSM
FPDEGHGGGGSTTAPGSENGTSSDSDGVKHRKDH

PQYDDDEQQNDNEANPEEEPAPGRATPTSSTETIRDPKRVKRILANRQSAQRSRVRKLQYISELERCVTTLQNEVSVLSP
RVAFLDQQRTILTVGNSHLKQRIAALAQDKIFKD

AHQEALKEEIERLRQVYQQQNLRMSSAPASDHAHGGPPPVRAEKELIS
>BdbZIP44

MDDGLYIPIPSHLLFPHPEIFSHGFDEFLMNTTAIPTCTHTHTCNNAPSSPLVAMHTHTCLHRHTQVLASAEQEPRNPRV
IKPLGNREAVRKYREKKKAHAAFLEEEVRSLRAA

NQQLLRRLQGHAALEAEVVRLTSLLFDVRAKIDAEIGDLPLQQKPCAFGTDHAPCTGEVAAAAIRDVREVDCGIDESGIA
SVEADLPELADSVMDADELCCLIG
>BdbZIP45

MESRRGGGPAAAAAEDARGPMPGFGAPQHTIPTDVNIMQTSRVTDFGALAQSAGFRIEDLANLSANTLFNLKPNNHTFIS
DPLQFGNYGKSISPTDLATTAAAAAAAAITTVDP

QALLQQKGVQSNIVALRTRNSENWGESSMADTSPRTDTSTDPDIDVDERNQMFEQGHLAAPTGSDSSDKSRDQLDHKSLR
RLAQNREAARKSRLRKKAYIQNLETSRLKLTQLE

QELQRARQQGIFISSSGDQSHSTSGNGALAFDMEYARWLEEHNKHINELRAGVNAHAGDDDLRSIVDCIMAHYDEFFRLK
GVAAKADVFHVLSGMWKTPAERCFMWLGGFRSSE

LLKLLAGQLEPLTEQQLAGICNLQQSSQQAEDALSQGMEALQQSLAETLASGSLGPAGSSSNVANYMGQMAMAMGKLGTL
ENFLRQADNLRLQTLQQMQRILTTRQSARALLAI

SDYFSRLRALSSLWLARPRE
>BdbZIP46

MASQPGRGGGDAGTSQRGQMQNLARQGSLYSLTLDEVQNHLGEPLQSMNLDELLRTVFPDEADPDGATTSKYEPSAGLLR
QGSITMPSELSKKTVDEVWKGIQDAPKKNVQEGG

RRRRERQTTLGEMTLEDFLVKAGVVAEGYLKDLNDAGNGGFVGRGATAAGATELTSGAQWLDPYQQQIAVSAIESHQHMQ
QIMPGAYMHSPLQLVPQPLNVVTAAPAILDSAYS

DGHNTSPMVSPTSDSQTPGRKRGIPGDVPNKFVERRQKRMIKNRESAARSRARKQAYTNELENKVSRLEEENERLKKQKE
LDMLLCSVALPEPKYQLRRTCSAAF
>BdbZIP47

MASEMSKNLMASDDQEVTSQQRDQLSGGAAAAAEGQGEQQVVAPLERQPSSILERTLEELSYSLYDGRGLGSINMDEFVA
NIWNTEEFQAATGGLMADMENQAAVVGAAGGSGG

AGAGGSGTLCRQGSFSLPPPLSRKTVDEVWAEINDEGPLAHAQVPAFLPQAPPQPLAVQPPMGNGGGVAANGRQVTLGSM
TLEDFLVKAGVVRGGIAGQGQPPMPAGQLAHGPM

SGMQQGQVQPVGPMMYPMAPANAMYQMMGDGMGFQANGYADMAILPPPPPPSQGGVCILSPGSSDGISAMTNCFGSGSQA
MMMMDNGARKRSAPEDRSGGMSMERRHRRMIKNR

ESAARSRARRQAYTVELEAELDKLKEENARLKAQEGSILMAKQQKMENEMMEKSKENASAKKGGLRRSSSCTW
>BdbZIP48

MVQGEESSWRMASAHDHERAAVHLNQAALPYHGGVQAHASAALPASFLDFQPAAAAAAYFGELEEALIHGAANAGGVGHH
PGSMITSDMHSAKSAAAGYLAARPPTLEIFPSWP

MRHQQQLHSGNSQQSVGSTTDSSSARNTMAQMELVSPGPASSIRPSPPSSSEQQPQQQRQEVMMVTTDDYSYKPAFAPPP
ATLAAAPSFQQQLQLPLHGGDHDKRKHGLARKDG

KLVDSKTERRLAQNREAAKKSRLRKKAYVQNLETSRVRLQQMEQELQRARSQGTFLGGCSGSGDLSSGAAVFDMEYARWL
DDDGKRLAELRGALQAHLVDGNLGLIVEECMRHY

DELFGLKEELARSDVFHLLTGSWATPAERCFFWIGGFRPSDILKILIQQLDPLTEQQLMGIYGLKQSSEQAEEALAQGLQ
QLHQSLADTVAAGTLNEGAAVPNYMGLMAIALDK

LASLEGFYQQADNLRKQTLHQMRRILTTRQAARCFLSIGEYYRRLRALSNLWASRPRENFIGTDSLSPTGTELQGLHHQP
QQNQFSGF
>BdbZIP49

MSSVRYCLGRDFQPAAAAAAYFGELEEALIHGAANAGGVGHHPGSMITSDMHSAKSAAAGYLAARPPTLEIFPSWPMRHQ
QQLHSGNSQQSVGSTTDSSSARNTMAQMELVSPG

PASSIRPSPPSSSEQQPQQQRQEVMMVTTDDYSYKPAFAPPPATLAAAPSFQQQLQLPLHGGDHDKRKHGLARKDGKLVD
SKTERRLAQNREAAKKSRLRKKAYVQNLETSRVR

LQQMEQELQRARSQGTFLGGCSGSGDLSSGAAVFDMEYARWLDDDGKRLAELRGALQAHLVDGNLGLIVEECMRHYDELF
GLKEELARSDVFHLLTGSWATPAERCFFWIGGFR

PSDILKILIQQLDPLTEQQLMGIYGLKQSSEQAEEALAQGLQQLHQSLADTVAAGTLNEGAAVPNYMGLMAIALDKLASL
EGFYQQADNLRKQTLHQMRRILTTRQAARCFLSI

GEYYRRLRALSNLWASRPRENFIGTDSLSPTGTELQGLHHQPQQNQFSGF
>BdbZIP50

MASSRVMASSSSPSHTASDLSRFATAAAGRPGGSGLGSMNVEELLRGIYGDIPTPAPADRPMSPVRPAQETAARRTADEV
WKEITGGGSGEEEEVEVAPAGPAAAAVVPGAVGG

ASEMTLEDFLARESAAKEDAVRVSGPSAPLEEQVAMGFLNGPDGARGGGGGRGRKRQQMDPMDRAAMQRQKRMIKNRESA
ARSRERKQAYIAELESLVSQLEEENAHLSREQEE

QNEKRLKEAVQVLAKAMESRLISSGVRMAERLLCSEQVSISRMIC
>BdbZIP51

MASSRVMASSSSPSHTASDLSRFATAAAGRPGGSGLGSMNVEELLRGIYGDIPTPAPADRPMSPVRPAQETAARRTADEV
WKEITGGGSGEEEEVEVAPAGPAAAAVVPGAVGG

ASEMTLEDFLARESAAKEDAVRVSGPSAPLEEQVAMGFLNGPDGARGGGGGRGRKRQQMDPMDRAAMQRQKRMIKNRESA
ARSRERKQAYIAELESLVSQLEEENAHLSREQEE

QNEKRLKELKGKVTPVIIAKTSSQDLRRTNSMEW
>BdbZIP52

MEEEVIWKDMSSLSSTPVAGLQQHYYLSSPARSCFHRPPPPTALALSSTLEFTYLGCAAATASNSSSGDDAQLMPPAADL
DDILISFAAASNNHTGAGSANNNKRSTSAITAGG

GCERRQRRMIKNRESAARSRARKQAYTNELELELAQLRRDNQMLLKRHQDLNARLAMEAQVPDRSTLQRCRSAPAP
>BdbZIP53

MGSNDPSTPSKPSKVPEQEQPPATTSGTTAPVYPEWPGFQGYPAMPPHGFFPPPVAAGQAHPYMWGAQHMVPPYGTPPPP
YMMYPPGTVYAHPSTPGVHPFHYPVPTNGNLDPA

GAAPGASEINGKNEPGKTSGPSANGITSNSESGSDSESEGSDANSQNDSHSKENDVNENVQSGAVIGGVAGPATNLNIGM
DYWGATGSSPLPAMRGKVPSGSVRGEQWDERELK

KQKRKLSNRESARRSRLRKQAECEELGQRAEVLKSENSSLRAELERVKKEYEELRLKNASLKEKLGEAGDSVPDMNEQSD
TNGGSHQQKEA
>BdbZIP54

MASSSGSGSATTTGSLSPSMAAVAGASEEEMRALMEQRRAKRMLSNRESARRSRMRKQRHLDDLAAQAAHLRRENAHVAA
ALGLTARGLLAVDAENAVLRTQAAELAARLASLN

DILSCIGMSAANNNSSGSSGCGGSNSNSNSNTAAAAAVALTVAAAAAATGGDPLLLGGFDGALDDMFRSSPDLFMFQPC
>BdbZIP55

MKKCASELELEAFIRGRGAAAAAVAEQKPGHAAAAAAGTHGPFGVFSAADLAGFGFADSNTLNGGIHNHLWSQSPNLGAR
LPAVSTTIDSQSSIYAAASPTSATNLSMKENQGF

GGTSGSDSDSESMFDMEGGLCDQSTNPTDVKRMRRMVSNRESARRSRKRKQAHLVELETQVDQLRGDNASIFKQLTDANQ
QFTTAVTDNRILKSDVEALRAKVKLAEKMVSQGA

LSCGLGHLGLSPAALVPDGLAGLDFLPGGGADDACFASLSPAEQVQGSPMQSMASLESLDHGGRMPGGGDVWGWDSNSNG
ALSK

>BdbZIP56

MFDMEGGLCDQSTNPTDVKRMRRMVSNRESARRSRKRKQAHLVELETQVDQLRGDNASIFKQLTDANQQFTTAVTDNRIL
KSDVEALRAKVKLAEKMVSQGALSCGLGHLGLSP

AALVPDGLAGLDFLPGGGADDACFASLSPAEQVQGSPMQSMASLESLDHGGRMPGGGDVWGWDSNSNGALSK
>BdbZIP57

MATHDEAVAADIICSLRGADLAGWTPPWSKRVEAEAAAAGELSWPAVARGKRSRRRSPSSAPAPEGKKGRRCAARSSPPS
PLEYSAGSASLRSGASTSGGEDGGSGFCSPWHRR

AAPATTKVESIRRPQLTFPAPPLRPTGQRQRKKMRLPEVQQLVRSLTAENGSLHEEMRALQRACSALAKENDKLEARVEQ
RSNSQNAIALKEEKGKQQIDQQPPNDSFALPDLN

LPAQDNADAVHC
>BdbZIP58

MQRQGAACGIAAYHHSGVFLPRQDYDDPLADVFASLSDMIAPPPYGFNGGEGVGSAAPGGDERKKRRLASNRESARRSRV
RKQRRLDELASRAARLRAANRRLLVELNRVAAEH

GRVARESARLREEASELRKKLDEMGMDSMEETQSAASASASAGSAEAEHLQE
>BdbZIP59

MAELDQALFLYLNSQDQTSVQEQPQTLNIFPSRPMHVVVEPSPRAAANPPPNVAAGSSKQQPSRPAAPAPGKNGKAAVKR
EGSGGGGGTPSTSEQEGPRTPNAKTLRRLAQNRE

AARKSRLRKKAYIQNLETSRIRLSQMEQEMQRCSAQVWLLHGFVDLRQPLWLAGPVGVGFEARCVRHGRAEAAWFDGEHA
RWVEEHERMMRHLRAAVELDDNNLQHQQGHQDDG

GQLRQLVDAAAAHHVVLAELKSAVARADVFHLVSGTWLPAAERCFLWIGGSRPSDLVKVRIFALITS
>BdbZIP60

MQEQGESSRPSSSERSSSSGGHHMEPKEGMESDDEIGMVPELGLEPGGPSTSGRAGGPERAGQSSTAQGSARRRGRTPAD
KEHKRLKRLLRNRVSAQQARERKKAYLGDLEVKV

KDLEKKNSELEERHSTLQNENQMLRQILKNTTVSRRGPSEGQ
>BdbZIP61

MAQLPPRAPSAAGQQDWSAAGEFLGFAASRRGAHRRSASDSAAFLEAVPMDDDVIGGGEFDRLDDEQLMSMFSDVDAPAV
SSDGGGGGFMDMGDGEEGSAGARAMAAAEGGYGD

PKRVKRILANRQSAQRSRVRKLQYISELERSVTGLQMEVSALSPRVAFLDHQRSLLTVGNSHLRQRIAALAQDKIFKDAH
QEALKEEIERLRQVYHQQQIKATGGADIATAASM

QAKQELLACEGAAMR
>BdbZIP62

MPTVAESSSSMGAIYERRHQHHPLHGAWAGYMPSPLSGPPGVHEAAAAAVRAMDLTDPKFEPPSQPLEQQEQEEEEDHHA
LALPLPESPDSSDHDPARPRDKIRKTRRLAQNRE

AARKSRLRKKAYIQNLETSRMKLARMEQELAMARQQHVLCFGRAGTSTSSPVGRLPLPPSFNPGVAAFEIEYARWVEEQG
RQTAELRAALQLLQPDPTRLRLLAEAALAHYDRL

FEAKSAAARRDVFFVMSGAWRSPAERFFLWISGFRPSDLLAVLSPHLQTELHDADHSPALAPALTEAQAEEVARLRRTSR
QAEDALFHGLVTLRQALAESLLAPAMAATAETQQ

EVSFDSGYGGGDGGEMGGAMGRLEELAGFVEQADHLRQQTLRNMYRILTPTQAARGLLALGEYFHRLRSLSELWVKRPRE
PA

>BdbZIP63

MSLSGGTLSSETLSGSSHGTQSYGSEGNLELQARMDLKRKRRKESNRESAKRSRLRKQQQLEELTTQVNQLRTEKQQLVT
TLNLTVQSYAAAETQNSVLRSQAMELESRLRALR

EIIYYMNSASTQFRIPTAASQTTAAAYYPTTSLMASAAASYDVAGANAWGSGMQMLQQQQPIELMYHRC
>BdbZIP64

MAPAHGSKQRAGLPPPAPPSSGSGGSHPQLHADACVVQMQQQEGQLLPLRKAHRRSRSDVPFGYFPPPSPKTESGSWAGA
GGDDLFSAYMSMEGMDSAGLSNNNSDGEYSRGSS

VPAAGNGADSSENESEDYGGGGEGQVFLWGGDAGGSGGKKRNAAGETAALAGRHARSLSMDSLMGRLSFSGANGNGEPGK
LFSLEFGSGEFTPAEMKRIMADEKLAEMALADPK

RVKRVLANRQSAARSKERRMRYIAELEHKVQILQTEATTLSAQLTHLQRDTSGLATQNNELKFRLQAMEQQAQLRDALNE
ALTGEVQRLKLSATTSELGDGGSSSNLAQKMQLR

CQNQMLELHKQQQQQIPFYQLEQPEQNGAASRTHESK
>BdbZIP65

MDLGDGGESERKGAAPPMPLARQGSVYSLTFDEFQSTLGGASGGGGLGKDFGSMNMDELLRSIWTAEESQAMASASAAPA
GELQRQGSLTLPRTLSIKTVDEVWRDFVRDASPG

AAGGGEPLPKRQPTLGEMTLEDFLVRAGVVRENPAAAAAVDAAVPPPLAARPIQVVNNGSMFFENFGGANGASGASAMGF
APVGIGDPSHPTMGNGMMPGVAGMGVGAVTVGPL

DTSMGQLDSVGKVNGELSSPVEPVPYPFEGVIRGRRSGGHVEKVVERRQRRMIKNRESAARSRARKQAYTMELEAEVQKL
KEQNEELQKKQEEMLEMQKNKALEVINNPYGHKK

RCLRRTLTGPW
>BdbZIP66

MYHHNHGEVASLHFLLPPNPSSSFTTHHHMNMALPPQAYFPPSFEPPALLTGDDTAAAFEFETILEEAAHLVSGNGSPSS
GSDGGGGNCYYQEGSAGAGAAAAEERRRRRMVSN

RESARRSRMRKQRQLSELWARVAHLRSANRRLLDELNRALRACADACRESARLRDEKTKLTEKLEQLLKQPAPEKTASSS
AASSNRSSCSSEPCKNSATAADGVTE
>BdbZIP67

MESKSPVPDGGGGGGGNGLPPKPSRRDGAAPPESDMSHMPESPRRAMGHRRAHSETIGGLPDDLDLGVPGGGGGGEGRASL
SDENEEELFSMFLDAEKLNASEAESSSCAPTGVG

ARPRHHQHSHSMDASSSFDAEQLLGAAAAVEGMSTAEAKKAMSNAKLAELALVDPKKAKRIWANRQSAARSKERKMRYIS
ELERKVQTLHAEATTLSTQLALLHRDTAGLSTEN

SELKMRLQNVEQQVHLQDALNDALKSELQRLRMATGQMGSNVGGMMNFMGPPLPQPFGGNQTMFHIQSQTAMQPLHHMQI
HPHHQQQQQQALLHPLQLQAQQQLLGQQHAAAPP

NRR
>BdbZIP68

MESKSPVPDGGGGGGGNGLPPKPSRRDGAAPPESDMSHMPESPRRAMGHRRAHSETIGGLPDDLDLGVPGGGGGGEGRASL
SDENEEELFSMFLDAEKLNASEAESSSCAPTGVG

ARPRHHQHSHSMDASSSFDAEQLLGAAAAVEGMSTAEAKKAMSNAKLAELALVDPKKAKRIWANRQSAARSKERKMRYIS
ELERKVQTLHAEATTLSTQLALLHRDTAGLSTEN

SELKMRLQNVEQQVHLQDERCYHLIRLQKKVDALFHNLI

>BdbZIP69

MDDSGGADPARQHQHPYHQQQQQLASPQQGGAAPVPRSPTPLDLAAYHRRLSASPRPPAHPQAPCARLPSPYGGGGGHQI
PSGGGHARSLSQPLFFSLDSLPPPPYADLAAARS

PPSSTSDPPPFGLPPRGHRRSHSDIPFGGFSQLSPPLPPPAPVKRELPSAPEGGRSGDDAALYDLVNAYMDLDGMDALNS
DDRDDSRASGTRAGSAAESSENEAESQSTSAQRK

DGGGKSRHCRSLSMDSFMGKLNFAAGDESPKLPLASSSGGGLSRSGSGSLDGGGAAALFGTEFANGEFSEAEKKKIMANE
RLAEIALTDPKRVKRILANRQSAARSKERKMRYI

QELEHKVQVLQTEATTLSAQLTMLQRDSGGLATQNNELKIRLQAMEQQAQLRDALNEALTGEVQRLKLATGETSDARMSK
MGLQQQMNSQLIQMQQLQIQQQQQQQPSQTRQAQ

QQQQQQSQQSS
>BdbZIP70

MSRSPHLPPRCPQLGPQITRRDDSLFTQSCRFPSDDPFFGEPPCWLDDLLADSGKTPNLPPLRRACSDSDAILDALTIFK
LPIFPSEEGDVQPASESGDSFDAVAGGESGSAVE

GSCVYGPNSPRQKSRLTSSESSMVNAVLENVPNNPLQYLTIDPSSTLHGNVANGTGDAYDDVNHLDQDKSFKRRSGQRSR
VRKLQYIADLERTVDSLQNMGADLALRVASHFQL

RNALSMENKQLRIQIASLQQAKLVKDGQTLFLKKETERLKQISAGHRRSRSVTSCFDTDSFGADPSAINWQMLDMSKLSL
NGSPVPVPPRGGYGL
>BdbZIP71

MYPAELASIPYLSSASAASFKPHYQVATNDILFQYNSLPVPQAISYQHVEHLVHEASLPVGNKSNSDESDDYQHSLAEER
RKRRMLSNRESARRSRMRKQKQLSELWAQVVHLR

STNRQLLDQLNHVIRDCDRILHDNSKLRAEQAELKQQLEKLPVENMESSVMGPGMT
>BdbZIP72

MDFPGGSGRQQNQQQPLPPMTPLPLGRQGSSVYSLTFDEFQSALGGPGKDFGSMNMDELLRNIWTAEESQALGAVVANAS
SSSAAAGADQGAGAQPIQRQGSLTLPRTMSQKTV

DEVWRDMVYFGGPSAAPAAAELPPAQRQQTLGEVTLEEFLVRAGVVREDMAGPPPPVSPAPAAQAQQPPPPQPQMLFPQS
NMFAPMVNPLSLGNGMMAGAFGQGGGGATTAVSP

ARPVLSNGFGKMEGLNLSSLSPPPMPYVFNGGLRGRKAPAMEKVVERRQRRMIKNRESAARSRQRKQSYMMELETEVAKL
KERNEELQKNQVEMLERQKNEVFENIRRQVGPKS

KRICLRRTLTGPW
>BdbZIP73

MSREEAGGSPGQLSLSSFSSLFSVSSATSTPTPHLPPLPSLSLSIGGGGNDEQPPLSSSSAVNQEEDEQRSVRMMKNRES
ALRSRARKRAYVQELEKEVSRLVDHNLKLKRQCK

QLKTEMAALVQAQQQPSKSPQYRRTPSSSTHL
>BdbZIP74

MSALEMPVLPPFFPGPDDIDAFLADLGFDDDHEAPTPPPALCTSPEEDQTSAGSAGTVGAATDAGAGGDRERLRRLRRRI
SNRESARRSRTRKQRHLEEQRATEAALRAGNRDL

AARLRSARARAAAAHVANARLRSEAHALSRRLAAARRALALRLQYLSSVSVSGAGGLFDMQQTAAAMQQDAFFSFSC
>BdbZIP75

MEFGADEVVEVNCARGGSGGGDPGVYAAVLKRKLDLYCAAVAKTMEAKPQESALGAMQLVSQASDTSQLVSQASFDGDGT

VVQGKPANSCTSREQSDVDGDLEENTDPANAKRV

KRMLSNRESARRSRKRKQAHQTDIESQVTQLRAENASLLKRLTDMTQKYKEATLGNRNLTVDMETMRRKVNIAEEAVRRV
TGASLLFSITSDMAGSSVPFSSCISDAASADAAP

TEESMSHLLQGFFEDDQIKPDLPQATTPVVPSGEEMASRPASLRRIASLENLQQRIHGDSIHSDTASAFSDHEFPENAL
>BdbZIP76

MSSSSLSPGGRLSGSDGDSGGAPMGGGGGDKRREKRRLSNRESARRSRLRKQQHLDELVQEVARLKAENARVLGRANDIA
GQYVRVEQENTVLRARAAELGDRLRSVNQVLRVV

EDFSGVAMDIQEECPPDDPLLRPWQIPYPATAMPIAATATHMLQY
>BdbZIP77

MMMANAKLQKQALLPPRSPFPTAASPYGDHGPIARPQGQGAATHHRFGHGHGHGHHQRTSSESIIEEQPSWLDDLLEEPE
TPVRRAGHRRSSSDSFALFDGSAASGAYANSFEE

MGGGGQAAPWGGLPEYYAKPSLYGRPQGRPWEQGMPNLAGYRPGPPMPLREKVGAHHGPPNMFRDHDHVLDRRAPDEGGY
DQKVGAERKEGVLPKHAQPQSDADTKRAKQQYAQ

RSRVRKLQYIAELEGKVQSLQSEGIEVSAEMEFLTQQNMMLDLENKALKQRLESIAKEQVIKRVQQEMFEREIGRLRSLY
QQQQQQQQQPQPSALARSNSRDLDSQFANLSLKH

KDPNAGRDAVSGPLRT
>BdbZIP78

MDPRFPPPAPAGGGGERGHHRRAQSETFIRFPDAADLLLDPDSDDFSFSDLDFPSLSDDSAPAASSAAPPQGLPPTHGGT
VSSSSYAPRPPGVCGGGAHVRSLSLDAAFFDGLQ

LQGGGGGGGLAGHKRSGSMDGASSPSEGESALSGGALPDYAKKAIPAERLAELALLDPRRAKRILANRQSAARSKERKIK
YTGELERKVQTLQTEATTLSAQLTLLQRDTSSLT

VENRELKLRLQSMEEQAKLRDALNDALREEVQRLKIAAGQAPNMHGNPFNGGQQQMQQQQMPNYFSQQQQMQMHYLGGHQ
GQHQNHQNHQQSSSNGGQSLSGQSLSDSMDFM
>BdbZIP79

MGFWPLFGGDLLLLCRLQPLSALSKRQRAQGDGPQVLNQFVGPAVIDKTDLNQPLGPAVTEKAAPQKRAVDSVLNCRCLP
GPPARSRGDQQASKQQRRRTVHPPPLAFTPLLFF

PNQPHELKLKLTAAATRRPRSPTFFPIEASNPEHKPSPVSGSMDPRFPPPAPAGGGGERGHHRRAQSETFIRFPDAADLL
LDPDSDDFSFSDLDFPSLSDDSAPAASSAAPPQG

LPPTHGGTVSSSSYAPRPPGVCGGGAHVRSLSLDAAFFDGLQLQGGGGGGGLAGHKRSGSMDGASSPSEGESALSGGALP
DYAKKAIPAERLAELALLDPRRAKRILANRQSAA

RSKERKIKYTGELERKVQTLQTEATTLSAQLTLLQRDTSSLTVENRELKLRLQSMEEQAKLRDALNDALREEVQRLKIAA
GQAPNMHGNPFNGGQQQMQQQQMPNYFSQQQQMQ

MHYLGGHQGQHQNHQNHQQSSSNGGQSLSGQSLSDSMDFM
>BdbZIP80

MTMMPSYQSSLSLSLWAGWLAPCKQAQAQLIKGNKFQGNFQLHCHTFHAGTTATGAAAARPPPTLDIFPTWTIRPLAPPH
HHTPKEGSNLTADSTDSESSSKNNNIKHSPDHQK

VQAVSMAMASQFHQISQQQQNHQQQQHHHQQKMATSSTHSDRTGKALDPNKIMRRLAQNREAARKSRLRKKAYIQQLESG
KIRLAQLELDLNRARSQGLLLGGAPGGNCTADAA

MFDAEYSRWLDDDSRRMIELRGGLHAHLPDSDLRAIVDDALTHYNELFRLKDTAARTDVFHLITGMWATPAERCFLWIGG

FRPSDMLKTLVPQLDPLTEQQVSGICSLRQSLQQ

AEEALTQGLEQLHQSLADTVAGSGSLTDDTNMGSFLGDMALALGKLSNLENFVIQTLHQMHRILTVRQAARCFLAIGEYH
NRLRALSSLWASRPREIMMADEGNCGELSIAAHP

SESQYAAF
>BdbZIP81

MGGFRAEDHHHNQPLELPLGFRTASPPPMIASSSMSKESTSYDMADFDQAAIFLYLDGHDQQSIQEQRQTLNIFPSQPMH
AAEQLAAAKINGGAAMAAMMLPNGGNPQQPSPRR

PEQHQQGAAGGLNASPASLLPNSAKENKNSSTNLIKKEGTSSGKGATSSSTDQEREAGVRRTQDPKTLRRLAQNREAARK
SRLRKKAYIQQLETSRIRLSQIEQQVQAARVQGV

LLGTTGDQHHQLQGLPNSAPSVAGMFDAEYGRWVEEHGKLIFQLRAALNEHVPDCNQLQALVGAAMAQHDELLNLKAAIA
RADIFHLLCGVWASPAERCFLWLGGFRPSDVIKV

MLKHVEPLSEAQLLGIYNLQQGVQETEEALNQGMESLQHSLSDTVAAPEVSAGNFMGHMSLALNKIASMEAIVRQADSLR
QQTLQKLHQTLTIRQAARCLVAIADYFHRLRAIS

TLWAARPRHDQQGPHP
>BdbZIP82

MSSGTSFGSSLGTRSSRSEEDDMDLQAQMEKRRKRRKESNRESARRSRVRKQQHLDDLSSQVDQLKNQKQQLGMALGVTT
QNLVAVQTQNSVMQIQKLELESRLCALREITCCM

NSIRNTTNPATMGITTGSGYDIFGTSSTWSQPLDLYQ
>BdbZIP83

MLTEDRWGGTEGIMEFKTGGSSSEHRPSVGGSTPLARQGSVYSLTFEEFQSTLGGGGLGKDFSSMNMDELLRSIWTTEER
QAMASASASASASAAGAGAGTPPTSLQRQGSLTL

PRTLSAKTVDEVWRNLVRDDPLAIGADGGEPQPHRQATLGEMTLEEFLVKAGVVREIPNAPPHPVPVAPKNTAFYRNFPG
ANDAGAAMLGFPPSGMGDLALCNGLVPRAVGVGG

NAGAVQTAANQLDSDSKGSEDLSSPSEPMPYSFDGIVRGRRAGGGVEKVVERRQRRMIKNRESAARSRARKQAYTMELEA
EVQKLKELNQKLEREQAEIMEMQKNEDVPEMKDQ

FGRKKRQCLRRTLTGPW
>BdbZIP84

MLHHYHGEVASLHCLTPPNPPLFHTHYQSSMITMTPPSFQFSAAAYDDDEPVQEALAAAISNNSSARSGGAAGDTDIHGR
AAAEEEERRRRRMVSNRESARRSRVRKQRQLSEL

WAQVLHLRGANRRLLDELNQAMRGRDDVRRENDRLSDEKAELEARLQQYLMQAQENNNTPSSSSSYKITATATE
>BdbZIP85

MMSAAGSQSQQQEIIRQHQQQQQEMEMNISFGMSSQHHSLHHHAPPSSSSSSSMHAAAAASFMSAKEAAAASGAYDQLGD
LDQALFMYLDHGHGSQHASAHHQEQRQTLNIFPS

QPMHVEPSPKGEISLAVLSPAPVGSKMPRSSPDHHQHQHQLQQAAMEELAGSRRQQEHHLAVQQQHQQPFAAEPAGVSKD
VKPLAKKDKRGLSTSERDPKTLRRLAQNREAARK

SRLRKKAYIQQLESSRIRLAQIEQELHSARAQGVFFPGSGLLTEQGVTGKGLGGIDGLSSEAAMFDVEYGRWQEEHYRVM
YELRAALQQQLPEGELQMYVENCLAHHDEVVAIK

DAVIKGDVFHLTSGVWRSPAERCFLWLGGFRPSEVIKMVLGHVEPLAEQQIVAVYGLQQSAAETEEALSQGLDALYQSLS
DTVVSDALSCPPANVANYMGQMHVAMNKLSTLEG

FVRQAENLRQQTLHRLHQVLTTRQMARSLLAVSDYFHRLRTLSSLWVTRPRPPPQDHQQPGDGAGAGGTPHSS
>BdbZIP86

MNKDRAWISAEGGGGDGLPPQSSRRAGPSSSTTPPPEYDISRMPDFPTRNPGHRRAHSEILSLPDDLDLSAPGGGDGPSL
SDENDEELFSMFLDVDKLNSSCGASSEAESSSAA

AGGGQAAGMGHGMRPKHQHSQSMDESMSIKTEELVGAQGMEGMSSVEAKKAVSAAKLAELALVDPKRAKRIWANRQSAAR
SKERKMRYIGELEHKVQTLQTEATTLSAQLALLQ

RDTTGLTSENSELKIRLQTMEQQVHLQDALNDTLKTEVQRLKVATGQLANGGGMMMNYGGMPQAPHQFGGNQQMFHNNQT
MQSLLATHQLQQLQLHQQPQQQALRPQHQQQPLH

PLQAQQLQRDLKMKGPMGGGQSLWGDGKSSGGSSGI
>BdbZIP87

MDDDPVRRRRLLALPGPRAPADSYGNRVPPSFLLPPVHPAPVRFGQGAFGYYQAPPLPPASAGGGGAGSSHHARSLSQPQ
LSSMDFLFGPNSYANPAAPTPIAFAPPQPPPANS

PSGLPPLRAGHRRSQSDFQLGFSQPNPQMPPPAPVNPQTPAPEGRESVTANKNKTLADGPLGSRKSPKGLDNVAGSSADG
AQERRDQVDSQARGPRAWSPADSSDNETESADGS

VPRHGRSLSADSFVGKLTFGSVGLVSSNLPPSSPGKEAPGKLARSGSGSIGGAAALVATDIAIGGFSEADKKKIMENERL
AEIVLTDPKRVKRILNNRVSAAKSKERKVRYMSE

LERKVQVLQKETATLTGQVAMIQRDHSVLSTHNNELKIRLRAMEQQAQLRDALSETLNSEVQRLKLAAGEISDPHVLNGS
QQQMSSQMIQLQQLQILKQSSETQQAQQQPQHSV

QPGTQQQQWNV
>BdbZIP88

MAAMEDDEDMWAVTTSPSASPPATAAAAAISTALSLNPRLHLLASSSSPFHPGFGGCSRNAAAASPPCFFSAAAASSFPH
FAAAPLDGAARGMCGLAPVPAWSGAAPTGTGAGP

VERRKKRMIKNRESASRSRARKQAHVTQLESEVRELQLENDELRIKYDQLKASVEAPVPVKRTLKRVLSAPF
>BdbZIP89

MGSSGADTPTKASKASTPQEQQPPATSSAATPVVYPDWTNFQGYPPIPPHGFFPSPVVSSPQGHPYMWGAQPMMQPYGTP
PYVMYPPGGIYAHPSMPPGAHPFAPYAMASANGN

ADATGTATAAAPSAGETDGKSSEGKEKSPIKSSKGSLGSLNMITGKNCVEHGKTSGASANGAISQSGESGSESSSEGSEP
NSQNDSHHKESGQEQDGEIRSSQNGVSRSPSQAK

LKQTMAIMPMPSSGSMPGPTTNLNIGMDYWANTASSPPAAHGKATPTAVPGTAVPTEPWMQDERELKRQRRKQSNRDSAR
RSRLRKQAECEELAQRAEVLKQENATLRDEVNRV

RKEYDELISKNNSLKDKLGDKEHKTDDAELDNKPQRSGDDSQKKETN
>BdbZIP90

MANGRLQKQALLPPRIPFPVAAAAASPSPQAELGPIARPRDAHHRQGHQRTSSESVLVDEQPSWLDDLLDEPDSPARPHG
RPGHRRSSSDSFTLFDGAAAAAAASSAGTYDNVF

DGIRGGGQPSSWGRAPEFFPEQNSFGRPQPQPQPQGLPWDPRQMFLQGVGMPLPVREKNVMHHGAVLNGVDMKGHVDSAH
DQMIGTERNEGDAHLRHSQSEADTKRAKQQYAQR

SRVRKLQYIAELERRVQALQTQGIEVSAEMDFLGQQNIMLDLENKSLKQRLESLSQEHVIKRVQQEMFEREIGRLRSLFQ
QQQQQQQHILQQQAPTHSRSNSRDLDSQFANMSL

KHNDPNSGRDAVPGLRI
>BdbZIP91

MDPRYQLPTPVPSSGVGGGARGHHRRAHSETFLRFPDAELLLDPDGDFSFSDLDFPSLSDDSPAASDPTPPPPPPLASSG
APAAAPRPPGGAHHNRSLSLDAAFFEGLAIQGGG

GHKRSGSMDGVNSPFEGESALSGGLPDYAKKAMPAERIAELALLDPKRAKRILANRQSAARSKERKIKYTGELERKVQTL
QTEATTLSAQLTLLQRDTSGLTAENRELKLRLQS

MEEQAKLRDALNDALREEVQRLKIAAGQASNMNGNPFNSGMQQIPSYFSQQQQQQQHQQQQQMAYLGGHQAQNRHPNHHQ
SPSNGGQSLSGQSLNDSMDFI
>BdbZIP92

MAGSSQRQCRRGVSAGGGDEDVELDAAMALADMAGAVAGAPPQPQAERSGAHGESEEEEASTRLSLELGRVGIQGAAAAS
SPCSSSSSAGGCPTHQQALPAVAAPGAGYGARPR

HSTLTEAEKEAKRLRRVLANRESARQTILRRQAIRDELARKVADLSSQNESMKKEKETVVAKTADRSPSPVPIAMPTGTA
EAPAQRAEASQQAAAPPPPPQPGFLYATAGPSSA

PVPVPYVWGTWPPGYEPPAAGAPPPPLCLPPMGAWYYPAPPSYAAQQSFAGAGGSPVGSGGGGTAGEEEDDTDDGADPCS
LTLGLEVGGNGGGGGRADKAATAAEARKRRKELT

RMKHARPGGDE
>BdbZIP93

MSSQGGSTAVMGKGHHRAHIQTLVREGSLYNLTLSEVESHLGAPLLSMNLDDLVRSVLPDDTSLPIRNGVGNSGSQNTPS
SGLERQGSSITVPPALSKKTVDEVWRDIQQDQES

SDDEERSSGCEAQLSFGEMTLEEFLHRVGIVSEQHQKDADELSGRVGTGEDSNLMTKVQDFPQGTSPIDAFIIRQSIAQP
LSVAIPSTMDAIYPDGQMSISPSVALSDLQTPTR

KRISSEDVVYKVVDRRQKRMIKNRESAARSRARKQAYTNELECKLSCLEEENKRLKREKELDRLLKSAPPPPEPKPLLRR
ARSASF
>BdbZIP94

MDVPSASVSTSAAAGSASRDVAAMPDTPPRRAARHRRAQSEILLGATALPDDLTFDADLGVVGEGSGGGGDNYDDEDDYE
EDEEGSGGGGGGSRMFEMFLESGGGLAEPLEATP

YPPPPPSRPRHQHSMSMDGSTSLAASSSAMAGRAGADAKKAISDAKLAELSLVDPKRAKRILANRQSAARSKERKMRYIA
ELERKVQTLQTEATTLAAQLSMLQIDTTGLTSEN

GDLKLRLQTIEQQVRMQDALNDRLRDEVQQLKIATGQVNASSGKMGNFGLSSYGSNPQSYQRSHVQSLLAAQQLQQLQIH
HSQQQQHQMQLQDQQHLATVQRQRQQQLLQEAMP

FRGDLKMKGIAMTSHVQNAAAFDAHARSEP
>BdbZIP95

MDVPSASVSTSAAAGSASRDVAAMPDTPPRRAARHRRAQSEILLGATALPDDLTFDADLGVVGEGSGGGGDNYDDEDDYE
EDEEGSGGGGGGSRMFEMFLESGGGLAEPLEATP

YPPPPPSRPRHQHSMSMDGSTSLAASSSAMAGRAGADAKKAISDAKLAELSLVDPKRAKRISIAEVDRDVLPLEKEAHEC
GLIDKTEFADEDYLHSFSIASDANKKYAIVFCVL

NLTSRPLGILANRQSAARSKERKMRYIAELERKVQTLQTEATTLAAQLSMLQIDTTGLTSENGDLKLRLQTIEQQVRMQD
ALNDRLRDEVQQLKIATGQVNASSGKMGNFGLSS

YGSNPQSYQRSHVQSLLAAQQLQQLQIHHSQQQQHQMQLQDQQHLATVQRQRQQQLLQEAMPFRGDLKMKGIAMTSHVQN
AAAFDAHARSEP

>BdbZIP96

MTSASAQFAPAPLRMGMYERPPPQQPMPPSPQPVVGMWNNSVDTYKVDSGQATSGSTIMEADTKFDDAELEEVPGMEELE
PTRDVDQEASKPPDKVLRRLAQNREAARKSRLRK

KAYIQQLETSRIKLAQLEQELQRTRQQQGLYPPGHSGMAGFGGVGGVPMDSGVAAFEIEYGHWVDEQNRHTRELRGALQP
GQQTTELELRMLVETGLGNYDHLFRIKNLAASAD

VFCVMYGLWRSPAERFFLWIGGFRPSEVLKVLRPQLEPLTDQQLEQVYHLQQSSTQAEDALSQGMERLQQTLADALTAAA
DPFASPDGYSGMNDAIGKLKGLVCFLHQADHLRL

ETLQQMQKILTTRQAARGLLALGDYFERLRALSSLWAARPRESAIS

Table S8 Homogous bZIPs in rice and Arabidopsis

BdbZIPs	logous Osl	Comments	SCORE	E-VALUE	ologous Atb	Comments	SCORE	E-VALUE
	OsbZIP33		518	0				
BdbZIP2	OsbZIP58	RISBZ1.	218	8.00E-68				
	OsbZIP63		595	0	AtbZIP45	TGA6, involve in the activation of SA-responsive genes.	482	6.00E-173
	OsbZIP28		569	0	AtbZIP20	AHBP-1B, TGA2.	480	3.00E-172
	OsbZIP08		522	0				
BdbZIP5	OsbZIP03	HBP 1b(c38).	500	3.00E-180				
BdbZIP6	OsbZIP62		221	1.00E-72				
	OsbZIP61		463	8.00E-165	AtbZIP18		251	4.00E-82
BdbZIP7	OsbZIP30	RF2b, Insertion element IS2A.	358	3.00E-124	AtbZIP52		216	1.00E-68
	OsbZIP60		632	0	AtbZIP17	regulate transcription as part of a salt and osmotic stress respon	258	2.00E-75
	OsbZIP39		308	1.00E-93	AtbZIP28	Up-regulated in response to heat.	248	2.00E-72
BdbZIP8					AtbZIP49	regulate transcription as part of a salt and osmotic stress respon	244	2.00E-71
	OsbZIP60		656	0	AtbZIP17	regulate transcription as part of a salt and osmotic stress respon	269	2.00E-82
	OsbZIP39		328	2.00E-105	AtbZIP28	Up-regulated in response to heat.	255	1.00E-77
BdbZIP9					AtbZIP49	regulate transcription as part of a salt and osmotic stress respon	252	7.00E-77
	OsbZIP55		217	3.00E-74				
BdbZIP10	OsbZIP56		217	3.00E-74				
	OsbZIP53		404	2.00E-144	AtbZIP23	involved in the adaptation to zinc deficiency. Binds ZDRE mot	214	3.00E-70
BdbZIP11					AtbZIP19	involved in the adaptation to zinc deficiency. Binds ZDRE mot	211	4.00E-69
	OsbZIP52	RISBZ5.	260	2.00E-87				
	OsbZIP15		202	4.00E-65				
BdbZIP13	OsbZIP20	Transcriptional activator protein.	200	8.00E-64				
BdbZIP15	OsbZIP50		309	3.00E-109				
	OsbZIP47		524	0	AtbZIP46	PAN, PERIANTHIA, TGA8.	390	5.00E-135
	OsbZIP8		427	5.00E-149				
	OsbZIP28		410	2.00E-144				
	OsbZIP03	HBP 1b(c38).	401	5.00E-141				
BdbZIP18	OsbZIP63		398	1.00E-139				
	OsbZIP46		413	3.00E-146				
	OsbZIP23		349	9.00E-121				

	OsbZIP72	ABI5.	258	8.00E-85				
BdbZIP19	OsbZIP66	ABI5.	224	3.00E-72				
BdbZIP20	OsbZIP59	Putative Em protein.	436	1.00E-153				
	OsbZIP30	RF2b, Insertion element IS2A.	481	2.00E-172	AtbZIP18		294	1.00E-98
	OsbZIP61		414	3.00E-145	AtbZIP52		228	2.00E-73
					AtbZIP59		222	2.00E-70
BdbZIP22					AtbZIP69		219	6.00E-69
BdbZIP23	OsbZIP29		215	9.00E-72				
	OsbZIP28		596	0	AtbZIP20	AHBP-1B, TGA2.	486	6.00E-175
	OsbZIP63		579	0	AtbZIP45	TGA6, involve in the activation of SA-responsive genes.	485	2.00E-174
	OsbZIP08		523	0				
BdbZIP24	OsbZIP03	HBP 1b(c38).	489	4.00E-176				
	OsbZIP26		593	0	AtbZIP16		268	2.00E-87
BdbZIP26	OsbZIP86		223	3.00E-70	AtbZIP68		216	9.00E-68
	OsbZIP25		372	5.00E-127	AtbZIP29		260	3.00E-82
	OsbZIP78		333	3.00E-112	AtbZIP30		240	3.00E-75
BdbZIP27	OsbZIP68		258	1.00E-82				
	OsbZIP02		270	6.00E-91	AtbZIP64	Involved in phyB signaling pathway.	246	7.00E-82
	OsbZIP06	Putative transcription activator RF2a	239	8.00E-80	AtbZIP34	Forms heterodimers with the related protein AtbZIP61.	206	3.00E-25
BdbZIP29	OsbZIP19		221	1.00E-72				
	OsbZIP03	HBP 1b(c38).	575	0	AtbZIP45	TGA6, involve in the activation of SA-responsive genes.	485	1.00E-174
	OsbZIP08		530	0	AtbZIP20	AHBP-1B, TGA2.	471	4.00E-169
	OsbZIP63		495	2.00E-178				
BdbZIP30	OsbZIP28		487	4.00E-175				
	OsbZIP45		584	0	AtbZIP55	induced by cold and water deprivation.	211	5.00E-66
BdbZIP31	OsbZIP05	Salt stress inducible bZIP protein.	389	2.00E-135				
BdbZIP32	OsbZIP44		225	2.00E-75				
BdbZIP33	OsbZIP44		225	2.00E-74				
	OsbZIP08		395	2.00E-137	AtbZIP20	AHBP-1B, TGA2.	356	5.00E-124
	OsbZIP43		372	9.00E-131				
	OsbZIP03	HBP 1b(c38).	367	2.00E-128				
	OsbZIP63		365	1.00E-127				

BdbZIP34	OsbZIP28		365	2.00E-127				
	OsbZIP42	AREB3.	452	2.00E-161	AtbZIP66		259	2.00E-86
BdbZIP35	OsbZIP09		362	7.00E-126	AtbZIP12	DPBF4, EEL, ENHANCED EM LEVEL.	224	5.00E-73
	OsbZIP41		762	0	AtbZIP21	TGA9.	401	4.00E-137
	OsbZIP11		638	0				
	OsbZIP79		410	1.00E-140				
	OsbZIP83		377	8.00E-128				
BdbZIP36	OsbZIP08		348	1.00E-116				
	OsbZIP40		259	3.00E-88				
BdbZIP37	OsbZIP12		204	1.00E-66				
	OsbZIP39		815	0	AtbZIP17	regulate transcription as part of a salt and osmotic stress respon	319	1.00E-100
	OsbZIP60		355	5.00E-116	AtbZIP49	regulate transcription as part of a salt and osmotic stress respon	299	8.00E-94
BdbZIP38					AtbZIP28	Up-regulated in response to heat.	265	2.00E-80
	OsbZIP05		486	1.00E-173	AtbZIP55	GBF3.	202	6.00E-63
BdbZIP41	OsbZIP45	Salt stress inducible bZIP protein.	355	7.00E-122				
	OsbZIP05	Salt stress inducible bZIP protein.	476	1.00E-169	AtbZIP55	GBF3.	198	3.00E-61
BdbZIP42	OsbZIP45		348	5.00E-119				
	OsbZIP06	RF2a.	332	2.00E-116	AtbZIP61	Forms heterodimers with the related protein AtbZIP34.	251	9.00E-84
	OsbZIP02		206	2.00E-66	AtbZIP34	Forms heterodimers with the related protein AtbZIP61.	217	2.00E-70
BdbZIP43	OsbZIP19		202	1.00E-65				
	OsbZIP08		761	0	AtbZIP45	TGA6.	499	9.00E-178
	OsbZIP03	HBP 1b(c38).	512	0	AtbZIP20	AHBP-1B, TGA2.	488	4.00E-173
	OsbZIP28		504	1.00E-179				
BdbZIP45	OsbZIP63		498	6.00E-177				
	OsbZIP09		399	1.00E-140	AtbZIP66		244	2.00E-80
BdbZIP46	OsbZIP42	AREB3.	363	1.00E-126	AtbZIP12	DPBF4, EEL.	210	1.00E-67
BdbZIP47	OsbZIP10	ABI5 isoform 1.	407	9.00E-142				
	OsbZIP11		807	0	AtbZIP21	TGA9.	429	7.00E-147
	OsbZIP41		654	0	AtbZIP65	TGA10.	363	3.00E-122
	OsbZIP79		400	7.00E-147				
BdbZIP48	OsbZIP83		390	1.00E-131				
	OsbZIP11		756	0	AtbZIP21	TGA9.	429	1.00E-146

	OsbZIP41		651	0	AtbZIP65	TGA10.	363	1.00E-122
	OsbZIP79		401	2.00E-136				
BdbZIP49	OsbZIP83		390	3.00E-132				
BdbZIP50	OsbZIP12		231	7.00E-77				
BdbZIP51	OsbZIP12		272	6.00E-93				
	OsbZIP13	HBP 1a.	446	3.00E-159	AtbZIP16		240	1.00E-77
BdbZIP53	OsbZIP86		253	5.00E-83	AtbZIP68		235	6.00E-76
	OsbZIP20	Transcriptional activator protein.	364	1.00E-127				
BdbZIP55	OsbZIP15		363	2.00E-127				
	OsbZIP20	Transcriptional activator protein.	244	1.00E-82				
BdbZIP56	OsbZIP15		231	8.00E-78				
BdbZIP57	[_Os02g08540		239	2.00E-80				
	OsbZIP17		300	1.00E-102				
BdbZIP58	OsbZIP70		216	2.00E-68				
	OsbZIP19		313	3.00E-109	AtbZIP34	Forms heterodimers with AtbZIP61.	202	3.00E-58
	OSbZIP06	RF2a.	247	2.00E-83				
BdbZIP61	OSbZIP02		210	4.00E-68				
	OsbZIP64	STGA1.	354	3.00E-120	AtbZIP47	TGA1.	299	2.00E-99
					At5g65210	TGA1.	299	2.00E-99
					AtbZIP50	TGA7.	292	6.00E-97
					AtbZIP22	TGA3.	291	2.00E-96
BdbZIP62					AtbZIP57		290	3.00E-96
BdbZIP63	OsbZIP65		211	1.00E-71				
	OsbZIP78		451	5.00E-159	AtbZIP29		260	4.00E-83
	OsbZIP25		377	5.00E-130	AtbZIP30		244	3.00E-77
BdbZIP64	OsbZIP68		259	1.00E-83				
	OsbZIP66		400	2.00E-140	At1g45249	ABF2, AREB1, ATABF2, ATAREB1, Enhances drought tolera	259	2.00E-84
	OsbZIP72	ABI5.	389	1.00E-135	AtbZIP35	ABF1, ATABF1.	250	3.00E-81
	OsbZIP23		270	3.00E-89				
BdbZIP65	OsbZIP46		258	3.00E-85				
	OsbZIP75	RF2a.	327	1.00E-111	AtbZIP59		258	3.00E-84
	OsbZIP36		202	3.00E-62	AtbZIP69		250	5.00E-81

BdbZIP67					AtbZIP18		202	4.00E-63
	OsbZIP75	RF2a.	285	3.00E-96	AtbZIP59		285	1.00E-74
BdbZIP68					AtbZIP69		229	5.00E-74
	OsbZIP68		495	3.00E-174	AtbZIP29		317	9.00E-104
	OsbZIP78		284	9.00E-93	AtbZIP30		264	1.00E-83
	OsbZIP25		268	2.00E-86				
BdbZIP69	OsbZIP35		215	3.00E-66				
BdbZIP71	OsbZIP22		273	6.00E-96				
	OsbZIP23		432	8.00E-153				
	OsbZIP46		324	7.00E-111				
	OsbZIP72	ABI5.	267	4.00E-88				
BdbZIP72	OsbZIP66		216	4.00E-69				
BdbZIP75	OsbZIP88		248	3.00E-82				
	C_Os12g37415		226	5.00E-78				
BdbZIP76	OsbZIP87	Ocs element binding factor 1.	226	5.00E-78				
BdbZIP77	OsbZIP82		444	2.00E-157	At1g58110		230	1.00E-73
	OsbZIP84		416	4.00E-147	AtbZIP51	SUE3, VIP1.	236	1.00E-76
BdbZIP78	OsbZIP81	RSG related.	290	1.00E-99				
	OsbZIP84		414	4.00E-144	AtbZIP51	SUE3, VIP1.	237	3.00E-75
BdbZIP79	OsbZIP81	RSG related.	290	2.00E-97				
	OsbZIP79		568	0	AtbZIP21	TGA9.	384	2.00E-130
	OsbZIP83		541	0				
	OsbZIP41		398	8.00E-136				
	OsbZIP11		386	1.00E-130				
BdbZIP80	OsbZIP28		336	5.00E-114				
	OsbZIP70		609	0	AtbZIP65	TGA10.	352	8.00E-119
	OsbZIP74		364	3.00E-122				
	OsbZIP63		333	1.00E-112				
	OsbZIP28		332	2.00E-112				
BdbZIP81	OsbZIP08		333	8.00E-111				
	OsbZIP72	ABI5.	457	3.00E-162				
	OsbZIP66		303	2.00E-102				

	OsbZIP23		264	7.00E-87				
BdbZIP83	OsbZIP46		245	5.00E-80				
	OsbZIP74		676	0	AtbZIP65	TGA10.	389	1.00E-132
	OsbZIP70		390	4.00E-132				
	OsbZIP63		341	5.00E-115				
	OsbZIP28		337	2.00E-113				
BdbZIP85	OsbZIP08		342	3.00E-113				
	OsbZIP75	RF2a.	449	1.00E-158	AtbZIP69		326	5.00E-110
	OsbZIP36		218	6.00E-68	AtbZIP59		320	9.00E-108
BdbZIP86	OsbZIP30	RF2b, Insertion element IS2A.	207	3.00E-65	AtbZIP18		233	7.00E-75
	OsbZIP68		244	1.00E-76				
BdbZIP87	OsbZIP35		226	4.00E-70				
	OsbZIP86		592	0	AtbZIP16		360	2.00E-123
	OsbZIP13	HBP 1a .	295	1.00E-98	AtbZIP68		353	4.00E-121
BdbZIP89	OsbZIP26		224	8.00E-71				
	OsbZIP82		330	7.00E-113	At1g58110		227	2.00E-75
BdbZIP90	OsbZIP85		227	2.00E-75	At4g06598		221	3.00E-70
	OsbZIP84		457	1.00E-163	AtbZIP51	SUE3, VIP1.	219	2.00E-70
	OsbZIP81	RSG related.	285	5.00E-98				
BdbZIP91	OsbZIP61		204	3.00E-64				
BdbZIP92	OsbZIP80		203	3.00E-63				
	OsbZIP42	AREB3.	252	3.00E-83	AtbZIP66		209	4.00E-67
BdbZIP93	OsbZIP09		235	1.00E-76				
	OsbZIP36		383	1.00E-131	AtbZIP59		261	2.00E-85
	OsbZIP75	RF2a.	232	2.00E-74	AtbZIP69		261	7.00E-85
BdbZIP94					AtbZIP18		201	1.00E-62
BdbZIP95	OsbZIP36		313	1.00E-103	AtbZIP698		205	6.00E-63
	OsbZIP37		479	3.00E-170	AtbZIP57		363	3.00E-125
					AtbZIP47	TGA1.	362	1.00E-124
					At5g65201	TGA1.	362	1.00E-124
					AtbZIP22	TGA3.	349	3.00E-119
BdbZIP96					AtbZIP50	TGA7.	343	4.00E-117

Table S9 Tissue specific expression datas

Tissues	root		stem		leaf		flower	
Genes	ΔΔCT valu	p value	ΔΔCT valu	p value	ΔΔCT valu	p value	ΔΔCT valu	p value
**BdbZIP1**	-1.1154	0.0000	0.2385	0.0000	2.0179	0.0000	-0.2754	0.0000
**BdbZIP2**	-2.1901	0.0542	-0.0269	0.0005	1.1263	0.0000	0.2324	0.0088
**BdbZIP3**	-0.1857	0.0028	1.1237	0.0000	1.8583	0.0000	0.2483	0.0001
**BdbZIP4**	-0.5815	0.4082	0.9277	0.0807	3.5642	0.0004	-0.7905	0.0001
**BdbZIP5**	3.5722	0.2838	4.7988	0.0018	6.7958	0.0000	4.8382	0.0094
**BdbZIP6**	0.4616	0.0008	2.2681	0.0000	3.0064	0.0000	2.2930	0.0003
**BdbZIP7**	-1.5662	0.0004	-0.0084	0.0139	0.9826	0.0001	-0.7712	0.0072
**BdbZIP8**	-0.5466	0.3742	0.2590	0.0016	1.8836	0.0001	-0.2881	0.0157
**BdbZIP9**	-1.0633	0.2070	-0.8554	0.0009	-0.5402	0.0000	-1.7540	0.0043
**BdbZIP10**	-0.6792	0.0006	0.3701	0.4628	1.8948	0.0002	0.3898	0.0374
**BdbZIP11**	-3.1735	0.0300	-1.2876	0.0265	-0.3669	0.0001	-2.0464	0.0031
**BdbZIP12**	-1.3301	0.0000	0.1269	0.0002	1.6202	0.0420	-0.3917	0.0001
**BdbZIP13**	-0.7625	0.0001	0.4111	0.0504	2.5746	0.0000	0.8842	0.0001
**BdbZIP14**	-0.9526	0.0004	-0.0182	0.0438	1.6150	0.0022	-0.1567	0.0013
**BdbZIP15**	-3.3127	0.0164	-0.6128	0.0166	1.7381	0.0000	-2.3092	0.1905
**BdbZIP16**	-1.0861	0.0647	-0.0083	0.0115	1.0103	0.0000	-0.2530	0.0004
**BdbZIP17**	1.1951	0.0001	2.6196	0.0000	4.6644	0.0000	2.7081	0.0000
**BdbZIP18**	-0.7533	0.0055	0.2182	0.0170	2.3064	0.0743	-0.7227	0.0021
**BdbZIP19**	-1.7773	0.0002	-0.6855	0.0687	0.3389	0.0122	-1.1031	0.1660
**BdbZIP20**	-1.7417	0.0013	-0.2361	0.2877	0.4641	0.0057	-0.6055	0.2475
**BdbZIP21**	0.2315	0.0002	0.5560	0.0001	1.8490	0.0000	-0.9835	0.0000
**BdbZIP22**	-2.5279	0.0002	-0.8789	0.0112	-2.1281	0.0002	-1.1152	0.0036
**BdbZIP23**	-2.8103	0.0000	-1.1238	0.0004	-2.3607	0.3077	-1.3532	0.0002
**BdbZIP24**	-1.4010	0.0057	-0.1122	0.0005	1.8479	0.0000	-0.6251	0.0382
**BdbZIP25**	-1.9378	0.0202	-0.0037	0.1367	1.1021	0.0138	-0.1994	0.1281
**BdbZIP26**	-1.8292	0.0000	-0.4156	0.0084	-3.0894	0.0001	-1.5639	0.1236
**BdbZIP27**	-2.0575	0.0000	-0.8653	0.0006	1.4980	0.0000	-1.0875	0.0003
**BdbZIP40**	-2.2706	0.0004	-0.8862	0.0985	-2.6233	0.0005	-1.0785	0.4332
**BdbZIP28**	-1.8433	0.3061	-0.6703	0.0110	1.1523	0.0000	-1.2507	0.0014
**BdbZIP29**	-0.6637	0.0012	0.6115	0.0928	2.7100	0.0000	1.0243	0.0275
**BdbZIP30**	-1.2558	0.0036	-0.5776	0.1426	0.6463	0.0002	0.1950	0.1436
**BdbZIP31**	-3.6135	0.0000	-2.1298	0.0141	0.0444	0.0001	-2.3091	0.0008
**BdbZIP32**	-2.1203	0.0000	-0.7667	0.0000	1.4557	0.0000	-0.5628	0.0000
**BdbZIP33**	-2.1307	0.0003	-1.9323	0.0000	-0.8392	0.0000	-1.7393	0.0000
**BdbZIP34**	3.8115	0.0000	4.8619	0.0000	6.9747	0.0000	4.9318	0.0000
**BdbZIP35**	3.0526	0.0046	2.0923	0.3907	3.5034	0.0001	0.7558	0.1349
**BdbZIP36**	0.4459	0.0979	1.3471	0.0072	3.8241	0.0373	0.9309	0.0001
**BdbZIP37**	0.0549	0.0000	0.3790	0.0233	0.0838	0.0000	-2.1046	0.0033
**BdbZIP38**	-0.7768	0.0005	0.2772	0.0030	1.2600	0.0000	-0.0708	0.1089
**BdbZIP39**	-0.8626	0.0412	0.0671	0.0255	0.7923	0.0001	0.7775	0.0103
**BdbZIP41**	-1.9288	0.1335	-0.1867	0.0010	1.7398	0.0003	-0.6679	0.0011
**BdbZIP42**	0.3696	0.0248	0.9658	0.0913	2.7120	0.0001	0.1882	0.0080
**BdbZIP43**	-2.7063	0.0006	-0.9021	0.1978	0.9982	0.0000	-2.8992	0.0007
**BdbZIP44**	-2.1020	0.0000	-0.2849	0.0000	0.3161	0.0000	-0.3827	0.0000
**BdbZIP45**	-3.1592	0.0030	-0.8075	0.0163	0.7393	0.0000	-2.1823	0.4446

**BdbZIP46**	8.1693	0.0738	9.6147	0.0658	11.8239	0.0026	10.9015	0.2737
**BdbZIP47**	1.0613	0.0000	2.2483	0.0000	4.1948	0.0000	2.2773	0.0000
**BdbZIP48**	-0.8444	0.0002	0.2615	0.0001	2.1836	0.0000	0.6757	0.0000
**BdbZIP49**	2.2901	0.0053	4.0902	0.0002	4.3934	0.0000	3.8932	0.0004
**BdbZIP50**	-1.9076	0.0000	-0.6598	0.0000	-3.8311	0.0000	-2.9135	0.0000
**BdbZIP51**	-1.7541	0.0009	-0.6573	0.0000	0.8322	0.0000	-0.8485	0.0001
**BdbZIP52**	0.5724	0.0016	-0.2365	0.0000	-1.1962	0.0000	-1.4335	0.0001
**BdbZIP53**	-1.6299	0.0004	0.1624	0.0087	1.8007	0.0001	0.1091	0.0589
**BdbZIP54**	1.4303	0.0225	2.2817	0.0096	4.6153	0.0000	2.6775	0.0080
**BdbZIP55**	-2.3119	0.0008	-0.3535	0.0004	-0.4769	0.0009	0.0722	0.0001
**BdbZIP56**	-1.8342	0.0137	-0.3641	0.0607	0.6394	0.0053	-0.0675	0.0013
**BdbZIP57**	-1.5345	0.0000	-0.0285	0.0110	0.8951	0.0009	-0.3809	0.0000
**BdbZIP58**	2.7294	0.0042	0.8269	0.0081	0.7070	0.0001	0.7779	0.0029
**BdbZIP59**	-2.0719	0.0005	-0.9354	0.0085	1.0468	0.0268	-1.4223	0.0036
**BdbZIP60**	-0.4489	0.0000	3.0867	0.0000	5.8978	0.0000	1.8663	0.0000
**BdbZIP61**	-2.9642	0.0004	-1.0182	0.0247	1.0434	0.0000	-2.1227	0.0001
**BdbZIP62**	2.2423	0.0289	3.1409	0.0007	5.1664	0.0000	3.1895	0.0039
**BdbZIP63**	-1.2838	0.1482	0.0367	0.0001	1.8519	0.0001	0.0035	0.0000
**BdbZIP64**	3.9852	0.0589	5.3028	0.0004	7.5438	0.0000	5.3580	0.0005
**BdbZIP65**	0.6027	0.0006	1.5888	0.0412	3.2859	0.1269	1.7809	0.0096
**BdbZIP66**	-0.2860	0.0000	-1.2241	0.0000	1.3744	0.0000	-1.1110	0.0000
**BdbZIP67**	-0.1160	0.0001	0.6078	0.0000	2.0415	0.0000	0.4583	0.0001
**BdbZIP68**	-0.3340	0.0047	0.2487	0.0004	2.4611	0.0000	0.5043	0.0001
**BdbZIP69**	-2.6384	0.0017	-0.5205	0.0309	1.1470	0.0176	-1.2542	0.0041
**BdbZIP70**	-1.0113	0.0161	-0.3049	0.1935	1.4563	0.0002	-0.0503	0.3013
**BdbZIP71**	0.9838	0.0067	0.8495	0.0000	2.8942	0.0000	0.9267	0.0000
**BdbZIP72**	-0.0148	0.0006	2.2228	0.0134	2.3503	0.0006	2.9968	0.0027
**BdbZIP73**	-0.9841	0.0000	-0.6456	0.0222	0.6010	0.0005	-1.8931	0.0003
**BdbZIP74**	-1.1148	0.0015	0.2417	0.0005	2.3137	0.0000	0.5403	0.0002
**BdbZIP75**	1.8614	0.1519	2.5767	0.0000	4.7016	0.0000	2.2890	0.0000
**BdbZIP76**	3.2872	0.0001	4.1574	0.0004	6.2149	0.0007	4.0829	0.0006
**BdbZIP77**	-3.6430	0.0001	0.3401	0.0550	-2.0324	0.0000	-1.1300	0.0020
**BdbZIP78**	4.6627	0.0000	5.3881	0.0060	7.5936	0.0000	5.1817	0.0160
**BdbZIP79**	0.2420	0.0001	1.8856	0.0156	3.2446	0.0002	1.2503	0.0125
**BdbZIP80**	-0.7713	0.0000	0.4783	0.0003	2.2778	0.0000	0.2761	0.0036
**BdbZIP81**	5.0443	0.0001	6.9116	0.0054	8.9074	0.0159	5.6288	0.0073
**BdbZIP82**	-2.3655	0.0177	-1.2852	0.1208	-2.9088	0.0017	-1.7279	0.0020
**BdbZIP83**	-0.3049	0.0007	1.0378	0.0189	2.3104	0.0007	0.3011	0.0085
**BdbZIP84**	-2.0781	0.0068	-0.5999	0.0253	1.0137	0.0001	0.0441	0.4095
**BdbZIP85**	0.7831	0.0000	1.8606	0.4739	3.7889	0.0013	1.7292	0.0005
**BdbZIP86**	-2.1372	0.0003	-0.9715	0.4790	1.2227	0.0011	0.1012	0.0138
**BdbZIP87**	2.1679	0.0033	2.7715	0.0001	5.3949	0.0000	2.5087	0.0016
**BdbZIP88**	-1.1191	0.0000	-0.8015	0.0001	1.0719	0.0412	-1.4855	0.0000
**BdbZIP89**	1.5592	0.0006	2.8795	0.4106	5.3487	0.0207	2.6393	0.2020
**BdbZIP90**	-0.4543	0.0009	-0.7307	0.0303	1.2461	0.0001	-0.7781	0.0505
**BdbZIP91**	1.5590	0.0011	2.3559	0.0501	4.9404	0.0001	2.3367	0.0024
**BdbZIP92**	-0.0974	0.0066	1.0056	0.1396	3.0156	0.0001	0.4916	0.0162
**BdbZIP93**	0.5889	0.3686	2.3561	0.0000	3.8186	0.0000	1.2082	0.0040

**BdbZIP94**	0.8140	0.0000	1.5372	0.0000	3.8975	0.0000	1.8421	0.0000
**BdbZIP95**	-2.2756	0.0001	-1.6037	0.0001	-2.7041	0.0000	-1.6307	0.0001
**BdbZIP96**	-4.1832	0.0125	-1.7520	0.0009	0.1960	0.0000	-3.8348	0.0135

Table S10 Stress expression datas, BdbZIP genes with expression level changes ≥2 folds.

Treatments	cold				heat			
	1h		6h		1h		6h	
Genes	fold change	p-value	fold change	p-value	fold change	p-value	fold change	p-value
BdbZIP1	0. 0084	7. 1298E-06	0. 1701	3. 2375E-04	0. 0152	6. 3184E-06	0. 0293	1. 1639E-06
BdbZIP2	0. 0224	9. 1392E-06	0. 3755	8. 8176E-04	0. 4658	7. 2122E-03	0. 3259	2. 0177E-04
BdbZIP3	0. 1504	4. 4582E-02	0. 9025	1. 4423E-01	0. 8139	3. 2572E-01	0. 5417	8. 6838E-04
BdbZIP4	0. 2602	3. 6910E-03	0. 3182	8. 0801E-04	12. 7111	2. 2532E-05	13. 3369	3. 4861E-06
BdbZIP5	0. 0686	4. 3195E-04	0. 3414	7. 2574E-04	0. 3626	1. 3608E-03	0. 4346	1. 3733E-03
BdbZIP6	0. 1062	3. 6012E-03	0. 5044	2. 5494E-02	0. 0939	3. 2984E-05	0. 2808	5. 1775E-06
BdbZIP7	0. 3084	6. 8568E-03	1. 7271	1. 1285E-03	3. 0638	6. 8898E-04	4. 1577	4. 8946E-04
BdbZIP8	3. 1401	6. 4212E-02	0. 6037	1. 1357E-01	0. 2589	9. 2240E-04	3. 4182	3. 1671E-04
BdbZIP9	0. 2342	5. 4533E-02	0. 6190	9. 0514E-02	0. 1427	3. 2364E-05	0. 3838	5. 8693E-04
BdbZIP10	0. 4053	4. 3389E-03	1. 5951	1. 9928E-03	2. 4444	2. 9198E-02	2. 3784	1. 7147E-03
BdbZIP11	0. 0897	2. 4381E-03	0. 3349	2. 1106E-03	0. 8860	4. 1564E-01	0. 7497	1. 8712E-02
BdbZIP12	0. 1103	1. 0655E-02	0. 6671	2. 2218E-01	1. 6537	9. 9646E-03	1. 3490	1. 5592E-01
BdbZIP13	0. 0556	8. 4821E-05	0. 4390	3. 4478E-04	2. 6505	5. 7389E-04	1. 8081	5. 5816E-03
BdbZIP14	0. 1823	4. 9093E-01	1. 3915	3. 0745E-03	6. 3239	2. 2043E-05	5. 1220	7. 1326E-05
BdbZIP15	0. 1085	1. 9268E-03	0. 7554	3. 2181E-01	0. 8233	4. 3248E-01	0. 7301	3. 2334E-03
BdbZIP16	0. 4615	7. 3872E-04	0. 5378	5. 6646E-03	14. 2443	2. 0816E-06	52. 7401	3. 9988E-06
BdbZIP17	0. 3158	1. 3209E-02	1. 2188	1. 7469E-02	0. 1217	1. 2202E-04	0. 1596	2. 5208E-04
BdbZIP18	0. 3476	5. 6915E-03	1. 6162	2. 6830E-03	3. 3952	8. 4361E-04	5. 9123	3. 5674E-04
BdbZIP19	0. 1668	1. 7243E-01	0. 8973	5. 1206E-03	0. 6547	5. 5028E-02	0. 8748	4. 6342E-02
BdbZIP20	0. 1772	4. 8258E-01	0. 7680	4. 0358E-01	0. 9556	2. 1912E-01	1. 1482	4. 3860E-01
BdbZIP21	0. 1645	3. 0564E-01	0. 8300	2. 2035E-01	1. 8844	1. 7606E-03	3. 0032	1. 8088E-03
BdbZIP22	0. 3282	1. 4877E-03	2. 2793	3. 9372E-04	3. 4380	4. 0448E-04	4. 2742	1. 4590E-04
BdbZIP23	0. 1688	2. 5415E-01	0. 5494	5. 0885E-02	1. 7184	4. 6463E-04	1. 6566	1. 4999E-02
BdbZIP24	0. 0499	1. 4594E-04	0. 1725	8. 2243E-05	0. 5060	1. 1314E-02	0. 3841	6. 9288E-04
BdbZIP25	0. 7858	7. 6020E-04	1. 2593	3. 0719E-02	1. 6682	1. 5930E-02	1. 5596	7. 0153E-02
BdbZIP26	0. 1058	5. 1693E-04	0. 5353	2. 0286E-04	1. 2255	1. 5699E-03	1. 2063	2. 6300E-02
BdbZIP27	0. 3576	3. 3171E-04	2. 4717	5. 2257E-05	3. 7380	2. 0543E-04	5. 1673	7. 3850E-05
BdbZIP28	0. 2735	6. 3524E-02	3. 7101	3. 6600E-03	0. 7558	5. 4702E-04	2. 5242	6. 0075E-04
BdbZIP29	0. 5884	3. 4269E-03	6. 7286	2. 9617E-04	9. 9553	1. 8314E-01	21. 4249	2. 9509E-04
BdbZIP30	0. 1056	3. 3922E-05	0. 6303	8. 8262E-06	0. 9087	2. 7109E-05	0. 9984	4. 7028E-06
BdbZIP31	0. 1461	9. 0560E-03	0. 5522	1. 9307E-01	0. 8619	2. 8287E-01	1. 0764	2. 5003E-01
BdbZIP32	3. 1588	4. 7482E-02	1. 0287	2. 6149E-02	0. 2426	4. 3714E-01	2. 4679	3. 0580E-01
BdbZIP33	0. 1412	6. 1490E-06	0. 7936	4. 6429E-03	1. 2208	8. 8261E-05	1. 4990	4. 1036E-03
BdbZIP34	0. 7687	2. 2334E-02	3. 9947	2. 2684E-01	3. 6119	7. 5759E-03	1. 8698	3. 3488E-02
BdbZIP35	0. 2019	5. 6509E-05	0. 8811	2. 1242E-04	2. 1892	3. 4158E-04	2. 6481	3. 4995E-03
BdbZIP36	0. 1412	9. 9613E-02	0. 4349	4. 7650E-02	0. 6729	5. 6789E-04	0. 8405	1. 9767E-04
BdbZIP37	0. 3170	2. 7824E-02	1. 3144	4. 9005E-03	0. 8256	8. 8933E-02	1. 7010	6. 1351E-02
BdbZIP38	0. 0892	1. 6131E-03	0. 2941	4. 9460E-03	0. 9440	4. 5836E-01	1. 2696	1. 3532E-02
BdbZIP39	0. 1336	1. 5573E-04	0. 2946	9. 2835E-05	0. 7512	2. 1660E-01	0. 6434	1. 4990E-01
BdbZIP40	0. 2155	5. 8116E-02	1. 5231	8. 1719E-04	2. 5635	2. 7374E-01	4. 3400	8. 3689E-03
BdbZIP41	0. 9921	1. 4647E-06	0. 4544	9. 0087E-05	0. 1505	3. 7931E-05	1. 3315	3. 1819E-02
BdbZIP42	0. 0604	1. 3472E-04	0. 3495	3. 6075E-03	0. 5002	8. 5681E-03	0. 8151	1. 7898E-02
BdbZIP43	0. 2879	5. 1201E-03	2. 4357	7. 1109E-05	2. 7140	1. 2593E-03	4. 5821	6. 2486E-05
BdbZIP44	0. 0423	5. 6822E-04	0. 3660	4. 1240E-03	1. 0632	1. 2722E-01	0. 9537	1. 9193E-01
BdbZIP45	0. 1110	1. 9812E-04	0. 5733	1. 2692E-02	1. 4694	1. 1396E-02	1. 4151	4. 1525E-02
BdbZIP46	0. 1440	1. 4651E-01	0. 8439	2. 3508E-01	1. 3877	2. 8242E-02	1. 2098	3. 3590E-01
BdbZIP47	0. 3109	1. 0153E-02	0. 7469	4. 8098E-01	2. 2993	1. 0067E-03	1. 2761	1. 7233E-01
BdbZIP48	0. 9788	3. 2361E-05	0. 8268	8. 5398E-02	0. 2541	1. 0922E-04	1. 6229	1. 5382E-03
BdbZIP49	0. 1822	4. 3284E-01	0. 6719	2. 6261E-01	0. 9000	3. 7334E-01	1. 3136	2. 1017E-01
BdbZIP50	0. 0198	5. 3386E-05	0. 2765	3. 4907E-04	0. 0547	2. 2609E-06	0. 0852	3. 5547E-05
BdbZIP51	0. 2356	7. 4958E-02	1. 2891	8. 0866E-03	0. 0932	2. 7797E-05	0. 5461	2. 0508E-03

Table S9 Tissue specific expression datas

Tissues	root		stem		leaf		flower	
Genes	ΔΔCT value	p value	ΔΔCT value	p value	ΔΔCT value	p value	ΔΔCT value	p value
**BdbZIP1**	-1.1154	0.0000	0.2385	0.0000	2.0179	0.0000	-0.2754	0.0000
**BdbZIP2**	-2.1901	0.0542	-0.0269	0.0005	1.1263	0.0000	0.2324	0.0088
**BdbZIP3**	-0.1857	0.0028	1.1237	0.0000	1.8583	0.0000	0.2483	0.0001
**BdbZIP4**	-0.5815	0.4082	0.9277	0.0807	3.5642	0.0004	-0.7905	0.0001
**BdbZIP5**	3.5722	0.2838	4.7988	0.0018	6.7958	0.0000	4.8382	0.0094
**BdbZIP6**	0.4616	0.0008	2.2681	0.0000	3.0064	0.0000	2.2930	0.0003
**BdbZIP7**	-1.5662	0.0004	-0.0084	0.0139	0.9826	0.0001	-0.7712	0.0072
**BdbZIP8**	-0.5466	0.3742	0.2590	0.0016	1.8836	0.0001	-0.2881	0.0157
**BdbZIP9**	-1.0633	0.2070	-0.8554	0.0009	-0.5402	0.0000	-1.7540	0.0043
**BdbZIP10**	-0.6792	0.0006	0.3701	0.4628	1.8948	0.0002	0.3898	0.0374
**BdbZIP11**	-3.1735	0.0300	-1.2876	0.0265	-0.3669	0.0001	-2.0464	0.0031
**BdbZIP12**	-1.3301	0.0000	0.1269	0.0002	1.6202	0.0420	-0.3917	0.0001
**BdbZIP13**	-0.7625	0.0001	0.4111	0.0504	2.5746	0.0000	0.8842	0.0001
**BdbZIP14**	-0.9526	0.0004	-0.0182	0.0438	1.6150	0.0022	-0.1567	0.0013
**BdbZIP15**	-3.3127	0.0164	-0.6128	0.0166	1.7381	0.0000	-2.3092	0.1905
**BdbZIP16**	-1.0861	0.0647	-0.0083	0.0115	1.0103	0.0000	-0.2530	0.0004
**BdbZIP17**	1.1951	0.0001	2.6196	0.0000	4.6644	0.0000	2.7081	0.0000
**BdbZIP18**	-0.7533	0.0055	0.2182	0.0170	2.3064	0.0743	-0.7227	0.0021
**BdbZIP19**	-1.7773	0.0002	-0.6855	0.0687	0.3389	0.0122	-1.1031	0.1660
**BdbZIP20**	-1.7417	0.0013	-0.2361	0.2877	0.4641	0.0057	-0.6055	0.2475
**BdbZIP21**	0.2315	0.0002	0.5560	0.0001	1.8490	0.0000	-0.9835	0.0000
**BdbZIP22**	-2.5279	0.0002	-0.8789	0.0112	-2.1281	0.0002	-1.1152	0.0036
**BdbZIP23**	-2.8103	0.0000	-1.1238	0.0004	-2.3607	0.3077	-1.3532	0.0002
**BdbZIP24**	-1.4010	0.0057	-0.1122	0.0005	1.8479	0.0000	-0.6251	0.0382
**BdbZIP25**	-1.9378	0.0202	-0.0037	0.1367	1.1021	0.0138	-0.1994	0.1281
**BdbZIP26**	-1.8292	0.0000	-0.4156	0.0084	-3.0894	0.0001	-1.5639	0.1236
**BdbZIP27**	-2.0575	0.0000	-0.8653	0.0006	1.4980	0.0000	-1.0875	0.0003
**BdbZIP40**	-2.2706	0.0004	-0.8862	0.0985	-2.6233	0.0005	-1.0785	0.4332
**BdbZIP28**	-1.8433	0.3061	-0.6703	0.0110	1.1523	0.0000	-1.2507	0.0014
**BdbZIP29**	-0.6637	0.0012	0.6115	0.0928	2.7100	0.0000	1.0243	0.0275
**BdbZIP30**	-1.2558	0.0036	-0.5776	0.1426	0.6463	0.0002	0.1950	0.1436
**BdbZIP31**	-3.6135	0.0000	-2.1298	0.0141	0.0444	0.0001	-2.3091	0.0008
**BdbZIP32**	-2.1203	0.0000	-0.7667	0.0000	1.4557	0.0000	-0.5628	0.0000
**BdbZIP33**	-2.1307	0.0003	-1.9323	0.0000	-0.8392	0.0000	-1.7393	0.0000
**BdbZIP34**	3.8115	0.0000	4.8619	0.0000	6.9747	0.0000	4.9318	0.0000
**BdbZIP35**	3.0526	0.0046	2.0923	0.3907	3.5034	0.0001	0.7558	0.1349
**BdbZIP36**	0.4459	0.0979	1.3471	0.0072	3.8241	0.0373	0.9309	0.0001
**BdbZIP37**	0.0549	0.0000	0.3790	0.0233	0.0838	0.0000	-2.1046	0.0033
**BdbZIP38**	-0.7768	0.0005	0.2772	0.0030	1.2600	0.0000	-0.0708	0.1089
**BdbZIP39**	-0.8626	0.0412	0.0671	0.0255	0.7923	0.0001	0.7775	0.0103
**BdbZIP41**	-1.9288	0.1335	-0.1867	0.0010	1.7398	0.0003	-0.6679	0.0011
**BdbZIP42**	0.3696	0.0248	0.9658	0.0913	2.7120	0.0001	0.1882	0.0080
**BdbZIP43**	-2.7063	0.0006	-0.9021	0.1978	0.9982	0.0000	-2.8992	0.0007
**BdbZIP44**	-2.1020	0.0000	-0.2849	0.0000	0.3161	0.0000	-0.3827	0.0000
**BdbZIP45**	-3.1592	0.0030	-0.8075	0.0163	0.7393	0.0000	-2.1823	0.4446

**BdbZIP46**	8.1693	0.0738	9.6147	0.0658	11.8239	0.0026	10.9015	0.2737
**BdbZIP47**	1.0613	0.0000	2.2483	0.0000	4.1948	0.0000	2.2773	0.0000
**BdbZIP48**	-0.8444	0.0002	0.2615	0.0001	2.1836	0.0000	0.6757	0.0000
**BdbZIP49**	2.2901	0.0053	4.0902	0.0002	4.3934	0.0000	3.8932	0.0004
**BdbZIP50**	-1.9076	0.0000	-0.6598	0.0000	-3.8311	0.0000	-2.9135	0.0000
**BdbZIP51**	-1.7541	0.0009	-0.6573	0.0000	0.8322	0.0000	-0.8485	0.0001
**BdbZIP52**	0.5724	0.0016	-0.2365	0.0000	-1.1962	0.0000	-1.4335	0.0001
**BdbZIP53**	-1.6299	0.0004	0.1624	0.0087	1.8007	0.0001	0.1091	0.0589
**BdbZIP54**	1.4303	0.0225	2.2817	0.0096	4.6153	0.0000	2.6775	0.0080
**BdbZIP55**	-2.3119	0.0008	-0.3535	0.0004	-0.4769	0.0009	0.0722	0.0001
**BdbZIP56**	-1.8342	0.0137	-0.3641	0.0607	0.6394	0.0053	-0.0675	0.0013
**BdbZIP57**	-1.5345	0.0000	-0.0285	0.0110	0.8951	0.0009	-0.3809	0.0000
**BdbZIP58**	2.7294	0.0042	0.8269	0.0081	0.7070	0.0001	0.7779	0.0029
**BdbZIP59**	-2.0719	0.0005	-0.9354	0.0085	1.0468	0.0268	-1.4223	0.0036
**BdbZIP60**	-0.4489	0.0000	3.0867	0.0000	5.8978	0.0000	1.8663	0.0000
**BdbZIP61**	-2.9642	0.0004	-1.0182	0.0247	1.0434	0.0000	-2.1227	0.0001
**BdbZIP62**	2.2423	0.0289	3.1409	0.0007	5.1664	0.0000	3.1895	0.0039
**BdbZIP63**	-1.2838	0.1482	0.0367	0.0001	1.8519	0.0001	0.0035	0.0000
**BdbZIP64**	3.9852	0.0589	5.3028	0.0004	7.5438	0.0000	5.3580	0.0005
**BdbZIP65**	0.6027	0.0006	1.5888	0.0412	3.2859	0.1269	1.7809	0.0096
**BdbZIP66**	-0.2860	0.0000	-1.2241	0.0000	1.3744	0.0000	-1.1110	0.0000
**BdbZIP67**	-0.1160	0.0001	0.6078	0.0000	2.0415	0.0000	0.4583	0.0001
**BdbZIP68**	-0.3340	0.0047	0.2487	0.0004	2.4611	0.0000	0.5043	0.0001
**BdbZIP69**	-2.6384	0.0017	-0.5205	0.0309	1.1470	0.0176	-1.2542	0.0041
**BdbZIP70**	-1.0113	0.0161	-0.3049	0.1935	1.4563	0.0002	-0.0503	0.3013
**BdbZIP71**	0.9838	0.0067	0.8495	0.0000	2.8942	0.0000	0.9267	0.0000
**BdbZIP72**	-0.0148	0.0006	2.2228	0.0134	2.3503	0.0006	2.9968	0.0027
**BdbZIP73**	-0.9841	0.0000	-0.6456	0.0222	0.6010	0.0005	-1.8931	0.0003
**BdbZIP74**	-1.1148	0.0015	0.2417	0.0005	2.3137	0.0000	0.5403	0.0002
**BdbZIP75**	1.8614	0.1519	2.5767	0.0000	4.7016	0.0000	2.2890	0.0000
**BdbZIP76**	3.2872	0.0001	4.1574	0.0004	6.2149	0.0007	4.0829	0.0006
**BdbZIP77**	-3.6430	0.0001	0.3401	0.0550	-2.0324	0.0000	-1.1300	0.0020
**BdbZIP78**	4.6627	0.0000	5.3881	0.0060	7.5936	0.0000	5.1817	0.0160
**BdbZIP79**	0.2420	0.0001	1.8856	0.0156	3.2446	0.0002	1.2503	0.0125
**BdbZIP80**	-0.7713	0.0000	0.4783	0.0003	2.2778	0.0000	0.2761	0.0036
**BdbZIP81**	5.0443	0.0001	6.9116	0.0054	8.9074	0.0159	5.6288	0.0073
**BdbZIP82**	-2.3655	0.0177	-1.2852	0.1208	-2.9088	0.0017	-1.7279	0.0020
**BdbZIP83**	-0.3049	0.0007	1.0378	0.0189	2.3104	0.0007	0.3011	0.0085
**BdbZIP84**	-2.0781	0.0068	-0.5999	0.0253	1.0137	0.0001	0.0441	0.4095
**BdbZIP85**	0.7831	0.0000	1.8606	0.4739	3.7889	0.0013	1.7292	0.0005
**BdbZIP86**	-2.1372	0.0003	-0.9715	0.4790	1.2227	0.0011	0.1012	0.0138
**BdbZIP87**	2.1679	0.0033	2.7715	0.0001	5.3949	0.0000	2.5087	0.0016
**BdbZIP88**	-1.1191	0.0000	-0.8015	0.0001	1.0719	0.0412	-1.4855	0.0000
**BdbZIP89**	1.5592	0.0006	2.8795	0.4106	5.3487	0.0207	2.6393	0.2020
**BdbZIP90**	-0.4543	0.0009	-0.7307	0.0303	1.2461	0.0001	-0.7781	0.0505
**BdbZIP91**	1.5590	0.0011	2.3559	0.0501	4.9404	0.0001	2.3367	0.0024
**BdbZIP92**	-0.0974	0.0066	1.0056	0.1396	3.0156	0.0001	0.4916	0.0162
**BdbZIP93**	0.5889	0.3686	2.3561	0.0000	3.8186	0.0000	1.2082	0.0040

**BdbZIP94**	0.8140	0.0000	1.5372	0.0000	3.8975	0.0000	1.8421	0.0000
**BdbZIP95**	-2.2756	0.0001	-1.6037	0.0001	-2.7041	0.0000	-1.6307	0.0001
**BdbZIP96**	-4.1832	0.0125	-1.7520	0.0009	0.1960	0.0000	-3.8348	0.0135

BdbZIP52	0. 2682	1. 2299E-02	2. 1068	3. 1321E-04	23. 4262	2. 0216E-07	7. 3217	4. 5099E-05
BdbZIP53	0. 1038	2. 8814E-03	0. 4754	9. 5900E-03	0. 8350	4. 6600E-01	0. 8271	2. 8570E-02
BdbZIP54	0. 1009	1. 6803E-02	0. 3389	1. 9975E-03	0. 2325	1. 4042E-03	0. 2326	4. 0798E-04
BdbZIP55	0. 7193	3. 8687E-06	0. 9313	7. 7919E-03	0. 2167	1. 9826E-06	0. 8910	1. 8013E-02
BdbZIP56	0. 1093	1. 7281E-02	0. 6754	2. 7326E-01	0. 4085	8. 7474E-03	0. 3951	1. 6434E-03
BdbZIP57	0. 1627	2. 4467E-01	1. 5959	1. 6980E-03	3. 3765	4. 6077E-04	2. 5414	1. 2967E-03
BdbZIP58	0. 3257	5. 0182E-03	1. 0549	7. 2267E-02	3. 1216	2. 4348E-04	1. 5207	5. 9527E-02
BdbZIP59	0. 1350	7. 1413E-02	1. 6965	7. 2081E-03	1. 3732	2. 8346E-02	20. 6881	1. 9337E-05
BdbZIP60	0. 3470	1. 8611E-01	0. 5123	6. 5001E-03	1. 4670	3. 2097E-03	0. 1004	4. 4444E-05
BdbZIP61	0. 6492	9. 4198E-05	2. 2918	1. 1071E-04	7. 3287	1. 2253E-05	10. 2739	2. 0942E-05
BdbZIP62	0. 0996	4. 3066E-04	1. 6118	1. 8243E-04	3. 4971	1. 8537E-04	2. 8303	1. 3295E-05
BdbZIP63	0. 0525	4. 4890E-04	0. 2241	3. 1579E-04	0. 1427	2. 1176E-04	0. 1751	4. 7446E-05
BdbZIP64	0. 8885	1. 5139E-04	4. 1224	1. 4642E-04	13. 1804	2. 0722E-05	7. 1778	4. 9809E-05
BdbZIP65	0. 1377	3. 6516E-02	0. 8340	1. 5289E-01	1. 3734	7. 8991E-03	1. 1067	4. 6373E-01
BdbZIP66	0. 2567	3. 7228E-02	3. 2446	1. 5335E-04	6. 1336	9. 4781E-03	5. 2527	4. 6466E-04
BdbZIP67	1. 0673	6. 5225E-05	0. 5533	6. 8096E-03	0. 1133	6. 9152E-06	1. 8799	8. 4932E-04
BdbZIP68	0. 0434	3. 1165E-04	0. 2282	7. 0030E-04	2. 1178	8. 8979E-04	2. 1503	6. 1710E-03
BdbZIP69	0. 1089	1. 2431E-02	0. 7396	4. 8973E-01	1. 1786	3. 7763E-02	1. 1434	4. 6927E-01
BdbZIP70	0. 1295	2. 0382E-02	0. 5083	1. 5505E-02	1. 1256	6. 6622E-02	0. 9931	2. 0617E-01
BdbZIP71	0. 0632	5. 6676E-05	0. 1575	1. 0695E-05	0. 6364	7. 0680E-02	0. 7525	1. 2713E-02
BdbZIP72	0. 4676	3. 4329E-04	1. 9291	3. 2732E-04	1. 3045	1. 3168E-02	1. 9979	2. 6022E-03
BdbZIP73	0. 0569	1. 7852E-04	1. 7018	2. 5140E-04	2. 1026	3. 2371E-03	1. 8293	7. 5302E-03
BdbZIP74	0. 1843	4. 4662E-01	0. 7584	4. 8773E-01	1. 2101	1. 0182E-01	0. 7611	5. 5342E-02
BdbZIP75	0. 4543	8. 0293E-05	0. 0270	4. 9394E-07	16. 9760	1. 9284E-05	8. 3930	4. 8291E-05
BdbZIP76	0. 2010	7. 9503E-02	14. 9777	1. 8925E-06	10. 3617	1. 9809E-06	19. 0548	1. 4882E-05
BdbZIP77	0. 1012	2. 8544E-04	0. 3854	1. 2468E-04	1. 4727	4. 1289E-03	1. 6854	1. 4685E-02
BdbZIP78	0. 1859	1. 8046E-05	0. 5339	4. 5119E-02	2. 0814	1. 5171E-05	1. 7366	8. 1455E-05
BdbZIP79	0. 1324	2. 6148E-01	0. 6247	2. 6532E-02	0. 2050	7. 1045E-04	0. 2454	2. 2353E-02
BdbZIP80	0. 0418	1. 5749E-04	0. 2544	9. 2552E-04	0. 3616	3. 6282E-03	0. 4339	1. 6138E-03
BdbZIP81	0. 0364	3. 7686E-04	0. 1424	5. 4284E-04	0. 1970	7. 6510E-04	0. 2917	1. 0028E-03
BdbZIP82	0. 0823	1. 5918E-03	0. 3704	2. 7466E-03	2. 0683	3. 4910E-03	2. 4316	2. 7368E-03
BdbZIP83	0. 3059	2. 3499E-02	1. 0033	6. 4430E-02	2. 8595	4. 8571E-04	3. 7059	1. 2219E-03
BdbZIP84	0. 2559	1. 2460E-02	1. 1721	1. 2677E-02	0. 7660	2. 8942E-01	0. 8721	8. 9904E-02
BdbZIP85	0. 1154	1. 7863E-02	0. 4574	4. 6837E-04	0. 9463	6. 7755E-02	1. 3376	3. 1192E-02
BdbZIP86	0. 1693	1. 5785E-01	0. 9101	1. 3947E-02	0. 7689	2. 8586E-01	1. 0856	3. 4721E-01
BdbZIP87	0. 2135	6. 4844E-02	0. 9646	8. 7772E-03	2. 0843	1. 0294E-03	2. 2524	2. 0228E-04
BdbZIP88	2. 6410	3. 6513E-07	1. 6162	7. 1897E-04	0. 4583	4. 9763E-04	4. 2735	1. 0426E-04
BdbZIP89	0. 1646	3. 0522E-01	0. 6825	3. 0069E-01	1. 8282	5. 3697E-03	2. 0837	1. 2008E-02
BdbZIP90	0. 1878	3. 1036E-01	1. 2230	7. 0848E-03	2. 0548	1. 6214E-03	2. 6557	2. 9300E-03
BdbZIP91	0. 1697	3. 2579E-01	0. 4602	3. 6554E-03	1. 6624	6. 4455E-03	1. 4002	8. 3926E-02
BdbZIP92	0. 1665	3. 2336E-01	1. 4299	4. 1622E-03	0. 6496	1. 2334E-01	1. 2194	2. 6059E-01
BdbZIP93	0. 0514	1. 0560E-05	0. 2523	7. 0221E-05	0. 6070	3. 2203E-02	0. 5198	1. 8769E-03
BdbZIP94	3. 3942	8. 6109E-08	2. 7672	9. 6575E-05	0. 6053	7. 8519E-03	7. 6580	7. 9542E-06
BdbZIP95	0. 4603	1. 6308E-04	2. 2098	9. 6550E-06	2. 6412	8. 0780E-05	3. 9632	2. 4996E-04
BdbZIP96	0. 3342	2. 5313E-03	1. 1271	5. 4067E-03	5. 2847	6. 4618E-05	5. 3790	2. 4759E-05

**Treatments**	**H2O2**				**PEG**			
	1h		6h		1h		6h	
**Genes**	fold change	p-value	fold change	p-value	fold change	p-value	fold change	p-value
BdbZIP1	0. 0012	1. 6629E-06	0. 0231	1. 5851E-05	0. 0048	2. 5799E-07	0. 1256	1. 9046E-05
BdbZIP2	0. 0475	1. 3684E-04	0. 1192	2. 8780E-04	0. 1514	8. 3955E-05	0. 1808	1. 7355E-04
BdbZIP3	0. 0506	3. 0216E-05	0. 4438	3. 9151E-01	0. 3540	8. 4724E-03	0. 8908	6. 7656E-02
BdbZIP4	0. 2176	3. 2630E-01	0. 8076	2. 2006E-03	0. 2321	6. 7673E-04	0. 8989	9. 2755E-02
BdbZIP5	0. 1286	6. 7350E-03	0. 1529	2. 5308E-04	0. 1654	4. 2469E-04	0. 2878	4. 3195E-04
BdbZIP6	0. 4289	1. 1616E-03	0. 2016	9. 7478E-04	0. 2028	1. 8922E-04	0. 2496	3. 5836E-04
BdbZIP7	1. 1055	1. 0478E-04	0. 9454	4. 4324E-03	1. 4902	5. 9761E-04	1. 4644	5. 3635E-03
BdbZIP8	0. 4340	1. 8689E-03	1. 1386	9. 6282E-04	0. 4328	2. 9935E-04	1. 3110	4. 6607E-02

BdbZIP9	0.1420	1.8570E-02	0.2886	8.7774E-03	0.1548	2.5752E-04	0.6941	3.4414E-01
BdbZIP10	1.4328	5.2165E-05	2.2535	2.0279E-04	1.7082	7.6774E-04	4.7619	1.6380E-03
BdbZIP11	0.1498	4.6510E-02	0.2011	4.5195E-03	0.3154	1.4060E-02	0.3848	2.9698E-03
BdbZIP12	0.1848	2.7954E-01	0.9503	1.9028E-03	0.2792	4.3094E-03	2.0689	1.0256E-04
BdbZIP13	0.2529	9.4728E-02	0.3228	4.2744E-02	0.1838	1.5584E-04	0.5497	4.8502E-02
BdbZIP14	3.6089	3.7750E-05	2.3743	5.8728E-05	2.1252	4.1586E-04	3.8132	2.4241E-04
BdbZIP15	0.1340	1.0393E-03	0.1410	4.7296E-04	0.2088	5.8624E-04	0.3760	2.1710E-03
BdbZIP16	0.2295	2.2282E-01	0.8255	1.5653E-03	0.2786	1.5558E-03	1.8884	1.4832E-03
BdbZIP17	0.0739	1.5870E-03	0.3357	1.2948E-01	0.2177	4.9039E-03	1.8915	3.6094E-03
BdbZIP18	0.5827	2.1169E-03	0.9836	5.5960E-03	0.9748	3.8491E-03	2.0173	2.4760E-03
BdbZIP19	0.3952	5.1438E-03	0.7062	1.1629E-02	0.3805	2.8644E-03	0.7126	3.9820E-01
BdbZIP20	0.3647	5.2426E-03	0.5873	6.0486E-02	0.3436	6.3916E-02	0.9789	4.1427E-02
BdbZIP21	0.2632	1.2124E-01	0.8284	4.0068E-03	0.3660	1.1131E-01	0.6291	2.1012E-01
BdbZIP22	1.3640	2.3157E-05	1.0483	4.7687E-04	1.4295	1.8748E-04	1.9608	3.8244E-04
BdbZIP23	0.3791	6.0555E-03	1.0569	1.0199E-04	0.4304	7.3438E-02	0.9576	3.4233E-02
BdbZIP24	0.0505	7.6175E-05	0.1007	3.9933E-04	0.0868	3.2470E-05	0.1856	2.9162E-04
BdbZIP25	0.9988	5.0925E-04	1.3862	1.8948E-03	1.2292	3.2651E-03	1.3484	2.3531E-02
BdbZIP26	0.1893	1.3543E-01	0.3595	1.1165E-01	0.2853	2.4299E-03	0.3689	1.8908E-03
BdbZIP27	1.3700	5.3011E-05	1.2145	7.3232E-04	1.9041	1.4427E-04	2.1351	2.8027E-04
BdbZIP28	0.1259	1.5128E-05	1.8697	7.0731E-03	0.3662	3.1381E-04	2.9033	7.5773E-03
BdbZIP29	2.5849	1.4104E-02	3.7315	3.0810E-05	1.2568	2.5546E-02	8.0440	3.0245E-05
BdbZIP30	0.2192	2.6962E-07	0.2754	1.5155E-06	0.4127	5.0453E-04	0.4044	2.0049E-05
BdbZIP31	0.1390	2.9423E-01	0.5115	2.4090E-02	0.1079	1.9695E-01	0.8660	1.5793E-02
BdbZIP32	0.5324	7.3420E-03	0.8495	2.9023E-02	0.9296	9.4366E-06	1.8330	8.2482E-02
BdbZIP33	0.1761	1.1362E-03	0.4155	5.4642E-04	0.6328	2.0703E-03	0.9671	8.5324E-04
BdbZIP34	0.3666	1.0527E-01	0.6691	3.7558E-01	0.9267	4.8420E-02	3.0430	5.9401E-02
BdbZIP35	0.4294	1.6122E-03	0.6835	1.9116E-02	0.5447	1.1307E-02	0.8508	3.8974E-04
BdbZIP36	0.1481	2.5721E-03	0.3547	2.4134E-02	0.5511	3.6770E-02	0.4774	7.2005E-02
BdbZIP37	0.0134	5.4464E-02	0.5629	1.5812E-01	0.3417	1.0672E-01	0.6170	1.7822E-02
BdbZIP38	0.1362	3.3690E-05	0.2896	7.7854E-03	0.1685	3.5570E-02	0.4532	5.5441E-02
BdbZIP39	0.0985	2.9895E-03	0.2945	2.4368E-02	0.3113	1.0444E-03	0.3158	1.9956E-03
BdbZIP40	1.5733	3.9009E-03	0.8684	2.0189E-02	1.2348	1.2649E-02	1.1795	3.1581E-03
BdbZIP41	0.3889	2.5622E-04	0.7454	4.5318E-04	0.3025	9.4451E-03	0.6867	1.8554E-01
BdbZIP42	0.0575	1.6172E-04	0.3239	7.6259E-02	0.1753	3.8746E-04	0.4367	5.8350E-03
BdbZIP43	1.1721	1.7701E-05	0.6759	7.4119E-03	1.1515	2.2917E-04	2.0776	7.7934E-04
BdbZIP44	0.0467	9.0742E-04	0.0558	1.6352E-04	0.1662	9.8191E-04	0.1534	3.4721E-04
BdbZIP45	0.1859	2.3930E-01	0.4503	3.1433E-01	0.5064	1.6947E-01	0.4947	4.5922E-04
BdbZIP46	0.2262	3.1189E-01	0.5336	1.3731E-01	0.3164	4.3997E-02	0.5481	1.1511E-01
BdbZIP47	0.1277	1.6366E-02	0.5999	2.6322E-02	0.1984	1.7220E-03	0.9359	5.4321E-02
BdbZIP48	0.4961	4.6957E-04	0.9955	8.3226E-04	1.2127	7.0018E-04	1.7411	1.8914E-04
BdbZIP49	0.1603	7.2967E-02	1.1797	7.5870E-04	1.0322	3.2398E-03	1.4017	3.0818E-03
BdbZIP50	0.0182	6.9566E-06	0.0703	5.0349E-06	0.0645	6.6881E-05	0.1165	7.9079E-06
BdbZIP51	0.3478	2.8469E-01	0.6031	4.1762E-02	0.1653	1.5177E-03	0.9363	7.6495E-02
BdbZIP52	0.8886	1.9522E-05	2.4039	4.9328E-05	1.2185	1.0978E-04	2.5972	1.8546E-05
BdbZIP53	0.1469	6.6413E-02	0.2775	9.6362E-03	0.3064	4.8957E-03	0.4513	8.0759E-03
BdbZIP54	0.2688	1.2976E-01	0.3959	3.0223E-01	0.2929	1.7506E-02	0.3492	3.5655E-03
BdbZIP55	0.3503	1.2377E-03	0.9159	2.7692E-05	0.4755	4.5773E-01	0.9538	8.7988E-04
BdbZIP56	0.1165	3.0593E-02	0.6700	2.4563E-02	0.2869	1.0072E-02	0.5952	1.4844E-01
BdbZIP57	0.2353	2.1248E-01	0.7529	6.9796E-03	0.6544	1.0175E-02	1.4097	1.6028E-03
BdbZIP58	0.3766	4.1719E-03	0.4235	4.6350E-01	0.5106	3.4477E-01	0.8462	2.2195E-01
BdbZIP59	0.1420	6.3791E-02	1.1504	3.0365E-03	0.2536	6.5844E-03	3.9356	1.2850E-04
BdbZIP60	0.0503	3.0212E-04	0.2887	2.2861E-02	0.2017	1.4774E-04	0.3722	1.2681E-03
BdbZIP61	0.8608	1.9855E-04	1.9069	3.2528E-04	1.5943	4.4238E-04	3.8672	7.0112E-05
BdbZIP62	0.5490	1.4790E-04	1.0015	1.8031E-04	1.5641	1.8472E-05	1.3874	3.6557E-03
BdbZIP63	0.1425	5.1459E-02	0.0811	3.9466E-04	0.2270	1.4829E-03	0.1026	1.4114E-04
BdbZIP64	0.3095	2.3698E-02	0.5891	2.6316E-02	0.3705	7.2325E-02	2.3541	1.2023E-03

BdbZIP65	0.2648	7.3553E-02	0.3635	1.4102E-01	0.4685	4.9159E-01	0.4691	1.5295E-02
BdbZIP66	1.1222	9.1439E-05	1.5749	6.1752E-04	0.3934	1.2866E-01	1.2129	1.0404E-02
BdbZIP67	0.1518	1.2787E-02	0.2741	3.1831E-03	0.2984	6.8330E-03	0.3636	4.9692E-04
BdbZIP68	0.0623	6.4420E-04	0.0975	1.3655E-04	0.1186	2.9508E-04	0.1689	1.7319E-04
BdbZIP69	0.1925	3.6475E-01	0.4205	4.2835E-01	0.3315	2.0446E-02	0.5738	9.3188E-02
BdbZIP70	0.2495	6.8802E-02	0.4983	1.4887E-01	0.3931	1.0881E-01	0.5948	1.1344E-01
BdbZIP71	0.3536	3.2937E-03	0.7016	1.9063E-02	0.3937	5.8775E-02	0.5333	5.1819E-02
BdbZIP72	0.2787	3.9282E-02	1.2334	1.5181E-03	0.1820	4.9436E-04	1.3664	6.1792E-03
BdbZIP73	0.4984	2.0968E-03	0.4881	1.9800E-01	0.4208	1.7843E-01	0.7377	4.3456E-01
BdbZIP74	0.0996	1.1726E-02	0.1560	1.0502E-03	0.3504	7.3931E-02	0.3720	5.8104E-03
BdbZIP75	0.1548	3.9860E-02	0.0112	1.0172E-07	0.1503	9.0402E-06	0.0255	3.7098E-06
BdbZIP76	0.3827	2.7453E-03	0.9407	6.8089E-04	3.3119	4.1945E-06	2.6475	9.3911E-06
BdbZIP77	0.3222	1.4087E-03	0.3402	8.2780E-02	0.3234	3.1305E-03	0.4286	4.8061E-03
BdbZIP78	0.1750	9.3081E-06	0.7473	1.3295E-08	0.3160	1.6244E-01	1.0879	5.8692E-07
BdbZIP79	0.0655	1.8079E-01	0.1667	5.4980E-03	0.1276	4.8498E-04	0.4978	1.7077E-03
BdbZIP80	0.0473	8.0943E-04	0.3820	2.2701E-01	0.9555	9.9596E-03	0.6036	1.4571E-01
BdbZIP81	0.0252	1.9745E-04	0.1568	2.8868E-03	0.1925	3.2747E-03	0.1918	1.7017E-03
BdbZIP82	0.3431	1.7733E-02	0.3761	1.9595E-01	0.8843	3.1710E-03	0.2688	5.3651E-04
BdbZIP83	0.4730	4.5438E-03	0.9295	3.1638E-03	0.7322	1.6857E-02	0.8835	1.1941E-01
BdbZIP84	1.0398	7.3883E-05	1.2299	7.4358E-04	1.6560	2.0827E-04	1.1436	1.3580E-02
BdbZIP85	0.0855	1.1164E-05	1.2577	7.0282E-04	1.3847	4.5622E-05	2.4646	1.1114E-05
BdbZIP86	0.2675	5.7784E-02	0.3143	1.5190E-02	0.4736	4.2182E-01	0.5877	2.8959E-02
BdbZIP87	0.0229	9.0021E-05	0.2257	9.2881E-04	0.1744	4.6200E-05	0.8212	6.8806E-02
BdbZIP88	1.8367	7.1282E-05	2.0079	6.7554E-05	0.7462	1.1629E-03	2.3809	5.3269E-05
BdbZIP89	0.3998	3.6163E-03	0.3900	2.8448E-01	0.3614	5.6393E-02	0.5803	9.8137E-02
BdbZIP90	0.1739	1.3620E-01	0.4391	4.7843E-01	0.2264	8.4220E-04	0.7309	4.8410E-01
BdbZIP91	0.1322	1.7404E-02	0.4868	2.2415E-01	0.2940	1.3429E-03	0.7365	4.8004E-01
BdbZIP92	0.3690	5.6157E-03	0.6631	4.3487E-02	0.9341	1.1628E-02	1.1151	3.0647E-02
BdbZIP93	0.1442	3.2475E-02	0.1834	2.3218E-03	0.2308	2.6658E-03	0.2235	2.0799E-05
BdbZIP94	1.5416	2.2477E-05	1.4690	2.4041E-06	0.9490	9.7313E-05	2.8762	1.4909E-07
BdbZIP95	0.5930	1.2539E-04	1.2369	5.3341E-05	0.8297	5.6441E-05	1.8872	7.9697E-04
BdbZIP96	0.4890	2.5859E-04	0.7407	2.5563E-03	1.7417	1.0459E-04	1.8101	1.3394E-03

Treatments	NaCl				Cu			
	1h		6h		6h		24h	
Genes	fold change	p-value	fold change	p-value	fold change	p-value	fold change	p-value
BdbZIP1	0.1471	6.6610E-06	0.5740	2.0856E-01	0.3925	4.4858E-03	0.5591	7.2597E-06
BdbZIP2	0.6633	4.1591E-05	0.1121	1.6325E-04	1.2671	5.6498E-05	0.6270	8.7674E-09
BdbZIP3	1.8046	9.3335E-03	1.0773	1.3843E-03	0.8206	3.7448E-03	0.5153	3.1182E-04
BdbZIP4	0.5435	2.5731E-05	0.9508	1.8892E-02	0.2566	3.4680E-06	0.3218	6.6440E-05
BdbZIP5	1.1589	2.3559E-04	0.2256	6.5379E-04	0.7550	3.0990E-02	0.5578	5.4555E-09
BdbZIP6	0.4999	1.7653E-04	0.1443	1.4015E-05	1.0445	2.7570E-03	1.2406	7.9216E-02
BdbZIP7	5.9771	9.1238E-03	1.6411	2.2646E-03	0.7077	3.8295E-02	0.5508	3.3425E-05
BdbZIP8	2.9621	3.1473E-04	0.8380	3.4947E-03	1.1948	1.0577E-03	1.2036	4.6456E-03
BdbZIP9	0.9504	2.7913E-04	0.2926	9.1888E-04	1.3963	4.2306E-04	1.3188	2.3942E-02
BdbZIP10	2.9168	4.6260E-01	1.7351	5.5864E-04	0.3860	8.8366E-03	1.0280	1.2106E-02
BdbZIP11	1.5633	3.1651E-03	0.2270	1.1293E-03	0.8606	7.6281E-04	0.4712	4.7211E-06
BdbZIP12	1.6953	9.2411E-04	1.0032	1.7941E-02	0.9656	8.7635E-03	0.5090	4.8010E-04
BdbZIP13	0.9816	3.1888E-05	0.6313	4.2820E-01	0.8433	4.1999E-03	0.3595	1.9774E-06
BdbZIP14	1.2912	1.3498E-03	2.7753	1.7578E-04	0.2127	1.4167E-06	0.2443	8.9725E-08
BdbZIP15	1.3177	8.0822E-05	0.2465	4.2101E-04	1.8422	6.1338E-04	1.2821	1.1043E-01
BdbZIP16	1.6521	7.8148E-04	0.8196	6.2553E-02	0.9125	1.7596E-03	0.1487	2.1577E-06
BdbZIP17	1.9085	2.7725E-02	0.2427	3.8591E-03	2.8401	4.0768E-06	7.5323	4.1721E-08
BdbZIP18	7.8411	2.1293E-03	3.2117	1.3035E-04	0.5844	4.9356E-01	0.2501	4.7800E-07
BdbZIP19	1.5881	4.9492E-04	0.7265	1.1597E-01	2.0548	4.1764E-04	1.5030	4.5348E-01
BdbZIP20	1.8902	1.6803E-02	0.6433	4.9869E-01	0.5681	2.1702E-01	0.4058	6.3629E-07
BdbZIP21	2.2324	8.8987E-02	0.5804	2.4650E-01	0.2998	7.8635E-04	0.4555	3.4378E-06

BdbZIP22	6.1978	7.6134E-04	1.9391	2.7805E-04	0.7194	1.2631E-03	0.4807	2.7444E-04
BdbZIP23	4.1222	2.5921E-02	0.8645	2.2123E-03	0.6629	1.1158E-01	0.2412	9.5311E-07
BdbZIP24	0.6923	1.1701E-04	0.1498	9.8254E-05	1.1678	1.0621E-04	0.6443	8.5233E-05
BdbZIP25	1.0875	5.3314E-03	1.0930	2.9215E-02	0.7338	9.6393E-02	0.2162	3.5360E-05
BdbZIP26	1.3728	5.2697E-05	0.3988	7.6973E-03	0.9618	8.0802E-05	0.6626	4.6528E-04
BdbZIP27	7.4235	5.8788E-05	2.3906	3.8134E-04	0.8608	1.3573E-02	0.5097	3.5288E-04
BdbZIP28	1.7989	3.6735E-01	1.3008	4.2751E-05	0.0426	7.2110E-08	0.1126	2.7609E-03
BdbZIP29	14.8203	6.8961E-03	11.3297	7.9692E-04	1.2440	8.1162E-06	1.1324	5.7780E-06
BdbZIP30	2.0816	1.9355E-04	0.3385	1.2031E-06	0.6209	1.3663E-05	0.4914	3.1645E-02
BdbZIP31	0.5245	3.4695E-02	0.6468	1.0879E-02	1.0215	1.6636E-01	0.1904	2.6508E-05
BdbZIP32	6.0797	5.6711E-06	0.5646	4.7424E-01	1.1305	9.0831E-06	1.0965	9.3718E-07
BdbZIP33	2.6458	1.7468E-03	0.4814	1.7176E-01	0.6342	7.1923E-05	0.4152	1.4879E-02
BdbZIP34	5.7643	1.6192E-01	0.9724	5.3611E-02	3.1866	1.8246E-01	3.4948	6.3798E-06
BdbZIP35	2.7149	7.4998E-04	0.7659	1.0501E-02	0.7931	3.7681E-06	0.5804	5.7357E-08
BdbZIP36	2.5927	1.8279E-01	0.2477	4.6046E-02	0.3667	1.2758E-03	0.1510	6.0982E-06
BdbZIP37	11.0740	1.5341E-01	0.3340	5.7118E-04	0.1638	8.2230E-03	0.4406	4.8695E-07
BdbZIP38	1.2222	7.5908E-05	0.3493	1.2899E-03	4.3552	6.9967E-07	2.1329	2.7946E-04
BdbZIP39	2.2130	6.2932E-04	0.4888	2.1245E-03	1.0170	9.0047E-06	0.2289	9.2827E-04
BdbZIP40	2.8174	7.1424E-02	3.4403	3.9132E-02	3.1543	2.8565E-04	1.8080	1.3618E-06
BdbZIP41	2.0291	1.0934E-02	0.3472	1.0789E-03	3.9488	1.8581E-07	1.1864	1.7424E-02
BdbZIP42	0.5885	5.0047E-05	0.2106	1.5018E-03	1.9066	4.0258E-08	0.9208	6.0519E-05
BdbZIP43	10.4401	2.3546E-04	1.8758	7.5759E-04	0.8077	5.1756E-02	0.6926	4.1999E-03
BdbZIP44	1.1248	6.3811E-03	0.1396	5.3424E-04	0.7201	1.6295E-01	0.5297	7.9357E-04
BdbZIP45	2.9368	4.7199E-01	0.5804	2.2777E-01	0.6378	1.6908E-01	0.6015	1.1585E-06
BdbZIP46	1.6786	1.9239E-02	0.5195	1.7228E-01	0.8900	1.5278E-03	0.4854	3.8690E-06
BdbZIP47	1.7390	1.3274E-02	0.4262	1.4952E-02	1.5780	4.4765E-05	1.4648	4.4042E-01
BdbZIP48	3.9740	5.1664E-02	0.3744	7.1639E-04	0.2272	1.5961E-04	0.7707	7.1101E-06
BdbZIP49	2.6701	2.5082E-01	0.3958	1.7696E-02	0.0956	3.5164E-05	0.4616	1.2037E-04
BdbZIP50	0.3155	1.5266E-05	0.1244	6.6457E-05	1.2808	3.3196E-06	0.2690	7.6892E-10
BdbZIP51	1.2153	3.2596E-03	0.5725	2.4617E-01	1.3197	3.1434E-04	0.4720	2.2442E-04
BdbZIP52	1.3424	5.9914E-05	60.2007	3.0613E-02	0.1173	5.1322E-07	0.1610	3.0143E-05
BdbZIP53	1.9333	8.6712E-03	0.4654	3.2933E-02	0.5708	3.4805E-01	0.4285	2.3452E-06
BdbZIP54	3.4234	2.1258E-01	0.3480	8.1635E-03	0.8251	1.4113E-02	1.4690	4.7151E-01
BdbZIP55	2.3463	1.0319E-02	0.5308	3.5201E-02	0.4822	4.1147E-04	0.5966	6.1401E-06
BdbZIP56	0.9657	7.2311E-04	0.3579	1.2631E-02	0.6406	2.2558E-01	0.6361	2.9338E-04
BdbZIP57	3.4721	1.0223E-01	0.9626	4.3155E-02	0.9544	1.1852E-03	0.4613	1.0604E-04
BdbZIP58	2.5532	2.1413E-01	0.8144	7.9841E-02	0.0638	2.0360E-05	0.9404	9.9683E-03
BdbZIP59	2.2326	8.1804E-02	0.6326	4.6346E-01	0.3560	9.7715E-03	1.1757	1.7801E-04
BdbZIP60	0.4712	8.0233E-05	0.2044	1.0690E-03	2.0099	1.4537E-07	13.4960	2.1985E-06
BdbZIP61	12.0466	6.3776E-05	2.2556	1.1117E-04	0.5200	1.1262E-01	0.1393	4.7300E-07
BdbZIP62	2.1361	2.7963E-02	0.9494	1.7901E-02	1.0796	2.8116E-05	3.5453	1.4969E-03
BdbZIP63	1.0200	4.3025E-04	0.1075	1.1723E-04	0.5005	6.0756E-02	0.3751	7.8692E-06
BdbZIP64	2.9330	4.7393E-01	1.3899	7.9010E-03	0.1931	4.8623E-04	0.1272	1.3130E-05
BdbZIP65	1.6778	4.1932E-03	0.3776	1.2149E-02	1.1070	8.2771E-07	0.6334	2.5071E-05
BdbZIP66	6.6035	2.5843E-01	0.6906	3.0306E-01	0.2786	7.2350E-04	0.9937	2.4568E-03
BdbZIP67	1.4158	1.6633E-03	0.2809	1.9115E-04	0.4181	1.6014E-03	0.3316	6.2760E-05
BdbZIP68	0.4473	2.7568E-04	0.1138	6.0912E-05	0.6016	4.3193E-01	0.1374	1.2732E-05
BdbZIP69	1.3806	1.7457E-03	0.4482	4.1616E-02	0.5297	2.4866E-02	0.3988	5.2709E-06
BdbZIP70	2.1599	2.3501E-02	0.6059	3.3831E-01	0.6545	4.6851E-02	0.3897	7.9001E-06
BdbZIP71	1.0974	1.1893E-04	0.6147	3.2689E-01	0.5573	3.6244E-01	0.2195	1.2466E-05
BdbZIP72	0.9547	2.3918E-04	0.4659	2.0530E-02	1.3723	3.3949E-05	0.9291	1.0733E-05
BdbZIP73	1.9235	1.2813E-02	1.0596	9.5559E-03	1.1748	1.2375E-03	0.1677	1.7777E-05
BdbZIP74	2.2167	9.3141E-02	0.3811	2.0300E-02	0.6627	2.3292E-01	0.2360	9.4210E-05
BdbZIP75	1.6359	2.7802E-03	0.0299	1.4278E-06	1.2686	2.6296E-05	1.8689	1.1125E-03
BdbZIP76	4.0163	5.9458E-02	3.3487	1.2457E-05	1.7411	9.3656E-04	1.5324	4.2825E-01
BdbZIP77	1.9864	2.5587E-02	0.4333	1.0270E-02	0.8586	2.5530E-02	0.3909	1.1549E-05

BdbZIP78	2.1416	4.4648E-01	0.6683	7.7168E-05	0.6373	7.9550E-02	0.4373	1.8451E-05
BdbZIP79	0.6798	5.7743E-03	0.3131	4.0094E-01	0.7192	6.2417E-04	0.4056	2.3976E-06
BdbZIP80	2.1229	6.5646E-02	0.1638	3.7065E-04	0.3393	1.3187E-02	0.5500	2.9584E-04
BdbZIP81	0.8151	1.0275E-03	0.0910	1.8393E-04	0.1229	2.4439E-06	0.6817	1.5200E-04
BdbZIP82	1.9318	1.1574E-02	0.3447	8.4386E-03	0.7101	1.0271E-01	0.3306	9.3719E-09
BdbZIP83	3.0032	4.4756E-01	0.5550	2.1770E-01	0.9944	3.1671E-04	1.0502	3.1333E-05
BdbZIP84	3.8434	4.5360E-02	0.6907	3.2251E-01	0.7997	1.6473E-03	0.5346	7.2663E-06
BdbZIP85	2.1962	2.6492E-02	0.4744	1.0517E-02	0.1957	6.6456E-04	0.6023	2.0539E-03
BdbZIP86	2.4069	3.9066E-02	0.4530	3.0245E-02	0.9314	4.8463E-03	0.5263	2.4659E-04
BdbZIP87	0.4452	4.8621E-06	0.3440	2.6295E-03	1.0706	1.9484E-04	0.4485	5.4080E-04
BdbZIP88	4.2753	1.1426E-03	1.1062	2.2665E-04	0.9225	5.2297E-03	0.1604	6.9360E-05
BdbZIP89	2.0507	2.8135E-02	0.4955	1.0081E-01	0.5819	4.8050E-01	0.4093	2.6797E-04
BdbZIP90	0.9727	3.0793E-04	0.4698	6.2373E-02	0.7626	1.1037E-03	0.5013	1.9900E-05
BdbZIP91	1.8613	1.2504E-03	0.5568	1.5744E-01	0.9702	1.3118E-02	0.5528	3.8625E-04
BdbZIP92	1.3648	2.3080E-03	0.6529	4.5150E-01	0.0491	1.3534E-08	0.1900	6.5780E-05
BdbZIP93	1.1051	1.5618E-04	0.2354	3.5562E-04	0.7024	3.3855E-02	0.3321	2.8520E-05
BdbZIP94	5.3076	4.8785E-03	1.7241	1.1566E-06	0.8702	4.1204E-03	0.4670	1.1088E-08
BdbZIP95	3.8548	2.6969E-02	1.6652	5.1511E-04	0.3317	4.4613E-04	0.5479	1.0364E-05
BdbZIP96	10.1136	7.3060E-04	1.0610	3.0669E-03	0.1476	5.4891E-09	0.5458	1.4766E-08

Treatments	Zn				Mn			
	6h		24h		6h		24h	
Genes	fold change	p-value	fold change	p-value	fold change	p-value	fold change	p-value
BdbZIP1	0.2509	2.4236E-06	1.0791	1.7354E-01	11.1492	4.7743E-06	6.7733	6.3927E-06
BdbZIP2	0.7378	6.7660E-05	0.8014	4.8983E-06	0.6482	8.5582E-06	0.1480	7.1809E-08
BdbZIP3	1.3079	3.0125E-01	1.8626	2.0618E-03	0.5363	1.1381E-03	0.4776	2.9885E-04
BdbZIP4	1.1504	5.9134E-02	1.1904	1.9692E-01	0.0702	4.1212E-07	0.1891	8.7823E-06
BdbZIP5	0.6150	4.8317E-08	0.7288	1.0496E-07	0.4323	1.1524E-05	0.2975	5.7889E-07
BdbZIP6	1.3123	2.6150E-01	1.7427	1.9066E-02	3.9128	9.2157E-05	1.7816	3.0442E-03
BdbZIP7	0.8661	2.4000E-03	0.7539	2.0941E-03	0.6722	1.0901E-03	0.3909	4.3046E-05
BdbZIP8	1.1102	3.5171E-03	0.9189	2.0213E-03	0.2016	1.4917E-06	0.9044	5.4387E-02
BdbZIP9	1.1804	4.4756E-02	0.9607	2.6128E-02	0.9280	2.2819E-01	0.9790	3.2949E-01
BdbZIP10	0.6759	3.1421E-03	0.8690	3.5731E-02	3.1334	2.9775E-04	2.1991	8.9195E-04
BdbZIP11	0.5891	1.0656E-05	0.5589	1.0789E-04	0.4623	1.0196E-04	0.2460	2.2884E-06
BdbZIP12	0.8653	7.8159E-03	0.4536	6.9014E-05	0.7758	8.0368E-02	0.3710	6.0658E-05
BdbZIP13	0.4502	3.3509E-06	0.3532	1.5477E-05	0.3710	1.5001E-04	0.1366	3.2986E-07
BdbZIP14	0.1400	4.0658E-07	0.3338	9.0918E-07	0.1301	5.4511E-08	0.3227	5.8282E-07
BdbZIP15	0.7516	2.0246E-03	0.3586	5.3106E-04	1.4764	3.0160E-02	0.8745	1.3390E-01
BdbZIP16	0.6654	4.8061E-05	0.2010	4.7030E-07	0.3820	1.8490E-04	0.1678	6.8039E-06
BdbZIP17	7.9443	4.4433E-06	17.7486	1.9130E-08	2.5096	3.0284E-06	0.9753	3.8559E-01
BdbZIP18	0.3112	2.5477E-06	0.7899	7.6449E-04	0.3418	1.4165E-05	0.2711	2.4740E-05
BdbZIP19	0.7415	6.2966E-04	0.6232	1.2668E-03	0.1719	2.1722E-05	0.1457	8.2356E-06
BdbZIP20	0.6844	4.5669E-06	0.6184	4.6348E-07	0.5459	1.6587E-08	0.0840	1.9036E-10
BdbZIP21	0.9195	3.6755E-04	0.6743	1.0432E-04	0.8073	4.3244E-03	0.4164	3.3087E-04
BdbZIP22	0.7751	5.1523E-04	0.9501	8.0527E-03	0.3261	5.3941E-06	0.1818	3.5443E-05
BdbZIP23	0.4334	3.7614E-05	0.6944	4.7374E-04	0.2283	2.0035E-06	0.1483	9.8201E-06
BdbZIP24	0.5228	2.3642E-04	0.4501	6.2882E-05	0.4827	1.2132E-03	0.0661	2.0680E-07
BdbZIP25	0.3844	1.6575E-04	0.1467	2.3979E-05	0.3467	8.1173E-04	0.3687	4.2685E-04
BdbZIP26	0.9066	4.1306E-03	0.8446	2.6343E-04	0.5970	4.5903E-04	0.5355	1.0215E-05
BdbZIP27	0.9679	1.6043E-02	1.1100	1.8072E-01	0.9811	4.4451E-01	0.3759	7.2477E-04
BdbZIP28	0.1051	1.7173E-03	0.1463	5.6135E-03	0.1202	6.1013E-02	0.1428	2.7373E-03
BdbZIP29	1.8025	6.4858E-06	1.8790	1.0955E-05	0.6226	1.8456E-05	1.2849	1.1514E-04
BdbZIP30	0.6386	7.7385E-04	0.6473	2.0957E-04	0.5229	2.8914E-04	0.3370	9.1321E-03
BdbZIP31	0.5927	2.3300E-07	0.4534	5.2077E-07	0.4759	3.1372E-04	0.3812	2.4237E-07
BdbZIP32	1.1774	9.3962E-07	1.2697	8.3591E-05	0.9003	5.7402E-04	0.5432	6.2489E-07
BdbZIP33	0.4715	6.9552E-03	0.5444	4.5246E-01	0.3619	2.0161E-01	0.2849	1.9168E-04
BdbZIP34	0.9397	5.9053E-05	2.1110	1.5429E-05	0.2382	1.1395E-04	0.2306	4.1952E-05

BdbZIP35	0.6776	1.7493E-04	0.6435	1.3288E-04	0.6511	4.5020E-05	0.4118	1.8549E-04
BdbZIP36	0.2967	3.9616E-06	0.4587	1.2266E-04	0.3481	1.6720E-03	0.2044	1.3730E-06
BdbZIP37	0.1465	1.4525E-05	0.5418	1.3173E-06	0.6362	4.6142E-05	0.1729	1.5498E-07
BdbZIP38	2.1388	2.6788E-05	2.5576	6.2157E-07	2.3204	1.5631E-03	0.9332	1.2687E-09
BdbZIP39	0.3813	2.3092E-03	0.6414	1.4934E-03	0.4014	1.5876E-04	0.1096	2.6710E-01
BdbZIP40	2.0210	3.0892E-06	0.9217	4.1568E-03	1.3651	8.1884E-05	1.2795	1.2071E-06
BdbZIP41	1.2595	4.0691E-02	1.6758	4.9803E-03	1.7749	7.3773E-03	1.4080	2.8716E-02
BdbZIP42	0.6883	6.1545E-04	0.8619	2.0002E-06	0.5047	4.7148E-04	0.5193	3.9227E-07
BdbZIP43	1.2032	2.1100E-01	1.3170	3.7939E-01	0.8999	2.6354E-01	0.8615	2.1614E-01
BdbZIP44	1.4647	3.9821E-01	1.5044	1.6458E-01	0.2098	1.5982E-04	0.1027	4.4464E-05
BdbZIP45	0.6508	1.9466E-07	0.7152	1.7048E-04	0.5886	9.5142E-04	0.4475	4.6644E-06
BdbZIP46	0.6789	2.4491E-05	0.6124	6.1250E-04	0.5389	7.4035E-05	0.3141	5.4810E-05
BdbZIP47	0.9032	1.2080E-05	0.5242	6.9556E-06	0.7462	2.5634E-05	0.3158	1.1881E-04
BdbZIP48	1.5723	6.9323E-02	1.6805	9.0510E-04	2.5687	2.5344E-04	1.6868	3.8231E-05
BdbZIP49	0.9532	2.6892E-02	1.0929	1.4556E-01	1.8370	1.1533E-03	1.8603	1.3635E-03
BdbZIP50	0.4065	1.2299E-08	1.5438	4.1483E-02	13.8520	1.1617E-05	8.4900	6.6532E-10
BdbZIP51	0.3497	1.8545E-05	0.1693	1.3748E-05	0.8355	3.1277E-02	0.6776	3.3675E-03
BdbZIP52	1.1097	1.0747E-03	1.4227	5.7437E-02	0.3476	1.4190E-04	0.5303	2.0244E-03
BdbZIP53	0.3748	5.8192E-06	0.7834	1.1150E-03	0.2548	5.2237E-05	0.2004	8.5493E-07
BdbZIP54	2.2062	5.9776E-03	2.5300	1.3347E-03	2.4150	5.9040E-04	1.5882	5.5900E-03
BdbZIP55	1.0245	3.3720E-05	0.7509	9.2110E-04	0.6256	9.5848E-06	0.2723	4.8401E-05
BdbZIP56	0.8666	2.5686E-03	0.7205	1.9471E-03	0.5636	4.4596E-03	0.2491	4.7793E-05
BdbZIP57	0.5869	4.6573E-04	0.5636	1.1469E-04	0.5728	5.4825E-03	0.2703	1.8290E-08
BdbZIP58	0.1769	1.9258E-05	0.6036	1.0391E-03	0.9562	3.4825E-01	1.3822	1.6272E-02
BdbZIP59	1.5137	1.6926E-02	0.9744	1.9848E-04	1.4908	1.5769E-03	1.3892	2.2678E-04
BdbZIP60	7.4408	1.5422E-07	12.5215	2.3646E-07	1.8526	5.9295E-08	5.2009	2.3613E-05
BdbZIP61	0.3733	6.5721E-07	0.6001	7.4177E-06	0.2947	8.6413E-05	0.2082	3.6496E-07
BdbZIP62	3.3328	8.5512E-07	2.7492	2.5010E-05	2.8159	2.4495E-04	2.1982	1.3778E-04
BdbZIP63	0.6867	1.9058E-05	0.4369	6.3403E-06	0.5569	2.9853E-03	0.2209	3.3569E-07
BdbZIP64	0.1024	1.1222E-05	0.1017	1.3006E-05	0.0661	1.4582E-05	0.0535	2.2440E-05
BdbZIP65	0.2868	9.1304E-06	0.5047	6.6749E-07	0.5532	7.5422E-04	0.3097	3.1674E-05
BdbZIP66	1.1273	7.5501E-03	1.5063	1.1278E-01	0.1548	5.3251E-07	0.9703	4.2559E-01
BdbZIP67	0.2598	3.0526E-06	0.2914	1.6288E-05	0.2297	1.7499E-04	0.1131	1.1336E-06
BdbZIP68	0.5272	2.4716E-03	0.5795	1.1802E-03	0.5563	3.1005E-03	0.3431	4.7555E-04
BdbZIP69	0.4381	2.1114E-04	0.3494	1.5150E-06	0.4863	2.0464E-03	0.2984	4.0655E-06
BdbZIP70	0.5515	3.9960E-06	0.5142	7.0260E-06	0.4076	6.1748E-07	0.2166	3.7184E-06
BdbZIP71	0.3958	4.9867E-06	0.3978	1.1845E-06	0.1829	4.6378E-05	0.0669	1.0932E-07
BdbZIP72	1.0005	1.5577E-04	0.7080	1.4374E-06	0.8022	2.8254E-04	0.6895	2.4460E-04
BdbZIP73	0.1451	1.5140E-05	0.2111	2.9552E-05	0.3178	1.6775E-04	0.1007	6.1957E-05
BdbZIP74	0.8795	1.4632E-02	1.4632	1.9673E-01	0.7978	5.9868E-02	0.4719	2.0666E-03
BdbZIP75	0.2812	3.6062E-05	0.9731	2.8649E-03	0.1604	1.6206E-05	0.5736	1.3956E-05
BdbZIP76	0.8752	2.1325E-02	1.4661	1.5801E-01	1.2480	1.2841E-01	0.9314	3.5582E-01
BdbZIP77	0.5421	3.3152E-06	0.5558	2.9006E-05	0.3950	8.0451E-05	0.2020	8.0199E-06
BdbZIP78	0.5996	1.7893E-04	0.4892	2.9505E-05	0.4165	7.3466E-04	0.2468	2.8649E-05
BdbZIP79	0.5404	1.6592E-06	0.5003	5.7018E-05	0.4335	6.5018E-04	0.2594	2.2562E-07
BdbZIP80	1.4562	3.8215E-01	1.6796	1.8201E-02	0.8931	1.7845E-01	0.3404	1.7547E-04
BdbZIP81	1.3779	7.5958E-02	1.2374	4.3662E-01	1.5375	1.4691E-04	1.0301	1.9629E-01
BdbZIP82	0.5608	1.3837E-06	0.5526	1.1817E-06	0.3174	4.0792E-05	0.1276	1.8120E-06
BdbZIP83	1.5863	7.1378E-02	1.3922	5.5381E-02	0.4913	2.0424E-05	0.5715	1.6465E-04
BdbZIP84	0.4850	1.7642E-05	0.8794	7.0776E-03	0.3110	5.0854E-05	0.2272	8.5011E-05
BdbZIP85	2.1023	2.1687E-02	1.4068	2.3537E-01	1.6846	1.6438E-02	1.1437	2.0065E-01
BdbZIP86	0.7505	6.7032E-03	1.0279	9.6548E-02	0.7304	3.7616E-02	0.1061	1.2007E-05
BdbZIP87	0.7174	8.1441E-04	0.7965	8.6979E-04	0.3510	2.7591E-04	0.3535	3.8867E-05
BdbZIP88	0.2793	4.3031E-05	0.1694	4.9289E-05	0.1720	5.9058E-05	0.0803	7.0328E-06
BdbZIP89	0.5639	4.3279E-04	0.5842	1.0420E-03	0.3541	9.1137E-04	0.2151	7.6657E-05
BdbZIP90	0.6077	8.3592E-04	0.4062	4.3073E-06	0.5669	9.4617E-04	0.3730	6.4753E-06

BdbZIP91	0.7647	1.0476E-02	0.7416	1.2862E-02	0.5687	8.7323E-03	0.3576	2.6954E-04
BdbZIP92	0.0838	2.3483E-09	0.1047	9.0946E-11	0.0572	1.6031E-09	0.0827	1.5618E-05
BdbZIP93	0.1488	4.3618E-06	0.3020	3.1218E-06	0.0836	2.8965E-07	0.1151	3.8290E-06
BdbZIP94	0.5892	1.6735E-05	0.6354	3.2305E-05	0.4985	1.5725E-03	0.2893	2.5625E-06
BdbZIP95	0.5179	5.3477E-10	0.4540	2.7236E-06	0.1656	6.8545E-06	0.0836	1.7988E-07
BdbZIP96	0.2311	5.2337E-06	0.1952	1.0156E-09	0.6535	3.5709E-03	0.8146	2.8947E-03

Treatments	Cd				Pb			
	6h		24h		6h		24h	
Genes	fold change	p-value	fold change	p-value	fold change	p-value	fold change	p-value
BdbZIP1	11.4751	1.0042E-07	4.0699	4.1651E-07	4.6229	2.0013E-06	1.2482	1.9244E-02
BdbZIP2	1.9361	2.9392E-04	1.6753	2.2393E-09	0.4574	3.4195E-05	1.1937	1.4404E-01
BdbZIP3	10.5415	2.4435E-06	12.6698	1.6197E-06	0.5075	1.5864E-04	0.8899	1.7189E-01
BdbZIP4	0.0887	2.9581E-05	0.0957	6.1931E-08	0.0610	7.0029E-09	0.0597	9.1239E-06
BdbZIP5	0.5382	2.2547E-06	1.0337	2.7239E-04	0.2691	1.9045E-05	0.0777	2.1304E-08
BdbZIP6	3.4530	1.9129E-04	4.3062	6.5634E-05	0.1481	2.0482E-05	1.0558	3.5795E-01
BdbZIP7	0.6595	8.9761E-03	0.7002	4.2805E-01	0.3024	1.9707E-05	0.1860	1.7925E-06
BdbZIP8	9.5610	3.3824E-07	15.7212	1.9025E-06	0.3428	3.0200E-06	0.7870	4.3360E-02
BdbZIP9	8.8068	7.6948E-07	15.5556	2.5102E-07	0.3342	5.7610E-06	0.9867	4.2580E-01
BdbZIP10	0.3727	3.1790E-04	0.3449	1.0713E-03	7.1383	5.8370E-05	1.2699	4.1779E-02
BdbZIP11	1.2219	1.0806E-02	1.2759	6.3146E-05	0.2704	3.9069E-05	0.1709	6.7884E-07
BdbZIP12	4.2785	1.1589E-04	8.0905	1.8866E-06	0.6374	2.5025E-04	0.8819	6.8785E-02
BdbZIP13	0.5694	1.9728E-04	0.8565	3.6556E-03	0.4357	1.6549E-05	0.2908	4.4532E-06
BdbZIP14	2.2297	2.3067E-06	1.2428	8.9907E-05	0.8399	1.3683E-04	1.1476	1.5440E-02
BdbZIP15	1.2986	3.3167E-02	3.5843	4.1872E-05	0.6006	8.7520E-04	0.1263	1.7000E-05
BdbZIP16	0.4687	2.2554E-05	0.5945	9.1231E-02	0.1663	2.3075E-06	0.0737	2.2055E-08
BdbZIP17	29.0716	1.7194E-06	25.2290	4.4096E-12	5.3549	1.4006E-08	3.4122	8.6518E-07
BdbZIP18	0.1066	3.5439E-06	0.1311	7.9952E-05	0.8054	1.1053E-03	0.1525	1.2840E-07
BdbZIP19	2.2799	3.8985E-04	0.7899	1.4264E-01	1.8395	9.7956E-03	0.7572	1.6087E-02
BdbZIP20	0.9786	3.2338E-01	0.4473	2.4809E-07	0.3400	2.1084E-09	0.2218	6.9494E-05
BdbZIP21	1.8384	6.5867E-04	0.9003	3.0036E-02	6.6356	2.6574E-06	3.9245	3.9845E-04
BdbZIP22	0.5872	3.1816E-03	0.4697	2.4716E-03	0.0702	1.9769E-08	0.2736	2.1432E-05
BdbZIP23	0.1462	2.0147E-06	0.1050	1.4609E-06	0.1756	8.7973E-07	0.0532	5.2521E-07
BdbZIP24	1.7299	1.5501E-03	3.2022	6.8509E-05	0.7724	1.6666E-04	0.2030	1.6950E-06
BdbZIP25	13.4987	1.0246E-05	1.2292	1.0853E-02	7.5655	2.1831E-04	1.2130	1.8451E-01
BdbZIP26	0.9463	1.5493E-01	0.5635	2.0843E-02	0.1956	2.7919E-05	0.0942	1.0162E-07
BdbZIP27	0.6634	1.2140E-02	0.3352	1.4828E-03	0.3113	4.7593E-04	0.3952	6.1868E-04
BdbZIP28	0.3277	4.8997E-04	0.1689	4.3977E-06	0.1132	8.0072E-05	0.0628	1.7945E-06
BdbZIP29	2.3054	4.8068E-04	15.9310	4.9239E-05	0.7180	6.0447E-06	0.9077	3.5399E-06
BdbZIP30	0.4821	6.5402E-06	1.0836	8.5808E-08	0.1850	8.4567E-05	0.0897	2.4357E-02
BdbZIP31	1.0243	9.5236E-05	0.4945	8.2523E-10	1.4925	1.6353E-06	0.9515	1.1939E-07
BdbZIP32	3.3129	1.7576E-01	4.6990	1.6514E-03	0.5712	1.8748E-03	0.1488	2.7531E-01
BdbZIP33	0.8909	1.0758E-05	1.9305	7.5820E-07	0.2516	5.7472E-05	0.0569	1.0611E-06
BdbZIP34	20.7422	6.6068E-02	21.7810	2.2762E-07	8.1191	1.1370E-07	11.7852	4.5824E-09
BdbZIP35	0.9335	8.6619E-09	1.2094	2.2882E-09	0.2299	2.5084E-04	0.2186	3.1526E-07
BdbZIP36	0.1684	3.1521E-01	0.2122	1.0197E-05	0.3496	1.5039E-05	0.0821	2.7936E-06
BdbZIP37	1.5701	4.3548E-05	0.9045	4.5990E-07	0.8179	4.6869E-06	0.3774	8.1833E-06
BdbZIP38	3.9457	3.2152E-04	2.2647	3.3430E-02	2.2349	2.0955E-04	0.5554	6.6627E-06
BdbZIP39	1.7620	5.2017E-06	0.9835	7.5165E-04	1.4287	1.3070E-04	1.0231	1.7061E-04
BdbZIP40	2.0844	1.7256E-04	3.6365	4.8032E-02	1.7165	4.4845E-02	2.1231	4.1016E-01
BdbZIP41	3.1570	6.0769E-08	0.2687	1.9705E-07	0.4891	2.0278E-05	0.0821	1.1434E-06
BdbZIP42	0.5563	4.2325E-04	0.4623	2.5676E-04	0.3566	7.3915E-08	0.0858	3.8577E-06
BdbZIP43	0.0978	5.5007E-05	0.0969	2.1459E-04	0.1859	1.2103E-04	0.0574	2.4110E-05
BdbZIP44	0.1866	1.2504E-04	0.1491	3.2481E-04	0.3023	2.2678E-04	0.1200	7.9315E-05
BdbZIP45	0.4694	2.2938E-07	0.2833	1.7421E-04	0.1668	1.3127E-05	0.1074	1.7823E-05
BdbZIP46	1.1477	1.2445E-01	1.7980	9.9827E-06	0.6110	4.3839E-04	0.3625	2.9309E-06
BdbZIP47	14.0915	4.1317E-06	7.1292	2.1432E-08	7.3159	2.0770E-07	4.1089	4.3327E-11

BdbZIP48	1.0076	4.5485E-01	0.2377	1.1402E-06	0.2562	2.0377E-07	0.6686	1.2218E-04
BdbZIP49	0.1054	7.9242E-06	0.1407	1.5953E-05	0.3813	8.8154E-05	0.3837	1.2930E-04
BdbZIP50	0.4560	2.7748E-03	1.2562	2.0293E-06	0.2218	2.3239E-06	0.3292	1.8122E-05
BdbZIP51	5.8734	1.8917E-05	2.1239	4.6563E-05	1.1473	1.4711E-01	0.6443	2.4317E-03
BdbZIP52	6.9122	4.2403E-07	1.1616	1.1400E-04	3.0851	3.0907E-05	1.0947	2.3862E-01
BdbZIP53	0.7423	1.6509E-03	0.2785	6.8804E-06	0.5715	1.4284E-05	0.0669	1.0072E-07
BdbZIP54	6.6589	2.4312E-05	5.3763	8.5593E-05	1.2981	3.9025E-01	24.7256	2.0070E-05
BdbZIP55	1.1227	2.0890E-01	1.4361	3.3799E-06	0.0744	9.7790E-06	0.4847	1.2726E-06
BdbZIP56	0.8744	1.4721E-01	1.2426	1.5512E-03	0.1278	6.1745E-06	0.5554	2.0739E-03
BdbZIP57	0.7125	2.7722E-07	1.1899	7.2893E-07	0.8352	1.0769E-04	1.1979	3.7132E-03
BdbZIP58	19.0758	1.4714E-05	10.4851	3.1653E-05	0.9852	3.8996E-02	2.3178	1.6203E-03
BdbZIP59	0.5074	9.3747E-06	0.8578	2.4309E-04	0.2010	2.0313E-06	0.3017	1.5269E-05
BdbZIP60	32.3848	2.5835E-08	16.7123	4.8112E-09	13.9259	5.1685E-62	3.6281	4.5030E-08
BdbZIP61	0.2452	7.0831E-06	0.1240	2.6337E-07	0.0719	7.7444E-06	0.0907	1.3131E-06
BdbZIP62	3.3066	7.7431E-06	15.4792	1.6594E-08	1.2080	5.0262E-03	2.0035	1.2885E-05
BdbZIP63	0.1864	2.5799E-06	0.4694	2.8152E-04	0.0732	3.0375E-06	0.0825	3.3935E-08
BdbZIP64	0.1008	1.7233E-05	0.3872	3.5556E-03	0.1645	3.4574E-05	0.1758	5.6899E-05
BdbZIP65	0.6200	4.0293E-05	0.2340	3.9264E-04	1.3929	7.2453E-03	0.5738	4.8026E-07
BdbZIP66	0.1701	2.4441E-07	0.1420	4.4742E-08	0.3791	5.5966E-07	0.4863	2.9145E-06
BdbZIP67	0.2244	1.3981E-05	0.1145	2.5525E-06	0.1146	4.0929E-06	0.1022	1.0104E-06
BdbZIP68	0.2893	1.7549E-04	0.2321	2.5062E-04	0.3143	1.2125E-04	0.0851	1.1111E-05
BdbZIP69	0.8101	9.2526E-04	0.4812	7.1574E-05	0.5371	3.8509E-05	0.2657	2.0080E-06
BdbZIP70	0.5578	2.0584E-05	0.7278	4.2255E-01	0.1941	3.4248E-08	0.0906	3.2993E-07
BdbZIP71	0.0870	1.8396E-05	0.2686	1.7936E-04	0.4011	3.8808E-06	0.0674	3.6507E-06
BdbZIP72	4.9068	2.7326E-08	2.3017	1.4595E-04	1.5400	1.5722E-04	1.5163	4.7333E-04
BdbZIP73	0.3767	2.9773E-04	0.1482	6.1847E-05	0.8898	5.2531E-02	0.0767	3.7857E-05
BdbZIP74	0.6593	1.1802E-02	0.8162	1.5881E-01	0.7368	5.0015E-03	0.7400	3.0406E-02
BdbZIP75	0.1332	5.9805E-07	0.4596	4.7783E-04	0.8899	1.7581E-04	0.0981	9.6375E-08
BdbZIP76	6.5002	8.8379E-05	20.5104	4.1880E-05	3.5675	2.1729E-03	2.1794	2.5369E-03
BdbZIP77	0.5836	3.2639E-04	0.5403	1.2673E-04	0.5621	5.9624E-06	0.1802	2.0712E-07
BdbZIP78	0.6884	1.2107E-02	0.9648	4.5322E-02	0.2300	3.2803E-06	0.2709	8.9462E-06
BdbZIP79	0.5978	2.1321E-06	0.9485	1.5979E-02	0.2506	7.0199E-07	0.1353	4.1048E-09
BdbZIP80	0.0868	7.1311E-06	0.3081	5.1358E-04	0.2514	6.4220E-05	0.0933	8.1877E-05
BdbZIP81	0.4673	2.2923E-05	0.1208	1.3111E-06	0.5221	8.0789E-09	0.1056	1.4656E-08
BdbZIP82	0.4305	3.8987E-06	0.1141	1.2958E-06	0.6513	3.0869E-08	0.1009	1.3386E-05
BdbZIP83	3.0285	2.4673E-06	1.0556	3.4447E-05	0.3915	1.1922E-06	0.1921	1.6339E-05
BdbZIP84	0.9600	3.1568E-01	0.2286	9.0796E-06	0.4524	6.4310E-06	0.1267	4.0728E-07
BdbZIP85	0.2952	7.3545E-04	0.4259	1.3409E-02	0.1528	7.4059E-05	0.5651	7.2938E-03
BdbZIP86	0.4385	6.7428E-04	0.6367	2.0336E-01	0.3684	1.1741E-04	0.4570	7.1448E-04
BdbZIP87	1.0521	2.1689E-01	0.5514	4.3166E-02	1.2659	4.8029E-01	0.6206	6.6393E-03
BdbZIP88	0.1926	8.3473E-05	0.2660	2.9609E-04	0.0699	7.1175E-06	0.0689	1.2893E-05
BdbZIP89	0.7143	2.8968E-02	0.5036	1.9060E-02	0.5713	8.6751E-04	0.1137	2.5253E-05
BdbZIP90	4.8067	1.4163E-06	6.5218	1.4879E-06	0.8548	3.4698E-04	0.5782	7.3297E-05
BdbZIP91	1.0361	4.1143E-01	0.1928	7.8046E-05	0.8964	2.1518E-02	0.2002	1.1251E-04
BdbZIP92	2.2420	1.5472E-03	0.8162	4.0657E-02	0.4428	3.6153E-09	0.1032	8.5600E-11
BdbZIP93	0.3822	6.9639E-04	0.1990	4.0170E-06	0.5872	3.2543E-04	0.0906	1.2966E-05
BdbZIP94	1.6260	1.4398E-04	1.8722	3.4801E-07	0.6299	1.3295E-03	0.5272	2.6126E-04
BdbZIP95	1.0847	1.3307E-01	0.5523	5.0981E-03	0.2324	2.0636E-05	0.1898	5.7864E-06
BdbZIP96	0.6381	2.8938E-07	0.1733	1.9180E-07	0.0620	1.5894E-06	0.0886	1.7440E-06

**Treatments**	**SA**				**6-BA**			
	1h		6h		1h		6h	
**Genes**	fold change	p-value	fold change	p-value	fold change	p-value	fold change	p-value
BdbZIP1	0.0193	3.6970E-06	0.1142	1.7303E-06	0.0151	3.4601E-07	0.2230	8.5903E-06
BdbZIP2	0.3523	8.8314E-05	0.4369	3.0185E-04	0.2588	3.2346E-04	0.3053	5.2842E-04
BdbZIP3	1.0158	8.9616E-02	0.6189	5.9320E-04	1.4254	6.3575E-02	1.0005	3.6133E-01
BdbZIP4	0.4071	7.5959E-04	0.8312	9.0806E-03	0.2883	4.5058E-04	0.3596	1.8513E-04

BdbZIP5	0.5992	5.4756E-04	0.3022	1.8150E-04	0.3426	1.4830E-04	0.2600	1.3993E-04
BdbZIP6	0.8869	1.0889E-03	2.6872	6.2617E-04	0.1927	1.8415E-06	0.3274	1.6812E-04
BdbZIP7	4.0903	4.8224E-04	1.6163	4.7423E-02	2.0278	6.1117E-03	1.6249	2.0265E-02
BdbZIP8	1.9950	2.9920E-04	2.0535	3.4670E-02	0.8412	5.1562E-04	1.4723	4.1148E-02
BdbZIP9	0.5414	5.6278E-04	0.9198	6.2806E-02	0.2749	2.3533E-04	0.6796	2.1669E-02
BdbZIP10	4.0231	3.6719E-04	2.4204	2.0626E-03	1.5629	4.3253E-02	5.8701	1.0486E-04
BdbZIP11	0.8055	8.4932E-03	0.4721	1.1003E-03	0.5430	3.8065E-03	0.3714	2.8686E-03
BdbZIP12	0.9090	1.5811E-02	3.0175	1.2318E-03	0.8904	8.6255E-02	3.0119	4.8761E-05
BdbZIP13	0.7747	1.6737E-03	0.9064	1.2349E-02	0.3892	2.1630E-04	0.6249	5.6401E-03
BdbZIP14	3.4952	2.7186E-03	13.5028	4.6427E-05	3.2008	1.2061E-03	5.8763	2.0179E-04
BdbZIP15	0.5756	4.8309E-04	0.3654	1.1965E-05	0.3765	4.2962E-04	0.3780	1.2404E-04
BdbZIP16	0.8225	1.4851E-02	1.7759	1.1975E-02	0.8008	7.0644E-02	1.5659	3.0769E-03
BdbZIP17	0.7494	1.9300E-02	2.9718	2.2071E-03	0.4801	4.3993E-03	0.9232	4.3436E-01
BdbZIP18	3.9464	2.0832E-03	1.8486	1.2608E-02	2.5013	4.2842E-03	2.9882	3.1925E-04
BdbZIP19	1.7536	6.3525E-04	1.2156	5.4245E-02	1.1375	7.6867E-02	1.4960	7.9624E-03
BdbZIP20	1.2388	4.3697E-01	1.1268	4.7975E-01	0.8637	1.3478E-01	0.9437	4.8480E-01
BdbZIP21	1.0683	1.3769E-01	0.7724	4.0926E-02	0.9930	4.0535E-01	1.0104	3.2960E-01
BdbZIP22	4.3498	2.3744E-04	2.0517	2.1380E-03	2.6097	8.1534E-04	2.1946	5.6032E-04
BdbZIP23	2.0182	6.6729E-03	1.2819	1.6836E-01	1.8505	4.7729E-03	0.9590	4.6068E-01
BdbZIP24	0.2541	1.6649E-04	0.2925	1.0938E-04	0.1519	4.1782E-05	0.2555	8.9857E-04
BdbZIP25	1.0605	2.1384E-01	1.6219	7.2954E-02	1.1939	2.6465E-01	1.5146	3.0079E-02
BdbZIP26	0.6899	1.3943E-03	0.7453	5.9635E-04	0.4646	1.2543E-03	0.6051	5.4427E-03
BdbZIP27	5.3572	1.2958E-05	2.5741	3.5885E-04	2.7046	4.1465E-05	2.4927	6.8772E-05
BdbZIP28	1.8538	3.0756E-03	3.3026	3.4718E-04	0.9726	5.6879E-04	1.4439	6.3890E-04
BdbZIP29	5.9982	5.3280E-03	6.7714	2.0576E-04	5.9502	2.9406E-01	5.5015	7.4770E-03
BdbZIP30	1.0161	4.0663E-06	0.7336	1.4222E-04	0.7181	1.4031E-06	0.5229	1.2979E-06
BdbZIP31	0.5035	1.1773E-01	2.2943	1.7530E-02	0.4329	5.2713E-02	1.7668	7.6791E-03
BdbZIP32	3.9563	5.8396E-05	1.8717	2.0076E-04	1.4561	9.8138E-04	1.1950	3.0184E-03
BdbZIP33	2.0566	7.3219E-05	1.3471	1.3976E-03	1.0562	3.5741E-02	1.0812	1.3080E-01
BdbZIP34	3.1116	1.4852E-02	4.7712	1.2136E-01	0.8269	4.5801E-01	7.4881	5.9451E-02
BdbZIP35	2.0385	1.1409E-03	1.4115	5.0470E-05	1.5344	7.3671E-02	1.3543	2.3436E-05
BdbZIP36	0.9571	1.3569E-03	0.5181	1.4690E-02	0.9744	2.4953E-02	0.4850	3.9901E-03
BdbZIP37	1.2430	2.5415E-02	3.5312	1.7942E-03	1.8920	3.4387E-01	0.8516	3.3901E-03
BdbZIP38	0.8184	4.0406E-01	0.7338	2.4109E-05	0.6529	1.7048E-03	0.5105	1.6044E-01
BdbZIP39	0.7686	8.8485E-04	0.6349	1.4411E-03	0.6698	5.5952E-04	0.4156	3.2362E-03
BdbZIP40	3.0933	1.2180E-02	3.7425	1.2622E-02	2.6784	1.7931E-02	3.7582	1.6711E-03
BdbZIP41	1.3416	2.3346E-01	1.9017	4.0389E-04	0.5026	7.2022E-04	1.6995	1.1285E-03
BdbZIP42	0.3542	3.0499E-04	0.8127	1.2149E-02	0.4406	5.1397E-04	1.2379	2.5653E-02
BdbZIP43	5.5111	3.3640E-05	1.8314	1.5637E-02	1.3807	3.4014E-02	1.4225	3.5230E-02
BdbZIP44	0.3556	6.6887E-04	0.1209	1.2702E-04	0.3891	1.2449E-03	0.1140	1.3839E-04
BdbZIP45	1.2217	3.5575E-01	0.6469	5.8687E-03	0.8481	1.3713E-01	0.4953	4.2548E-04
BdbZIP46	0.8494	5.9672E-02	0.9474	2.1445E-01	0.5211	1.2906E-02	0.8204	2.5212E-01
BdbZIP47	1.6326	5.1833E-02	0.8002	3.5883E-02	1.5210	3.4780E-02	1.2915	4.1477E-02
BdbZIP48	2.5792	8.0338E-04	1.7816	2.6741E-03	1.5483	8.0034E-03	1.0761	4.6599E-02
BdbZIP49	1.0555	1.5463E-01	1.2763	1.9391E-01	1.6660	2.8172E-02	0.9677	4.5091E-01
BdbZIP50	0.1137	3.0159E-06	0.2773	3.5572E-03	0.1152	3.1557E-06	0.1967	6.3146E-05
BdbZIP51	1.9431	5.1133E-02	1.1106	4.8059E-01	0.3257	5.2429E-04	1.5244	2.6230E-02
BdbZIP52	5.4004	1.6047E-04	4.2233	4.4263E-05	4.3854	5.1692E-05	9.7196	2.4671E-06
BdbZIP53	0.8887	2.7987E-02	0.6212	3.0778E-03	0.5738	6.8276E-03	0.3704	2.2047E-03
BdbZIP54	0.8291	1.3170E-02	0.6234	5.8756E-03	0.8781	1.7366E-01	0.3728	9.1990E-04
BdbZIP55	1.1667	1.6246E-01	1.9915	1.6018E-04	0.4744	1.3829E-05	1.1401	6.1522E-03
BdbZIP56	0.4775	2.8630E-03	1.2463	2.6690E-01	0.4371	3.6496E-03	0.9841	3.9868E-01
BdbZIP57	1.3043	4.7500E-01	2.2471	4.3341E-03	1.5599	1.7572E-02	1.5378	1.9295E-02
BdbZIP58	1.1084	1.8266E-01	1.1221	4.7894E-01	1.3221	8.4131E-02	0.8384	2.4834E-01
BdbZIP59	0.9028	5.8003E-02	1.2148	3.4557E-01	0.7798	5.5300E-02	2.0272	4.3358E-03
BdbZIP60	0.1589	5.9504E-05	0.0524	6.8866E-06	0.6659	2.0692E-03	0.0530	1.4202E-05

BdbZIP61	5.2149	2.2482E-04	2.3152	3.8754E-03	1.3599	6.4670E-02	2.2309	3.6248E-03
BdbZIP62	1.1691	1.7043E-01	2.4661	3.0575E-03	1.1400	2.4735E-01	3.7905	2.0405E-05
BdbZIP63	0.6024	3.0804E-03	0.1537	6.9451E-05	0.4377	2.8879E-03	0.1034	5.5778E-05
BdbZIP64	0.9186	5.6421E-02	2.7074	1.4942E-03	0.4732	1.3889E-03	3.2870	3.7215E-04
BdbZIP65	1.2921	4.5846E-01	1.0877	4.0196E-01	0.9910	3.8491E-01	0.9429	4.5549E-01
BdbZIP66	4.8028	2.7163E-04	3.2290	9.6637E-04	1.9115	1.5900E-02	2.2285	5.1702E-03
BdbZIP67	0.8431	9.3828E-03	0.8951	1.7487E-02	0.3516	3.9993E-04	0.3655	1.4303E-04
BdbZIP68	0.2184	4.9125E-05	0.2306	1.5331E-04	0.3683	1.2404E-03	0.4795	5.1883E-03
BdbZIP69	0.9739	4.8302E-02	1.0511	3.3705E-01	0.6122	1.9211E-02	0.8755	3.1623E-01
BdbZIP70	1.2729	4.8039E-01	0.9882	1.5248E-01	0.7339	3.8722E-02	0.6695	1.8533E-02
BdbZIP71	0.9664	1.0502E-02	0.4650	7.3475E-04	0.6028	2.0204E-03	0.2683	4.5961E-04
BdbZIP72	0.9039	1.4510E-02	4.1409	6.2391E-04	0.9134	1.7431E-01	3.7993	1.0949E-04
BdbZIP73	2.9249	4.4404E-03	2.0957	1.3338E-03	1.1152	2.8645E-01	1.8250	1.4800E-03
BdbZIP74	0.4066	7.6161E-04	0.4257	2.2109E-03	0.4516	3.4595E-03	0.4550	6.2903E-03
BdbZIP75	1.0563	1.1481E-01	0.0457	9.9715E-07	0.4334	1.4380E-03	0.0224	4.8647E-06
BdbZIP76	3.4543	1.4150E-03	8.8511	1.0019E-05	2.0879	2.6865E-03	5.3987	8.3547E-06
BdbZIP77	1.1824	2.7312E-01	0.5895	5.0634E-04	0.6690	3.1933E-03	0.6637	4.6374E-03
BdbZIP78	1.3903	2.1925E-04	1.2787	1.3928E-03	0.7496	4.2477E-02	1.3692	6.6922E-04
BdbZIP79	0.3418	1.1727E-01	0.8099	7.1492E-02	0.2321	3.1706E-02	0.6637	5.1902E-03
BdbZIP80	0.4691	1.3075E-03	0.5640	3.0882E-03	0.5800	1.0023E-02	0.4816	7.7278E-03
BdbZIP81	0.2738	4.1081E-04	0.1611	1.6711E-04	0.1941	5.2560E-04	0.2273	7.0048E-04
BdbZIP82	2.0800	1.2005E-02	0.6478	6.5726E-03	1.2857	1.0866E-01	0.5258	1.2082E-02
BdbZIP83	2.4883	9.7881E-03	2.8428	1.8495E-03	2.6711	2.4763E-03	1.7700	1.4129E-02
BdbZIP84	5.5889	1.0013E-04	1.1274	4.8860E-01	2.4480	2.3715E-03	2.0102	8.6948E-04
BdbZIP85	0.9758	1.5899E-02	1.4901	4.5393E-03	0.9581	2.9747E-01	0.9950	2.9556E-01
BdbZIP86	1.3977	3.3095E-01	0.9475	2.9200E-02	0.7919	5.2960E-02	0.6099	6.8680E-03
BdbZIP87	0.1710	2.6711E-06	1.0180	1.2153E-01	0.1266	1.2119E-05	0.8828	3.0911E-01
BdbZIP88	5.3286	2.1401E-04	4.0358	2.8954E-04	3.8110	3.1248E-04	2.9849	4.2102E-05
BdbZIP89	1.3078	4.2139E-01	0.7601	2.6484E-02	0.9167	2.6466E-01	0.7001	7.0385E-02
BdbZIP90	0.6125	2.8241E-03	1.2714	2.1747E-01	0.3827	7.8804E-04	1.0724	1.7188E-01
BdbZIP91	0.7911	1.3343E-02	1.1616	4.0063E-01	0.5413	2.0846E-03	0.9308	4.2455E-01
BdbZIP92	1.2042	3.6205E-01	2.9830	3.3491E-03	3.0713	7.8808E-04	1.4533	4.2464E-02
BdbZIP93	0.6317	1.8438E-03	0.4379	1.9465E-05	0.4963	1.1492E-04	0.3235	1.5485E-05
BdbZIP94	5.3633	3.6513E-06	3.4751	5.0959E-07	2.3962	8.9465E-05	3.2380	1.8408E-05
BdbZIP95	2.7639	2.5170E-03	2.6946	1.7160E-04	1.5175	1.6647E-02	2.9393	2.3428E-03
BdbZIP96	2.3708	7.8670E-04	2.4223	1.0745E-03	3.7808	1.6991E-05	4.0411	3.7751E-05

**Treatments**	**ABA**				**MeJA**			
	1h		6h		1h		6h	
**Genes**	fold change	p-value	fold change	p-value	fold change	p-value	fold change	p-value
BdbZIP1	0.0296	6.8533E-06	0.2646	7.1050E-07	0.9106	4.1761E-06	14.7960	1.3874E-03
BdbZIP2	0.4058	6.8409E-02	3.1492	1.9280E-03	1.6693	8.1657E-04	1.6862	3.5979E-04
BdbZIP3	0.3220	1.2333E-01	0.9224	3.9394E-03	1.6852	3.2468E-02	2.9047	3.3755E-02
BdbZIP4	0.4120	1.2909E-01	16.6822	9.6952E-04	3.4469	1.9963E-01	19.9388	7.8194E-04
BdbZIP5	0.5455	3.5161E-01	3.6919	2.1972E-02	3.1550	4.2573E-02	3.5543	6.1480E-03
BdbZIP6	0.5990	3.7403E-01	0.3710	1.2658E-05	0.4462	5.2352E-05	1.2299	1.0231E-04
BdbZIP7	0.4878	3.5397E-01	3.2377	2.0321E-03	4.3790	2.3599E-01	5.5980	1.0298E-01
BdbZIP8	0.6663	1.5512E-01	13.3749	4.8101E-01	3.3960	1.1922E-02	8.9320	2.1716E-02
BdbZIP9	0.7042	4.3567E-06	7.5643	5.3591E-03	2.2815	6.8718E-06	6.0135	5.9467E-04
BdbZIP10	1.0891	4.9116E-03	4.0613	6.1013E-02	8.0761	1.0586E-02	10.2381	3.4191E-02
BdbZIP11	0.5722	1.9853E-01	5.0651	3.4156E-01	3.3911	1.1078E-01	4.4360	1.5739E-02
BdbZIP12	0.2930	4.0643E-03	4.5648	7.1674E-02	2.1439	2.8694E-03	11.7550	8.3325E-03
BdbZIP13	0.4249	9.4114E-02	4.2310	7.2816E-02	3.3344	1.2649E-01	4.7688	4.4831E-02
BdbZIP14	0.4957	3.6892E-01	6.7795	6.9606E-03	1.4515	1.5999E-04	13.3401	3.0722E-03
BdbZIP15	0.3775	3.8501E-02	3.5859	1.8549E-02	2.1842	8.1698E-03	3.9308	3.4919E-03
BdbZIP16	0.4926	3.9788E-01	9.8620	1.0365E-02	1.9727	6.6429E-03	9.4580	1.2009E-02
BdbZIP17	0.3651	2.3584E-02	7.5602	4.3323E-02	0.8314	5.1172E-05	9.8618	1.7408E-02

BdbZIP18	0.4834	3.5341E-01	11.0497	1.9910E-03	6.3583	1.0914E-02	11.7664	4.4511E-03
BdbZIP19	0.5380	3.5367E-01	2.0867	1.3050E-03	4.6457	2.0247E-01	11.3791	1.7516E-02
BdbZIP20	0.5228	4.0727E-01	5.6125	4.1519E-01	6.5223	3.5359E-03	8.3601	5.3335E-02
BdbZIP21	0.7429	2.8909E-02	3.4391	2.7174E-02	3.2179	1.1924E-01	8.2652	9.2955E-02
BdbZIP22	0.4489	1.5231E-01	3.9207	3.2054E-02	5.1827	4.7152E-02	7.3250	2.1504E-01
BdbZIP23	1.0871	1.0291E-02	10.8048	4.2816E-03	5.6056	7.2757E-02	14.2985	1.0001E-02
BdbZIP24	0.3742	4.6534E-02	3.4609	1.0110E-02	2.5389	1.1355E-02	3.2396	1.1362E-03
BdbZIP25	0.4930	4.2809E-01	0.7763	2.1876E-04	0.9294	7.5126E-04	1.2516	3.8657E-04
BdbZIP26	0.4251	1.1415E-01	3.4992	1.8066E-02	3.1261	6.1710E-02	5.2030	7.3408E-02
BdbZIP27	0.4625	1.8378E-01	3.9727	2.6980E-03	4.3153	1.3520E-01	7.3784	2.4657E-01
BdbZIP28	0.2728	1.3007E-02	4.0267	7.1108E-03	1.3966	9.4048E-03	5.6308	5.2556E-03
BdbZIP29	0.5789	6.5742E-03	16.9281	1.2365E-02	4.0773	1.4019E-04	11.1410	9.3582E-02
BdbZIP30	0.5086	2.2137E-01	3.1039	9.2821E-04	3.4317	4.4839E-01	3.4111	1.3152E-02
BdbZIP31	0.1724	4.9135E-01	2.1249	7.1790E-03	1.7586	1.0096E-01	7.5223	1.4778E-03
BdbZIP32	1.6101	7.5919E-04	6.5752	5.7761E-04	3.1595	1.2026E-03	3.9292	2.1599E-01
BdbZIP33	0.8541	4.4109E-05	4.4589	3.8542E-02	3.1395	5.2638E-04	6.9320	1.0591E-04
BdbZIP34	1.1348	2.3273E-02	14.2998	9.3667E-02	1.6941	9.4413E-02	4.2109	4.1170E-01
BdbZIP35	0.6596	1.5405E-03	4.5363	8.2688E-04	4.3390	2.6315E-03	6.9747	1.7912E-02
BdbZIP36	0.5628	2.2356E-02	6.6901	7.3560E-02	6.5028	1.7575E-01	7.6066	3.1367E-01
BdbZIP37	0.0926	2.6398E-01	0.3062	6.2089E-02	3.0525	3.7825E-03	1.3538	1.9175E-01
BdbZIP38	0.6790	3.4575E-04	4.9733	5.3914E-05	3.5065	8.9965E-02	6.4221	2.4097E-04
BdbZIP39	0.6672	2.3020E-02	2.2089	2.6196E-01	3.3815	9.9175E-02	3.2406	3.8722E-01
BdbZIP40	0.8095	1.1792E-01	10.3123	2.2445E-03	2.3846	1.2881E-01	15.4723	4.9362E-03
BdbZIP41	0.4788	3.2649E-01	0.8930	3.1098E-05	2.5561	4.7676E-03	8.1182	7.4575E-02
BdbZIP42	0.2019	9.0799E-04	0.7049	1.7696E-05	5.2093	1.2303E-02	11.2698	7.6100E-02
BdbZIP43	0.3884	6.7047E-02	6.0178	2.8740E-01	3.8406	3.8751E-01	6.9905	4.0090E-01
BdbZIP44	0.5789	1.7454E-01	6.1251	1.3214E-01	2.3053	2.9469E-03	0.6991	5.1712E-06
BdbZIP45	0.6246	1.1568E-01	6.1420	2.5000E-01	5.6122	3.7972E-02	4.6514	4.1926E-02
BdbZIP46	0.4141	1.1822E-01	4.4129	1.2476E-01	3.4064	2.0715E-01	6.8409	4.2833E-01
BdbZIP47	0.4151	1.8304E-02	2.3621	1.4381E-04	1.7556	8.0946E-04	5.5138	1.4569E-01
BdbZIP48	0.9234	4.7653E-03	7.3378	4.8182E-03	1.5507	2.1651E-04	4.3198	6.7825E-03
BdbZIP49	0.5528	2.3273E-01	9.4895	4.5262E-03	3.2294	5.6926E-02	5.1645	9.5160E-02
BdbZIP50	0.1902	3.3428E-03	0.2807	3.6524E-05	1.8077	3.6462E-03	3.1846	4.1399E-03
BdbZIP51	0.5298	3.5690E-01	0.8661	5.0647E-05	0.6850	9.3485E-05	6.3299	3.3208E-01
BdbZIP52	0.3786	6.3139E-02	13.9377	5.6151E-04	1.7077	2.0558E-03	27.3613	1.2867E-04
BdbZIP53	0.5979	1.9395E-01	6.4499	1.4128E-01	3.8522	4.0321E-01	6.0022	2.6952E-01
BdbZIP54	0.3399	2.4445E-02	2.7424	1.2379E-03	1.7707	1.2026E-03	2.5892	3.0091E-03
BdbZIP55	0.7593	1.0026E-05	7.6990	2.1822E-03	2.3409	5.3460E-04	6.7828	4.8913E-01
BdbZIP56	0.4507	2.2408E-01	4.8639	2.3529E-01	2.1692	6.8035E-03	6.7536	4.8989E-01
BdbZIP57	0.4374	1.7656E-01	6.6253	8.5269E-02	2.8962	1.7554E-02	7.4611	1.8036E-01
BdbZIP58	1.1104	4.1205E-03	3.1423	2.6486E-02	2.6330	3.9918E-02	4.1725	3.5449E-02
BdbZIP59	0.6167	1.2703E-01	5.3902	4.9179E-01	3.5521	1.9854E-01	5.3444	6.5104E-02
BdbZIP60	1.5420	1.6675E-04	1.2496	9.4548E-06	3.1231	1.3231E-02	0.2009	6.5600E-07
BdbZIP61	0.3146	8.7901E-03	4.8604	2.2777E-01	2.5723	1.2688E-02	6.5816	4.6774E-01
BdbZIP62	0.3152	1.9610E-02	1.8660	7.6848E-05	1.7724	9.4991E-04	2.1512	1.5935E-04
BdbZIP63	0.6542	7.0270E-02	2.5194	2.0623E-03	3.3491	1.5895E-01	3.4168	4.3409E-02
BdbZIP64	0.2723	3.4436E-02	7.1878	1.3440E-01	1.6831	1.0690E-02	13.0488	2.3541E-02
BdbZIP65	0.4395	1.5918E-01	1.6658	3.8270E-04	3.3003	9.0750E-02	5.1543	6.1556E-02
BdbZIP66	0.5530	3.4389E-01	1.7878	2.8210E-03	1.0349	8.0347E-04	2.8069	2.9870E-03
BdbZIP67	1.1026	1.0234E-03	11.8094	2.7281E-03	2.9779	2.5221E-02	3.2332	3.2145E-03
BdbZIP68	0.5910	1.8181E-01	5.5128	4.1682E-01	3.3683	1.5990E-01	3.2284	3.8532E-03
BdbZIP69	0.3164	9.4087E-02	2.7347	4.6577E-02	2.0661	4.4024E-02	4.6100	1.4005E-01
BdbZIP70	0.5625	1.9227E-01	5.5890	3.3683E-01	3.9974	4.8084E-01	6.0522	2.3423E-01
BdbZIP71	0.2432	2.6819E-03	3.2081	1.4887E-02	5.5068	3.0013E-02	16.1029	1.1212E-03
BdbZIP72	0.2818	4.0182E-03	2.4741	2.8377E-03	1.8008	1.0375E-03	18.2457	3.0435E-04
BdbZIP73	0.3319	1.4986E-02	4.7063	1.9781E-01	6.1261	1.5577E-02	7.2299	3.4333E-01

BdbZIP74	0.4158	1.6064E-01	0.6477	7.4186E-05	2.1438	1.5314E-02	1.8985	1.1446E-03
BdbZIP75	0.4566	1.7120E-01	0.5352	7.8446E-06	4.0377	4.4706E-01	0.2540	1.5134E-06
BdbZIP76	0.3834	1.0001E-01	9.5170	1.3355E-02	2.1555	2.1566E-02	16.7409	1.9680E-03
BdbZIP77	0.8026	1.2781E-02	6.9295	4.1568E-02	4.5280	1.3764E-01	5.5931	1.3307E-01
BdbZIP78	0.3167	7.0257E-05	8.1180	4.9211E-05	2.8131	2.6716E-02	10.2024	2.0244E-02
BdbZIP79	0.4487	5.8561E-03	5.3345	2.7343E-03	2.2282	1.3475E-02	9.4838	2.5405E-02
BdbZIP80	0.3658	3.4962E-02	4.9640	2.5748E-01	6.5473	3.5988E-03	3.5002	4.2015E-04
BdbZIP81	0.5026	4.7065E-01	4.2727	1.0378E-01	5.0030	1.0034E-01	3.9977	1.1824E-02
BdbZIP82	0.8434	1.4257E-02	0.9341	8.7463E-05	6.6512	9.8264E-03	4.0595	8.2133E-03
BdbZIP83	0.4889	4.1175E-01	2.3793	3.1633E-03	3.7984	3.8122E-01	8.3475	1.3686E-01
BdbZIP84	1.1487	8.8958E-04	6.8821	1.3186E-01	4.4119	2.4765E-01	0.8778	7.9101E-05
BdbZIP85	0.5033	4.7660E-01	7.4411	8.2498E-02	4.8018	1.5080E-01	5.7558	1.7109E-01
BdbZIP86	0.5506	2.8078E-01	4.2750	5.2669E-02	4.2549	3.9281E-01	5.8325	1.8748E-01
BdbZIP87	0.1700	7.0456E-04	9.3089	3.9534E-03	1.1293	1.9648E-04	9.9674	5.3493E-03
BdbZIP88	0.8520	3.6617E-03	3.9565	2.2140E-02	3.4231	8.1464E-02	4.6934	1.7846E-02
BdbZIP89	0.6435	4.4395E-02	4.3451	4.5804E-02	3.7848	3.4779E-01	4.7350	3.8889E-02
BdbZIP90	0.2517	3.3215E-03	3.9207	4.1062E-02	1.5305	1.8361E-03	6.0770	2.5879E-01
BdbZIP91	0.4140	1.3534E-01	7.9307	5.1253E-02	2.9489	4.9573E-02	9.5818	3.4752E-02
BdbZIP92	0.4546	2.3591E-01	3.5986	1.6465E-02	3.0389	4.2701E-02	4.2917	1.6649E-02
BdbZIP93	0.5482	3.5827E-01	2.9487	3.6950E-03	3.6473	2.8255E-01	3.7416	8.0503E-03
BdbZIP94	0.5799	6.2236E-02	5.0832	3.1084E-01	2.8944	4.8374E-03	4.0051	3.1260E-03
BdbZIP95	0.3950	1.2819E-01	3.3993	2.8180E-02	2.7697	6.4883E-02	7.1210	4.0629E-01
BdbZIP96	2.3133	4.7920E-05	2.8534	3.6544E-03	15.4243	3.7002E-05	4.6769	3.3051E-02

Red colors means the up-regulated genes and the green colors means the down-regulated genes.

Table S11 BdbZIPs in models.

modules	BdbZIPs
Module	BdbZIP1, 60, 90
Module 02	BdbZIP29, 44, 51, 61, 71
Module	BdbZIP9, 15, 43
Module	BdbZIP53
Module	BdbZIP45
Module	BdbZIP58
Module	BdbZIP57
Module	BdbZIP69
Module	BdbZIP41
Module	BdbZIP31, 72
Module	BdbZIP2
Module	BdbZIP75
Module	BdbZIP54
