# Supplementary figures and images for: Genome-wide evolutionary characterization and analysis of bZIP transcription factors and their expression profiles in response to multiple abiotic stresses in Brachypodium distachyon
Source: BMC Genomics. 2015 Mar 22;16(1):227. doi: 10.1186/s12864-015-1457-9 (PMC4393604; doi:10.1186/s12864-015-1457-9)

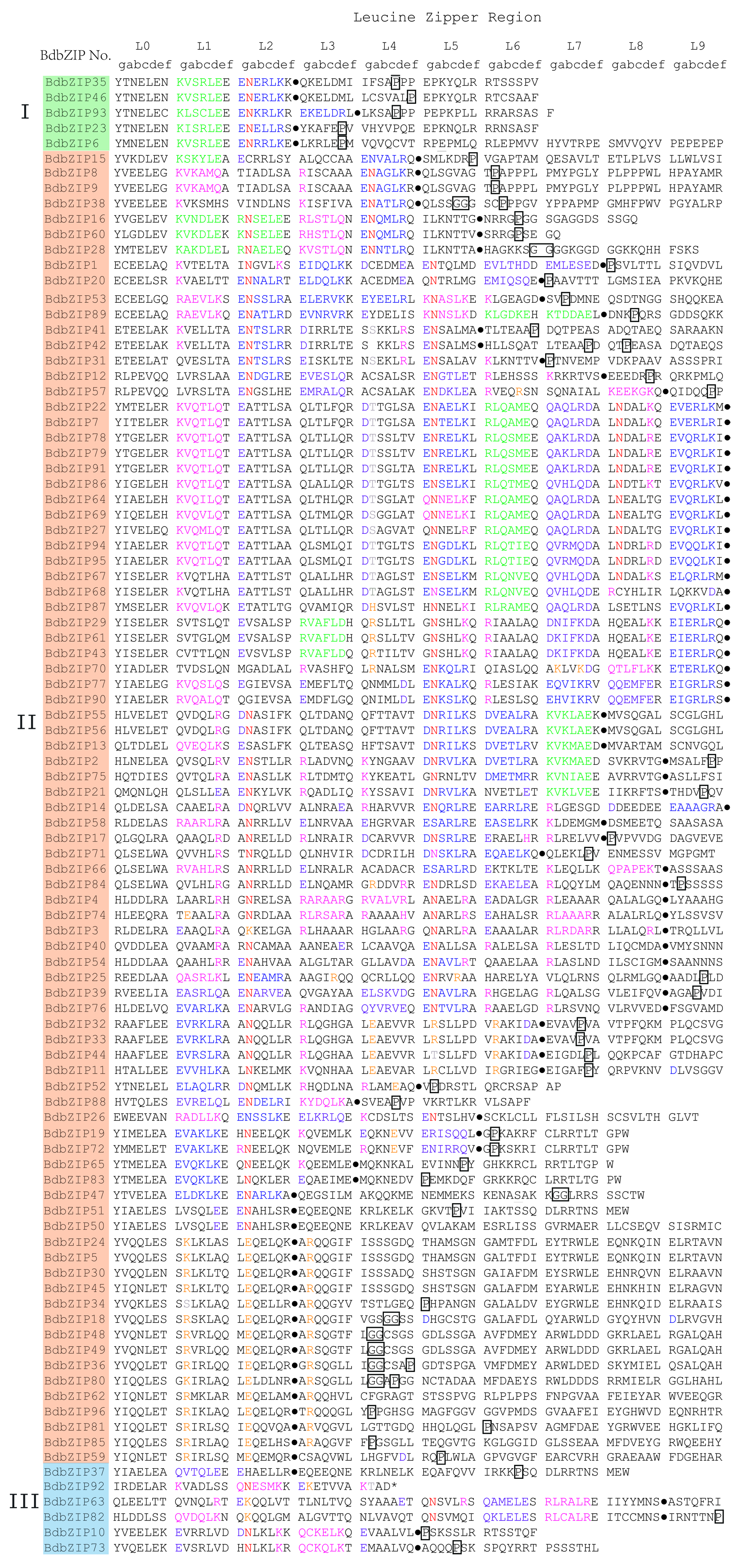

Supplement: Additional file 3: Figure S2. — The leucine zipper regions. BdbZIPs were classified as three major sub-families according to the predicted dimerization specificity. BdbZIPs in Sub-family I are homo-dimerization specific. BdbZIPs in Sub-family II are homo-hetero-dimerization, and BdbZIPs in sub-family III are hetero-dimerization specific. The leucine zipper regions are divided into heptads (gabcdef) from L0 to L9 to display the potential g↔ e' pairs. Based on the electrostatic charges at the g and e positions, the g↔e pairs were grouped into 4 types, which were displayed with 4 different colors: the frequency of the attractive basic-acidic pairs (+/−) was displayed with green color, attractive acidic-basic pairs (−/+) was displayed with blue color, repulsive acidic pairs (−/−) was displayed with purple color, and repulsive basic pairs (+/+) was displayed with pink color. If single amino acid at the positions e or g is charged, the residue is colored pink for basic amino acid and purple for acidic amino acid. If a or d position is polar, it is colored grey and if either is charged, it is colored orange. Asparagines at a position are colored red. The prolines and glycines are boxed to indicate a potential break in α-helix. The predicted C-terminal boundary is denoted by the symbol ●. [file 12864_2015_1457_MOESM3_ESM.tiff]

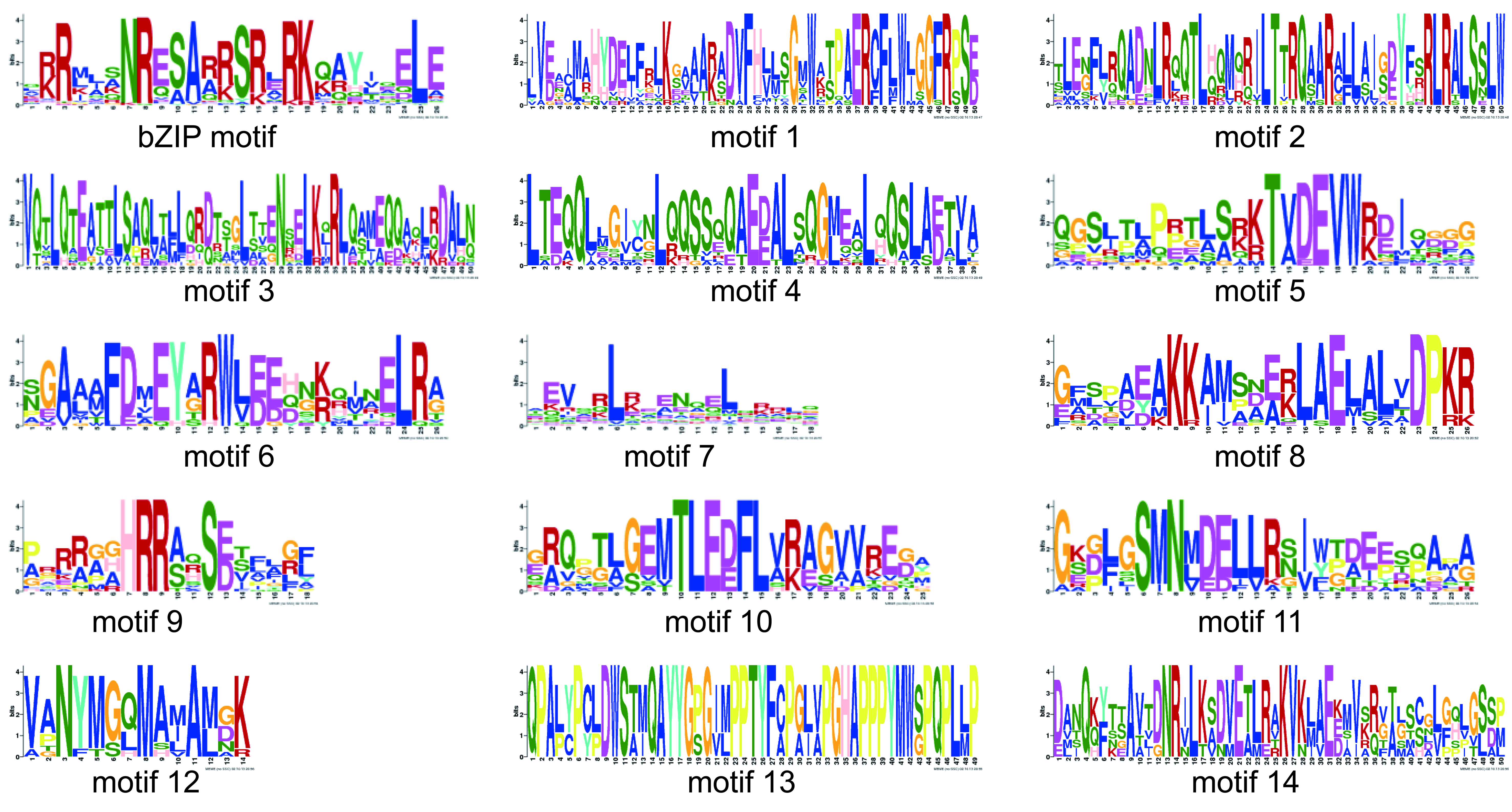

Supplement: Additional file 4: Figure S3. — The additional conserved motifs of BdbZIP proteins predicted by MEME. [file 12864_2015_1457_MOESM4_ESM.tiff]

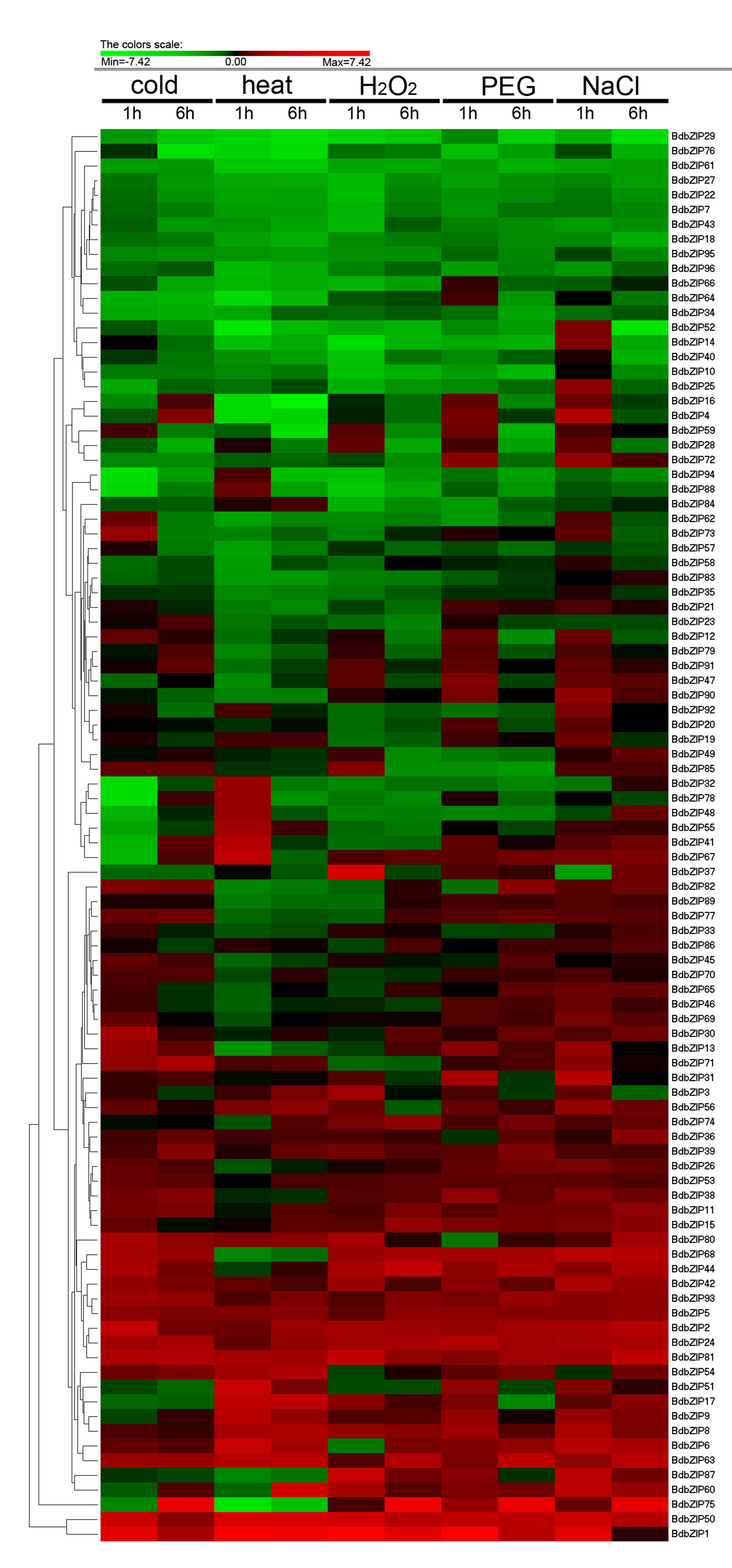

Supplement: Additional file 5: Figure S4. — Exression profiles of BdbZIP genes differentially expressed under environmental factors (cold, heat, H2O2, PEG, NaCl). [file 12864_2015_1457_MOESM5_ESM.tiff]

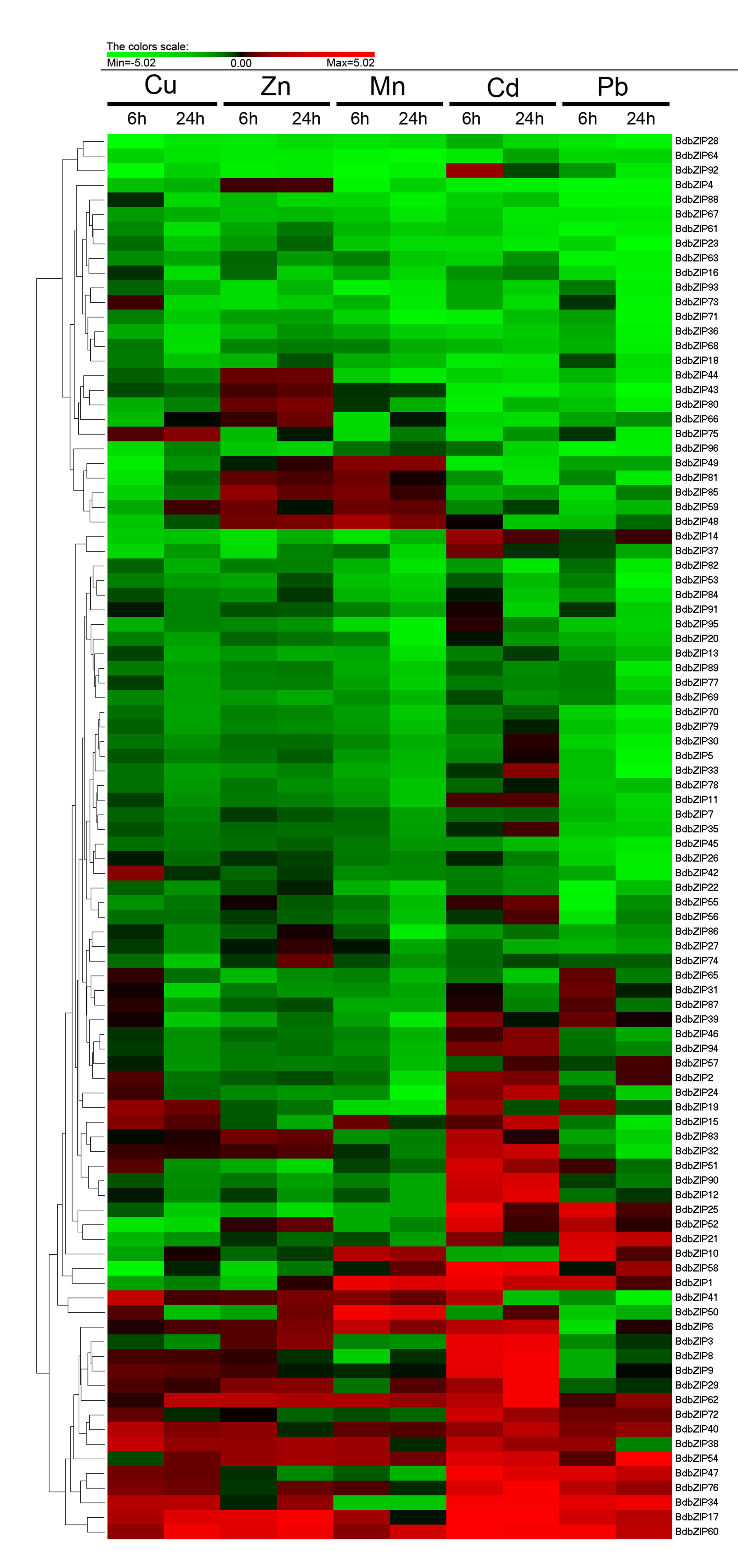

Supplement: Additional file 6: Figure S5. — Exression profiles of BdbZIP genes differentially expressed under heavy metal factors (Cu, Zn, Mn, Cd, Pb). [file 12864_2015_1457_MOESM6_ESM.tiff]

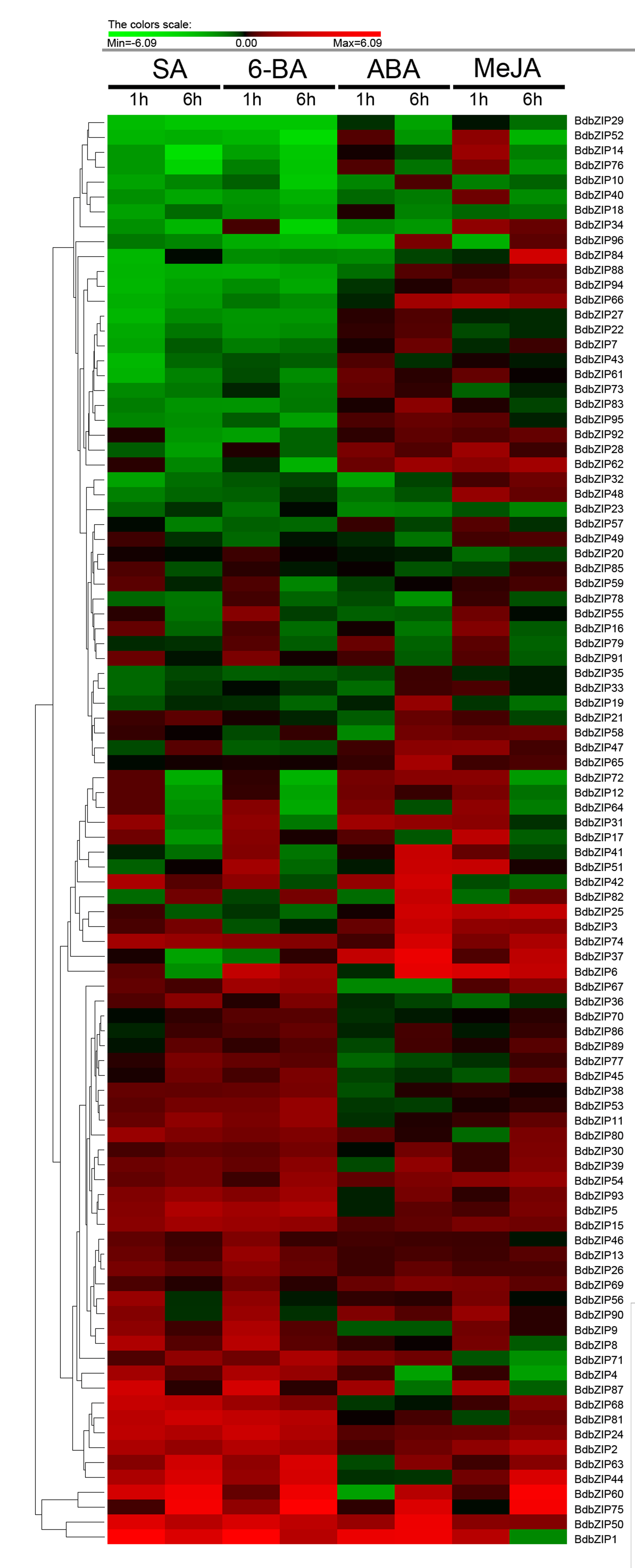

Supplement: Additional file 7: Figure S6. — Exression profiles of BdbZIP genes differentially expressed under hytohormones (SA, 6-BA, ABA, MeJA). [file 12864_2015_1457_MOESM7_ESM.tiff]
